# A benchmark study of *k*-mer counting methods for high-throughput sequencing

**DOI:** 10.1093/gigascience/giy125

**Published:** 2018-10-22

**Authors:** Swati C Manekar, Shailesh R Sathe

**Affiliations:** Department of Computer Science and Engineering, Visvesvaraya National Institute of Technology, Nagpur 440 010, India

**Keywords:** *k*-mer counting, high-throughput sequencing, disk-based counting, in-memory counting, hash table, sorting

## Abstract

The rapid development of high-throughput sequencing technologies means that hundreds of gigabytes of sequencing data can be produced in a single study. Many bioinformatics tools require counts of substrings of length *k* in DNA/RNA sequencing reads obtained for applications such as genome and transcriptome assembly, error correction, multiple sequence alignment, and repeat detection. Recently, several techniques have been developed to count *k*-mers in large sequencing datasets, with a trade-off between the time and memory required to perform this function. We assessed several *k*-mer counting programs and evaluated their relative performance, primarily on the basis of runtime and memory usage. We also considered additional parameters such as disk usage, accuracy, parallelism, the impact of compressed input, performance in terms of counting large *k* values and the scalability of the application to larger datasets.We make specific recommendations for the setup of a current state-of-the-art program and suggestions for further development.

## Introduction

*k*-mer counting is an important step in many bioinformatics applications that are used to analyze sequencing data. Recently, several tools and techniques have been developed to count the frequency of *k*-length substrings (*k*-mers) in reads generated from high-throughput sequencing [1]. *k*-mer counting involves counting the number of substrings that have length *k* in a string *S*, or a set of strings, where *k* is a positive integer.

Let *∑* = {*A, C, G, T, N*} denote the alphabet of DNA nucleotide sequences, where *N* denotes an undetermined character. A read *r* is a sequence of nucleotides over the alphabet *∑*. In a sequence dataset, different reads can contain the same sequence of nucleotides. Let *R* denote a dataset having *n* reads, such that *R* = {*r*_i_; 1 ≤ *i* ≤ *n*}. Consider an example dataset *R* containing three reads each of length 6, *R* = {*ACGTTA, ACGTTA, ACGTTT*}, having two sequences {*ACGTTA, ACGTTT*}. For *k* = 4, there are nine 4-mers (three in each read): {*ACGT, CGTT, GTTA, ACGT, CGTT, GTTA, ACGT, CGTT, GTTT*}. On counting, four unique 4-mers are obtained, which can be represented along with their counts in a tab-delimited format, e.g., {*ACGT* 3, *CGTT* 3, *GTTA* 2, *GTTT* 1} [2]. *k*-mer counting has applications in genome assembly, e.g., using the overlap layout consensus approach [3–5] or the de Bruijn graph approach [6–9]. Errors in sequencing reads are corrected to improve the quality of genome assemblies. Error correction based on the *k*-mer spectrum approach [10–13], or multiple sequence alignment approach [14], relies on counting and keeping track of *k*-mers. *k*-mer counting is also used (for fast distance estimation) to create multiple protein sequence alignments [15]. In *de novo* genome projects, genomic characteristics such as genome size, repeat structure, and rate of heterozygosity are estimated by analyzing the *k*-mer frequency distribution in a sequencing dataset [16]. Statistical analysis can reveal the high-frequency *k*-mers in a given dataset, which are used as “seeds” to build a set of repeat families [17]. ReAS [18] also uses these high-frequency *k*-mers (obtained from *k*-mer repeat libraries) in the reconstruction of transposable elements. Identifying repeated sequences is a main step in genome analysis and annotation. *De novo* repeat identification techniques such as RAP [19], FORRepeats [20], and that described by Healy et al. [21] use *k*-mer occurrences to find candidate regions (i.e., to identify repeated regions). Tallymer [22] also uses *k*-mer frequencies to annotate repetitive plant genomes. Quantitative features of complex repetitive DNA have been studied in several genomes by determining the distribution of frequencies of long *k*-mers (20 ≤ *k *≤ 100) [23]. *k*-mer counts are also used to infer the genotypes of known variants [24].

Although *k*-mer counting is simple and straightforward, it becomes challenging when billions of reads generated by next-generation sequencing (NGS) techniques must be processed using reasonable amounts of memory and in minimal time. A naive approach for *k*-mer counting is to use a dictionary, with *k*-mers as keys and their counts as values. However, when there are billions of such input reads, computer memory is often overwhelmed. Approaches to *k*-mer counting proposed so far have mainly targeted memory-efficient and time-efficient solutions. One way to achieve memory efficiency is to represent the string data using unsigned integers. Disks are always cheaper than memory. Therefore, many researchers have focused on using the disk-based/external memory/out-of-core approach, as opposed to the in-memory/internal memory approach, to reduce memory usage.

Here, we review and comparatively evaluate *k*-mer counting approaches for high-throughput sequencing data. The main aim is to provide a general set of benchmarks and assessment metrics of some popular *k*-mer counters, but we also cover experimental analysis of *k*-mer counting tools to provide thorough insight for beginners and consultants alike. Perez et al. [25] studied various *k*-mer counting tools for two values of *k*, i.e., 31 and 55, on a single dataset. Building on this, we evaluate the performance of several of the latest and competitive tools on different datasets of varying sizes, primarily focusing on runtime and memory usage. However, we also consider several other parameters including scalability to larger values of *k*; scalability to larger datasets; the impact (on runtime, memory, disk, and central processing unit [CPU] utilization) of compressed inputs, i.e., gzip and bzip2 (multiple compressed FASTA/FASTQ input files); scaling properties (speedup) with respect to thread number; accuracy; and maximum temporary disk usage. Scalability is measured in terms of runtime, memory, disk usage, and CPU utilization.

Time, CPU, and memory are bounded (limited) resources, whereas the disk can be considered a plentiful resource. Disk-based approaches may additionally use hundreds of gigabytes of disk space for large datasets such as the human genome. Hence, we record the maximum disk utilization of all disk-based approaches. Disk-based approaches achieve very high efficiency with marginal increase in input/output (I/O) costs.

Advancements in NGS technologies mean that long reads are generated in bioinformatics. Among other advantages, such long reads are excellent for resolving complex RNA-splicing patterns from cDNA libraries [26], resolving repetition in genome assemblies, and facilitating better resolution of structural variants present in DNA samples and genomic repeat content [26, 27]. However, longer Illumina reads suffer from lower accuracy [27]. Large *k* values (up to 200) help improve the accuracy of longer Illumina reads (particularly of repeat-overlapping reads) and contig assemblies [28]. Empirically, the best assemblies (i.e., those without misassembly errors) and the highest N50 (only when there is sufficiently high coverage) are obtained at an optimal choice of *k*, which seems to be larger values of *k* [29, 30]. Hence, we evaluate the performance of different *k*-mer counting tools at large *k* values.

## Overview of *k*-mer Counting Approaches

*k*-mer counting tools can be categorized based on the approach and data structures they use, as shown in Table 1. Comprehensive information about each *k*-mer counting tool is given in Supplementary Table S1.

**Table 1:** Ontology of *k*-mer counting approaches

Approach for *k*-mer counting	Disk-based	In-memory
Hash table	Gerbil [31], MSPKmerCounter [32], DSK [33]	Squeakr [34], Jellyfish [35], BFCounter [36]
Sorting	KMC3 [37], GenomeTester4 [38], KMC2 [39], KAnalyze [40], KMC1 [41]	Turtle [42]
Burst tries	-	KCMBT [43]
Enhanced suffix array	-	Tallymer [22]

### *k*-mer counting using the sorting approach

The sorting approach works by sorting all *k*-mers extracted from each read. Thus, *k*-mer frequencies can be easily counted because, after sorting, repeating *k*-mers lay at adjacent positions in the sorted list.

GenomeTester4 (GListMaker) [38] uses the sorting approach and works as follows: (i) in the reading phase, temporary arrays are used to gather all *k*-mers from the input file and (ii) *k*-mers stored in these arrays are first sorted and then counted during the collation phase. Counting results in temporary arrays (tables), which are then merged to produce the final *k*-mer count list. GenomeTester4 uses multiple threads to speed up *k*-mer counting.

Turtle [42] uses a novel sorting and compaction (SAC)-based algorithm, which is memory efficient. Turtle works as follows: *k*-mers are added to a large array up to a certain point, each with a count of 1. This array is then sorted, identical *k*-mers are compacted, and their counts are added up in the compaction step. The compaction process frees up space in the array, which is used for a new set of *k*-mers. The SAC approach is then applied to existing and newly added *k*-mers and is performed iteratively until all *k*-mers are counted. The compaction process is similar to run-length encoding [44]. Turtle has three implementations, scTurtle, cTurtle, and aTurtle, which vary in their outputs. scTurtle has false positives and outputs only *k*-mers with frequency >1. cTurtle accepts small rates of false positives and false negatives and gives only *k*-mers with frequency >1 without any counts. cTurtle gives an approximate set of frequent *k*-mers by using a counting Bloom filter. aTurtle provides *k*-mers of all frequencies with their counts. Although multithreaded, cTurtle and scTurtle do not count perfectly, whereas single-threaded aTurtle does. Hence, in this study, aTurtle is considered.

scTurtle [42] uses a pattern block Bloom filter to remove all single-occurrence (spurious) *k*-mers. The remaining *k*-mers are then counted using the SAC approach. Pattern block Bloom filter [45] is a cache-friendly variant of the Bloom filter, which has a very small cache miss ratio.

### *k*-mer counting using a hash table

A hash table [46] can be used to count *k*-mers, in which *k*-mers are stored as keys and their counts are stored as values. Jellyfish [35] uses a lock-free hash table to allow parallel insertion of *k*-mers and frequency updates by multiple threads using a compare-and-swap (CAS) assembly instruction [47]. The CAS operation detects simultaneous access to a shared memory location in the multithreaded environment. The entire memory capacity is used to store the hash table. Once the hash table is full, it is written to disk instead of doubling its size in the memory, and intermediate *k*-mer counts are then merged [48, 49]. Jellyfish works as follows: whenever a new *k*-mer appears, the program obtains its key and searches for it in the hash table. If it exists, the frequency count increases by 1. If not, this *k*-mer is inserted (with frequency set to 1) into the hash table using the reprobe strategy. If a collision occurs, it is resolved using a quadratic probing (open addressing) technique [46].

Jellyfish 2 is a more efficient version of Jellyfish, which has an additional Bloom filter-based mode to remove all singleton *k*-mers (i.e., *k*-mers occurring only once in the dataset). KAT [50] counts *k*-mers using a modified version of the Jellyfish 2 library.

### *k*-mer counting using a Bloom filter and its variants, and counting quotient filter

A majority of any genomic dataset is made up of single-frequency *k*-mers, which are mainly attributed to sequencing errors. A Bloom filter [51] is a probabilistic data structure used for dynamic membership query lookup, which can implicitly store all *k*-mers. It is used to filter out singleton *k*-mers. The frequency of every non-singleton *k*-mer can then be counted using any of the approaches in Table 1. The Bloom filter returns some false-positive membership query results, which may lead to *k*-mer miscounts. However, with a reasonable choice of hash functions, the false-positive rate can be minimized to an acceptable degree [52]. It also requires very little memory (i.e., enough to store a bit-vector [52]), reducing the overall memory requirement.

Using a similar concept, BFCounter [36] filters out singleton *k*-mers and uses a hash table to store and count non-singleton *k*-mers. Some false-positive singleton *k*-mers are erroneously included in the hash table, leading to miscounts, but BFCounter generates correct results by reiterating over the sequence reads.

Squeakr [34] is an in-memory approach for counting *k*-mers both approximately and exactly. It uses a counting filter data structure (counting quotient filter [CQF] [53]) to store *k*-mer counts. *k*-mers are hashed using a one-way hash function, and these hashes are stored in a counting filter. The single-phase algorithm is based on the following ideas: multiple threads read input data from the disk in chunks and simultaneously insert *k*-mers into a global shared thread safe CQF for *k*-mer counting. Each thread maintains a local CQF to temporarily hold the *k*-mer counts to reduce waiting time, while acquiring a lock in the global CQF (it is hardest to acquire the lock when repetitive *k*-mers are present in the dataset). Once the local CQF is full, it dumps counted *k*-mers into the global CQF before processing a new set of *k*-mers. For this benchmark study, we have not considered Squeakr (exact), which counts the frequency of each *k*-mer exactly using an invertible hash function, because its code is not yet available.

### Enhanced suffix array-based counting

Tallymer [22] is an in-memory approach, which uses a longest common prefix (lcp)-interval tree constructed from an enhanced suffix array [54] to count *k*-mers. The lcp-interval tree implicitly stores the number of occurrences of all substrings of *s*’ (reads are concatenated into a string *s*’ with a unique termination symbol ($_i_) appended to each read). The algorithm has two steps: (i) the divide step splits sequence *s*’ into smaller distinct partitions, and the *k*-mers in each partition are then counted using the lcp-interval tree; and (ii) the merging step, in which a final count is generated by merging the counts generated from all distinct partitions using the sequence *s*’. Suffix array construction of a string is expensive in terms of computation and memory requirement. Suffix array size increases linearly with the length of string *s*’.

### Trie data structure-based *k*-mer counting

KCMBT [43] is an in-memory approach using burst trie, a variant form of a trie [55]. Burst tries efficiently manage large string sets in the memory and maintain strings in a sorted or nearly sorted order. KCMBT uses extended *k*-mers, similar to KMC2. Extended *k*-mers ((*k* + *x*)-mers for *x* > 0) are substrings of length greater than *k* and were introduced by KMC2.

The KCMBT algorithm has three phases. First, (*k* + *x*)-mers are generated from the input reads and inserted into the corresponding trees. To do this, a fixed-length container is initially maintained for each tree. When a container is full, it bursts and is replaced by a new trie node and a set of child containers. These child containers partition (*k* + *x*)-mers of the original container among themselves by taking a one-symbol prefix (matching A/C/G/T) of the (*k* + *x*)-mers (*x* is chosen empirically to be 0 ≤ *x* ≤ 3 for better performance). Second, each (*k* + *x*)-mer tree ((*k* + 1)-mer, (*k* + 2)-mer, and (*k* + 3)-mer trees) is traversed to count all unique (*k* + *x*)-mers [42], which are then broken into *k*-mers to obtain a count of constituent *k*-mers. *k*-mers are then inserted into *k*-mer trees. Finally, *k*-mer trees are traversed to produce counts of all unique *k*-mers, and these, along with their counts, are written to disk. Because of (*k* + *x*)-mers, the inserted number of *k*-mers and the time required for traversal in the last phase are much reduced, leading to faster computation. Thousands of trees with smaller heights are generated to reduce the overall insertion and traversal time required to count huge numbers of *k*-mers. Burst trie has a very rapid search time, but its size becomes large for large volumes of sequencing data.

### Disk-based *k*-mer counting

The disk-based approach to *k*-mer counting has a much lower memory requirement than in-memory approaches and was designed to make it possible to count *k*-mers in large genomic datasets, such as a human genome dataset, on commodity hardware. Memory usage can be greatly reduced using a disk because *k*-mers are processed in chunks and stored on disk.

DSK [33] is a disk-based approach to counting *k*-mers using very little memory and disk space. To achieve this, DSK calculates the number of partitions needed to bring data in parts from disk to memory, depending on (i) the total bits required to store the *k*-mers and (ii) the disk size available. DSK calculates the number of iterations needed to read the entire set of input in parts, depending on (i) the total number of bits required to represent the entire set of *k*-mers, (ii) the memory size required to hold the hash table, (iii) the number of partitions, and (iv) the load factor for which hash table gives the best performance. Each *k*-mer is distributed to one of multiple disk-stored partitions, depending on its hash value and an iteration number. *k*-mers are counted by loading a partition into the memory one at a time, using hash tables in multiple iterations. The partition strategy means that DSK efficiently addresses memory constraints, but it may result in a high I/O cost.

KAnalyze [40] uses a sorting-based approach to count *k*-mers. The algorithm has two phases. First, *k*-mers are filled into a temporary array of predefined size. Once the array is full, *k*-mers are sorted, counted, and written to disk so that space becomes available to count the next incoming chunk of *k*-mers. The process is repeated until all the *k*-mers are processed. In the second phase, count files are loaded from disk to memory and are merged in multiple steps to generate final *k*-mer counts.

### Approaches using the concept of super *k*-mer: minimizers and signatures

The disk-based compression technique minimum substring partitioning (MSP) [56] is used to further reduce memory requirements and I/O operations. In this technique, input reads are broken into multiple disjoint partitions.

The adjacency relationship between each pair of *k*-mers means that *k*-mers carry highly redundant data. With MSP, if consecutive *k*-mers share the same lexicographical minimum substring *s*, then they are stored as one substring of length greater than *k*. This substring is called a “super *k*-mer” and is stored in a disk partition corresponding to the lexicographical minimum substring *s*, where *s* is termed a “minimizer.” Larger numbers of consecutive *k*-mers sharing the same minimum substring *s* give a better compression ratio, which ultimately reduces I/O overhead and storage space.

MSPKmerCounter [32] is the first tool to implement MSP for *k*-mer counting. It works as follows: (i) reads are decomposed into super *k*-mers and distributed to disk partitions (bins) identified by canonical minimizers. The storage of super *k*-mers with the same canonical minimizer in the same partition ensures that all the occurrences of the same *k*-mer belong to the same partition, thus eliminating the need to merge the counts of each partition. These smaller partitions are easily accommodated into the memory and are processed independently. (ii) Once the partitions are ready, all super *k*-mers are broken into *k*-mers using simple bit shift operations. (iii) Finally, *k*-mers are counted using hash tables, and counts are stored on disk.

KMC2 [39] is another disk-based approach that is similar to the MSP employed in MSPKmerCounter. Here, the minimizer is refined to signatures, which significantly reduce the overall memory requirements and temporary disk space. Canonical minimizers are used as signatures with the following three prerequisites: canonical minimizers (1) do not begin with prefix *AAA*, (2) do not begin with prefix *ACA*, and (3) do not contain *AA* anywhere apart from at the beginning. The KMC2 algorithm has two major phases: distribution and sorting. The distribution phase is similar to that of MSPKmerCounter, the only difference being that super *k*-mers are distributed to different temporary files (bins) based on signatures instead of minimizers. In the sorting phase, bins are processed by fetching them into the memory. For every such bin, extended *k*-mers, i.e., (*k* + *x*)-mers, are extracted from super *k*-mers, and a radix sort is applied. *k*-mer statistics are then collected from these sorted (*k* + *x*)-mers and stored on disk.

KMC3 is an extension of the KMC2 approach, with the following improvements: efficient input file reading to achieve a better I/O subsystem, a memory-efficient way of assigning signatures to bins, and an efficient sorting approach [57], rather than using a radix sort, for larger values of *k*.

Gerbil [31] uses a hashing approach to *k*-mer counting that is similar to DSK. The algorithm has two major phases. The first is slightly advanced but similar to the KMC2 distribution phase in which the hash values (obtained using a partHash [31] function) of *k*-mers (extracted from super *k*-mers) are used to ensure that multiple occurrences of the same *k*-mer are assigned to the same thread. In the second phase, super *k*-mers stored in the temporary files are sequentially re-read from the working disk. All *k*-mers are extracted from the super *k*-mers, and then counted using a hash table. Collisions are resolved using quadratic hashing. Each thread counts the assigned *k*-mers using its hash table, and these are then written into an output file. The algorithm makes optimal use of the hardware by concurrently running multiple threads. To achieve memory efficiency, hash table size is estimated using a simple linear model. In its graphics processing unit (GPU) implementation, the second phase is performed on the GPU side with proper load balancing between GPU and CPU.

## Tools Assessed, Benchmark Datasets Used, and Evaluation Methodology

We evaluated the most recently available versions of KMC3, Gerbil (version 1.0), KCMBT (version 1.0), MSPKmerCounter (version 0.1), GenomeTester4 (version 4.0), aTurtle (version 0.3), KAnalyze (version 2.0.0), DSK (version 2.2.0), Jellyfish (version 2.2.6), and BFCounter (version 1.0). Tools from 2010 or earlier were excluded. All tools used are freely available to download (refer to Supplementary Table S2).

To make a reasonable assessment of these tools, we applied them to seven datasets of varying sizes, mostly those used by Kokot et al. [37]. Table 2 summarizes details of the datasets used. FV and DM are small datasets; HS2 is the largest. NC and AT (the same used by Gerbil [31]) were chosen because their longer read lengths would allow performance with larger values of *k* to be tested. All seven datasets are available to freely download (see Supplementary Table S2).

**Table 2:** Datasets used in our study

Sr. no.	Dataset ID	Organism	Genome size (Mb)	Input FASTQ/FASTA file size (GB) (1 GB = 10^9^ bytes)	Average read length (bases)	Total no. of bases (Gb)	Total no. of reads
1	FV	*F. vesca*	214	10.9	353	4.5	12,803,137
2	DM	*D. melanogaster*	122	10.5	76	3.7	48,432,878
3	MB	*M. balbisiana*	472	197.1	100	56.3	562,968,372
4	HS1	*H. sapiens 1*	2,991	292.1	151	123.7	819,148,264
5	HS2	*H. sapiens 2*	2,991	339.5	100	135.3	1,339,740,542
6	NC	*N. crassa*	41	23.3	7,778.3	22.9	2,942,564
7	AT	*A. thaliana*	120	72.7	4,804.6	36.1	7,515,360

Sequencing reads in each file (for each dataset) were first decompressed and then concatenated into a single FASTA/FASTQ file to facilitate the smooth running of each tool. However, not all tools support direct decompression. All datasets used in this study had multiple compressed files. Some *k*-mer counting tools directly support compressed (raw) input and can thus effectively perform parallelization in their first phase by reading from individual input files using separate threads. This means that restricting the input to a single file effectively limits them to one or two threads (e.g., one to parse and one to bin/partition). Most tools would likely perform even better on multifile inputs without being concatenated (normalized) into a single file. We tested the effect of compressed input (gzip and bzip2) on the performance of various programs by running them directly on compressed input files.

Tables 4–8 present comparisons of the different tools that were tested on the FV, DM, MB, HS1, and HS2 datasets for two values of *k* (28 and 55). Tests lasting >15 hours were interrupted.

Tools that approximate the frequency histogram of *k*-mer occurrences (and/or estimate the number of unique *k*-mers and singleton *k*-mers) by streaming data analysis are not considered in this article. These include KmerStreame [58], ntCard [59], KmerGenie [30], and Khmer [60]. To make a fair comparison, we have only tested tools that generate exact *k*-mer counts.

The wall clock time was measured using the C++ function, “gettimeofday()” averaged over three runs. A shell script by Shin [61] was used to measure the maximum memory usage. This script uses the Linux *ps* utility to determine the peak memory use of a process and its threads by monitoring resident set size values, where resident set size reports the amount of memory actually allocated to a process and is in memory (random access memory [RAM]). We reported the maximum disk usage by the program using our own shell script, which logs the disk usage at regular time intervals between consecutive checks using the Linux command *du*. This script also captures average CPU utilization as a percentage with the help of the Linux command *top*. These scripts were executed with a sampling rate of 3 for HS1 and HS2 datasets and a sampling rate of 1 for FV, DM, MB, NC, and AT datasets. Invocations of all executables were monitored by these two scripts. Run time, memory usage, disk usage, and CPU utilization were measured simultaneously.

All experiments were performed on a test machine configured as shown in Table 3. Commands used to run all programs were adapted from their documentation and/or publications (see Supplementary Material). Commands used to list *k*-mers and their counts in human-readable format and *k*-mer coverage distribution (histogram for *k*-mer abundance) are also given in the Supplementary Material.

**Table 3:** Test machine configuration

Processor	Intel(R) Xeon(R) CPU E5–2698 v3 @ 2.30GHz
Main memory	64 GB
Hard disk drive	1 TB
CPU(s)	16
Online CPU(s) list	0–15
Thread(s) per core	2
Core(s) per socket	16
No. of sockets	1

We evaluated the accuracy of each counting program by comparing their *k*-mer frequency histograms on two small datasets with two values of *k*. This histogram is a table of *f*_i_ values, where *f*_i_ denotes the number of distinct *k*-mers that appear *i* times in the set of reads [58]. Some tools, such as Jellyfish, DSK, Gerbil, and MSPKmerCounter, can directly create histograms. For the other tools, *k*-mer frequency histograms were obtained as follows. First, the dump subroutine of the tool was run to write *k*-mer occurrences into a tab-separated text file. Second, we used our program, written in C++ using OpenMP for multithreaded computing and Linux commands (*grep* and *wc*), to generate the *k*-mer frequency histogram from this text file.

For each tool, exact numbers of *f*_1_–*f*_10_ are given in the Appendix. Space limitation means that results are only reported up to *f*_10_, but all frequency counts were considered and compared. Results for Jellyfish 2.2.6, DSK 2.2.0, KAnalyze 2.0.0, KMC3, Gerbil 1.0, KCMBT 1.0, GenomeTester4, and BFCounter 1.0 were the same for both datasets and values of *k*. In contrast, the results for MSPKmerCounter 0.1, aTurtle 0.3, and Gerbil 1.0 (only for *k* = 55) were different. Error rates of these three tools are provided in the Supplementary Tables S3–S6). MSPKmerCounter had the highest error rates, as depicted in Supplementary Tables S3–S6.

Lists of *k*-mers and their counts generated by Turtle, MSPKmerCounter, and Gerbil (only for *k* = 55) did not always match with outputs from other tools for the same datasets. For more rigorous analysis, we used our shell script, written using a set of Linux utilities, i.e., *sort* (to sort in lexicographical order) and *diff* (which analyzes two files and prints the lines that are different) to validate all lexicographically sorted *k*-mers and their counts.

DSK output is used as a reference to validate the output of aTurtle because these two tools use the same alphabetical order (*A* < *C* < *T* < *G*) while obtaining canonical *k*-mers. Variations were found in the lexicographically sorted *k*-mers in the outputs of aTurtle and DSK (although the frequency counts of aTurtle matched with those of DSK for the DM dataset for *k* = 55). The aTurtle output included unmatched frequency *k*-mers and some additional *k*-mers that were not present in the DSK output, and some *k*-mers present in the DSK output were missing from aTurtle. Similar variations were observed between MSPKmerCounter and KMC3 and between Gerbil and KMC3 (but only for *k* = 55).

We thus infer that the recent versions of MSPKmerCounter, aTurtle, and Gerbil may contain bugs in their implementations.

## Result and Discussion

Entries in bold with * indicate best results; entries in bold italic (including the second lowest for CPU utilization) show average results. Since MSPKmerCounter has the highest error rates, these results, and those for compressed input, were not considered in the best and average results. In the “Disk” column, only disk-based tools were considered in the best and average results. Abbreviations are s = seconds, GB = gigabytes. Failure messages are indicated above if a program failed to complete the computation because of insufficient memory/disk space or within a stipulated time (15 hours).

For the FV and DM datasets, all programs completed the *k*-mer count within 15 hours. However, for the HS1 and HS2 datasets, KCMBT and GenomeTester4 did not complete within 15 hours and neither did Jellyfish for the HS1 dataset, even in Bloom-filter-based mode. These jobs also had to be killed because high memory usage froze the system. For the HS1 and HS2 datasets, aTurtle failed, returning the “std::bad_alloc Aborted (core dumped)” error message because of high memory usage and KAnalyze with the “java.io.IOException: No space left on device” error.

For the HS1 and HS2 datasets, BFCounter was unable to complete within 15 hours and the system froze, therefore the job was killed. For the HS1 dataset, MSPKmerCounter failed during phase 2, returning the “OutOfMemoryError” error.

### Runtime, memory, and disk usage

Table 9 provides an easily readable comparison of all 10 tools (excluding compressed input results), including the best and average programs in terms of time, memory, disk, and CPU utilization.

Of all the tested programs, only DSK and KMC3 generated accurate results for both *k* values within the stipulated time and without system freeze issues for all seven datasets (see Appendix Tables A1 and A2; accuracy checked against FV and DM datasets only).

Because it is single-threaded, aTurtle will always be lowest in terms of CPU utilization. Therefore, the second lowest entries are also mentioned in the “CPU Utilization (%)” column.

DSK consistently used a moderate amount of memory, had reasonable speeds, and, in passing all the tests, demonstrated robustness. KMC3 was often superior in terms of running time but used more memory than its top competitor, Gerbil. However, it was often close to being the best in terms of disk utilization.

Interestingly, Gerbil was consistently the most memory- and disk-efficient approach. For most of the datasets, Gerbil had the lowest disk utilization but was slower than KMC3 (the GPU implementation of Gerbil was not considered). Gerbil attempts to reduce disk and memory utilization, making it economical in terms of both of these parameters. As seen in Table 9, hash table-based counting approaches seem to be more efficient than sorting-based approaches in terms of hardware use. Newer tools, specifically KMC3 and Gerbil, which use MSP and bin size (signature) balancing, performed best in terms of memory requirements.

For most of the datasets, and for both *k* values, KAnalyze had much higher runtime and disk usage compared to the other disk-based approaches. KAnalyze needs more time for the merging step because its partitioning step is relatively straightforward.

In-memory approaches need no extra disk space because these are completely memory based. Among the in-memory algorithms, BFCounter utilized the smallest amount of memory because of the underlying memory-efficient Bloom filter. For the MB dataset, Jellyfish was the fastest in-memory algorithm and had the highest CPU utilization, but for the HS1 dataset, it was not able to complete within 15 hours. For the datasets that Jellyfish was able to complete within the stipulated time, both time and memory requirements were comparable. Gerbil and Jellyfish often had the highest CPU utilization. Perez et al. [25] reported similar behaviors of various *k*-mer counting tools in terms of runtime and memory usage.

### Performance for larger values of *k*

**Figure 1:**
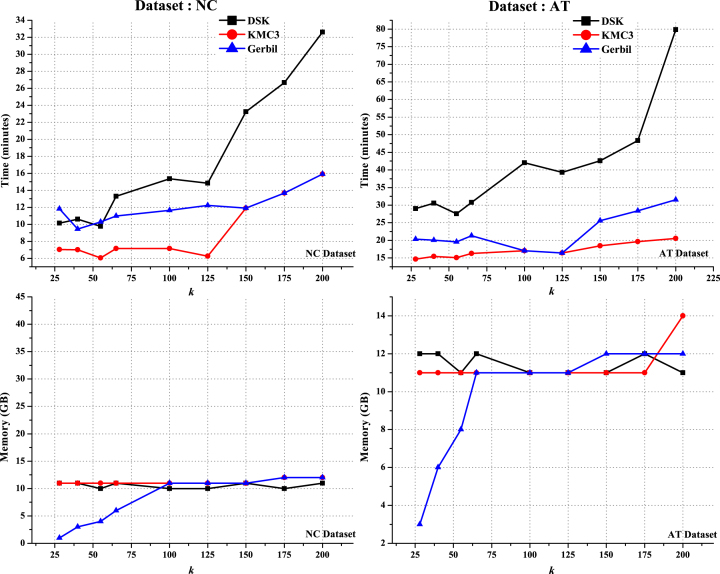
Analysis of time and memory utilization of *k*-mer counting algorithms for the NC and AT datasets for different values of *k* (28, 40, 55, 65, 100, 125, 150, 175, and 200).

GenomeTester4, KCMBT, and aTurtle do not support large values of *k* (see Supplementary Table S1). For the NC and AT datasets, BFCounter failed, returning a “segmentation fault (core dumped)” error, whereas Jellyfish and KAnalyze did not complete within 15 hours. Only KMC3, DSK, and Gerbil succeeded in generating results for the different *k* values (28, 40, 55, 65, 100, 125, 150, 175, and 200) for these datasets within the stipulated time (see Fig. 1). MSPKmerCounter was unable to generate output for the NC dataset, but for the AT dataset, succeeded for all values of *k* (28, 40, 55, 65, 100, 125, 150, 175, and 200). However, because of its high error rate, MSPKmerCounter is not included in our comparisons.

Gerbil was consistently the most memory-efficient approach, but when the value of *k* increased, it utilized almost the same amount of memory as DSK and KMC3. For the NC dataset, KMC3 was faster than Gerbil, but at higher *k* values (150, 175 and 200), they had similar runtime. For the AT dataset, KMC3 was faster than DSK and Gerbil. DSK used almost the same amount of memory as KMC3 but was slower than KMC3 and Gerbil. In taking minimal time and using moderate amounts of memory, KMC3 and Gerbil efficiently support both small and large values of *k*.

### Scalability to varying sizes of datasets

For datasets with shorter reads, such as DM, the runtime of each tool decreases with increasing values of *k* (Table 5). Only three programs, Gerbil, KMC3, and DSK, succeeded in generating results for large datasets (HS1 and HS2) within a reasonable timeframe and without freezing (Tables 7 and 8). These tools use a disk-based approach, are more efficient in terms of time and memory utilization, and are scalable for large datasets (size >200 GB) compared to tools based on an in-memory approach. Jellyfish is memory efficient and, for the HS2 dataset, was the only in-memory algorithm that completed a *k*-mer count within a reasonable time, consuming 58 GB and 48 GB memory for *k* = 28 and 55, respectively (Table 8).

### The impact of compressed input

Because of their large size, sequencing data are generally stored in a compressed format, usually gzip. One advantage of programs that support compressed input is that the I/O throughput improves with the use of hard drives because the algorithm consumes the data faster. Data throughput is increased because compressed data are read directly from the disk, thereby overcoming the cost of file decompression in memory.

Currently, only the following five tools support compressed input: KMC3, Gerbil, DSK, KAnalyze, and BFCounter. As shown in Tables 4–8, reading the input in a compressed form reduces the running time, having a noticeable, positive impact on counting *k*-mers in large datasets, i.e., HS1 and HS2. KMC3 was the fastest of the tested programs that support compressed input. For the HS1 and HS2 datasets, KMC3 and Gerbil's gzipped input time was much less than the normalized input time, whereas the opposite trend was seen with DSK. As shown in Tables 7 and 8, using gzipped input compared to normalized input, KMC3 was approximately 47% and 53% faster (*k* = 28 and *k* = 55, respectively) for the HS1 dataset and 44% and 48% faster (*k* = 28 and *k* = 55, respectively) for the HS2 dataset. Gerbil was approximately 30% and 32% faster (*k* = 28 and *k* = 55, respectively) for the HS1 dataset and approximately 26% and 27% faster (*k* = 28 and *k* = 55, respectively) for the HS2 dataset. DSK was approximately 19% slower (*k* = 28 for HS1) and 17% and 28% slower (*k* = 28 and *k* = 55, respectively) for HS2.

**Table 4:** Experimental results for the FV dataset

SN	Tools (version; compression type)	*k* = 28				*k* = 55			
		Time (s)	RAM (GB)	Disk (GB)	CPU utilization (%) (comment)	Time (s)	RAM (GB)	Disk (GB)	CPU Utilization (%) (comment)
1	Jellyfish (2.2.6)	138.33	7.9	0	1093.55 (consistent)	226	**36.19**	0	1,050.93* (consistent)
2	DSK (2.2.0)	56.33	6.35	6	866.50 (consistent)	78.33	7.04	5	633.49 (declined from ∼1174 to ∼129.7)
3	DSK (2.2.0; gzip)	197	4	6	402.71 (first 80% of time consistent with ∼300; last 20% inconsistent to ∼1,200 with sudden increase)	222	6	5	441.21 (first 75% of time consistent with ∼390; last 25% inconsistent to ∼1,200 with sudden increase)
4	KAnalyze (2.0.0)	***2,042***	10	***22.2***	509.20 (initially in the range 1,000–2,000 then declined to ∼200)	***4095***	11	***42***	337.46 (initially in the range 1,000–2,000 then declined to ∼150)
5	KAnalyze (2.0.0; gzip)	1,999	9	22.9	507.84 (first 30% of time inconsistent in the range 2,250–750; last 70% consistent with sudden drop to ∼200)	3,395	11	12.8	360.456 (first 25% of time inconsistent to ∼900; last 75% consistent to ∼200 with a sudden drop)
6	KMC3	38.66	7.66	4*	998.10 (consistent)	35*	11.2	4	987.891 (consistent)
7	KMC3 (gzip)	35	7	2.2	1,004.61 (consistent)	37	11	0	1,056.25 (consistent)
8	Gerbil (1.0)	33.66*	0.83*	4*	1,110.38* (consistent)	60.33	1.29*	3*	1,030.50 (consistent)
9	Gerbil (1.0; gzip)	49	0.82	1.5	858.46 (first 50% of time consistent with ∼600; last 50% suddenly increased to ∼1,200)	55	1	1	880.77 (first 50% of time consistent with ∼600; last 50% suddenly increased to ∼1,300)
10	KCMBT (1.0)	137.5	***30.98***	0	628.87 (inconsistent)	Not supported			
11	MSPKmerCounter (0.1)	59.33	4.45	1	811.70 (phase 1: consistent (∼200); phase 2: consistent (∼1,500))	67.33	4.61	1	770.87 (phase 1: consistent (∼200); phase 2: consistent (∼1,500)
12	aTurtle (0.3)	671	14	0	***99.14*** (consistent)	1,185	26	0	***94.12*** (consistent)
13	GenomeTester4	214	26	0	***202.33*** (consistent)	Not supported			
14	BFCounter (1.0)	1731	3	0	274.10 (first 80% of time almost 100, then gradual increase to ∼1,000)	1,790	9	0	***271.80*** (consistent to ∼100, but bars hiking to ∼1,000 in the middle and end)
15	BFCounter (1.0; gzip)	1,847	3	0	259.70 (inconsistent)	1,889	9	0	251.49 (inconsistent)

**Table 5:** Experimental results for the DM dataset

SN	Tools (version; compression type)	*k* = 28				*k* = 55			
		Time (s)	RAM (GB)	Disk (GB)	CPU utilization (%) (comment)	Time (s)	RAM (GB)	Disk (GB)	CPU utilization (%) (comment)
1	Jellyfish (2.2.6)	77	4	0	1,055.25 (consistent)	71	9	0	917.79 (consistent)
2	DSK (2.2.0)	52	2	4.2	736.09 (initially ∼600, increasing to ∼1,173)	49	2	2.7	622.36 (initially ∼500, increasing to ∼1,150)
3	DSK (2.2.0; gzip)	183	4.68	3.6	331.88 (first 90% of time consistent with ∼270, last 10% suddenly increasing to ∼1,200)	173	4.45	2.4	300.70 (first 90% of time consistent with ∼250 then suddenly increasing to ∼1,200)
4	KAnalyze (2.0.0)	794	10	***14.3***	695.64 (gradually declined)	393	11	***12.8***	829.45 (gradually declined from ∼2,000 to ∼100)
5	KAnalyze (2.0.0; gzip)	822	9	14.4	691.15 (first 40% of time consistent with ∼1,250; last 60% inconsistent with sudden drop to ∼200)	411	11	12.9	843.79 (first 60% of time inconsistent in the range 2,250–900, rest of time inconsistent with sudden drop to ∼200)
6	KMC3	18*	5	2.23	942.26 (consistent)	13*	8	0.6*	1,023.54* (consistent)
7	KMC3 (gzip)	35	5	1.64	739.01 (last 20% of time consistent with ∼1,250, rest consistent with ∼700)	31	8	0	637.29 (last 20% of time consistent with ∼1,250, rest consistent with ∼600)
8	Gerbil (1.0)	20	0.81*	2.11*	1,184.23* (consistent)	16.5	0.81*	4	1,010.89 (consistent)
9	Gerbil (1.0; gzip)	33	0.82	1.31	821.69 (first 55% of time consistent with ∼700, last 45% suddenly increased to ∼1,200)	29	0.81	0	685.74 (first 55% of time consistent with ∼600, last 45% inconsistent with sudden increase to ∼1,200)
10	KCMBT (1.0)	61	2	0	595.37 (initially ∼300 then increased towards end to ∼900)	Not supported			
11	MSPKmerCounter (0.1)	234	5	14.2	912.92 (both phases: consistent)	219	5	11.2	914.62 (phase 1: initially ∼1,000 then declined to ∼300; phase 2: consistent)
12	aTurtle (0.3)	423	7	0	***97.20*** (consistent)	330	***12***	0	***95.39***(consistent)
13	GenomeTester4	144	***23***	0	***183.92*** (consistent)	Not supported			
14	BFCounter (1.0)	***914***	1	0	307.53 (consistent)	***477***	2	0	***331.48*** (first 95% of time in the range 250–400, then increasing to ∼800)
15	BFCounter (1.0; gzip)	1,002	2	0	321.78 (last 20% of time ∼500, rest is ∼300)	559	2	0	306.30 (last 20% of time ∼500, rest ∼300)

**Table 6:** Experimental results for the MB dataset

SN	Tools (version; compression type)	*k* = 28				*k* = 55			
		Time (s)	RAM (GB)	Disk (GB)	CPU utilization (%) (comment)	Time (s)	RAM (GB)	Disk (GB)	CPU utilization (%) (comment)
1	Jellyfish (2.2.6)	1,467*	15	0	800.13* (consistent)	1,440*	***24***	0	691.65* (consistent)
3	DSK (2.2.0)	3,358	12	59	185.09 (consistent)	3039	11	45	***208.54***(consistent)
4	KAnalyze (2.0.0)	***51,422***	10	***189***	279.40 (initially ∼2,000, then declining to ∼150)	***45,367***	11	***245***	248.04 (declined from ∼2,000 to ∼100)
5	KMC3	2,019	9	36	216.93 (initially in the range 12–400; increasing towards end to ∼600)	1,804	10	14	211.12 (initially in the range 12–400, increasing towards end to ∼600)
6	KMC3 (bz2)	3,341	11	36.3	289.46 (first 90% of time consistent in the range 200–400; last 10% up to ∼1,300)	3,250	11	13	282.77 (first 90% consistent in range 200–400; last 10% up to ∼1,300)
7	Gerbil (1.0)	2,238	2*	32*	269.52 (initially within 150, increasing towards end to ∼800)	1,941	3*	11*	250.32 (initially within 150, increasing towards end to ∼800)
8	Gerbil (1.0; bz2)	3,487	2	30.7	306.37 (first 90% of time consistent with ∼270; last 10% suddenly increasing to ∼1,300)	3,137	3	11	304.02 (first 90% of time consistent with ∼270; last 10% suddenly increasing to ∼1,300)
9	KCMBT (1.0)	1,644	34	0	***135.87***(consistent)	Not supported			
10	MSPKmerCounter (0.1)	11,094	8	173	316.90 (consistent)	8,759	9	118	1,284.05 (consistent)
11	aTurtle 0.3	8,764	***61***	0	***75**.07***** (consistent)	>15 hours			
12	GenomeTester4	3,520	60	0	153.67 (consistent)	Not supported			
13	BFCounter (1.0)	18,950	10	0	300.37 (consistent)	15,264	19	0	295.40 (first 50% up to ∼254 then increasing to ∼434)

**Table 7:** Experimental results for the HS1 dataset

SN	Tools (version; compression type)	*k* = 28				*k* = 55			
		Time (s)	RAM (GB)	Disk (GB)	CPU utilization (%) (comment)	Time (s)	RAM(GB)	Disk (GB)	CPU utilization (%) (comment)
1	Jellyfish (2.2.6)	>15 hours (system hang)				>15 hours (system hang)			
2	DSK (2.2.0)	***7,722***	***12***	***133***	***210.2*** (inconsistent)	***9,389***	***14***	***48***	***255.862***(inconsistent)
3	DSK (2.2.0; gzip)	9,240	11	134	218.77 (inconsistent)	8,480	12	104	284.68 (inconsistent)
4	KAnalyze (2.0.0)	Failed: “IO error writing segment file: no space left on device”				Failed: “IO error writing segment file: No space left on device”			
5	KAnalyze (2.0.0; gzip)	Failed: “IO error writing segment file: no space left on device”				Failed: “IO error writing segment file: No space left on device”			
6	KMC3	3,725*	10	78	276.64 (gradually declined)	3,466*	11*	28	270.55 (inconsistent)
7	KMC3 (gzip)	1,964	11	79	620.84 (inconsistent)	1,626	11	29	663.31 (inconsistent)
8	Gerbil (1.0)	4,078	6*	66*	370.77* (initially within ∼200, increasing towards end to ∼1,200)	3,818	11*	21*	320.21* (inconsistent)
9	Gerbil (1.0; gzip)	2,849	6	66	569.83 (first 70% of time consistent to ∼420, then increasing to ∼1,000 for last 30%)	2,614	11	22	541.63 (first 70% of time consistent to ∼400, then increasing to ∼1,000 for last 30%)
10	KCMBT (1.0)	>23 hours				Not supported			
11	MSPKmerCounter (0.1)	>15 hours (phase 2 failed: “OutOfMemoryError”)				>15 hours (phase 2 failed: “OutOfMemoryError”)			
12	aTurtle (0.3)	Aborted (core dumped)				Aborted (core dumped)			
13	GenomeTester4	>15 hours				Not supported			
14	BFCounter 1.0	>15 hours				>15 hours			
15	BFCounter (1.0; gzip)	>15 hours				>15 hours			

**Table 8:** Experimental results for the HS2 dataset

SN	Tools (version; compression type)	*k* = 28				*k* = 55			
		Time (s)	RAM (GB)	Disk (GB)	CPU utilization (%) (comment)	Time (s)	RAM (GB)	Disk (GB)	CPU utilization (%) (comment)
1	Jellyfish (2.2.6)	3,310*	***58***	0	1,000.29* (consistent)	***11,126***	***48***	0	376.578* (declined from ∼1,000 to ∼100)
2	DSK (2.2.0)	***8,879***	13	***145***	***186.66***(consistent)	7,982	13	***109***	***211.54*** (consistent)
3	DSK (2.2.0; gzip)	10,360	10	146	242.01 (inconsistent)	10,199	12	109	240.21 (first 60% of time consistent with ∼300, last 40% suddenly increasing to ∼650)
4	KAnalyze (2.0.0)	Failed: “IO error writing segment file: no space left on device”				Failed: “IO error writing segment file: no space left on device”			
5	KAnalyze (2.0.0; gzip)	Failed: “IO error writing segment file: no space left on device”				Failed: “IO error writing segment file: no space left on device”			
6	KMC3	4,252	10	85	218.02 (increased towards end to ∼600, otherwise up to ∼12)	3,846*	11	29	214.99 (increased towards end to ∼600, otherwise up to ∼12)
7	KMC3 (gzip)	2,362	10	86	580.72 (inconsistent)	1,995	11	29	556.31 (inconsistent)
8	Gerbil (1.0)	4,553	5*	74*	371.26 (increased towards end to ∼1,000, otherwise up to ∼250)	4,260	9*	23*	317.65 (initially ∼250, increasing towards end to ∼1,000)
9	Gerbil (1.0; gzip)	3,358	5	74	553.59 (first 70% of time consistent to ∼400, then increasing to ∼1,000 for last 30%)	3,121	9	23	507.19 (first 70% of time consistent to ∼450, then increasing to ∼1,000 for last 30%)
10	KCMBT (1.0)	>15 hours				Not supported			
11	MSPKmerCounter (0.1)	3,128	6	22.2	120.17 (consistent)	3,124	9	5.7	340.49 (consistent)
12	aTurtle (0.3)	Aborted (core dumped)				Aborted (core dumped)			
13	GenomeTester4	>15 hours				Not supported			
14	BFCounter (1.0)	>15 hours				>15 hours			
15	BFCounter (1.0; gzip)	>15 hours				>15 hours			

**Table 9:** Summary of Tables 4–8

Dataset ID	*k* length	Time		RAM		Disk		CPU utilization (%)	
		Highest	Lowest	Highest	Lowest	Highest	Lowest	Highest	Lowest
FV	28	KAnalyze	Gerbil	KCMBT	Gerbil	KAnalyze	Gerbil, KMC3	Gerbil	GenomeTester4, aTurtle
	55	KAnalyze	KMC3	Jellyfish	Gerbil	KAnalyze	Gerbil	Jellyfish	BFCounter, aTurtle
DM	28	BFCounter	KMC3	GenomeTester4	Gerbil	KAnalyze	Gerbil	Gerbil	GenomeTester4, aTurtle
	55	BFCounter	KMC3	aTurtle	Gerbil	KAnalyze	KMC3	KMC3	BFCounter, aTurtle
MB	28	KAnalyze	Jellyfish	aTurtle	Gerbil	KAnalyze	Gerbil	Jellyfish	KCMBT, aTurtle
	55	KAnalyze	Jellyfish	Jellyfish	Gerbil	KAnalyze	Gerbil	Jellyfish	DSK
HS1	28	DSK	KMC3	DSK	Gerbil	DSK	Gerbil	Gerbil	DSK
	55	DSK	KMC3	DSK	Gerbil, KMC3	DSK	Gerbil	Gerbil	DSK
HS2	28	DSK	Jellyfish	Jellyfish	Gerbil	DSK	Gerbil	Jellyfish	DSK
	55	Jellyfish	KMC3	Jellyfish	Gerbil	DSK	Gerbil	Jellyfish	DSK

Regardless of input format (compressed or normalized), KMC3 was the fastest, whereas Gerbil was the most consistent in terms of using the lease memory and disk space. bzip2 has a high compression ratio but very slow decompression. Thus, processing a bzip2 file is costlier than gzipped input (Tables 4–8). Only KMC3 and Gerbil support input data that are compressed with the bzip2 data compressor; these took long to process bzip2 files than normalized input for the MB dataset (Table 6).

### Scalability by the number of threads

**Figure 2:**
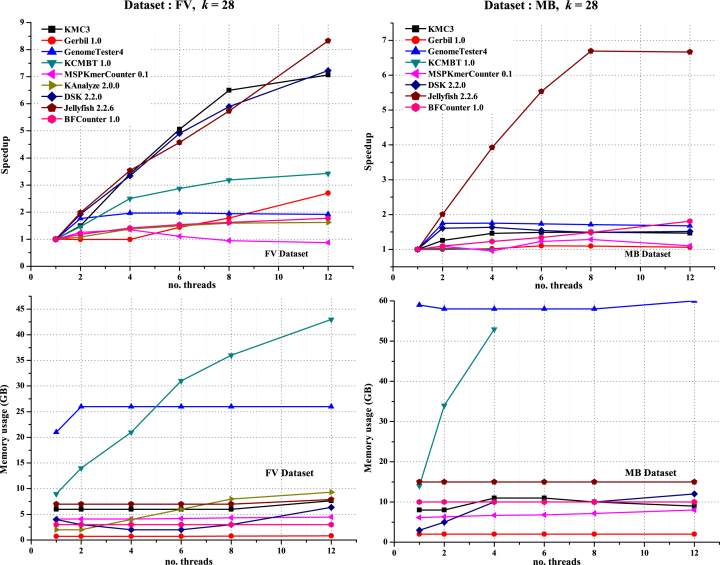
Scalability comparison of different *k*-mer counting tools based on the number of threads.

All programs (except for aTurtle, as it is single-threaded) run with different numbers of threads (1, 2, 4, 6, 8, and 12) to assess CPU-related performance. FV and MB datasets were chosen with a *k* value of 28 so that all tools could complete their execution within the stipulated time. Figure 2 shows the scalability of these tools according to the number of threads. No tool was able to achieve linear speedup.

Jellyfish, an in-memory approach using a multithreaded lock-free hash table, has the highest speedup, i.e., 8.3 and 6.7 for FV and MB datasets, respectively, for 12 threads. DSK and KMC3 have good speedup for the FV dataset, i.e., 7.2 and 7.1, respectively, for 12 threads. For the MB dataset, each program achieves only low speedup in the range of 1–2 (except for Jellyfish). This is caused by increased threading overhead; resource demand is increased by each thread because of large input size but limited underlying resources. KCMBT is the fastest for a single thread on the MB dataset (Supplementary Table S8). However, for the four threads, KCMBT was not able to complete a *k*-mer count within 15 hours for the same dataset owing to threading overheads, while memory requirements are almost constant for increasing numbers of threads with the other tools. The overall speedup achieved by each program is not very high because *k*-mer counting is fundamentally an I/O-intensive task.

## Conclusions and Future Directions

*k*-mer counting is used to solve many problems in bioinformatics. While high-throughput sequencing technologies can generate billions of reads per instrument run, there is a need to continue developing a memory- and time-efficient *k*-mer counting system for large reads.

Many disk-based and in-memory *k*-mer counting approaches are available that aim to generate results from large genomic datasets in a minimum amount of time, using personal computers with limited resources (memory, disk, etc.).

Of all the tools considered herein, KMC3, DSK, and Gerbil are the most flexible and efficient, as they have higher speeds, minimum memory requirements, and better scalability to larger datasets. They also have automatic parameter selection, are more robust, support larger values of *k* (large *k* being an important use case for longer reads), and allow compressed input. Reading the input in a compressed form improves the overall processing time. These tools are optimized to gain significant speedup by parallelizing the available cores in the machine.

As sequencing technologies evolve, research endeavors must continue to improve to develop better *k*-mer counting systems for increasingly large sequencing datasets.

## Additional files

Appendix_revised_4.doc

Supplementary_revised_4.doc

## Abbreviations

CAS: compare-and-swap; CPU: central processing unit; CQF: counting quotient filter; GPU: graphics processing unit; I/O: input/output; lcp: longest common prefix; MSP: minimum substring partitioning; NGS: next-generation sequencing; RAM: random access memory; SAC: sorting and compaction.

## Competing interests

The authors declare no conflict of interest, financial or otherwise.

## Author Contributions

Swati C. manekar and Shailesh R. Sathe conceived of the presented review. Swati C. Manekar developed the theory and performed the computations under the supervision of Shailesh R. Sathe. Both authors discussed the results and contributed to the final manuscript.

## Supplementary Material

GIGA-D-17-00245_Original_Submission.pdf

GIGA-D-17-00245_Revision_1.pdf

GIGA-D-17-00245_Revision_2.pdf

GIGA-D-17-00245_Revision_3.pdf

GIGA-D-17-00245_Revision_4.pdf

Response_to_Reviewer_Comments_Original_Submission.pdf

Response_to_Reviewer_Comments_Revision_1.pdf

Response_to_Reviewer_Comments_Revision_2.pdf

Response_to_Reviewer_Comments_Revision_3.pdf

Reviewer_1_Report_(Original_Submission) -- Qingpeng Zhang 11/4/2017 Reviewed

Reviewer_1_Report_(Revision_1) -- Qingpeng Zhang 3/7/2018 Reviewed

Reviewer_2_Report_(Original_Submission) -- Rayan Chikhi 11/13/2017 Reviewed

Reviewer_2_Report_(Revision_1) -- Rayan Chikhi 3/14/2018 Reviewed

Reviewer_2_Report_(Revision_2)-Attachment.txt

Reviewer_2_Report_(Revision_2) -- Rayan Chikhi 9/5/2018 Reviewed

Reviewer_3_Report_(Original_Submission) -- Rob Patro 11/22/2017 Reviewed

Supplemental Files
