# Supplementary material for: A benchmark study of k-mer counting methods for high-throughput sequencing
Source: Gigascience. 2018 Oct 22;7(12):giy125. doi: 10.1093/gigascience/giy125 (PMC6280066; doi:10.1093/gigascience/giy125)
Supplement: GIGA-D-17-00245_Original_Submission.pdf [file giy125_giga-d-17-00245_original_submission.pdf]

GigaScience

A benchmark study of k-mer counting methods for high-throughput sequencing

--Manuscript Draft--

**Manuscript Number:**	GIGA-D-17-00245
**Full Title:**	A benchmark study of k-mer counting methods for high-throughput sequencing
**Article Type:**	Review
**Funding Information:**	
**Abstract:**	High-throughput sequencing technologies revolutionized the ways in which many gigabytes of data are being generated. Many applications of bioinformatics require counting substrings of length k in this data, e.g. genome and transcriptome assembly, error correction, multiple sequence alignment, repeat detection and many other such applications. Many techniques for counting k-mers of sequencing data have been developed in the recent years. All k-mer counting approaches aim to process such enormous amount of data in a way that realizes a memory, time and disk trade-off. This paper presents an assessment strategy for k-mer counting programs to evaluate their relative advantages and disadvantages. Counting performance is evaluated on the basis of accuracy, runtime, memory usage, disk usage and scalability of several k-mer counting approaches by rigorous experimental analysis on wide range of various real world datasets. The scalability of each tool with respect to the larger values of k is also evaluated by considering datasets having longer reads. This review provides specific recommendations for the current state-of-the-art program for particular setup and provides suggestions for further development. All the tools evaluated in this article are freely available and can be downloaded from hosting website.
**Corresponding Author:**	Swati Chandrakant Manekar, M.Tech.<br>Visvesvaraya National Institute of Technology<br>Nagpur, Maharashtra INDIA
**Corresponding Author Secondary Information:**	
**Corresponding Author's Institution:**	Visvesvaraya National Institute of Technology
**Corresponding Author's Secondary Institution:**	
**First Author:**	Swati Chandrakant Manekar, M.Tech.
**First Author Secondary Information:**	
**Order of Authors:**	Swati Chandrakant Manekar, M.Tech.
	Shailesh Sathe, Ph.D.
**Order of Authors Secondary Information:**	
**Opposed Reviewers:**	
**Additional Information:**	
**Question**	**Response**
Are you submitting this manuscript to a special series or article collection?	No
**Experimental design and statistics**<br><br>Full details of the experimental design and statistical methods used should be given in the Methods section, as detailed in our Minimum Standards Reporting Checklist. Information essential to interpreting the data presented should be made available in the figure legends.	Yes

Powered by Editorial Manager® and ProduXion Manager® from Aries Systems Corporation

Have you included all the information requested in your manuscript?	
**Resources**<br><br>A description of all resources used, including antibodies, cell lines, animals and software tools, with enough information to allow them to be uniquely identified, should be included in the Methods section. Authors are strongly encouraged to cite Research Resource Identifiers (RRIDs) for antibodies, model organisms and tools, where possible.<br><br>Have you included the information requested as detailed in our Minimum Standards Reporting Checklist?	Yes
**Availability of data and materials**<br><br>All datasets and code on which the conclusions of the paper rely must be either included in your submission or deposited in publicly available repositories (where available and ethically appropriate), referencing such data using a unique identifier in the references and in the “Availability of Data and Materials” section of your manuscript.<br><br>Have you have met the above requirement as detailed in our Minimum Standards Reporting Checklist?	Yes

Manuscript Click here to download Manuscript Manuscript_GigaScience.doc

A benchmark study of *k*-mer counting methods for high-throughput sequencing

Swati C. Manekar[1*] and Shailesh R. Sathe[1]

[1]Department of Computer Science and Engineering, Visvesvaraya National Institute of Technology, Nagpur 440 010, India

*corresponding author, Email: swati.manekar@gmail.com

Abstract

High-throughput sequencing technologies revolutionized the ways in which many gigabytes of data are being generated. Many applications of bioinformatics require counting substrings of length *k* in this data, e.g. genome and transcriptome assembly, error correction, multiple sequence alignment, repeat detection and many other such applications. Many techniques for counting *k*-mers of sequencing data have been developed in the recent years. All *k*-mer counting approaches aim to process such enormous amount of data in a way that realizes a memory, time and disk trade-off. This paper presents an assessment strategy for *k*-mer counting programs to evaluate their relative advantages and disadvantages. Counting performance is evaluated on the basis of accuracy, runtime, memory usage, disk usage and scalability of several *k*-mer counting approaches by rigorous experimental analysis on wide range of various real world datasets. The scalability of each tool with respect to the larger values of k is also evaluated by considering datasets having longer reads. This review provides specific recommendations for the current state-of-the-art program for particular setup and provides suggestions for further development. All the tools evaluated in this article are freely available and can be downloaded from hosting website.

Keywords: *k*-mer counting, high-throughput sequencing, disk-based counting, in-memory counting, hash table, sorting.

Introduction

k-mer counting is vital in many applications of bioinformatics in the analysis of sequencing data. Tools and techniques have evolved in last few years to count the frequency of *k* length substring (*k*-mer) in the sequencing reads generated from high-throughput sequencing [1].

k-mer counting is the process of counting the occurrence frequency of fixed length substring of the input set of strings (strings are DNA/RNA sequences termed as reads). Let $\sum = \{A, C, G, T, N\}$ denotes alphabet of nucleotides of DNA sequence, where *N* denotes the undetermined character by the sequencer. A read can be considered as any finite sequence over the alphabet $\sum$. Set of all strings (reads) over the alphabet $\sum$ is denoted by $\sum^*$. Set of all such input reads is represented by *S* where $S \in \sum^*$.Every read of set *S* indicated by *r* with $r[i]$ accounts for every i^{th} character of *r* with index starting at 0 to *l*-1, where *l* is the length of *r*. Reads can be of variable lengths from l_{min} to l_{max}. *k*-mers of every such read is obtained by sliding a window of length *k* over read *r* one base at a time in right hand side till the end of read is reached by the right end corner of window. Hence for every read *r* i.e. $r\ [0…..l\text{-}1]$, a *k*-mer is any consecutive sub-string of length *k* of read *r* indicated by $r\ [i....j]$ having *i* as a start index and $j = i + k – 1$ as an end index such that $0 \le i \le j \le l\text{-}1$.

Let's consider an example where we have 2 reads of fixed length say 6 in a set *S*, *S* = {{*ACGTTA*},{*ACGTTT*}}.A 4-mer is a 4-character long substring of every such reads in input set *S* is obtained as set *s*, *s*= {{*ACGT*, *CGTT*, *GTTA*}, {*ACGT*, *CGTT*, *GTTT*}}.*s* indicates the set of *k*-mers of reads. This is called as *k*-mer extraction process. These *k*-mers are now counted by using various approaches, i.e. either by using sorting & counting approach or hash table based counting approach. The final result of the counting process is *k*-mer along with its count as

denoted by set *f* where *f* = {(*ACGT* 2), (*CGTT* 2), (*GTTA* 1), (*GTTT* 1)}.

Counting *k*-mers is applied in the de novo genome assembly viz., the overlap layout consensus approach [2, 3] and the de Bruijn graph [4-7] based assembly. It has application in transcriptome assembly and in identifying the protein binding sites [8]. Error correction of reads is performed to improve genome assembly quality. Error correction based on *k*-mer spectrum approach [9-12] and multiple sequence alignment based approach [13] also uses the frequency count of *k*-mers. The probable misalignment in the reads is either due to errors or genuine nucleotide variations can be estimated using *k*-mer frequencies. *k*-mer counting also aids in multiple sequence alignment of protein sequences [14]. The length of the read, error rate of the sequencer and the size of the genome can be estimated using *k*-mer frequency statistics [15]. de novo repeat annotation techniques like ReAS make use of high-frequency *k*-mer as a seed to find repeats [16], whereas RepeatScout also uses the same to build set of repeat families [17]. Precompiled repeat library to identify the repeats in the genome have been used in RAP [18] and FORRepeats [19]. Precompiled repeat library is also used to identify the exact word matches to annotate large genomes in [20]. Tallymer a *k*-mer counting tool uses the k-mer frequencies to annotate repetitive plant genome [21]. Duplication studies have been conducted to quantify complex repetitive features of DNA in several genomes for varying lengths of *k*-mers [22]. The count of *k*-mers has also been used to infer the genotypes of known variants [23].

The *k*-mer counting though is a simple and straightforward task, becomes very difficult once billions of next generation sequencing (NGS) data need to be processed. The simple and basic approach for counting *k*-mers can be implemented using an array with substring indexing. The approach becomes infeasible in terms of memory and time when input reads are billions in number. Considering this fact, the approaches proposed so far have mainly targeted memory

efficient solution for *k*-mer counting. In the light of this, many heuristic techniques and approaches have been implemented in the various research works. One way to achieve memory efficiency can be representing the string data into an integer. Many memory efficient data structures used by various researchers can be listed as, enhanced suffix array, burst trie, lock free hash table, membership query data structure like bloom filter, pattern block bloom filter, counting quotient filter (*CQF*) and so on. Further, as the secondary memory is always a magnitude cheaper than the primary memory, many researchers have focused on using the disk with heuristics for scaling to larger data sets. The approach is termed as disked based / external memory/ out-of-core approach as opposed to in-memory / internal memory approache.

In this article, a review of all such algorithms for *k*-mer counting of high throughput sequencing data and their comparative evaluation is presented. The main purpose of this article is to provide a general set of benchmark and assessment matrix along with the experimentation assessment of *k*-mer counting tools giving a thorough insight to the beginners and consultant. Perez et al. [24] studied various *k*-mer counting tools for *k*-mer lengths of 31 and 55. The presented benchmark study covers all the areas of evaluation where as the existing literature review is incomplete or inadequate. The parameters considered for this assessment are such that they give a comprehensive idea for assessments of counting programs. The parameters used in the evaluation are accuracy, runtime, memory usage, temporary disk usage, scalability to process larger k value and capability to deal with enormous real world data. This article is organized as follows. For every listed tool, first, we present the algorithmic study of their approach under various categories; the next section introduces the tools considered for benchmark study, followed by datasets used and multiple dimensions (parameters) taken into account for an

assessment. The results of comparisons are discussed in the next section. Finally, we conclude with the guidelines and future research directions.

Overview of *k*-mer counting approaches

Depending upon the approach and data structure used the various *k*-mer counting tools can be categorised as shown in Table 1.

Table 1 Approaches for *k*-mer counting

Approach for counting	Disk-based	In-memory
Hash-table	Gerbil [25], MSPKC [26], DSK [27],	Squeakr [28], Jellyfish [15], BFCounter [29]
Sorting	KMC3 [30], GTester4 [31], KMC2 [32], KAnalyze [33], KMC1 [34]	Turtle [35]
Burst tries	-	KCMBT [36]
Enhanced suffix array	-	Tallymer [21]

k-mer counting using sorting approach

Sorting approach works by extracting *k*-mers from reads and then *k*-mers are sorted in lexicographical order. *k*-mers can then be easily counted using the sorted list as similar *k*-mers lie adjacent to each other in this sorted list.

GTester4 [31] efficiently uses the sort and count approach for *k*-mer counting implemented using an array type data structure. The algorithm is implemented in two phases wherein, in reading phase, temporary arrays are used to gather all *k*-mers from the input file. While in collation phase, k-mers in these arrays are sorted and then counted. The temporary table outputs are then merged to produce the final *k*-mer count list. GTester4 cannot process large dataset of NGS reads which contains many repeated *k*-mers and a large number of reads. Running time and memory requirement by algorithm depends on input size and can count *k*-mers up to maximum length of 32.

k-mer counting using a hash table

For *k*-mer counting, a typical data structure which can hold *k*-mer against its count is needed such as hash table [37] and is found suitable. By using the proper hash function, the index of an array can be calculated to insert a *k*-mer. whenever a new *k*-mer appears; first the hash table is searched for that *k*-mer. If the *k*-mer already exists, the frequency for that *k*-mer is incremented by one, else inserted into the best possible location with a count equal to one. Thus, the counting of *k*-mers is accomplished. However, if multiple keys are assigned to the same index, then *k*-mer must be accommodated using an appropriate collision handling technique.

Under the same framework, Jellyfish [15] uses a hash table for *k*-mer counting and implements the quadratic probing (open addressing) for collision handling. It introduces lock-free hash table to allow parallel insertion of *k*-mers and frequency updating by multiple threads using CAS (compare-and-swap) assembly instruction [38]. CAS detects simultaneous access to a shared memory location in a multithreaded environment. For storing hash table, the entire memory is used. When a hash table becomes full, it gets written to the disk, instead of doubling size of the hash table in the memory, and intermediate *k*-mer counts are then merged [39][40]. At run time, it requires the user to specify the estimated number of distinct *k*-mers i.e. the size of the hash table.

The algorithm is implemented using two arrays (hash tables) namely, ‘key array’ a map type structure for holding keys and ‘value array’ to hold the frequency of respective key. *k*-mer is encoded in an integer called key. It works as follows, insert keys into key array using appropriate hash function, (a) the key where it hashes, if that slot is free then inserts the key and update the count of respective slot in the value array; (b) else if the slot is occupied by the same key then increment count in respective slot of value array; (c) else if keys are different then the collision

has occurred. The collision is resolved and then the key is inserted into the appropriate new slot along with its count updated in value array.

More efficient version of jellyfish is available as Jellyfish 2, with the additional BF-based mode, which implements bloom filter to remove all singleton *k*-mers. A modified version of the Jellyfish 2 library is used by KAT [41] for *k*-mer counting to its maximum advantage.

k-mer counting with the application of bloom filter, its variants and counting quotient filter

A major part of the entire genomic data set is consumed by single frequency *k*-mers which is mainly due to sequencing errors. A bloom filter [42] is a probabilistic data structure used for dynamic membership query look up which can implicitly store all *k*-mers and can be used to filter out such single frequency *k*-mers. The frequency of every non-singleton *k*-mer can then be counted using any of the approaches discussed above. The bloom filter has some amount of false positive membership query results, hence miscounting may occur. However, with a reasonably chosen number of hash functions, the false positive rate can be minimised to an acceptable degree. Though bloom filter has some false positive membership query result, it requires very low memory, i.e. only to store a bit vector, which highly reduces the overall memory requirement.

BFCounter [29] uses the same concept of bloom filter to filter out singleton *k*-mers and uses the hash table to store and count the remaining *k*-mers. Some percent of singleton *k*-mers may also get added in a hash table because of false positives giving erroneous counting result. But BFCounter generates correct results by reiterating over input data.

Pattern block bloom filter is an enhanced version of the bloom filter in which the cache miss ratio is very small [43]. For bigger genomic data set, the size of bloom filter increases such that it cannot be accommodated entirely into the cache memory, resulting in a large number of cache

misses. Which can be resolved by grouping the *k*-mers into blocks having near proximity bit patterns i.e. having a set of hash values in a certain range and then query *k*-mers block by block.

scTurtle [35] which is an internal memory approach uses a pattern block bloom filter to remove all such spurious k-mers. Then all non-spurious *k*-mers with frequency > 1 are added to a big array with a frequency equal to one. An array is then sorted once it is full then identical k-mers are clubbed together and their counts are added up in the compaction step. Compaction process will free up space in an array which is used to count the rest non-spurious k-mers. The process is repeated till all k-mers are counted. This is called as a novel sorting and compaction-based algorithm which is memory efficient solution for counting k-mers instead of using a hash table. The compaction process is similar to run-length encoding [44].

Squeakr [28] is another in-memory approach that uses hashing with a linear probing technique for collision handling. It uses approximate membership query (AMQ) data structure, a counting quotient filter (*CQF*) [56] that is better than bloom filter in terms of its ability to modify and remove *k*-mers. *CQF* is also better than count-min sketch in terms of lossless compression of *k*-mers. *CQF* maintains *k*-mers and its count in an array *Q* of size 2*q*. Index of an array *Q* is quotient *q* which is obtained by diving the hash value of each *k*-mer *x* and remainder *r* will be stored as the key of that *k*-mer. Squeakr exploits ‘shared thread safe *CQF*’ with lock for multithreaded environment. To scale with the highly distorted data having high multiple occurrences of *k*-mers, local *CQF* is maintained by every thread. Every such thread will then dump their results into global shared *CQF* once it is completely filled before processing next set of *k*-mers.

Enhanced suffix array based counting

Tallymer [21] is an in memory approach based on enhanced suffix array [45]. It uses lcp-interval tree constructed from lcp (longest common prefix) table for *k*-mer counting. An interval [*i*...*j*] of the lcp-interval tree represents a string occurring ($j - i + 1$) times in *s*' (reads are concatenated into a string *s*' with unique termination symbol ($\$_i$) appended to each read). It is implemented in two steps: (i) divide step split sequence *s*' into smaller distinct partitions. Then *k*-mers in each such partitions are counted using the lcp-interval tree (ii) in merging step the partially counted *k*-mer files are combined using the sequence *s*'. But for strings constructing suffix array is quite expensive in terms of computation and again suffix array memory requirement increases linearly with the size of the genome and its coverage.

Trie data structure based k-mer counting

KCMBT [36] is a *k*-mer counter based on a novel trie based approach called burst trie [46] which is a modified suffix trie. It also implements extended *k*-mer which was first introduced and implemented by KMC2. Extended *k*-mer i.e. ($k + x$)-mers for $x > 0$, is a compact way to store and count *k*-mers. The algorithm consists of three phases viz., the generation phase, the insertion phase, and the traversal phase. In the first phase, the canonical *k*-mers which is the lexicographically smaller sub-string (*k*-mer) than its reverse complement are extracted from reads. For every possible case, extended *k*-mers (($k + x$)-mers) are obtained. In the second phase, these (k + x)-mers and *k*-mers are inserted into the burst tries. When buckets of burst tries exceed the predefined capacity, all extended *k*-mers are distributed across the child buckets. Finally, in the third phase, the trees are traversed to count their frequencies. Because of the ($k + x$)-mer the computation becomes fast, as the number of insertions is minimized. To reduce overall insertion

and traversal time for a huge number of *k*-mers, thousands of trees with smaller heights are generated.

Burst trie is the data structure where the search is very fast, but the size of the trie becomes large when input size is high. To run on the sufficiently big data set, it requires a computer with bigger memory than the desktop system.

Disked-based *k*-mer counting

Furthermore, vast memory requirements have been lessened by disk-based approaches. The disk-based approach is a memory frugal approach for counting *k*-mers specially designed to make possible the *k*-mer counting of large genomes on commodity hardware. By making use of the disk, memory usage can be greatly minimized as an entire data structure need not be stored in the memory at a time. The *k*-mers are instead processed in chunks. Disk space is used to hold these chunks. In the following section, the disk-based approaches like DSK, KAnalayze, MSPKC, KMC2, KMC3 and Gerbil are presented along with their comparative analysis. DSK, KMC and Gerbil implemented two-disk architecture.

DSK [27] can count *k*-mers using very low memory and disk space by implementing a single large hash table. It achieves this by calculating the number of partitions needed to bring data in parts from disk to memory depending upon (i) the total bits required to store the *k*-mers and (ii) disk size available. It calculates the number of iterations required to process entire set of input reads in parts, depending on, (i) the total number of bits required to represent the entire set of *k*-mers, (ii) memory size to hold the hash table, (iii) number of partitions and (iv) load factor for which hash table gives the best performance. The extracted *k*-mers are assigned to the partitions depending on their hash values and an iteration number. Partitions are stored on the disk. *k*-mers

are in turn counted by loading a partition in memory at a time using a hash table in multiple iterations. DSK incorporates an efficient partitioning strategy implemented to deal with memory constraints though it may turn out to be high I/O demanding.

KAnalyze [33] is a *k*-mer toolkit which implements disk based approach that uses sorting for counting. In the initial phase, extracted *k*-mers are added to a temporary array of predefined size. Once the array is full, *k*-mers are counted and this partially counted result is dumped into a disk so that space becomes available to process next incoming chunk of *k*-mers. The same process is repeated till all the *k*-mers are processed. In the second phase, the partially counted result files (disk files/bins/partitions) are loaded from disk into memory and are merged to generate a final count file using suitable merging technique.

Approaches using concept of super k-mer: Minimizer and Signatures

To further minimise memory requirement and I/O operations, the disk based compression technique MSP (minimum substring partitioning - super *k*-mer) proposed in [47] came into picture wherein input set of reads are partitioned into the various partitions/bins.

Super *k*-mer is the compressed way to store consecutive *k*-mers sharing the same minimum substring into one single substring of length greater than *k* into the disk partition. *k*-mers of reads carries highly redundant data, as there exist the adjacency relationship between every pair of *k*-mers. The *k*-1 nucleotides of first *k*-mers are exactly same as the *k*-1 nucleotides of second adjacent *k*-mer. Consider a read R, $R = \{R_1\ R_2\ R_3\ ....\ R_m\}$ of length m. In super *k*-mer extraction process, instead of breaking every read into substring of length k, if adjacent x *k*-mers from $R[i, i+k-1]\ ...\ R[i+j-1, i+j+k-2]$ share the same lexicographical minimum substring s of length l, where $l << k$, then these *k*-mers can be stored as a one substring $R_i\ R_{i+1}...\ R_{i+j+k-2}$. This substring is called as super *k*-mer and is stored into disk partition corresponding to the lexicographical

minimum substring *s*, where *s* is termed as minimizer. If the number of consecutive *k*-mers *x* sharing the same minimum substring *s* is larger in number then high compression ratio is achieved, reducing I/O overhead with minimum storage space requirement.

MSPKC [26] was the fisrt which implemented same idea wherein canonical minimizer, a canonical minimum substring *s* of length *l* (*l*-mer) is considered. The algorithm works as follows. (i) Reads are first decomposed into super *k*-mers and distributed to the respective disk partitions (bin) identified by canonical minimizer. Super *k*-mers sharing same canonical minimizer in a partition assures that same *k*-mers will always occur in the same partition eliminating the tedious process of merging partial results at the end as faced in KAnalyze [33]. These smaller partitions are easily accommodated into main memory and processed independently. (ii) All super *k*-mers are then broken into *k*-mers using simple bit shift operation once partitions (bins) are ready. (iii) Finally *k*-mer are counted using hash tables.

KMC2 [32] is another disk based approach that uses the same approach of super *k*-mer as employed in MSPKC [26]. However, minimizer approach suffers from the imbalance of bin size, wherein a bin with minimizer corresponding to the minimizer *AA* ... *A* can be so large that such partition may occupy entire memory. Hence KMC2 addressed the problem using the concept of signature and subsequently was used by Gerbil [25]. Signature is a canonical minimizer with 3 pre-requisites: (1) it should not begin with the prefix *AAA* (2) should not begin with the prefix *ACA* and (3) it can start with the prefix *AA* but not anywhere else. Overall memory and disk space are reduced due to signatures as compared to simple canonical minimizer. The KMC2 algorithm consists of two major phases, distribution phase and sorting phase. The first phase is similar to the first phase of MSPKC, with the only difference that the super *k*-mers are distributed to the different temporary files (bins) based on the signatures instead of a minimizer. Super *k*-

mers are dumped into the disk when the bin is full to release the space for the next incoming super *k*-mer; this process is repeated till all reads are processed. In the second phase, bins are processed by bringing them into memory. For every such bin extended *k*-mers i.e. (*k*, *x*)-mers and *k*-mers are then extracted from the super *k*-mer. After sorting (radix sort) the identical entries ((*k*, *x*)-mers and *k*-mers) are clubbed and their counts are added in compaction process. *k*-mer statistics (*k*-mers with their frequencies) are then collected from these sorted (*k*, *x*)-mers and *k*-mer. Finally, results are stored into disk.

The KMC3 is an extension of KMC2 approach having few improvement such as (i) efficient input file reading to achieve better I/O subsystem (ii) memory efficient approach to assigning signatures to block (iii) their own efficient sorting approach [48] rather than radix sort to make it efficiently work for larger values of *k*.

The overall performance of *k*-mer counting is moreover affected by the length of minimizer (larger or smaller) and the total ordering on minimizer ($C < G < A < T$ / $C < A < T < G$ / $A < C < G < T$ [32] with 3 prerequisites to avoid larger size of bins / random order / ascending and descending sorting of minimizer to get rare minimizer, etc). Empirical analysis on various datasets can best suggest the optimum length of a minimizer. For most of the datasets, it is observed that a minimum number of super *k*-mers (minimum disk space, more compression) is more advantageous than even bin size (small maximum number of *k*-mers per minimizer) [25] which is achieved by following the strategy (signature) adopted in KMC2 as discussed above.

Gerbil [25] uses hashing approach for counting similar to DSK. For *k*-mers grouping, it uses the concept of super *k*-mer with minimizer similar to MSPKC and ranking of minimizer similar to KMC2. It consists of two major stages. The first stage is similar to that of KMC2 with little advancement. To make sure that the multiple occurrences of the same *k*-mer should get assigned

to the same thread in a subset, hash values of *k*-mers (extracted from super *k*-mers) are used which is obtained with the application of part hash function. Every thread then counts the respectively allocated *k*-mers using the respective hash tables. Finally, the hash table containing counts of *k*-mer are written into an output file. In GPU implementation, the only second phase is performed on GPU side with proper load balancing as GPU is much faster than CPU. Every possible step of the algorithm is parallelized and hash table size is estimated using a simple linear model. It makes use of a hash table to improve efficiency when higher *k* values need to be processed. Suzuki S. et.al. [57] have also leveraged the power of GPUs for counting *k*-mers.

Benchmark data-sets and evaluation methodology

The total seven data sets are chosen for evaluating the performance of various tools most of which are referred from KMC3 publication to have reasonable assessment. Of these seven datasets, FV and DM are two small sized data sets and HS2 is the largest one. To evaluate the scalability of each tool with respect to bigger size of input data sets, HS1 and HS2 are used in this study. To test the performance of various tools for higher *k* values, datasets with long reads are chosen similar to that used by Gerbil. NC and AT are longer read datasets having average read length of 7778.3 and 4804.6 respectively. The datasets chosen are from actual experiments which include variety of genomic coverage and read lengths. Details of the data sets used are summarized in Table 2. For each dataset genome size information is collected from the ncbi website i.e. https://www.ncbi.nlm.nih.gov/genome/browse/ and the total number of bases information is collected from the website i.e. https://www.ncbi.nlm.nih.gov/sra/?term=SRX040485. The total number of reads in given fasta or fastq file is calculated by using the commands ‘grep -c "^>" data_set.fasta’ and ‘ awk

'{s++}END{print s/4}' data_set.fastq.' respectively. The datasets available in compressed form gzip and bz2 are first decompressed and then concatenated into a single fastq/fasta file for tool execution. For all data set downloaded in .sar file format is first converted to fastq/fasta files format using command './fastq-dump SRR_no.sra --fasta' and './fastq-dump SRR_no.sra'. Finally, all fasta/fastq files are concatenated into one file using Linux *cat* command. SRA toolkit is downloaded from https://trace.ncbi.nlm.nih.gov/Traces/sra/sra.cgi?view=software. All the information for downloading all used datasets is listed in supplementary document.

Table 2 Data set specifications

Sr. No.	Data set ID	Organism	Genome size (M Base)	Input FASTQ file size (GigaBytes)	Average read length(Bases)	Total No. Of bases (G Bases)	Total no. of reads
1	FV	F. vesca	214	10.9	353	4.5	12803137
2	DM	D. melanogaster	122	10.5	76	3.7	48432878
3	MB	M. balbisiana	472	197.1	100	56.3	562968372
4	HS1	H. sapiens 1	2,991	292.1	151	123.7	819148264
5	HS2	H. sapiens 2	2,991	339.5	100	135.3	1339740542
6	NC	N. crassa	41	23.3	7778.3	22.9	2942564
7	AT	A. thaliana	120	72.7	4804.6	36.1	7515360

We have evaluated KMC3, Gerbil (version 1.0), KCMBT (version 1.0), MSPKC (version 0.1), GTester4 (version 4.0), scTurtle (version 0.3), kAanalyze (version 2.0.0), DSK (version 2.2.00), Jellyfish (version 2.2.6), and BFCounter (version 1.0) according to their chronological order of their release and only tools released after year 2010 with their recent version are considered for benchmark study. Turtle has three implementations, scTurtle, cTurtle and aTurtle varying in their output formats. cTurtle gives only *k*-mer with frequency >1 without its count and aTurtle gives *k*-mer with all frequencies with counts. For benchmarking purpose, we have selected only scTurtle as it count *k*-mer with frequency >1. We have not considered aTurtle for experimentation purpose as it also counts one frequency *k*-mers and it is found to be very slow as compared to

scTurtle. The source code is free for all selected tools and can be found from the links as given in Table A in supplementary document.

The tools which give an approximation of *k*-mer counts histogram by streaming analysis of the data are not considered in this paper. Like KmerStreame [49], ntCard [50] and KmerGenie [51] implement a streaming algorithm, estimating *k*-mer abundance histogram only. Similarly, khmer [52] built on library implemented in [53] uses hyperloglog which is the probabilistic approach for approximate cardinality estimation. Whereas khmer [54] uses a count-min sketch which is a probabilistic data structure. It generates approximate multi set representation of *k*-mer counts. khmer [55] is a toolkit for *k*-mer-based dataset analysis. All these use a significantly lower amount of memory and are reasonably fast.

Time is recorded using function 'gettimeofday' in c++ which considers the wall clock time. For every tool, disk, memory and % CPU utilizations are recorded simultaneously. A shell script by Jaeho Shin is used to calculate memory usage which monitors the *rss* peak for a multithreaded program with a sampling rate of 1 (downloaded from https://github.com/jhclark/memusg). It simply watches the values given by '*ps* -o *rss*=' where *rss* is the real memory (resident set) size of the process. The script calculates the memory usage of the whole process tree using Linux *ps* command considering all forked child processes. Shell scripts implemented using Linux command *du* and *top* are used to record temporary disk utilization and average % CPU utilization respectively. These scripts are executed with a sampling rate of 3 for all bigger datasets (HS1 and HS2) and 1 for smaller datasets (FV, DM, MB, NC and AT).

All the experiments were performed on the machine with the configuration as shown in Table 3. Commands used to execute the tools are adopted from the documentation of respective tools

and also from the publications of KMC3 and KMC2. The commands used for *k*-mer counting, for dumping results and histogram generation are given in the supplementary document.

Table 3 Machine Configuration

**Processor :**	Intel(R) Xeon(R) CPU E5-2698 v3 @ 2.30GHz
**Main memory :**	64GB
**Hard Disk Drive :**	1 TB
**CPU(s) :**	16
**On-line CPU(s) list :**	0-15
**Thread(s) per core :**	2
**Core(s) per socket :**	16
**No of socket :**	1

All tools except BFCounter, Jellyfish, scTurtle and MSPKC have automated parameter selection for many of the program parameters. An improvement could be achieved by including automated parameter selection in these tools.

Jellyfish requires pre-specifying the size of the hash table and document says to set its size 10% more than the total estimated unique number of *k*-mers. Hence the sizes were set 10 % more than the estimated values taken from KMC3 for all the datasets. For instance, the number of unique counted *k*-mers by KMC3 for DM is 132108719 hence we set the hash table size equal to 250 M (250000000). As scTurtle requires a setting expected number of unique *k*-mers to select the size of an array, the same values are set here. In case of BFCounter, the estimated number of distinct *k*-mer (an upper-bound) needs to be specified and we specified the same.

For Turtle, 11 numbers of threads were adopted, as the tool demands number of threads to be prime [35]. For KAnalyze, the parameter i.e. threads amongst *k*-mer generation step (- *l*) and spilt step (-*d*) are set according to the document specification.

For Gerbil, KMC3 and DSK the max amount of RAM in GB, was set to 12 for all the tests whereas, they do not even use the entire maximum allocated memory. KCMBT requires the number of threads to be the order of power of 2, and for our machine configuration, threads = 8

give the best performance in terms of memory and time requirement. The average length of reads must be set accurately for MSPKC as improperly set lengths do not generate the output. The number of blocks and the minimum substring length are set according to the documentation while executing MSPKC. The rest of the parameters for all the tools are set by default as per guidelines provided by the tool.

The counting time has only been considered for every tool excluding the dumping time. Each tool was executed three times for all the input datasets. The average of all three run times is reported as actual time taken by each tool. We have executed all the tools by making sure that it should consider only canonical forms of *k*-mers and it should also exclude *k*-mers occurring less than two as singleton *k*-mers are considered to appear in a read library due to sequencing errors. Thus, the uniformity can be maintained and the statistics for all the tools can be collected on the equality basis. For all tools by default canonical forms of *k*-mers are considered, except for Jellyfish (-*C*) and KAnalyze 2(-*rcanonical*), the option to consider only canonical form is explicitly specified by passing the parameter.

For KAnalyze, the time recorded by the tool includes the time required for counting *k*-mers with frequency one also, as the program does not provide an option to exclude the singleton *k*-mers. KAnalyze generates the output in human readable text format instead of the encoded format, which is not the case with other tools. And hence, other tools require one more additional step to dump the *k*-mers along with their count out of the encoded format. In case of MSPKC , the first phase generates nodes files containg super *k*-mers are written to the disk in text format rather in encoded format but output file containing *k*-mers with their counts is generated in encoded format. The current version of all the listed tools skips counting of *k*-mers containing '*N*' character, which frequently occurs in NGS data.

Considering time and memory limitations two small size datasets i.e. FV and DM are used for validating the results of each tool. The accuracy of all the tools presented in our study has been tested using the frequency count histogram, obtained in two steps. First, the output in the form of *k*-mers with their count is dumped into a single file through the tools. Next, our own parallel testing program using OpenMP with Linux command *grep* and *wc* (word count) are used to produce the *k*-mer frequency count histogram from this output file. Some tools are readily available with histogram program like Jellyfish, DSK, Gerbil and MSPKC. In this article the *k*-mer abundance histograms are reported up to a frequency equal to 10, owing to space limitation though all the frequency counts were studied and compared. The results are shown in Table 4 and Table 5 for FV dataset and Table 6 and Table 7 for DM dataset. The frequency histogram for output generated by Jellyfish 2.2.6, DSK 2.2.0, kAanalyze 2.0.0, KMC3, Gerbil 1.0, KCMBT 1.0, GTester4 and BFCounter 1.0 were the same for $k = 28$ and $k = 55$ for both the datasets, except in case of Gerbil for $k = 55$ the obtained statistics did not exactly match with the other methods. scTurtle is itself having some false positive results hence its frequency counts histogram did not agree with the other methods. Histograms obtained using MSPKC histogram tool were compared with the histograms obtained for the concatenated MSPKC outputs using our testing program, they were the same. It is to be noted that the frequency counts histogram of MSPKC is not consistent with the other tools for both datasets and both values of *k* values. The average and maximum error% of MSPKC 0.1, scTurtle 0.3 and Gerbil (only for $k = 55$) for datasets FV and DM for both values of *k* ($k = 28$ and 55) are included in supplementary document (Table B, Table C, Table D, and Table E). The results of MSPKC are found to be very different compared to the results of the other tools for most of the data sets. Hence for this

benchmark study we only compare the tools generating accurate results or results in near proximity range.

Table 4 Frequency count statistics for FV dataset for *k* = 28

***k*-mer freq.**	**Number of 28-mers with the respective frequency**									
	**Jellyfish 2.2.6**	**DSK 2.2.0**	**kAanalyze 2.0.0**	**KMC3**	**Gerbil 1.0**	**KCMBT 1.0**	**MSPKC 0.1**	**scTurtle 0.3**	**GTester 4.0**	**BFCounter 1.0**
1	0	363420998	0	0	0	363420998	**69066437 ***	**0**	0	0
2	49080695	49080695	49080695	49080695	49080695	49080695	**18115152 ***	**50099589 ***	49080695	49080695
3	18049815	18049815	18049815	18049815	18049815	18049815	**5639542 ***	**18314415 ***	18049815	18049815
4	11150813	11150813	11150813	11150813	11150813	11150813	**2208574 ***	**11249389 ***	11150813	11150813
5	9843691	9843691	9843691	9843691	9843691	9843691	**1091696 ***	**9886549 ***	9843691	9843691
6	10378494	10378494	10378494	10378494	10378494	10378494	**629357 ***	**10398638 ***	10378494	10378494
7	11430920	11430920	11430920	11430920	11430920	11430920	**390902 ***	**11441755 ***	11430920	11430920
8	12495242	12495242	12495242	12495242	12495242	12495242	**259176 ***	**12501891 ***	12495242	12495242
9	13289486	13289486	13289486	13289486	13289486	13289486	**185636 ***	**13293619 ***	13289486	13289486
10	13661314	13661314	13661314	13661314	13661314	13661314	**144101 ***	**13664321 ***	13661314	13661314

Bold and * marked entries indicate varying results. Abbreviation: freq. = frequency.

Table 5 Frequency count statistics for FV dataset for *k* = 55

***k*-mer freq.**	**Number of 55-mers with the respective frequency**									
	**Jellyfish 2.2.6**	**DSK 2.2.0**	**kAanal-yze 2.0.0**	**KMC3**	**Gerbil 1.0**	**KCMBT 1.0**	**MSPKC 0.1**	**scTurtle 0.3**	**GTester 4.0**	**BFCounter 1.0**
1	0	652176330	0	0	**79102 ***	-	**39121998 ***	**0**	-	0
2	76579833	76579833	76579833	76579833	**35492642 ***	-	**7250617 ***	**77031752 ***	-	76579833
3	28281157	28281157	28281157	28281157	**13566991 ***	-	**2084150 ***	**28373957 ***	-	28281157
4	18211721	18211721	18211721	18211721	**12986541 ***	-	**832867 ***	**18237616 ***	-	18211721
5	16401875	16401875	16401875	16401875	**8473838 ***	-	**414097 ***	**16410156 ***	-	16401875
6	16723214	16723214	16723214	16723214	**8615812 ***	-	**227106 ***	**16725855 ***	-	16723214
7	17266403	17266403	17266403	17266403	**8908818 ***	-	**131829 ***	**17267514 ***	-	17266403
8	17420694	17420694	17420694	17420694	**12170688 ***	-	**82141 ***	**17421394 ***	-	17420694
9	16983119	16983119	16983119	16983119	**8661491 ***	-	**52736 ***	**16983391 ***	-	16983119
10	16005955	16005955	16005955	16005955	**8144646 ***	-	**37158 ***	**16006249 ***	-	16005955

Bold and * marked entries indicate varying results. If a method fails to process for higher *k* value, corresponding entries are denoted by '-', Abbreviation: freq. = frequency.

Table 6 Frequency count statistics for DM dataset for *k* = 28

***k*-mer freq.**	**Number of 28-mers with the respective frequency**									
	**Jellyfish 2.2.6**	**DSK 2.2.0**	**kAanalyze 2.0.0**	**KMC3**	**Gerbil 1.0**	**KCMBT 1.0**	**MSPKC 0.1**	**scTurtle 0.3**	**GTester 4.0**	**BFCoun-ter 1.0**
1	0	155281400	0	0	0	155281400	**146698167 ***	**0**	0	0
2	8864191	8864191	8864191	8864191	8864191	8864191	**8707861 ***	**8930719 ***	8864191	8864191
3	4905173	4905173	4905173	4905173	4905173	4905173	**4980239 ***	**4910935 ***	4905173	4905173
4	4626001	4626001	4626001	4626001	4626001	4626001	**4790519 ***	**4627538 ***	4626001	4626001
5	5117297	5117297	5117297	5117297	5117297	5117297	**5329251 ***	**5117986 ***	5117297	5117297
6	5763542	5763542	5763542	5763542	5763542	5763542	**5981513 ***	**5763701 ***	5763542	5763542
7	6302198	6302198	6302198	6302198	6302198	6302198	**6510090 ***	**6302440 ***	6302198	6302198
8	6658595	6658595	6658595	6658595	6658595	6658595	**6838360 ***	**6658653 ***	6658595	6658595
9	6817573	6817573	6817573	6817573	6817573	6817573	**6963587 ***	**6817669 ***	6817573	6817573
10	6799383	6799383	6799383	6799383	6799383	6799383	**6918078 ***	**6799433 ***	6799383	6799383

Bold and * marked entries indicate varying results. Abbreviation: freq. = frequency.

Table 7 Frequency count statistics for DM dataset for *k* = 55.

***k*-mer freq.**	**Number of 55-mers with the respective frequency**									
	**Jellyfish 2.2.6**	**DSK 2.2.0**	**kAanalyze 2.0.0**	**KMC3**	**Gerbil 1.0**	**KCMBT 1.0**	**MSPKC 0.1**	**scTurtle 0.3**	**GTester 4.0**	**BFCounter 1.0**
1	0	132512502	0	0	**45255 ***	-	**127152484 ***	**0**	-	0
2	15209916	15209916	15209916	15209916	**2025688 ***	-	**15386744 ***	**15213552 ***	-	15209916
3	14350714	14350714	14350714	14350714	**1926881 ***	-	**14551570 ***	**14350896 ***	-	14350714
4	14070907	14070907	14070907	14070907	**4197271***	-	**14200127 ***	**14070963 ***	-	14070907
5	12981796	12981796	12981796	12981796	**1679797 ***	-	**13035273 ***	**12981815 ***	-	12981796
6	11361229	11361229	11361229	11361229	**1458782 ***	-	**11342261 ***	**11361233 ***	-	11361229
7	9542344	9542344	9542344	9542344	**1232507 ***	-	**9474395 ***	**9542347 ***	-	9542344
8	7807048	7807048	7807048	7807048	**2659122 ***	-	**7709343 ***	**7807059 ***	-	7807048
9	6272227	6272227	6272227	6272227	**808026 ***	-	**6169544 ***	**6272238 ***	-	6272227
10	4979037	4979037	4979037	4979037	**634728 ***	-	**4880327 ***	**4979038 ***	-	4979037

Bold and * marked entries indicate varying results. If a method fails to process for higher *k* value, corresponding entries are denoted by '-'.
Abbreviation: freq. = frequency.

Result and discussion

The *k*-mer counting results in terms of time, memory, disk and % CPU utilization are summarized in Table 8 for FV dataset, for DM in Table 9, for MB in Table 10, for HS1 in Table 11 and for HS2 in Table 12. For benchmarking results we decided to wait for 15 hours for every tool taking longer time for execution and hence some data are missing in respective tables. All tools were executed for initial five datasets for two values of *k* i.e. 28 and 55. For initial five data sets, the best (* marked) and worst (italic) result of different methods are shown in bold for all considered dimensions in Table 8, Table 9, Table 10, Table 11 and Table 12.

For data set FV and DM, all tools were able to execute completely within 15 hours. For MSPKC time is calculated by adding the first phase and second phase time and memory utilization by considering the maximum out of two phases. %CPU utilization is calculated as the average of two phases in case of MSPKC.

For data set MB, HS1 and HS2, KCMBT could not finished its execution within 15 hours and the job had to be killed due to the large size of RAM usage, leaving the system in a frozen state. Jellyfish for HS1 for both *k* values could not be completed owing to the same reasons. KAnalyze failed on HS1 and HS2 with 'java.io.IOException: No space left on device' error for both values

of *k*. scTurtle for *k* = 28 on HS1 and HS2 could not finish the task in stipulated time and the job was killed due to the large size of RAM usage, again leaving the system in the frozen state. For *k* = 55 scTurtle for data set HS1 and HS2 failed with 'std::bad_alloc Aborted (core dumped)' error message. Similarly, for data set HS1 and HS2, GTester4 and BFCounter could not complete within the time limit of 15 hours and the process was killed due to a system hang. MSPKC failed to process HS1 data set in phase 2 with 'OutOfMemoryError'.

Table 8 Experimental results for FV data set

SN	Tools (Version)	*k* = 28				*k* = 55			
		**Time (sec)**	**RAM (GB)**	**Disk (GB)**	**%CPU Utilization (Comment)**	**Time (sec)**	**RAM (GB)**	**Disk (GB)**	**%CPU Utilization (Comment)**
1	Jellyfish 2.2.6	138.33	7.9	0	1093.55 (Consistent)	226	***36.19***	0	**1050.93*** (Consistent)
2	DSK 2.2.0	56.33	6.35	6	866.50 (Consistent)	78.33	7.04	5	633.487 (Decline from 1174 to 129.7)
3	KAnalyze 2.0.0	***2487***	9.3	***22.2***	436.665 (Initially in the range of 1000 to 2000 then declined to 200)	***4095***	11	***42***	***337.456*** (Initially in the range of 1000 to 2000, then declined to 150)
4	KMC3	38.66	7.66	**4***	998.084 (Consistent)	**35***	11.2	4	987.891 (Consistent)
5	Gerbil 1.0	33.66	**848MB***	**4***	**1110.38*** (Consistent)	60.33	**1.2919***	**3***	1030.5 (Consistent)
6	KCMBT 1.0	137.5	***30.98***	0	628.868 (Inconsistent)	Not Supported			
7	MSPKC 0.1	59.33	4.45	**1**	811.693 (Phase 1 : consistent (200) Phase 2 : consistent (1500))	67.33	4.61	**1**	770.87 (Phase 1 : consistent (200) Phase 2 : consistent (1500))
8	scTurtle 0.3	175.5	15.67	0	677.859 (Declined from 1000 to 100)	376	24.6	0	496.48 (Declined from 900 to 100)
9	GTester 4.0	214	26	0	***202.331*** (Consistent)	Not Supported			
10	BFCounter 1.0	**31***	3.71	0	272.669 (consistent in rage of 100 to 300)	Not Supported			

Bold and * marked entries indicates best results and bold italic entries show average results. After validation of results, MSPKC results are found to be highly varying compared to other tools. Hence its results are not considered here. For column 'Disk' the best (bold *) and average (bold italic) are highlighted considering disked based tools only. Abbreviations: sec = Seconds, GB = Gigabytes, MB = Megabytes.

Table 9 Experimental results for DM dataset

SN	Tools (Version)	*k* = 28				*k* = 55			
		**Time (sec)**	**RAM (GB)**	**Disk (GB)**	**%CPU Utilization (Comment)**	**Time (sec)**	**RAM (GB)**	**Disk (GB)**	**%CPU Utilization (Comment)**
1	Jellyfish 2.2.6	77	4	0	1055.25 (Consistent)	71	9	0	917.7885 (Consistent)
2	DSK 2.2.0	52	2	4.2	736.0856 (Initially 600 ,increased toward end to 1173)	49	2	2.7	622.364 (initially 600 then increased to 1150)
3	KAnalyze 2.0.0	794	10	***14.3***	695.635 (Gradually declined)	***393***	11	***12.8***	829.45 (Gradually declined rom 2000 to 100)
4	KMC3	**18***	5	2.23	942.263 (Consistent)	**13***	8	**614.4 MB***	**1023.54*** (Consistent)
5	Gerbil 1.0	20	**827 MB***	**2.11***	**1184.225*** (Consistent)	16.5	**826 MB***	4096 MB	1010.8805 (Consistent)
6	KCMBT 1.0	61	2	0	595.3625 (Initially 300 ,increased toward end to 900)	Not Supported			
7	MSPKC 0.1	234	5	14.2	912.9165 (phase1,phase2 : Consistent)	219	5	11.2	914.6175 (phase1 : initially 1000 and then declined to 300 phase2 : Consistent)
8	scTurtle 0.3	85	15	0	716.2095 (Consistent in range of 900 to1000)	71	***15***	0	***536.285*** (Consistent)
9	GTester 4.0	144	***23***	0	***183.917*** (Consistent)	Not Supported			
10	BFCounter 1.0	***914***	1	0	307.528 (Consistent)	Not Supported			

Bold and * marked entries indicates best results and bold italic entries show average results. After validation of results, MSPKC results are found to be highly varying compared to other tools. Hence its results are not considered here. For column 'Disk' the best (bold *) and average (bold italic) are highlighted considering disked based tools only. Abbreviations: sec = Seconds, GB = Gigabytes, MB = Megabytes.

scTurtle and BFCounter could not execute on dataset NC with error message 'segmentation fault (core dumped)' whereas GTester , Jellyfish and KAanalyze could not finished their execution within 15 hours. KCMBT and BFCounter could not generate results in case of AT dataset with 'segmentation fault (core dumped)' whereas scTurtle, GTester, Jellyfish and KAnalyze could not finished their execution within 15 hours. Hence we have conducted

experiments to compare DSK, KMC3, Gerbil and KCMBT (NC dataset only). These tools are highly optimized to support large values of *k* except for KCMBT. Figure 1 shows the running time, memory and disk utilization of these tools for *k* values 28, 40, 55, 65, 100, 125, 150, 175 and 200. MSPKC could not generate results for NC data set but succeeded to generate for AT data set for all values of *k*. Here we haven't considered it for comparison (Figure 1) as their results are highly different compared to the others.

Table 10 Experimental results for MB dataset

SN	Tools (Version)	***k* = 28**				***k* = 55**			
		**Time (sec)**	**RAM (GB)**	**Disk (GB)**	**%CPU Utilization (Comment)**	**Time (sec)**	**RAM (GB)**	**Disk (GB)**	**%CPU Utilization (Comment)**
1	Jellyfish 2.2.6	**1467***	15	0	**800.129*** (Consistent)	**1440***	***24***	0	**691.645*** (Consistent)
2	DSK 2.2.0	3358	12	59	185.084 (Consistent)	3039	11	45	***208.538*** (Consistent)
3	KAnalyze 2.0.0	***51422***	10	***189***	279.401 (Initially 2000 and then declined to 150)	***45367***	11	***245***	248.044 (Declined from 2000 to 100)
4	KMC3	2019	9	36	216.928 (Initially in the range of 12 to 400 , increased toward end to 600)	1804	10	14	211.1245 (Initially in the range of 12 to 400, increased toward end to 600)
5	Gerbil 1.0	2238	**2***	**32***	269.5225 (Initially within 150, increased towards end to 800)	1941	**3***	**11***	250.3215 (Initially within 150, increased towards end to 800)
6	KCMBT 1.0	>15 Hour (system freeze)				Not Supported			
7	MSPKC 0.1	11998	8	173	1258.065 (Consistent)	8759	9	118	1284.05 (Consistent)
8	scTurtle 0.3	1720	43	0	745.217 (Consistent)	>15 Hour (system freeze)			
9	GTester 4.0	3641	***49***	0	***154.278*** (Consistent)	Not Supported			
10	BFCounter 1.0	18950	10	0	300.367 (Consistent)	Not Supported			

Few of programs failed to process a data set within 15 hours, or due to insufficient RAM/Disk space, corresponding entries are denoted by respective mentioned failure message. Bold and * marked entries indicates best results and bold italic entries show average results. After validation of results, MSPKC results are found to be highly varying compared to other tools. Hence its results are not considered here. For column 'Disk' the best (bold *) and average (bold italic) are highlighted considering disked based tools only. Abbreviations: sec = Seconds, GB = Gigabytes, MB = Megabytes.

Table 11 Experimental results for HS 1 dataset

SN	Tools (Version)	*k* = 28				*k* = 55			
		**Time (sec)**	**RAM (GB)**	**Disk (GB)**	**%CPU Utilization (Comment)**	**Time (sec)**	**RAM (GB)**	**Disk (GB)**	**%CPU Utilization (Comment)**
1	Jellyfish 2.2.6	>23 Hours (system hang)				>23 Hours (system hang)			
2	DSK 2.2.0	***7722***	***12***	***133***	***210.2*** **(**Inconsistent)	***9389***	***14***	***48***	***255.862*** **(**Inconsistent)
3	KAnalyze 2.0.0	Failed , Error: IO error writing segment file: No space left on device				Failed , Error: IO error writing segment file: No space left on device			
4	KMC3	**3725***	10	78	276.64 (Gradually declined)	**3466***	**11***	28	270.554 (Inconsistent)
5	Gerbil 1.0	4078	**6***	**66***	**370.769*** (Initially within 200, increased towards end to 1200)	3818	**11***	**21***	**320.21*** (Inconsistent)
6	KCMBT 1.0	>15 Hour (system freeze)				Not Supported			
7	MSPKC 0.1	>15 Hours(Phase 2 failed , OutOfMemoryError)				>15 Hours(Phase 2 failed , OutOfMemoryError)			
8	scTurtle 0.3	>15 Hour (system freeze)				Aborted (core dumped)			
9	GTester 4.0	>15 Hours				Not Supported			
10	BFCounter 1.0	>15 Hours				Not Supported			

Few of programs failed to process a data set within 15 hours, or due to insufficient RAM/Disk space, corresponding entries are denoted by respective mentioned failure message. Bold and * marked entries indicates best results and bold italic entries show average results. After validation of results, MSPKC results are found to be highly varying compared to other tools. Hence its results are not considered here. For column 'Disk' the best (bold *) and average (bold italic) are highlighted considering disked based tools only. Abbreviations: sec = Seconds, GB = Gigabytes, MB = Megabytes.

Only two programs—KMC3 and Gerbil—succeeded in generating results for all values of *k* from 28 to 200 on both the data sets NC and AT in stipulated period. KCMBT could produce results for *k* value of 28 and 40 only. From Figure 1, we can see that KCMBT a burst trie based in-memory approach is the fastest for NC data set, but it utilizes the highest RAM as compared to other tools. Again Gerbil consistently the most memory and disk frugal but as *k* value increases both Gerbil and KMC3 have the same performance in most of the cases which can be clearly seen from Figure 1. KMC3 is faster as compared to Gerbil but when *k* reaches to a higher value, for example from *k* = 150 and 200 both required the same amount of time for data set NC. In the case of data set AT, KMC3 is faster as compared to DSK and Gerbil. For DSK results are

recorded for k length up to 125 as it does not handle $k > 127$. It has comparable memory requirement to KMC3 and Gerbil but it is slower and required higher disk space.

Table 12 Experimental results for HS 2 dataset

SN	Tools (Version)	***k* = 28**				***k* = 55**			
		**Time (sec)**	**RAM (GB)**	**Disk (GB)**	**%CPU Utilization (Comment)**	**Time (sec)**	**RAM (GB)**	**Disk (GB)**	**%CPU Utilization (Comment)**
1	Jellyfish 2.2.6	**3310***	***58***	0	**1000.29*** (Consistent)	***11126***	***48***	0	**376.578*** (Declined from 1000 to 100)
2	DSK 2.2.0	***8879***	13	***145***	***186.663*** (Consistent)	7982	13	***109***	***211.541*** (consistent)
3	KAnalyze 2.0.0	Failed, Error: IO error writing segment file: No space left on device				Failed, Error: IO error writing segment file: No space left on device			
4	KMC3	4252	10	85	218.024 (%CPU utilization increased toward end to 600. Otherwise it is within 12)	**3846***	11	29	214.992 (%CPU utilization increased toward end to 600. Otherwise it is within 12)
5	Gerbil 1.0	4553	**5***	**74***	371.257 (%CPU utilization increased toward end to 1000 otherwise it is within 250)	4260	**9***	**23***	317.646 (Initially 250, increased towards end to 1000)
6	KCMBT 1.0	>15 Hours (system frozen)				Not Supported			
7	MSPKC 0.1	3128	6	22.2	120.166 (Consistent)	3124	9	5.7	340.49 (Consistent)
8	scTurtle 0.3	>15 Hour (system freeze)				Aborted (core dumped)			
9	GTester 4.0	>15 Hours				Not Supported			
10	BFCounter 1.0	>15 Hours				Not Supported			

Few of programs failed to process a data set within 15 hours, or due to insufficient RAM/Disk space, corresponding entries are denoted by respective mentioned failure message. Bold and * marked entries indicates best results and bold italic entries show average results. After validation of results, MSPKC results are found to be highly varying compared to other tools. Hence its results are not considered here. For column 'Disk' the best (bold *) and average (bold italic) are highlighted considering disked based tools only. Abbreviations: sec = Seconds, GB = Gigabytes, MB = Megabytes.

In the following section, we will discuss the results on seven data sets separately for every parameter considered. Among those tools, under comparison for underlying hardware, only DSK

and KMC3 could generate accurate results (Table 4, Table 5, Table 6, and Table 7) for both values of k within the stipulated time limit without system hang for all datasets.

KMC3 often comes on the top for running time (Table 13, Figure 1), but it is not that memory frugal as compared to its top competitor, i.e. Gerbil but often not far from the best in case of disk utilization. DSK is consistently using a moderate amount of memory with reasonable speed for all the range of dataset. It is also robust concerning passing all the test.

Table 13 Summary Table

**Data-set ID**	***k*-length**	**Time**		**RAM**		**Disk**		**%CPU**	
		**Highest**	**Lowest**	**Highest**	**Lowest**	**Highest**	**Lowest**	**Highest**	**Lowest**
FV	28	kAanalyze	BFCounter	KCMBT	Gerbil	kAanalyze	Gerbil , KMC3	Gerbil	GTester 4
	55	kAanalyze	KMC3	Jellyfish	Gerbil	kAanalyze	Gerbil	Jellyfish	kAanalyze
DM	28	BFCounter	KMC3	GTester	Gerbil	kAanalyze	Gerbil	Gerbil	GTester 4
	55	KAnalyze	KMC3	scTurtle	Gerbil	kAanalyze	KMC3	KMC3	scTurtle
MB	28	kAanalyze	Jellyfish	GTester	Gerbil	kAanalyze	Gerbil	Jellyfish	GTester 4
	55	kAanalyze	Jellyfish	Jellyfish	Gerbil	kAanalyze	Gerbil	Jellyfish	DSK
HS1	28	DSK	KMC3	DSK	Gerbil	DSK	Gerbil	Gerbil	DSK
	55	DSK	KMC3	DSK	Gerbil, KMC3	DSK	Gerbil	Gerbil	DSK
HS2	28	DSK	Jellyfish	Jellyfish	Gerbil	DSK	Gerbil	Jellyfish	DSK
	55	Jellyfish	KMC3	Jellyfish	Gerbil	DSK	Gerbil	Jellyfish	DSK

Interestingly Gerbil is consistently the most memory and disk frugal which is little superior to KMC3 (Table 13). Even for the majority of datasets Gerbil's disk utilization is the lowest among all the tools along with the comparable time to KMC3 (here we did not have a benchmark for GPU implementation of Gerbil) (Table 13). Gerbil has tried to reduce the utilization of disk and memory, revealing an astonishing result - memory and disk economical which can process massive size of input data like human irrespective of underlying hardware configuration. Their results are obtained due to the approach of efficient data partitioning similar to KMC3 approach.

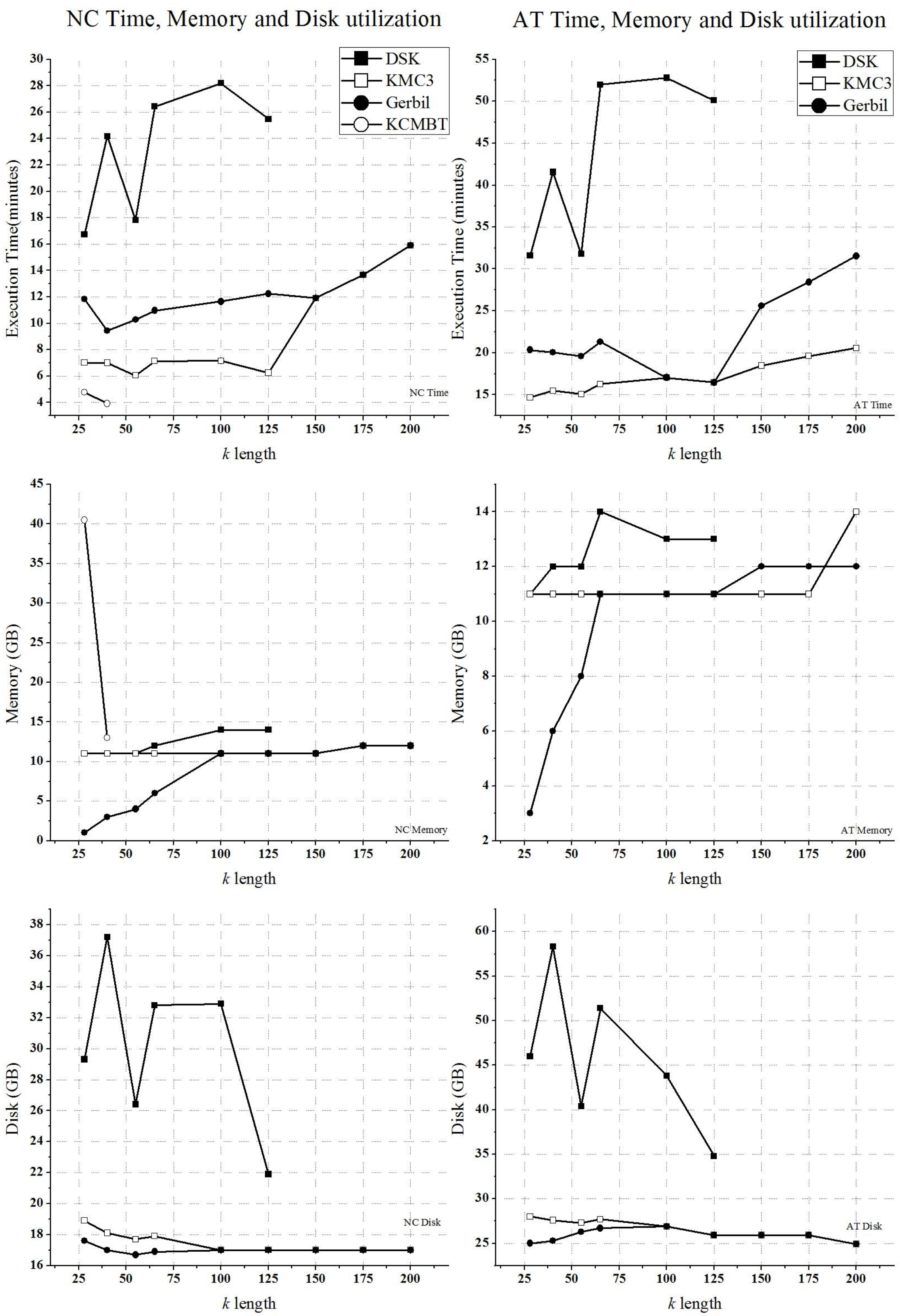

Figure 1. Analysis of time (second), memory (GB) and disk (GB) utilization of counting algorithms on AT and GT datasets for longer *k* length with, *k* = 28, 40, 55, 65, 100, 125, 150, 175 and 200

Additionally it has used effective hashing approach to count *k*-mer with a heuristic to handle weak *k*-mers and its dynamic way to predict hash table size that may add vital costs to the memory and disk usages. Low memory usage by Gerbil leaves the buffer space in memory for disk operation which effectively reduces the overall cost of expensive I/O operation. Due to lower I/O activity it has highest % CPU utilization. Hash table based counting is more hardware frugal as compared to sorting approach. But KMC3 is faster compared to Gerbil due to the efficient approach of sorting used along with other effective schemes of parallelization and input file reading.

MSP gives astonishing compression ratio which effectively reduces I/O cost and ultimately reduces overall time and space requirement. As I/O operation is much more expensive compared to counting operation. The recent tools which implement this strategy along with balance in the size of bins are KMC3 and Gerbil, which outperforms the rest tools. KAnalyze which is a disk based *k*-mer counter has much higher run-time and disk usage for the majority of datasets for both values of *k* compared with other disk based approaches (Table 13). Its performance is significantly worse compared with GTester4 which is a in-memory approach. It can be noticed that, KAanalyze 2.0.0 needs more time in merging step as its partitioning step is straightforward.

For in-memory approaches like Jellyfish, scTurtle, KCMBT, GTester4 and BFCounter, only Jellyfish and scTurtle can handle higher *k* values, but scTurtle has some false positive result. In-memory approaches need no extra disk space as these are completely memory-based. For in-memory approaches the entire dataset need to be in memory. Out of all in-memory approach based tool, BFCounter utilizes the lowest RAM. This is because of the Bloom filter data structure which is highly memory efficient. For data set MB, Jellyfish is the fastest with highest CPU utilization, but for HS1 it could not finish within 15 hours of time. Jellyfish executes with

comparable time and memory requirement whenever it could finish its execution within the time limit of 15 hours.

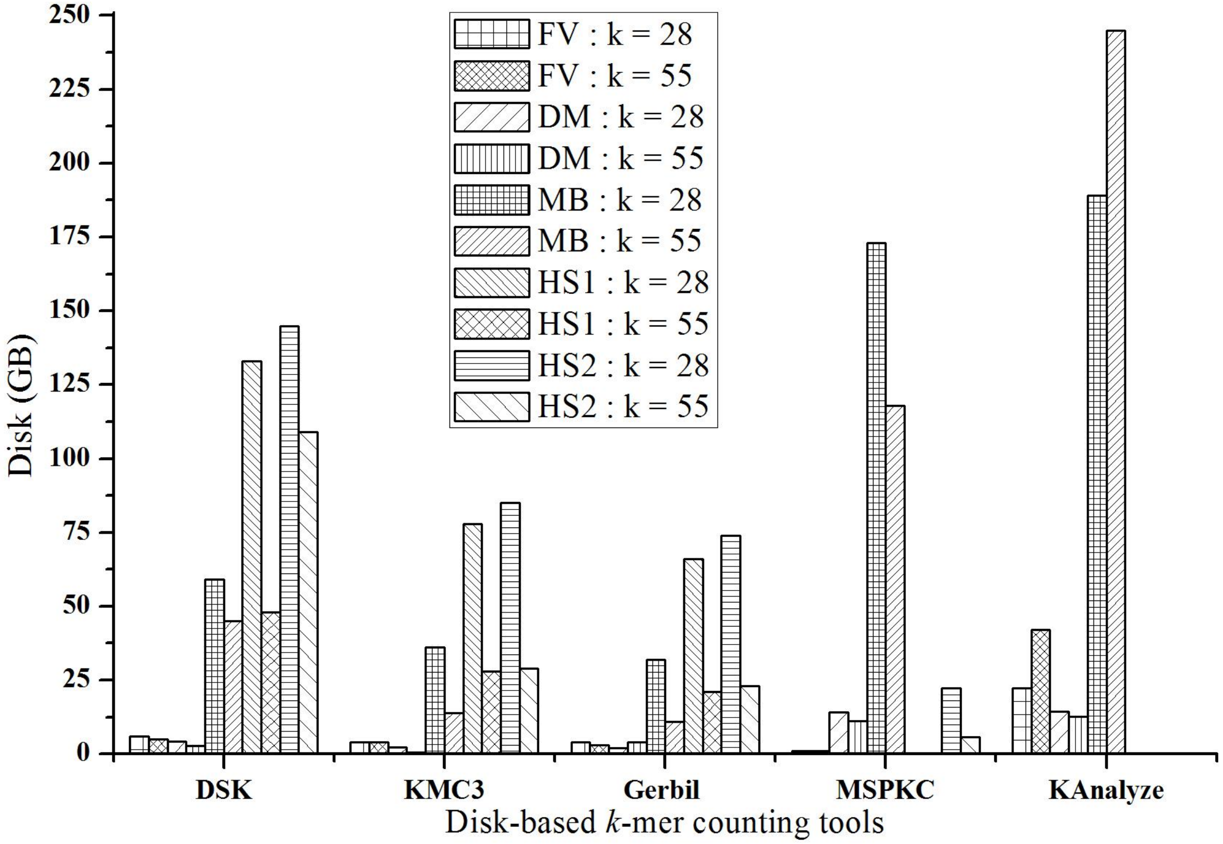

Figure 2. Analysis of disk (GB) utilization of the disk based algorithms for k-mer counting for increasing value of *k* , *k* = 8 *and k* = 55

In the case of disk based approaches it can be noticed from Figure 2 that the amount of disk utilized is reduced when k size increases from *k* = 28 to 55. This is because the number of *k*-mers/super *k*-mers dreases as the size of *k* increases. For small read length dataset i.e. DM (Table 9), the execution is faster for higher *k* in the case of most of the tools. For low machine configuration and for bigger size data sets like human data set Gerbil, KMC3 and DSK generate output with the reasonable time required. For HS1 and HS2 data-set, Gerbil, KMC3 and DSK could finish with reasonable time and without system hang. From Figure 1 we can conclude that KCMBT is more suitable for powerful computers having large RAM and disk, while disk based

approaches like DSK, Gerbil, KMC3 are a better choice for commodity computers having small RAM and disk. Gerbil and Jellyfish often have the highest % CPU utilization. For bigger datasets with size > 200 GB only the disk based approaches perform well for our machine configuration. The tools using disk based approach are more efficient in terms of memory utilization and scalability for bigger size data sets and larger value of k as compared to tools based on in-memory approach. Currently, DSK, Gerbil and KMC3 are the only programs that can handle large k values. KMC3 and Gerbil provide better scalability to larger k values owing to data set with long reads (Figure 1).

Conclusions and future directions

Considering the extensive variety of applications of k-mer counting for solving many problems in bioinformatics, latest tools till date have concentrated on the processing enormous quantity of NGS data with least required hardware and in least time because of the subsequent computational advantages. We trust this article shall provide the reader a reasonable outline of all latest tools for k-mer counting. This survey can be concluded with remarks as follows.

One objective of all programs is to reduce the time required along with little memory and disk usage. The goal might be difficult to design a program that enhances the present state of the art considering each of the three measures namely time, memory and disk simultaneously so that further analysis can be done in a faster way.

Still, there will always remain a scope of improvement in terms of time, memory and disk considering the enormous amount of high-throughput sequencing data. There is a requirement for enhancing run-time and memory impression of the k-mer counting approaches in light of the factor of 10 throughputs progresses for each year that impelled some NGS systems into the scope of billions of reads per experiment. As sequencing technologies keep on evolving, k-mer

counting algorithms should improve performance with changing field. Research endeavours must keep on improving for better establishment about how the size of data influences the *k*-mer counting process.

Reference

1. Reuter JA, Spacek D V., Snyder MP. High-Throughput Sequencing Technologies. Molecular Cell. 2015.
2. Miller JR, Delcher AL, Koren S, Venter E, Walenz BP, Brownley A, et al. Aggressive assembly of pyrosequencing reads with mates. Bioinformatics. 2008;24(24):2818–24.
3. Jaffe DB, Butler J, Gnerre S, Mauceli E, Lindblad-toh K, Mesirov JP, et al. Whole-Genome Sequence Assembly for Mammalian Genomes : Whole-Genome Sequence Assembly for Mammalian Genomes : Arachne 2. 2003;91–6.
4. Miller JR, Koren S, Sutton G. Assembly algorithm for next-generation sequencing data. Genomics. 2010;95(6):315–27.
5. Pevzner PA, Tang H, Waterman MS. An Eulerian path approach to DNA fragment assembly. Proc Natl Acad Sci [Internet]. 2001;98(17):9748–53. Available from: http://www.pnas.org/content/98/17/9748.abstract
6. Zerbino DR, Birney E. Velvet: Algorithms for de novo short read assembly using de Bruijn graphs. Genome Res. 2008;18(5):821–9.
7. Simpson JT, Wong K, Jackman SD, Simpson JT, Wong K, Jackman SD, et al. ABySS : A parallel assembler for short read sequence data ABySS : A parallel assembler for short read sequence data. 2009;1117–23.
8. Newburger DE, Bulyk ML. UniPROBE: An online database of protein binding microarray data on protein-DNA interactions. Nucleic Acids Res. 2009;37(SUPPL. 1):77–82.
9. Kelley DR, Schatz MC, Salzberg SL. Quake: quality-aware detection and correction of sequencing errors. Genome Biol [Internet]. 2010;11(11):R116. Available from: http://genomebiology.com/2010/11/11/R116%5Cnhttp://genomebiology.biomedcentral.com/articles/10.1186/gb-2010-11-11-r116
10. Shi H, Schmidt B, Liu W and M-WW. A parallel algorithm for error correction in high-throughput short-read data on CUDA-enabled graphics hardware. J Comput Biol. 2010;17(4):603–15.
11. Liu Y, Schröder J, Schmidt B. Musket: A multistage k-mer spectrum-based error corrector for Illumina sequence data. Bioinformatics. 2013;29(3):308–15.
12. Medvedev P, Scott E, Kakaradov B, Pevzner P. Error correction of high-throughput sequencing datasets with non-uniform coverage. 2011;27:137–41.
13. Salmela L, Schröder J. Correcting errors in short reads by multiple alignments. 2011;27(11):1455–61.
14. Edgar RC. MUSCLE: Multiple sequence alignment with high accuracy and high throughput. Nucleic Acids Res. 2004;32(5):1792–7.
15. Marçais G, Kingsford C. A fast , lock-free approach for efficient parallel counting of occurrences of k -mers. 2011;27(6):764–70.
16. Li R, Ye J, Li S, Wang J, Han Y, Ye C, et al. ReAS: Recovery of ancestral sequences for transposable elements from the unassembled reads of a whole genome shotgun. PLoS Comput Biol. 2005;1(4):0313–21.
17. Price AL, Jones NC, Pevzner PA. De novo identification of repeat families in large genomes. Bioinformatics. 2005;21(SUPPL. 1):351–8.
18. Campagna D, Romualdi C, Vitulo N, Del Favero M, Lexa M, Cannata N, et al. RAP: A new computer program for de novo identification of repeated sequences in whole genomes. Bioinformatics. 2005;21(5):582–8.
19. Lefebvre A, Lecroq T, Dauchel H, Alexandre J. FORRepeats: Detects repeats on entire chromosomes and between genomes. Bioinformatics. 2003;19(3):319–26.
20. Healy J, Thomas EE, Schwartz JT, Wigler M. Annotating large genomes with exact word matches. Genome Res. 2003;13(10):2306–15.
21. Kurtz S, Narechania A, Stein JC, Ware D. A new method to compute K-mer frequencies and its application to annotate large repetitive plant genomes. BMC Genomics. 2008;9:517.
22. Sindi SS, Hunt BR, Yorke JA. Duplication count distributions in DNA sequences. Phys Rev E - Stat

Nonlinear, Soft Matter Phys. 2008;78(6).

23. Pajuste F-D, Kaplinski L, Möls M, Puurand T, Lepamets M, Remm M. FastGT: from raw sequence reads to 30 million genotypes in less than an hour. bioRxiv. 2016;60822.
24. Pé Rez N, Gutierrez M, Vera N. Computational Performance Assessment of k-mer Counting Algorithms.
25. Erbert M, Rechner S, M??ller-Hannemann M. Gerbil: A fast and memory-efficient k-mer counter with GPU-support. Lect Notes Comput Sci (including Subser Lect Notes Artif Intell Lect Notes Bioinformatics). BioMed Central; 2016;9838:150–61.
26. Li Y, XifengYan. MSPKmerCounter: A Fast and Memory Efficient Approach for K-mer Counting. CsUcsbEdu [Internet]. 2015;1–7. Available from: http://arxiv.org/abs/1505.06550
27. Rizk G, Lavenier D, Chikhi R. DSK: K-mer counting with very low memory usage. Bioinformatics. 2013;29(5):652–3.
28. Pandey P, Bender MA, Johnson R, Patro R. Squeakr: An Exact and Approximate k-mer Counting System. bioRxiv [Internet]. 2017;1–7. Available from: http://www.biorxiv.org/content/early/2017/03/29/122077?%3Fcollection=
29. Melsted P, Pritchard JK. Efficient counting of k-mers in DNA sequences using a bloom filter. BMC Bioinformatics [Internet]. 2011;12(1):333. Available from: http://bmcbioinformatics.biomedcentral.com/articles/10.1186/1471-2105-12-333
30. Kokot M, Długosz M, Deorowicz S. KMC 3: counting and manipulating k-mer statistics. 2017;2(May):1–3. Available from: http://arxiv.org/abs/1701.08022
31. Kaplinski L, Lepamets M, Remm M. GenomeTester4: a toolkit for performing basic set operations - union, intersection and complement on k-mer lists. Gigascience [Internet]. GigaScience; 2015;4(1):58. Available from: http://download.springer.com/static/pdf/869/art%253A10.1186%252Fs13742-015-0097-y.pdf?originUrl=http%3A%2F%2Fgigascience.biomedcentral.com%2Farticle%2F10.1186%2Fs13742-015-0097-y&token2=exp=1495299499~acl=%2Fstatic%2Fpdf%2F869%2Fart%25253A10.1186%25252Fs1
32. Deorowicz S, Kokot M, Grabowski S, Debudaj-grabysz A. KMC 2 : Fast and resource-frugal k -mer counting by. :1–20.
33. Audano P, Vannberg F. KAnalyze: A fast versatile pipelined K-mer toolkit. Bioinformatics. 2014;30(14):2070–2.
34. Deorowicz S, Debudaj-grabysz A, Grabowski S. Disk-based k -mer counting on a PC. 2013;
35. Roy RS, Bhattacharya D, Schliep A. Turtle : Identifying frequent k -mers with cache-efficient algorithms. 2014;1–8.
36. Mamun A, Pal S, Rajasekaran S. Sequence analysis KCMBT : a k -mer Counter based on Multiple Burst Trees. 2016;32(June):2783–90.
37. Cormen T H, Leiserson C E RRL and SC. Chapter 11: Hash Tables. In: Introduction to Algorithms [Internet]. 2nd ed. MIT Press and McGraw-Hill; 2001. p. 221–45. Available from: http://is.ptithcm.edu.vn/~tdhuy/Programming/Introduction.to.Algorithms.pdf
38. Purcell C, Harris T. Non-blocking hashtables with open addressing. Lect Notes Comput Sci (including Subser Lect Notes Artif Intell Lect Notes Bioinformatics). 2005;3724 LNCS(639):108–21.
39. Gao H, Groote J, Hesselink W. Almost wait-free resizable hashtables. Parallel Distrib … [Internet]. 2004;0(C):1–37. Available from: http://www.win.tue.nl/~jfg/articles/CS-Report03-03.pdf%5Cnhttp://ieeexplore.ieee.org/xpls/abs_all.jsp?arnumber=1302969%5Cnhttp://ieeexplore.ieee.org/lpdocs/epic03/wrapper.htm?arnumber=1302969
40. Shalev O, Shavit N. Split-ordered lists: Lock-free extensible hash tables. J ACM JACM [Internet]. 2006;53(3):379–405. Available from: http://portal.acm.org/citation.cfm?id=1147954.1147958
41. Mapleson D, Accinelli GG, Kettleborough G, Wright J, Clavijo BJ, Marc J. KAT : A K-mer Analysis Toolkit to quality control NGS datasets and genome assemblies. 2016;(d):4–6.
42. Bloom BH. Space/Time Trade-offs in Hash Coding with Allowable Errors. 1970;13(7). Available from: https://www.ece.cmu.edu/~ece447/s13/lib/exe/fetch.php?media=p422-bloom.pdf
43. Putze F, Sanders P, Singler J. Cache-, hash-, and space-efficient bloom filters. J Exp Algorithmics. 2009;14:4.4.
44. David Salomon. Data compression: the complete reference [Internet]. Springer Science & Business Media; 2004. Available from: https://books.google.co.in/books?hl=en&lr=&id=PT1fcX321I4C&oi=fnd&pg=PR7&dq=%5B55%5D+Salomon+D+2004+Data+compression:+the+complete+reference.+Springer+Science+%26+Business+Media&ots=5iX8lXF0qJ&sig=_GM_InxCPFWXnbN3BZI_mACj8_k#v=onepage&q&f=false

45. Abouelhoda MI, Kurtz S, Ohlebusch E. Replacing suffix trees with enhanced suffix arrays. J Discret Algorithms. 2004;2(1 SPEC. ISS.):53–86.
46. Heinz S, Zobel J, Williams HE. Burst tries: a fast, efficient data structure for string keys. ACM Trans Inf Syst. 2002;20(2):192–223.
47. Li Y, Kamousi P, Han F, Yang S, Yan X, Suri S. Memory Efficient Minimum Substring Partitioning. 39th Int Conf Very Large Data Bases. 2013;6(3):169–80.
48. Kokot M, Deorowicz S, Debudaj-Grabysz A. Sorting Data on Ultra-Large Scale with RADULS. New Incarnation of Radix Sort. 2016; Available from: http://arxiv.org/abs/1612.02557
49. Melsted P, Halldórsson B V. KmerStream: Streaming algorithms for k-mer abundance estimation. Bioinformatics. 2014;30(24):3541–7.
50. Mohamadi H, Khan H, Birol I. ntCard: A streaming algorithm for cardinality estimation in genomics data. Bioinformatics. 2017;33(9):1324–30.
51. Chikhi R, Medvedev P. Informed and automated k-mer size selection for genome assembly. Bioinformatics. 2014;30(1):31–7.
52. Jr LCI, Brown CT. Efficient cardinality estimation for k-mers in large DNA sequencing data sets. 2016;1–5.
53. Crusoe MR, Alameldin HF, Awad S, Boucher E, Caldwell A, Cartwright R, et al. The khmer software package: enabling efficient nucleotide sequence analysis. F1000Research [Internet]. 2015;4:900. Available from: http://f1000research.com/articles/4-900/v1
54. Zhang Q, Pell J, Canino-Koning R, Howe AC, Brown CT. These are not the K-mers you are looking for: Efficient online K-mer counting using a probabilistic data structure. PLoS One. 2014;9(7).
55. Pell J, Hintze A, Canino-Koning R, Howe A, Tiedje JM, Brown CT. Scaling metagenome sequence assembly with probabilistic de Bruijn graphs. Proc Natl Acad Sci U S A [Internet]. 2012;109(33):13272–7. Available from: http://www.ncbi.nlm.nih.gov/pubmed/22847406%5Cnhttp://www.pubmedcentral.nih.gov/articlerender.fcgi?artid=PMC3421212
56. Pandey P, Bender MA, Johnson R. Counting quotient filter. http://www3.cs.stonybrook.edu/~rp/tech_reports/sbcstrc6ff764fdd8f9d2b5ea3b31972a787bc/report.pdf. [Online; accessed 29-Apr-2016].
57. Suzuki S, Masanori K, Ishida T, Akiyama Y. Accelerating identification of frequent *k*-mers in DNA sequences with GPU. In: GTC; 2014.

Click here to access/download
Supplementary Material
supplimentary.doc
