# Supplementary material for: A benchmark study of k-mer counting methods for high-throughput sequencing
Source: Gigascience. 2018 Oct 22;7(12):giy125. doi: 10.1093/gigascience/giy125 (PMC6280066; doi:10.1093/gigascience/giy125)
Supplement: GIGA-D-17-00245_Revision_1.pdf [file giy125_giga-d-17-00245_revision_1.pdf]

GigaScience

A benchmark study of k-mer counting methods for high-throughput sequencing

--Manuscript Draft--

Manuscript Number:	GIGA-D-17-00245R1
Full Title:	A benchmark study of k-mer counting methods for high-throughput sequencing
Article Type:	Review
Funding Information:	
Abstract:	High-throughput sequencing technologies have revolutionized the ways of producing gigabytes of data. Many applications of bioinformatics require counting substrings of length k in this data, e.g. genome and transcriptome assembly, error correction, multiple sequence alignment, repeat detection and other such applications. Several techniques for counting of k-mers in sequencing data have been developed in the recent years. All k-mer counting approaches aim to process such enormous amount of data in a way that realizes time and memory trade-off. This paper presents an assessment strategy for k-mer counting programs and evaluates their relative advantages and disadvantages. Counting performance is evaluated primarily on the basis of runtime and memory usage. Additional parameters like disk usage, parallelization, impact of compressed input, scalability with respect to both the larger k and large datasets like the human datasets during rigorous experimental analysis have also been considered herein to evaluate the performance of various k-mer counting tools. This review provides specific recommendations for the current state-of-the-art program for particular setup and provides suggestions for further development. All the tools evaluated in this article are freely available and can be downloaded from the hosting website.
Corresponding Author:	Swati Chandrakant Manekar, M.Tech.<br>Visvesvaraya National Institute of Technology<br>Nagpur, Maharashtra INDIA
Corresponding Author Secondary Information:	
Corresponding Author's Institution:	Visvesvaraya National Institute of Technology
Corresponding Author's Secondary Institution:	
First Author:	Swati Chandrakant Manekar, M.Tech.
First Author Secondary Information:	
Order of Authors:	Swati Chandrakant Manekar, M.Tech.
	Shailesh Sathe, Ph.D.
Order of Authors Secondary Information:	
Response to Reviewers:	Reply Letter (GIGA-D-17-00245)<br>Title : A benchmark study of k-mer counting methods for high-throughput sequencing<br><br>Authors : Swati Chandrakant Manekar, M.Tech.; Shailesh Sathe, Ph.D.<br>Date: 20th February 2018<br><br>Dear Editors,<br>We gratefully acknowledge the status of our manuscript and your review result. We are thankful to you for the time and effort spent by the reviewers to provide us their valuable insights and helpful feedback. The comments received from the reviewers helped to significantly improve our manuscript. We have worked hard to suffice each of the review comments with our responses. We believe we have incorporated all the comments and suggestions made by the reviewers and enhanced our manuscript wherever necessary. In this letter we address the comments individually with suitable responses. The comments from the reviewers are indicated in italic bold, followed by our response in normal text for each of them.

Powered by Editorial Manager® and ProduXion Manager® from Aries Systems Corporation

Reviewer #1:

Comment 1.1
In the testing of the k-mer counting tools, k = 22 and 55 were used. But later k size with range from 28 to 200 was used to do the testing. Firstly, it will be nice if the authors can explain more about the applications of the counting of long k-mers like 150, 175, 200. In most situation in bioinformatics, we do not count the k-mer with such long length.
>>
We thank the reviewer for a detailed review. The applications for counting of long k-mers (for k = 150 to 200) are appended in the manuscript with related references.

Text Added in manuscript is on the page no.5 line no.16-23, page no.6 line no 1-3. References are added to support the text added is on page no.33 line no.10-20.
>>

Comment 1.1.2
Secondly, if some of the k-mer counting tools make it clear that they do not support long k-mers counting, the authors may not need to actually test them and make them fail.
>>
We agree to the point raised herein, and hence, we have modified the text in this regards as KCMBT which does not support the longer k length has been removed from Figure 1 (old) titled “Analysis of time (second), memory (GB) and disk (GB) utilization of counting algorithms on AT and GT datasets for longer k length with, k = 28, 40, 55, 65, 100, 125, 150, 175 and 200” along with its description. Though the tools like BFCounter, KAnalyze 2.0.0 etc support long k-mer counting, they were unable to execute completely on AT and NC datasets. The reason possibly could be that they cannot handle longer length reads.
>>

Comment 1.2
One important application of k-mer counting is to get the frequency of a specific k-mer. It will be nice to test the performance of these tools to retrieve the frequencies of a group of k-mers. For example, this may be tested by retrieving the frequency of the first 100000 k-mers (or randomly selected k-mers) in a reads data set and measure the time, memory and disk usage of this process.
>>
Out of all the tools considered in this study, only five tools support the online retrieval of k-mer frequencies namely: 1) KCMBT 2) Jellyfish 3) MSPKmerCounter, 4) Tallymer and 5) Squeakr. These tools do not support the same forms of retrieval of the k-mers frequencies. For example:
1) KCMBT supports retrieval of a range of frequency of k-mers but not a k-mer or a group of k-mers.
2) MSPKmerCounter supports retrieval of a k-mer, one at a time but not a set of k-mers,
3) Jellyfish, Tallymer and Squeakr retrieve a set of k-mers.
Tallymer is not considered in our analysis, as we have only studied tools that were released after 2010. Squeakr is also not considered in this study as its exact k-mer counter code has not been released yet.
Hence, we could not test the performance of these tools, to retrieve the frequencies of a group of k-mers. As these tools support retrieval of varying types of input, a fair comparison amongst them cannot be achieved. Secondly, there is only one tool, Jellyfish, out of the other tools in this study that support the retrieval of a frequency of a set of k-mers.
>>

Comment 1.3
Related to the previous points, it will be nice to have a more comprehensive table listing more details of each k-mer counting tools, like the algorithm/data structure used, if the tool support long k-mers, or the limit of k-size, if the tool support online k-mer frequency retrieval, etc.
>>
In the original manuscript the section titled “Overview of k-mer counting approaches”

Powered by Editorial Manager® and ProduXion Manager® from Aries Systems Corporation

cover the information regarding data structure and the algorithm used by each tool. Hence the table covering comprehensive information of all the mentioned points as above is added in the supplementary (please see Table S1 along with its description). We believe that the original table titled "Approaches for k-mer counting" is the best way to show the ontology of k-mer counting approaches, which categorizes different tools according to the underlying principles they use as well as the relationship between these approaches (especially for those tools spanning multiple approaches or borrowing / using many different ideas). Hence, we kept the original Table 1 as it is in the manuscript.
>>

Comment 1.4
In Tables such as Table 4, Table 5, Table 6, some tools have varying results compared to other tools. Are the results purely wrong? If it is, what is the reason? Is it because the tools do not support the experiment by design? Here more discussions may be needed since the accuracy of the counting of these tools is important information to the readers.
>>
As suggested by Reviewer #2, Table 4-7 are merged and now reflected in two Tables numbered as A1 and A2 in the appendix with the removal of redundant data. The varying results may be due to the bugs in the considered recent versions of the tools. Actually we cannot state the exact reason for the non-matching results as there can be n number of reasons like synchronization problem while multithreading, estimation of the size of an array, etc. at the implementation level of the various approaches. We have taken special care while executing the tools by carefully setting all the parameters as per the guidelines given in the documentation of the tools. The answers to the above queries are reflected in the manuscript.

Text Added in manuscript is on the page no.18 line no.14-23, page no.19 line no 1-7.
>>

Comment 1.5
On Page 17, the authors made it clear that "The tools which give an approximation of k-mer counts histogram by streaming analysis of the data are not considered in this paper." However on Page 20 Line 14, the authors mentioned that "scTurtle is itself having some false positive results". What causes such false positive results? Is it some kind of approximation of k-mer counts? Does this contradict the previous statement?
>>
We agree to your point that scTurtle is not an exact k-mer counter. The scTurtle reports only an approximate count of the genuine k-mers because of its probabilistic nature (false positive results) owing to the underlying bloom filter it has used. Whereas BFCounter also uses a bloom filter, it makes one more pass to remove all the possibilities of false positive results (additional filtering to get exact results). To remove the contradiction as pointed out in the above comment, the scTurtle results are replaced by single threaded aTurtle (one of the variants of Turtle implementation) results as the aTurtle provides perfect counting. This benchmark study now considers only the exact k-mer counters. The paragraph related to aTurtle has been added to the manuscript.

Text Added in manuscript is on the page no.9 line no. 5-14.
>>

Comment 1.6
In Table2, "Genome size (M Base)", may be "M Bases".
>>
We have modified the above term in Table 2 titled as "Dataset specifications", on page number 16.
>>

Comment 1.7
On Page3, the description of the k-mer counting problem is a little bit confusing, which may need be rewritten, like the sentence on Line 10, "Every read of set S indicated by r with r[i] accounts for every ith character of r with index starting at 0 to l-1, where l is the length of r. "

Powered by Editorial Manager® and ProduXion Manager® from Aries Systems Corporation

>>
To respond to the above comment, the paragraph explaining the k-mer counting process in the introduction section has been carefully revised, and the changes made in the manuscript are as follows,

Text Added in manuscript is on the page no.3 line no. 6-16.
>>

Reviewer #2:
Major remarks:

Comment 2.1
Another k-mer counting benchmark is cited: "Computational Performance Assessment of k-mer Counting Algorithms" from Perez et al, 2016. I will agree that the current manuscript is much more up to date with respect to the software and versions. Please nevertheless discuss whether your conclusions are in line with what Perez et al report. Please also recall what the conclusions of Perez et al are, in your introduction.
>>
Our conclusions are in line with Perez et al. who have reported on parameters like time, memory utilization and parallelization. However, we have also tested the performance of the tools; with respect to additional parameters e.g. impact of compressed input (as suggested by reviewer #3) and scalability of different tools for long k-mers and accuracy. We have also tested the performance of tools on a range of datasets from small (D. Melanogaster) to large (human). We have considered only the exact k-mer counters as opposed to stochastic k-mer counters to provide a fair comparison. These parameters not only provide a good assessment of the actual performance of the k-mer counting programs but also provide a clear picture of the current state of the art programs used for counting of k-mers. Our study also considers the latest tools to date. We have added the supporting text for each such additional parameter in the manuscript.

Text Added in manuscript in Introduction Section, page no.5 line no.1-16
Text Added in manuscript in Result and discussion Section, page no.28 line no.20-21
>>

Comment 2.2
The authors put much similar emphasis on the memory usage and disk usage as on running time. However one should note that, from a user perspective, when it comes to counting large datasets such as human, it does not matter so much if a tool uses 100 GB of disk or 150 GB of disk, as this resource is generally plentiful. Thus, I would have appreciated a more pragmatic viewpoint when it comes to comparing tools with respect to the various metrics.
>>
We have agreed to your point, and hence we have mainly focused on the prime parameters of time and memory usage instead of focusing on disk usages of any tools. To support this, we have removed the old figure numbered as 2 titled “Analysis of disk (GB) utilization of the disk based algorithms for k-mer counting for increasing value of k, k = 8 and k = 55” from the manuscript along with its description. For newly added parameters like the impact of the compressed input file and multithreading analysis, the disk utilization is not discussed.
We responded to this comment by adding the paragraphs in the manuscript which gives a more pragmatic viewpoint, to the comparison of tools concerning the various metrics.

Text is added in Abstract and Result and discussion section
Text Added in manuscript page no.5, line no3-16
>>

Comment 2.3
The middle of the results section, for instance the specific details regarding how each tool was run, should be moved to the appendix (a section or table of subsidiary matter at the end of a book or document.).
>>
Thank you very much for your valuable comment. The specific details regarding how

each tool was run have been moved to the appendix (page no.2, 3 and 4).
>>

Comment 2.4
Tables 4,5,6,7 too. The authors nicely spotted that MSPKmerCounter and Gerbil have bugs, apparently yet all these tables in the main text contain a lot of redundant information.
>>
We are grateful for your comment regarding Tables 4,5,6,7 and redundant data. We have merged Table 4, Table 5, Table 6 and Table 7(old) now and have prepared two tables (Table A1 and A2) in the appendix. We have also done our best to remove the redundant data.
As suggested by Reviewer #1 the scTurtle results have been replaced by single threaded aTurtle (one of the variants of Turtle implementation) results. We have used aTurtle to perform the benchmark study having only exact k-mer counters. The paragraph related to aTurtle is also added to the manuscript.
>>

Minor remarks:

Comment 2.1*
grammar/typo: "till" in Introduction, "approache" page 5.
>>
Typo errors have been corrected carefully.
>>

Comment 2.2*
Formatting, lines 17 and 19 page 3 e.g. "S = {{ACGTTA}, {ACGTTT}}.A": lack of space after comma. Also why is the set "s" a set of sets? Counting k-mer implies returning the set s = {ACGT, CGTT, GTTT}, as it does not matter which read the k-mer comes from.
>>
The paragraph in the introduction section that explains the k-mer counting process has been rewritten carefully as the same was also suggested by Reviewer #1 and is now reflected in the manuscript.

Text Added in manuscript is on the page no.3, line no. 6-16.
>>

Comment 2.3*
Page 4 line 10, reference [15] is Jellyfish, and it does not perform k-mer frequency statistics.
>>
We agree to your point on Jellyfish, and a proper sentence with proper citation is added to the manuscript as follows,

Text added in manuscript on page no.4, line no.2-3 and reference is added on page no.32, line no.35
>>

Comment 2.4*
Page 5, regarding the sentence "In the light of this, many heuristic techniques and 2 approaches have been implemented in the various research works". Not all k-mer counters are heuristics, in fact the majority of them return exact results.
>>
We agree with your point and hence removed the sentences creating confusion from the manuscript.
>>
Comment 2.5*
Page 5, the sentence lacks citations: "Many memory efficient data structures used by various researchers can be listed as, enhanced suffix array, burst trie, lock free hash table, membership query data structure like bloom filter, pattern block bloom filter, counting quotient filter (CQF) and so on". Although I realize each of these objects will be described later, it is confusing to list them all here.
>>

Powered by Editorial Manager® and ProduXion Manager® from Aries Systems Corporation

	Sir, we agree with your comment. We have removed the sentence as mentioned above from the manuscript. Citations for each of the data structures referred have been added in manuscript at respective places.<br>Also to be in line with Reviewer #1 (Comment: to have a more comprehensive table listing more details of each k-mer counting tools, like the algorithm/data structure used, whether the tool supports long k-mers, or the limit of k-size, whether the tool support online k-mer frequency retrieval, etc.) we have added Table S1 titled ‘Overview of various k-mer counting tools’ in the supplementary.<br><br>Comment 2.6*<br>Page 5, please define "primary/secondary" memory.<br>>><br>To maintain uniformity throughout, we have used the terms memory and disk instead of primary and secondary memory respectively. The line including the above terms is added in the manuscript.<br><br>Text added in manuscript on page no.4, line no.17-18.<br>>><br><br>Comment 2.7*<br>Reference 24 is badly formatted.<br>>><br>The said reference is now correctly formatted and added to the manuscript.<br><br>Reference added in manuscript on page no.33, line no.8-9<br>>><br><br>Comment 2.8*<br>Page 5, the sentence "The presented benchmark study covers all the areas of evaluation where as the existing literature review is incomplete or inadequate" is quite vague.<br>>><br>We removed the inconsistency by modifying and adding supporting sentences in the manuscript.<br><br>Text added in manuscript on page no.5, line no.1-15.<br>>><br><br>Comment 2.9*<br>Page 5, the terms "capability to deal with enormous real world data" and "various categories" are vague.<br>>><br>When we use the term “capability to deal with enormous real-world data” we want to suggest that “the scalability of tools for processing large datasets like human datasets has also been considered in this benchmark study”. However, we modified the sentence containing the above terms with an additional sentence to improve the manuscript.<br><br>Text added in manuscript on page no.5, line no.1-15.<br>>><br><br>Comment 2.10*<br>GTester4 is actually named GenomeTester4. I would argue that the term "efficiently" in "efficiently uses the sort and count approach" page 6 is disputable, given that other tools also do that and yet are more efficient.<br>>><br>We have modified the sentence containing the above terms to improve the manuscript.<br><br>Text added in manuscript on page no. 7, line no.1.<br>>><br><br>Comment 2.11*<br>Page 7, the sentence "For k-mer counting, a typical data structure which can hold k-mer against its count is needed such as hash table [37] and is found suitable." reads

awkwardly.
>>
We have modified the sentence containing the above terms to improve the manuscript.

Text added in manuscript on page no. 7, line no.8.
>>

Comment 2.12*
Page 7, the terms "proper hash function" and "best possible location" are vague.
>>
The sentence containing the above terms has been removed from the manuscript. To above the repetition, the paragraph is removed. Because the use of hash table for k-mer counting has already been coved in the subsequent paragraph.
>>

Comment 2.13*
Page 8 and onwards, there are several more minor remarks regarding the use of the English language as well as the precision of technical terms, but I would rather read a revised manuscript after it has been proof-read by a native speaker.
>>
We have rigorously worked on the suggestions recommended by you and have worked hard to improve the quality of written English and precision of technical terms throughout the manuscript.
>>

Technical remarks:
Comment 2.1**
Bloom should be capitalized.
>>
Bloom is capitalized in the manuscript at the respective places.
>>

Comment 2.2**
scTurtle is not an exact k-mer counter, please mention it.
>>
We have mentioned that scTurtle is not an exact k-mer counter. As suggested by Reviewer #1 (Comment: On Page 17, the authors made it clear that "The tools which give an approximation of k-mer counts histogram by streaming analysis of the data are not considered in this paper." However, on Page 20 Line 14, the authors mentioned that "scTurtle is itself having some false positive results". What causes such false positive results? Is it some kind of approximation of k-mer counts? Does this contradict the previous statement?) we have replaced results of scTurtle with single threaded aTurtle (one of the variants of Turtle implementation) as the aTurtle provides perfect counting.

Text Added in manuscript is on the page no.9 line no. 5-14.
>>

Comment 2.3** and 2.4**

2.3** : Page 9, "Ability to modify [..] k-mers" is inaccurate, k-mers are not "modified".

2.4** : The description of CQF is unclear. For instance: What are the values of the array? What does it mean for an array to have a "key"? (dictionaries have keys, not arrays). How is CQF "better" in terms of loss-less compression of k-mers than count-min sketches? (Count-min sketches do not compress k-mers at all and are not even exact data structures.) Why are CQFs sensitive to high counts? ("highly distorted" is not the right term)
>>
Necessary changes have been made in the sentence containing the above terms, and we have removed the vague terms from the paragraph, explaining the CQF.

Text added in manuscript on page no. 9, line no.15-20
>>

Powered by Editorial Manager® and ProduXion Manager® from Aries Systems Corporation

Comment 2.5**
Page 10: no, a burst trie is not a modified suffix trie.
>>
We agree with your point Sir, and the necessary change has been made in the sentence containing the above terms.

Text added in manuscript on page no.10, line no.18-19
>>

Comment 2.6**
The KCMBT section overall reads quite awkwardly, it needs to be revised carefully.
>>
We have carefully revised the KCMBT section, to improve the manuscript.

Text added in manuscript on page no.10, line no.18-22 and page no.11, line no.1-17
>>

Comment 2.7**
Page 11: no, DSK does not use a single large hash table. (it uses multiple ones, as k-mers are partitioned).
>>
The sentence corresponding to the above point is rewritten.

Text added in manuscript on page no.12, line no.11
>>

Comment 2.8**
MSPKC is actually MSPKmerCounter.
>>
MSPKC is replaced with MSPKmerCounter at respective places in the manuscript, appendix and supplementary.
>>

Comment 2.9**
Page 7: the same tool (khmer) is described 4 times.
>>
We accept your point, Sir. We have described the latest implementation/approach of khmer, published under the title "Efficient cardinality estimation for k-mers in large DNA sequencing datasets." only in the manuscript. The old khmer implementations/approaches have been removed from the manuscript.

Text added in manuscript on page no. 16, line no.14-16
>>

Comment 2.10**
Note: DSK handles k > 127 when it is recompiled (as per the github readme).
>>
Sir, we recompiled the code and took the reading for k = 28,40, 55, 60,100,125,150,175 and 200 once again for datasets NC and AT, and the changes are reflected as shown in Figure No.1 on page no.28 along with the modified description in the manuscript.
>>

Reviewer #3
Comment 3.1
As a general, minor comment; though the manuscript is nicely laid out, the quality of the written English should be improved. There are a number of places with awkward phrasing, typos, etc.
>>
We have rigorously worked on the suggestions recommended by you and have worked hard to improve the quality of written English throughout the manuscript. We have also removed the awkward phrasing, typos, etc. as suggested by you.
>>

Major technical comments

Comment 3.1*
For example, the authors state that input datasets are decompressed and "concatenated" into a single input file before running each of the tools. While this may seem a reasonable approach to normalize for potential differences (e.g., if not all of the tools support decompression directly), it may have a non-trivial (significant.)effect on the results as they could deviate from how the tools might be used following best practices. Specifically, it is common to run most k-mer counting tools directly on the compressed files without first decompressing the reads. This has the benefit, especially on "traditional" hard drives, of improving the I/O throughput and allowing the actual counting algorithms to consume data faster.
This isbecause certain compression schemes like gzip have quite limited computational overhead, and so the cost of having to decompress the data in memory is overcome by the corresponding increase in data throughput that results from reading compressed data from disk. Since this is likely a common strategy for running k-mer counters, it should be included in the existing benchmarks (at least in addition to the current benchmarks).
Another potential issue stems from normalizing the data by concatenating the reads into a single file. Some k-mer counting tools (e.g. KMC2, though I am not entirely certain of the changes made in KMC3) effectively perform parallelization in their first phase by reading from individual input files using separate threads. This means that restricting the input to a single file effectively limits them to 1 or 2 threads (e.g., one to parse and one to bin / partition). Given KMC3 %CPU utilization numbers this may, indeed, be occurring. It looks as though parallelism effectively increases when KMC3 enters the second phase (that doesn't depend on input), but it would likely be able to do even better on multi-file inputs if those inputs were not first concatenated into a single file. This may also be true of other tools.
>>
Thank you, sir, for the above comment. The benchmarking of all the k-mer counting tools supporting compressed input is performed by running them on compressed input files (gzip/bz2), and the results are uploaded in table numbered as 4, 5, 6, 7 and 8. The related description has also been added in manuscript.

Text added in manuscript on page no.29 and line no. 2-18
>>

Comment 3.2*
My final concern is not, unfortunately, one where I am able to offer a great solution. That is, given the wide variation in adopted settings, it's not completely clear to me that the CPU resource comparisons are fair. For example, when different programs are executed with different numbers of threads, their running times aren't directly comparable. Moreover, given the difficulty in achieving "perfect scaling", it's not necessarily much better to examine non-wall-clock-time metrics. I realize that it is not always possible to compare all programs with exactly equal parameters --- as some programs, for example, restrict the specific number of threads that can / must be used. Perhaps, in this case, the best approach to analyzing CPU-related performance comprehensively is to run each program using a few configurations in terms of the number of threads. Though things may still not be directly comparable, it will at least give some insight into the scaling properties of the different tools with respect to the number of threads they are allowed to use.
>>
We agree to the suggestion made herein, and we have performed the benchmark study of all the tools using the following number of threads (thread = 1, 2, 4, 6, 8 and 12) on two different datasets, FV and MB. The results of which (speedup and memory plot) are shown in the figure no. 2 and the corresponding readings are added to the supplementary document (Table S8 – S9). The related description is added in the manuscript.

Text added in manuscript on page no. 29 and 30
>>

Powered by Editorial Manager® and ProduXion Manager® from Aries Systems Corporation

	Comment 3.3*<br>As an aside, when certain in-memory tools were not able to complete a task in the specified time or resources (e.g., running out of memory), I wonder if the authors considered the strategies such programs had to minimize RAM usage. For example, Jellyfish 2 supports filtering "erroneous" k-mers using a Bloom filter. This capability is mentioned in the introduction. However, I wonder if the authors attempted to use this feature when Jellyfish 2 exceeded the allocated resources when processing certain datasets. It is possible that other programs also expose such options. Perhaps it is reasonable to avoid benchmarking all tools in such "non-standard" configurations, nonetheless, it would be useful to know if it is at least possible to allow such tools to complete processing these datasets on the benchmarking machine.<br>>><br>Thank you, sir, for your valuable comment. As on our benchmarking machine Jellyfish in its default mode could not execute on HS1. Hence, we tried to run Jellyfish in BF-based mode on HS1 for both values of k (28 and 55). But as Jellyfish's - BF-based mode for phase 1 could not finish execution within 15 hours for both k values and as the system froze, we had to terminate its execution forcefully.<br>Jellyfish was also tested in the BF-based mode for dataset MB (M. balbisiana). Where its performance degraded regarding time (by 21% for k = 28 and 45% for k = 55) and memory (by 25% for k = 28 and 45% for k = 55) as compared to the Jellyfish's default mode. The results of execution for Jellyfish in BF-based mode on dataset MB for k = 28 and k = 55 are added in supplementary documents.<br><br>Text added in manuscript on page no. 20 , line no.1-3<br>>>
**Additional Information:**	
**Question**	**Response**
Are you submitting this manuscript to a special series or article collection?	No
**Experimental design and statistics**<br><br>Full details of the experimental design and statistical methods used should be given in the Methods section, as detailed in our Minimum Standards Reporting Checklist. Information essential to interpreting the data presented should be made available in the figure legends.<br><br>Have you included all the information requested in your manuscript?	Yes
**Resources**<br><br>A description of all resources used, including antibodies, cell lines, animals and software tools, with enough information to allow them to be uniquely identified, should be included in the Methods section. Authors are strongly encouraged to cite Research Resource Identifiers (RRIDs) for antibodies, model organisms and tools, where possible.<br><br>Have you included the information	Yes

requested as detailed in our Minimum Standards Reporting Checklist?	
**Availability of data and materials**<br><br>All datasets and code on which the conclusions of the paper rely must be either included in your submission or deposited in publicly available repositories (where available and ethically appropriate), referencing such data using a unique identifier in the references and in the “Availability of Data and Materials” section of your manuscript.<br><br>Have you have met the above requirement as detailed in our Minimum Standards Reporting Checklist?	Yes

Manuscript Click here to download Manuscript manuscript_revised.doc

A benchmark study of *k*-mer counting methods for high-throughput sequencing

Swati C. Manekar[1*] and Shailesh R. Sathe[1]

[1]Department of Computer Science and Engineering, Visvesvaraya National Institute of Technology, Nagpur 440 010, India

*corresponding author, Email: swati.manekar@gmail.com

Abstract

High-throughput sequencing technologies have revolutionized the ways of producing gigabytes of data. Many applications of bioinformatics require counting substrings of length *k* in this data, e.g. genome and transcriptome assembly, error correction, multiple sequence alignment, repeat detection and other such applications. Several techniques for counting of *k*-mers in sequencing data have been developed in the recent years. All *k*-mer counting approaches aim to process such enormous amount of data in a way that realizes time and memory trade-off. This paper presents an assessment strategy for *k*-mer counting programs and evaluates their relative advantages and disadvantages. Counting performance is evaluated primarily on the basis of runtime and memory usage. Additional parameters like disk usage, parallelization, impact of compressed input, scalability with respect to both the larger *k* and large datasets like the human datasets during rigorous experimental analysis have also been considered herein to evaluate the performance of various *k*-mer counting tools. This review provides specific recommendations for the current state-of-the-art program for particular setup and provides suggestions for further development. All the tools evaluated in this article are freely available and can be downloaded from the hosting website.

Keywords: *k*-mer counting, high-throughput sequencing, disk-based counting, in-memory counting, hash table, sorting.

Introduction

k-mer counting is an important step of many bioinformatics applications used in the analysis of sequencing data. Several tools and techniques have evolved in last few years to count the frequency of *k* length substring (*k*-mer) in the sequencing reads generated from high-throughput sequencing [1].

k-mer counting is a process of counting the number of occurrences of every substring of length *k* in a string *S* or a set of strings, where *k* is a positive integer. Let ∑= {*A*, *C*, *G*, *T*, *N*} denotes alphabet of nucleotides of DNA sequence, where *N* denotes the undetermined character by the sequencer. A read say *r* is a sequence of nucleotide over alphabet ∑. In a sequence dataset, different reads can contain the same sequence of nucleotides. Let *R* denote the dataset having *n* number of reads such that $R = \{r_i; 1 \leq i \leq n\}$.

Let's consider an example dataset *R* containing 3 reads of length 6, *R* = {*ACGTTA*; *ACGTTA;* *ACGTTT*}, having 2 sequences {*ACGTTA*; *ACGTTT*}, for *k* = 4, there are nine 4-mers (3 in each read) {*ACGT*; *CGTT*; *GTTA*; *ACGT*; *CGTT*; *GTTA*; *ACGT*; *CGTT*; *GTTT*}, on counting, there are four unique 4-mers which can be represented along with their counts as {*ACGT*(3); *CGTT*(3); *GTTA*(2); *GTTT*(1)} [2].

Counting *k*-mers is applied in the de novo genome assembly viz., the overlap layout consensus approach [3, 4] and the de Bruijn graph [5-8] based assembly. It has application in transcriptome assembly and in identifying the protein binding sites [9]. The error correction of reads is done to improve genome assembly quality. Error correction based on *k*-mer spectrum approach [10-13] and multiple sequence alignment based approach [14] also uses the frequency count of *k*-mers. The probable misalignment in the reads is either due to errors or genuine nucleotide variations

can be estimated using *k*-mer frequencies. *k*-mer counting also aids in multiple sequence alignment of protein sequences [15]. The size of the genome and sequencing depth can be estimated using *k*-mer frequency statistics [16]. de novo repeat annotation techniques like ReAS makes use of high-frequency *k*-mer as a seed to find repeats [17], whereas RepeatScout also uses the same to build a set of repeat families [18]. To identify the repeats in the genome, precompiled repeat library have been used in RAP [19] and FORRepeats [20]. This library is also used to identify the exact word matches to annotate large genomes [21]. Tallymer [22] also uses *k*-mer frequencies to annotate repetitive plant genome. Duplication studies have been conducted to quantify complex repetitive features of DNA in several genomes for varying lengths of *k*-mers [23]. The count of *k*-mers has also been used to infer the genotypes of known variants [24].

Although, *k*-mer counting is a simple and straightforward task, it becomes very difficult when billions of next-generation sequencing (NGS) data needs to be processed. The simple and basic approach for counting *k*-mers can be implemented using an array with substring indexing. This approach becomes infeasible in terms of memory and time when there are billions of input reads. The approaches proposed so far have mainly targeted memory efficient solution for *k*-mer counting. One of the ways, to achieve memory efficiency is representation of the string data as an integer. The disk is always a magnitude cheaper than the memory; therefore many researchers have focused on using the disk for scaling to larger datasets. The approach is termed as disked based/ external memory/ out-of-core approach as opposed to in-memory/ internal memory approach.

In this article, a review of such *k*-mer counting approaches for high throughput sequencing data and their comparative evaluation is presented. The main purpose of this article is to provide a general set of benchmarks and an assessment matrix. It also covers the experimental analysis of

k-mer counting tools giving a thorough insight to both the beginners and consultants. Perez et al. [25] studied various *k*-mer counting tools for *k*-mer lengths of 31 and 55, on a single dataset. With a quest to benchmark the performance of tools including the latest ones, we did an exhaustive study considering multiple datasets for evaluation. Majorly, all the *k*-mer tools are evaluated for runtime and memory usage in the present study. However, the present benchmark study also considered several other parameters in addition to the existing state-of-the-art literature for evaluation of *k*-mer counting tools. Like, for disk-based approaches, we also record the maximum temporary disk usage. Time, CPU, memory are the bounded (limited) resources, where as disk can be considered as plentiful resource. The disk-based approach may additionally use hundreds of gigabytes of disk space for a large dataset like human, they achieve a very high efficiency at a marginal increase in the cost. Additionally, we consider parameters like accuracy, the impact of compressed input i.e. gzip and bzip2 (multiple compressed FASTA/FASTQ input files), the multithreaded analysis and scalability of *k*-mer counting approaches on a wide range of real datasets. The scalability of each tool with respect to the larger values of *k* is also evaluated by considering datasets having longer reads.

In bioinformatics, with the advancement in next-generation sequencing technologies, longer reads are generated. Such longer reads have many applications in genome assembly projects as it aids in giving high consensus accuracy, uniform coverage, detection of epigenetic modifications, better resolution of structural variants as well as genomic repeat content [26], etc. However, longer reads of Illumina technology suffers from less accuracy [27]. Empirically, *k*-mers larger than two-thirds of the read length can correct errors if sequencing coverage is high [28]. Longer *k* sizes (up to 200) are used to improve the accuracy of long sequencing reads especially in repeats. Empirically it is shown that best assemblies (without miss-assembly) and highest N50

are obtained at an optimal choice of *k* which seems to be larger value of *k* [29, 30]. Considering such applications of the larger length of *k*, we test the scalability of various *k*-mer counting tools with respect to longer *k*-mers.

The article is organized as follows. We first present the algorithmic study of each approach. The next section introduces the tools considered for benchmark study, followed by datasets used and multiple dimensions (parameters) taken into account for the assessment. The results of comparisons are discussed in the subsequent section. And finally, we conclude with the guidelines and future research directions.

Overview of *k*-mer counting approaches

The *k*-mer counting tools can be categorised depending on the approaches and data structure used, as shown in Table 1. For more comprehensive information regarding each *k*-mer counting tools like the approach, the data structure it uses, the *k*-size it can handle, etc. (please see the Supplementary Table S1).

Table 1 Approaches for *k*-mer counting

**Approach for *k*-mer counting**	**Disk-based**	**In-memory**
Hash-table	Gerbil [31], MSPKmerCounter [32], DSK [33]	Squeakr [34], Jellyfish [35], BFCounter [36]
Sorting	KMC3 [37], GenomeTester4 [38], KMC2 [39], KAnalyze[40], KMC1 [41]	Turtle [42]
Burst tries	-	KCMBT [43]
Enhanced suffix array	-	Tallymer [22]

k-mer counting using sorting approach

In the sorting approach, the *k*-mers are first sorted in lexicographical order. *k*-mers are then easily counted as similar *k*-mers lie adjacent to each other in a sorted list.

GenomeTester4 [38] uses the sort and count approach for *k*-mer counting. The algorithm is implemented in two phases wherein, in the reading phase, temporary arrays are used to gather all *k*-mers from the input file. During the collation phase, *k*-mers in the arrays are first sorted and then counted. The temporary table outputs are then merged to produce the final *k*-mer count list. GenomeTester4 cannot process large dataset of NGS reads containing many repeated *k*-mers. The running time and memory requirement of the algorithm depends on the size of the input.

k-mer counting using a hash table

For *k*-mer counting, a hash table [44] is used which hold *k*-mer against its count. Jellyfish [35] uses a hash table for *k*-mer counting. It introduces lock-free hash table to allow parallel insertion of *k*-mers and frequency updates by multiple threads using CAS (compare-and-swap) assembly instruction [45]. CAS detects simultaneous access to a shared memory location in a multithreaded environment. For storing hash table, the entire memory is used. When a hash table becomes full, instead of doubling the size of the hash table in the memory, it gets written to the disk, and intermediate *k*-mer counts are then merged [46, 47]. At runtime, it requires the user to specify an estimated number of distinct *k*-mers (the size of the hash table).

To minimize memory usage of *k*-mers stored in the hash table, Jellyfish follows a ‘quotienting’ technique and works as follows: Whenever a new *k*-mer appears, its key is obtained and it is searched in the hash table; if it exists in the hash table its frequency count is incremented by 1; otherwise, this *k*-mer is inserted into the hash table using reprove strategy by setting its initial frequency equal to 1. If a collision occurs, it is resolved by using quadratic probing (open addressing) technique [44].

A more efficient version of Jellyfish is available as Jellyfish 2, with additional BF-based mode that implements Bloom filter to remove all singleton *k*-mers. A modified version of the Jellyfish 2 library is used by KAT [48] for *k*-mer counting.

k-mer counting with the application of Bloom filter, its variants and counting quotient filter

A major part of the entire genomic data set is consumed by single frequency *k*-mers which are mainly due to sequencing errors. A Bloom filter [49] is a probabilistic data structure used for dynamic membership query lookup which can implicitly store all *k*-mers. It is used to filter out such single frequency *k*-mers. The frequency of every non-singleton *k*-mer is then counted using any one of the approaches like hashing or sorting approach. The Bloom filter has some amount of false positive membership query results which may lead to miscounting of *k*-mers. However, with a reasonable choice of the number of hash functions, the false positive rate can be minimised to an acceptable degree [50]. Though Bloom filter has some false positive membership query results, it requires very low memory, i.e. only to store a bit vector, which highly reduces the overall memory requirement.

BFCounter [36] uses the same concept of Bloom filter to filter out singleton *k*-mers and uses the hash table to store and count the non-singleton *k*-mers. Some percent of singleton *k*-mers may also get added in a hash table because of false positives giving erroneous counting results. However BFCounter generates correct results by reiterating over the sequence reads.

Pattern block Bloom filter [51] is a cache-friendly variant of the Bloom filter in which the cache miss ratio is very small.

Turtle [42] uses a novel sorting and compaction (SAC) based algorithm instead of the hash table which is memory efficient solution for counting *k*-mers. In this approach, the *k*-mers are added to a big array up to a certain point with a frequency equal to one. The array is then sorted

once an array is full, the identical *k*-mers are then compacted and their counts are added up in the compaction step. The compaction process frees up space in the array which is used further to count the rest of the *k*-mers iteratively following the SAC approach on the entire length of the array. The process is repeated till all *k*-mers are counted. The compaction process is similar to run-length encoding [52]. Turtle has three implementations, scTurtle, cTurtle and aTurtle varying in their output formats. scTurtle [42] uses a pattern block Bloom filter to remove all single occurrence (spurious) *k*-mers. All such non-spurious *k*-mers with frequency > 1 are then counted using SAC approach. cTurtle accepts small rate of false positive and false negative, which gives an approximate set of frequent *k*-mers by using a counting Bloom filter.

scTurtle outputs only *k*-mer with frequency >1 whereas cTurtle gives only *k*-mer with frequency >1 without any counts and aTurtle provides *k*-mer with all frequencies along with their counts. Although being multithreaded, cTurtle and scTurtle do not give the perfect counting, whereas single threaded aTurtle provides a perfect *k*-mer count. Hence for benchmarking purpose, we have selected only aTurtle which provides perfect counting.

Squeakr [34] is another in-memory approach for both the approximate and exact *k*-mer counting. It uses a counting filter data structure (counting quotient filter (CQF) [53]) to store the counts of *k*-mers. The *k*-mers are hashed using a one-way hash function and the hashes are stored in the counting filter. The Squeakr representation of the *k*-mer multiset processes fast queries and dynamic *k*-mer insertion, deletion, and modification and is therefore suitable for downstream processing e.g. de Bruijn graph traversal.

The algorithm consists of a single phase. Multiple threads read data from the disk in chunks and insert *k*-mers simultaneously into a global shared thread safe CQF for *k*-mer counting. To reduce waiting while acquiring a lock in the global CQF (hardest to acquire the lock when

repetitive *k*-mers are present in dataset), each thread maintains its local CQF to keep the counts of *k*-mers temporarily. Once the local CQF is full, it dumps the temporary counted *k*-mers into global CQF before processing a new set of *k*-mers. Squeakr(exact) which counts the frequency of each *k*-mer exactly is not considered in our benchmark study. This is because the code for Squeakr(exact) which can list *k*-mers with their counts is not available yet (currently not suitable for benchmark study).

Enhanced suffix array based counting

Tallymer [22] is an in-memory approach which uses (longest common prefix) lcp-interval tree constructed from an enhanced suffix array [54] for k-mer counting. lcp-interval tree implicitly stores the number of occurrences of all substrings of *s*' (reads are concatenated into a string *s*' with unique termination symbol ($\$_i$) appended to each read). The algorithm is implemented in two steps: (i) the divide step split sequence *s*' into smaller distinct partitions. The *k*-mers in each such partitions are then counted using the lcp-interval tree (ii) a final count is generated by merging the counts generated from all distinct partitions (using the sequence *s*'). Suffix array construction of a string is expensive in terms of computation and memory requirement. Suffix array size increases linearly with the size of the genome and its coverage.

Trie data structure based k-mer counting

KCMBT [43] is an in-memory approach which uses burst trie [55] which is a variant form of a trie. Burst trie manages large sets of strings efficiently in the memory and maintains strings in sorted or nearly-sorted order. Like KMC2, it implements extended *k*-mer. Extended *k*-mer ((k + x)-mers for $x > 0$) is a substring of length more than *k*. The KCMBT consists of three phases as below.

In the first phase, (k + x)-mers are generated from input reads and inserted into the corresponding trees. For inserting (k + x)-mers into trees, a fixed length container is maintained initially for each tree. When a container is full, it bursts, which means it is replaced by a new trie node and a set of child containers that partition between themselves (k + x)-mers of the original container depending upon the prefix A, C, G and T (x is chosen empirically to be $0 \leq x \leq 3$ for better performance). In the second phase all (k + 1)-mer, (k + 2)-mer and (k + 3)-mer trees are traversed to get their counts [42]. Now, these (k + x)-mers are converted to k-mers. As from the counts of the (k + x)-mers the counts of its constituents k-mers are obtained because (k + x)-mer covers (x + 1) k-mers. Then each k-mers is inserted into the k-mer tree having the same prefix. Finally, the k-mer trees are traversed to produce the counts of all the unique k-mers. The k-mers along with their counts are then written to the disk.

As the inserted numbers of k-mers have been reduced a lot due to (k + x)-mers, the time for traversal in the last phase is reduced. Because of the (k + x)-mer the computation becomes fast, as the number of insertions is minimized. To reduce overall insertion and traversal time for a huge number of k-mers, thousands of trees with smaller heights are generated. Burst trie is a data structure where the search is very fast, but the size of the trie becomes large when input size is high.

Disked-based *k*-mer counting

Furthermore, huge memory requirement of the in-memory approach is reduced by disk-based approaches. The disk-based approach is a memory frugal approach especially designed to make the k-mer counting possible for large genomic datasets like a human dataset on commodity hardware. With the use of a disk, memory usage can be greatly minimized as k-mers are processed in chunks and are stored on to the disk. In the following section, the disk-based

approaches like DSK, KAnalayze, MSPKmerCounter, KMC2, KMC3 and Gerbil are presented along with their comparative analyses.

DSK [33] is a disk-based algorithm which counts the *k*-mers using very low memory and disk space. It achieves this by calculating the number of partitions needed to bring data in parts from the disk to the memory. This depends on (i) the total bits required to store the *k*-mers and (ii) disk size available. It calculates the number of iterations required to process the entire set of input reads in parts, depending on, (i) the total number of bits required to represent the entire set of *k*-mers, (ii) memory size to hold the hash table, (iii) number of partitions and (iv) load factor for which hash table gives the best performance. The extracted *k*-mers are distributed to the partitions depending on their hash values and iteration number. Partitions are stored on the disk. *k*-mers are counted by loading a partition on to the memory one at a time using hash tables in multiple iterations. DSK incorporates an efficient partitioning strategy implemented to deal with memory constraints though it may turn out to be high I/O demanding.

KAnalyze [40] is a *k*-mer toolkit which implements disk-based approach that uses sorting for counting. In the initial phase, *k*-mers are filled into a temporary array of predefined size. Once the array is full, *k*-mers are sorted and counted. The counted *k*-mers are then written to the disk so that space becomes available to count the next incoming chunk of *k*-mers. This process is repeated till all the *k*-mers are processed. In the second phase, the count files are loaded from the disk to the memory and are merged in multiple steps to generate a final count of *k*-mers.

Approaches using concept of super k-mer: Minimizer and Signatures

To further minimise memory requirement and I/O operations, the disk-based compression technique, i.e. MSP (Minimum Substring Partitioning) [56] is used wherein input reads are broken into multiple disjoint partitions.

k-mers of reads carries highly redundant data, as there exists an adjacency relationship between every pair of *k*-mers. In MSP approach the consecutive *k*-mers of a read, if sharing the same lexicographical minimum substring *s*, the k-mers are stored as one single substring of length greater than *k*. This substring is called as super *k*-mer and is stored into a disk partition corresponding to the lexicographical minimum substring *s*, where *s* is termed as minimizer. If larger number of several consecutive *k*-mers, say *x*, share the same minimum substring *s* then, a high compression ratio is achieved which ultimately reduces both the I/O overhead and storage space.

MSPKmerCounter [32] is the first to implement MSP in *k*-mer counting. The algorithm works as follows; (i) The reads are first decomposed into super *k*-mers and distributed to the respective disk partitions (bins) identified by canonical minimizer. Super *k*-mers sharing the same canonical minimizer in a partition assures that the same *k*-mers will always be in the same partition. This eliminates the step of merging the counts of each partition. These smaller partitions are easily accommodated into the main memory and are processed independently. (ii) All super *k*-mers are then broken into *k*-mers using simple bit shift operation once partitions are ready. (iii) Finally, *k*-mers are counted using hash tables, and the count is stored on the disk.

KMC2 [39] is another disk-based approach that uses the same approach of MSP as employed in MSPKmerCounter [32]. Minimizer suffers from an imbalance in bin size. KMC2 addresses this problem using the concept of signature that reduces the overall memory and disk space usage. Signature is a canonical minimizer with the following three pre-requisites: (1) it should not begin with the prefix *AAA* (2) it should not begin with the prefix *ACA* and (3) it can start with a prefix *AA*, however *AA* should not occur anywhere else.

The KMC2 algorithm consists of two major phases, distribution phase and sorting phase. The first phase is similar to the first phase of MSPKmerCounter, with the only difference of the super *k*-mers being distributed to the different temporary files (bins) based on the signatures instead of a minimizer. In the second phase, bins are processed by bringing them into memory. For every such bin, extended *k*-mers, i.e. ($k + x$)-mers are then extracted from the super *k*-mer, and radix sort is applied on them. *k*-mer statistics (*k*-mers with their frequencies) are then collected from these sorted ($k + x$)-mers. Finally, results are stored onto the disk. KMC3 is an extension of KMC2 approach having few improvements such as (i) efficient input file reading to achieve better I/O subsystem (ii) memory efficient approach of assigning signatures to block (iii) KMC3 has its efficient sorting approach [57] rather than radix sort to make it efficiently work for larger values of *k*.

Gerbil [31] uses hashing approach for counting like the DSK. It uses the concept of super *k*-mer with minimizer for *k*-mer grouping like MSPKmerCounter and the ranking of minimizers (signatures) similar to KMC2. It consists of two major phases. In the first phase, the minimizers of all *k*-mers of the input reads are computed. All the super *k*-mers sharing same minimizers are allocated to the same temporary file and are stored on the working disk. This stage is similar to that of KMC2 with a little advancement. To make sure that multiple occurrences of the same *k*-mer get assigned to the same thread in a subset, hash values of *k*-mers (extracted from super *k*-mers) are used. This is achieved with the application of a part hash function. In the second phase, the temporary files are sequentially re-read from the working disk and *k*-mers are counted by a hash table using quadratic hashing for collision handling. Every thread counts the assigned *k*-mers using its own hash table. Finally, the hash table containing the counts of *k*-mer is written into an output file. In its GPU implementation, the second phase is performed on the GPU side

with proper load balancing between GPU and CPU. The algorithm avails high paralleliztion, and the hash table size is estimated using a simple linear model.

Benchmark data-sets and evaluation methodology

A total of seven data sets are chosen for evaluating the performance of various tools. Most of these datasets are referred from Kokot et al. [37] to have a reasonable assessment. Of these seven datasets (Table 2), FV and DM are two small sized data sets while HS2 is the largest one. To evaluate the scalability of each tool with respect to larger datasets, HS1 and HS2 are used in this study. To test the performance of various tools for higher *k* values, datasets with long reads are chosen like the one used by Erbert et al. [30]. NC and AT are longer read datasets having average read length of 7778.3 and 4804.6 respectively. The datasets chosen are from actual experiments which include a variety of genomic coverage and read lengths. The details of the data sets used are summarized in Table 2. The datasets available in compressed form gzip and bz2 are first decompressed and then concatenated into a single FASTA/FASTQ files for tool execution. The information for downloading all used datasets is listed in the supplementary document (Table S2).

Some *k*-mer counting tools can directly run on the compressed files without decompression of reads (without normalizing the input data). In such case, the tools can effectively perform parallelization in their first phase by reading from individual input files using separate threads. This means that restricting the input to a single file effectively limits them to 1 or 2 threads (e.g., one to parse and one to bin/partition). It is likely that most of the tools would be able to do even better on multi-file inputs without being concatenated into a single

file. Hence, we test the performance of various tools by running them directly on compressed input files wherever supported.

Table 2 Dataset specifications

Sr. No.	Data set ID	Organism	Genome size (M Bases)	Input FASTQ file size (GigaBytes)	Average read length(Bases)	Total No. Of bases (G Bases)	Total no. of reads
1	FV	F. vesca	214	10.9	353	4.5	12803137
2	DM	D. melanogaster	122	10.5	76	3.7	48432878
3	MB	M. balbisiana	472	197.1	100	56.3	562968372
4	HS1	H. sapiens 1	2,991	292.1	151	123.7	819148264
5	HS2	H. sapiens 2	2,991	339.5	100	135.3	1339740542
6	NC	N. crassa	41	23.3	7778.3	22.9	2942564
7	AT	A. thaliana	120	72.7	4804.6	36.1	7515360

We evaluate KMC3, Gerbil (version 1.0), KCMBT (version 1.0), MSPKmerCounter (version 0.1), GenomeTester4 (version 4.0), aTurtle (version 0.3), kAanalyze (version 2.0.0), DSK (version 2.2.00), Jellyfish (version 2.2.6), and BFCounter (version 1.0) according to their chronological order of their release. We consider the recent versions of the tools that were released after 2010, for the benchmark study. The tools are freely available to download (refer Table S2 of the supplementary document).

The tools which give an approximation of *k*-mer counts histogram by streaming analysis of the data are not considered in this paper. Like KmerStreame [58], ntCard [59] and KmerGenie [30] implement a streaming algorithm, estimating only the *k*-mer abundance histogram. Similarly, khmer [60] the latest one built on library [61] uses hyperloglog which is the probabilistic approach for approximate cardinality estimation. All these tools use a significantly lower amount of memory and are reasonably fast. In this study, we test only those tools which generate exact *k*-mer counts to have a fair comparison.

Time is recorded using function ‘gettimeofday’ in c++ which considers the wall clock time. For each tool, disk, memory and % CPU utilizations are recorded simultaneously. A shell script

by Jaeho Shin is used to calculate memory usage which monitors the *rss* peak for a multithreaded program with a sampling rate of 1 (downloaded from https://github.com/jhclark/memusg). It simply watches the values given by '*ps* -o *rss*=' where *rss* is the real memory (resident set) size of the process. The script calculates the memory usage of the whole process tree using Linux *ps* command considering every forked child process. Shell scripts implemented using Linux command *du* and *top* are used to record temporary disk utilization and average % CPU utilization respectively. These scripts are executed with a sampling rate of 3 for all larger datasets (HS1 and HS2) and 1 for smaller datasets (FV, DM, MB, NC and AT).

All the experiments were performed on a machine with the configuration as shown in Table 3. Commands used to execute the tools are adopted from the documentation of respective tools and from the publications of KMC3 and KMC2. The commands used for *k*-mer counting, dumping results and histogram generation are given in the supplementary document.

Table 3 Machine Configuration

**Processor**	Intel(R) Xeon(R) CPU E5-2698 v3 @ 2.30GHz
**Main memory**	64GB
**Hard Disk Drive**	1 TB
**CPU(s)**	16
**On-line CPU(s) list**	0-15
**Thread(s) per core**	2
**Core(s) per socket**	16
**No of socket**	1

Considering time and memory limitations, two small size datasets, i.e. FV and DM are used for validating the results of each tool. The accuracy of all the tools presented in our study is tested using the frequency count histogram, which is obtained in two steps. Firstly, the output in the form of *k*-mers with their count is dumped into a single file by the tools. Next, a parallel testing program using OpenMP with Linux command *grep* and *wc* (word count) produces the *k*-mer frequency count histogram from this output file. Some tools like Jellyfish, DSK, Gerbil and

MSPKmerCounter are readily available with histogram program. In this article, the *k*-mer abundance histograms are reported up to a frequency of 10, owing to space limitation, though all the frequency counts were studied and compared. The results are shown in Table A1 for FV dataset and Table A2 for DM dataset as listed in the appendix. The frequency histogram generated by Jellyfish 2.2.6, DSK 2.2.0, kAanalyze 2.0.0, KMC3, Gerbil 1.0, KCMBT 1.0, GenomeTester4 and BFCounter 1.0 were the same for k = 28 and k = 55 for both the datasets, except in case of Gerbil for k = 55, the obtained statistics did not exactly match with the other methods. The frequency counts histogram of MSPKmerCounter is not consistent with the other tools for both datasets and both values of k. The average and maximum error% of MSPKmerCounter 0.1, aTurtle 0.3 and Gerbil 1.0 (only for k = 55 in case of Gerbil) for datasets FV and DM for both values of k (k = 28 and 55) are included in the supplementary document (Table S3 - S6). The results of MSPKmerCounter are found to be very different compared to the results of the other tools for both the datasets.

The results are not entirely wrong for aTurtle, MSPKmerCounter and Gerbil (only for k = 55 in case of Gerbil). For more rigorous analysis, a shell script containing set of Linux utilities i.e. *sort* (to sort output in lexicographic order) and *diff* command (as *diff* analyzes two files and prints the lines that are different) are used to verify results line by line for all the considered tools.

We use DSK output as the reference output for validating the output of aTurtle. This is because DSK and aTurtle considers the same order on bases, i.e. $A < C < T < G$. As seen from Tables S3 – S5 in the supplementary document, there are little differences in frequency count of *k*-mers between the DSK and aTurtle. On comparing, the lexicographically sorted *k*-mers of the two outputs, we found that variations existed (though frequency statistics of aTurtle matched in case of dataset DM for k = 55 with DSK) but with other tools matching results are obtained. There

were unmatched frequency *k*-mers, missing *k*-mers and some additional *k*-mers present in the output of aTurtle as compared to the DSK. The similar type of differences were observed on comparing results of MSPKmerCounter and Gerbil (only for k = 55) with the results of KMC3. Output of these three tools were compared as they consider the same order on bases, i.e. $A < C < G < T$.

From the above results, we conclude that the recent versions of these tools considered in this study i.e. MSPKmerCounter, aTurtle and Gerbil have bugs.

Result and discussion

The *k*-mer counting results for the normalized input and multiple gzip/bz2 inputs in terms of time, memory, disk and % CPU utilization are summarized in Table 4 for dataset FV, for DM in Table 5, for MB in Table 6, for HS1 in Table 7 and HS2 in Table 8. For benchmarking results, we decided to wait for 15 hours for every tool execution, but some tools took longer time for execution and hence some data is missing in respective tables. All tools are executed on initial five datasets for two values of *k*, i.e. 28 and 55. For initial five datasets, bold and * marked entries show the best results and bold italic entries show (including second lowest for %CPU utilization) average results of the tools for all the considered dimensions in Tables 4 to 8. The gzip/bz2 results are not considered while highlighting the best and average results to have a comparison on an equal platform.

For FV and DM datasets, all tools executed completely within 15 hours. For HS1 and HS2 datasets, KCMBT could not finish its execution within 15 hours, and the job had to be killed due to the large size of RAM usage, leaving the system in a frozen state. Jellyfish for HS1 for both *k* values could not complete its execution within15 hours owing to the similar reasons.

We tested Jellyfish with its Bloom filter based mode also to allow it to complete its execution on HS1 dataset. But for both *k* values (*k* = 28 and 55) it could not complete within 15 hours in phase 1 and also because of system freeze, we had to forcefully terminate its execution.

Table 4 Experimental results for FV data set

**SN**	**Tools (Version)**	***k* = 28**				***k* = 55**			
		**Time (sec)**	**RAM (GB)**	**Disk (GB)**	**%CPU Utilization (Comment)**	**Time (sec)**	**RAM (GB)**	**Disk (GB)**	**%CPU Utilization (Comment)**
1	Jellyfish 2.2.6	138.33	7.9	0	1093.55 (Consistent)	226	***36.19***	0	**1050.93*** (Consistent)
2	DSK 2.2.0	56.33	6.35	6	866.50 (Consistent)	78.33	7.04	5	633.49 (Decline from 1174 to 129.7)
3	DSK 2.2.0 (gzip)	197	4	6	402.71 (Initial 80% consistent to 300, last 20% sudden increase to 1200)	222	6	5	441.21 (Initial 75% consistent to 390, last 25% sudden increase to 1200)
4	KAnalyze 2.0.0	***2042***	10	***22.2***	509.20 (Initially in the range of 1000 to 2000 then declined to 200)	***4095***	11	***42***	337.46 (Initially in the range of 1000 to 2000, then declined to 150)
5	KAnalyze 2.0.0 (gzip)	1999	9	22.9	507.84 (Initial 30% inconsistent in range 2250 - 750, last 70% sudden drop to 200)	3395	11	12.8	360.456 (Initial 30% inconsistent in range 2250 - 750 , last 70% sudden drop to 200)
6	KMC3	38.66	7.66	**4***	998.10 (Consistent)	**35***	11.2	4	987.891 (Consistent)
7	KMC3 (gzip)	35	7	2.2	1004.61 (Consistent)	37	11	0	1056.25 (Consistent)
8	Gerbil 1.0	**33.66 ***	**848 MB***	**4***	**1110.38*** (Consistent)	60.33	**1.29***	**3***	1030.50 (Consistent)
9	Gerbil 1.0 (gzip)	49	841 MB	1.5	858.46 (Initial 50% consistent in range 600, last 50% sudden increase to 1200)	55	1	1	880.77 (Initial 50% consistent in range 600, last 50% sudden increase to 1300)
10	KCMBT 1.0	137.5	***30.98***	0	628.87 (Inconsistent)	Not Supported			
11	MSPKmerCounter 0.1	59.33	4.45	1	811.70 (Phase 1 : consistent (200) Phase 2 : consistent (1500))	67.33	4.61	1	770.87 (Phase 1 : consistent (200) Phase 2 : consistent (1500))
12	aTurtle 0.3	671	14	0	***<u>99.14</u>*** (Consistent)	1185	26	0	***<u>94.12</u>*** (Consistent)
13	GenomeTester4	214	26	0	***202.33*** (Consistent)	Not Supported			
14	BFCounter 1.0	1731	3	0	274.10 (Initial 80% almost 100 then gradually increased to 1000)	1790	9	0	***271.80*** (initial 80% in range 100 to 800 then consistent in range 700 – 900)
15	BFCounter 1.0 (gzip)	1847	3	0	259.70 (Inconsistent)	1889	9	0	251.49 (Inconsistent)

Bold and * marked entries indicates best results and bold italic entries (including second lowest for %CPU utilization) show average results excluding gzip results. After validation of results, MSPKmerCounter results are found to be highly varying compared to other tools. Hence its results are not considered here. For column 'Disk' the best (bold *) and average (bold italic) are highlighted considering disked based tools only. Abbreviations: sec = Seconds, GB = Gigabytes, MB = Megabytes.

KAnalyze failed on HS1 and HS2 with 'java.io.IOException: No space left on device' error for both values of *k*. For *k* = 55, aTurtle on dataset MB could not complete within the time limit of 15 hours. aTurtle for both values of *k* on dataset HS1 and HS2 failed with 'std::bad_alloc Aborted (core dumped)' error message due to the large RAM usage. Similarly, for dataset HS1 and HS2, GenomeTester4 and BFCounter could not complete within the 15 hours time limit, and the process was killed due to a system hang. MSPKmerCounter failed to process HS1 dataset in phase 2 with 'OutOfMemoryError' error.

GenomeTester4, KCMBT and aTurtle does not support longer *k* values (Figure 1). Hence, these tools are not considered for benchmarking of longer *k*. BFCounter could not execute on dataset NC and gave an error message 'segmentation fault (core dumped)' whereas Jellyfish and KAanalyze could not finish their execution within 15 hours. BFCounter could not generate results in case of AT dataset with 'segmentation fault (core dumped)' error whereas Jellyfish and KAnalyze could not finish their execution within 15 hours. Hence, we conducted experiments to compare DSK, KMC3 and Gerbil. These tools are highly optimized to support large values of *k*. Figure 1 shows the running time, memory and disk utilization of these tools for *k* values 28, 40, 55, 65, 100, 125, 150, 175 and 200. MSPKmerCounter could not generate results for NC data set but succeeded to generate for AT data set for all values of *k*. Here we haven't considered it for comparison (Figure 1) as their results are highly different compared to the others.

Table 5 Experimental results for DM dataset

**SN**	**Tools (Version)**	***k* = 28**				***k* = 55**			
		**Time (sec)**	**RAM (GB)**	**Disk (GB)**	**%CPU Utilization (Comment)**	**Time (sec)**	**RAM (GB)**	**Disk (GB)**	**%CPU Utilization (Comment)**
1	Jellyfish 2.2.6	77	4	0	1055.25 (Consistent)	71	9	0	917.79 (Consistent)
2	DSK 2.2.0	52	2	4.2	736.09 (Initially 600, increased toward end to 1173)	49	2	2.7	622.36 (Initially 600 then increased to 1150)
3	DSK 2.2.0 (gzip)	183	4.68	3.6	331.88 (Initial 90% consistent to 270, last 10% sudden increase to 1200)	173	4.45	2.4	300.70 (Initial 90% consistent to 270, last 10% sudden increase to 1200)
4	KAnalyze 2.0.0	794	10	***14.3***	695.64 (Gradually declined)	393	11	***12.8***	829.45 (Gradually declined from 2000 to 100)
5	KAnalyze 2.0.0 (gzip)	822	9	14.4	691.15 (initial 40% consistent to 1250, last 60% sudden drop to 200)	411	11	12.9	843.79 (Initial 60% inconsistent in range 2250 - 900, then sudden drop to 200)
6	KMC3	**18***	5	2.23	942.26 (Consistent)	**13***	8	**614.4 MB***	**1023.54*** (Consistent)
7	KMC3 (gzip)	35	5	1.64	739.01 (last 20% consistent to 1250 rest is consistent to 700)	31	8	0	637.29 (last 20% consistent to 1250 rest is consistent nearly to 600)
8	Gerbil 1.0	20	**827 MB***	**2.11***	**1184.23*** (Consistent)	16.5	**826 MB***	4	1010.89 (Consistent)
9	Gerbil 1.0 (gzip)	33	837 MB	1.31	821.69 (initial 55% consistent to 700 , last 45% sudden increase to 1200)	29	834 MB	0	685.74 (Initial 50% consistent to 600, last 45% sudden increase to 1200)
10	KCMBT 1.0	61	2	0	595.37 (Initially 300, increased toward end to 900)	Not Supported			
11	MSPKmerCounter 0.1	234	5	14.2	912.92 (For both phases : Consistent)	219	5	11.2	914.62 (Phase1 : initially 1000 and then declined to 300 Phase2 : Consistent)
12	aTurtle 0.3	423	7	0	***97.20*** (Consistent)	330	12	0	***95.39*** (Consistent)
13	GenomeTester4	144	***23***	0	***183.92*** (Consistent)	Not Supported			
14	BFCounter 1.0	***914***	1	0	307.53 (Consistent)	***477***	2	0	***331.48*** (Initial 95% in range 250-400, then increase to 800)
15	BFCounter 1.0 (gzip)	1002	2	0	321.78 (End 20% 500, rest is 300)	559	2	0	306.30 (End 20% 500, rest is 300)

Bold and * marked entries indicates best results and bold italic entries (including second lowest for %CPU utilization) show average results excluding gzip results. After validation of results, MSPKmerCounter results are found to be highly varying compared to other tools. Hence its results are not considered here. For column 'Disk' the best (bold *) and average (bold italic) are highlighted considering disked based tools only. Abbreviations: sec = Seconds, GB = Gigabytes, MB = Megabytes.

Table 6 Experimental results for MB dataset

SN	Tools (Version)	*k* = 28				*k* = 55			
		**Time (sec)**	**RAM (GB)**	**Disk (GB)**	**%CPU Utilization (Comment)**	**Time (sec)**	**RAM (GB)**	**Disk (GB)**	**%CPU Utilization (Comment)**
1	Jellyfish 2.2.6	**1467***	15	0	**800.13*** (Consistent)	**1440***	***24***	0	**691.65*** (Consistent)
3	DSK 2.2.0	3358	12	59	185.09 (Consistent)	3039	11	45	***208.54*** (Consistent)
4	KAnalyze 2.0.0	***51422***	10	***189***	279.40 (Initially 2000 and then declined to 150)	***45367***	11	***245***	248.04 (Declined from 2000 to 100)
5	KMC3	2019	9	36	216.93 (Initially in the range of 12 to 400, increased toward end to 600)	1804	10	14	211.12 (Initially in the range of 12 to 400, increased toward end to 600)
6	KMC3 (bz2)	3341	11	36.3	289.46 (Initial 90% consistent in range of 200-400, last 10% up to 1300)	3250	11	13	282.77 (Initial 90% consistent in range of 200-400, last 10% up to 1300)
7	Gerbil 1.0	2238	**2***	**32***	269.52 (Initially within 150, increased towards end to 800)	1941	**3***	**11***	250.32 (Initially within 150, increased towards end to 800)
8	Gerbil 1.0 (bz2)	3487	2	30.7	306.37 (Initial 90% consistent in range of 270, last 10% sudden increase to 1300)	3137	3	11	304.02 (Initial 90% consistent in range of 270, last 10% sudden increase to 1300)
9	KCMBT 1.0	1644	34	0	***135.87*** (Consistent)	Not Supported			
10	MSPKmerCounter 0.1	11094	8	173	316.90 (Consistent)	8759	9	118	1284.05 (Consistent)
11	aTurtle 0.3	8764	***61***	0	***75.07*** (Consistent)	>15 Hours			
12	GenomeTester 4	3520	60	0	153.67 (Consistent)	Not Supported			
13	BFCounter 1.0	18950	10	0	300.37 (Consistent)	15264	19	0	295.40 (Initial 50% up to 254 then increased to 434)

Few programs failed to process the dataset within 15 hours, or due to insufficient RAM/Disk space, corresponding entries are denoted by respective mentioned failure message. Bold and * marked entries indicates best results and bold italic entries (including second lowest for %CPU utilization) show average results excluding gzip results. After validation of results, MSPKmerCounter results are found to have high variance compared to other tools. Hence its results are not considered here. For column 'Disk' the best (bold *) and average (bold italic) are highlighted considering disked based tools only. Abbreviations: sec = Seconds, GB = Gigabytes, MB = Megabytes.

Only three programs KMC3, DSK and Gerbil succeeded in generating results for all values of *k* ranging from 28 to 200 on both the data sets NC and AT in stipulated time (Figure 1). Moreover, again Gerbil was consistently the most memory frugal, but as *k* value increased Gerbil, DSK and KMC3 had the same performance in most of the cases which can be seen from Figure 1. KMC3 is faster as compared to Gerbil but when *k* reaches to a higher value, for example from *k* = 150 and 200, both the tools required the same amount of time for dataset NC. In case of dataset AT, KMC3 was faster as compared to DSK and Gerbil. DSK has the same

memory requirement as that of KMC3 and Gerbil, but it is slower.

In the following section, we discuss the results of all datasets separately based on every parameter considered. Among all the tools under comparison and underlying hardware, only DSK and KMC3 could generate accurate results (Appendix - Table A1 and Table A2, accuracy checked for FV and DM datasets only) for both values of *k* within the stipulated time, without any system hang for all the datasets.

Table 7 Experimental results for HS1 dataset

SN	Tools (Version)	*k* = 28				*k* = 55			
		Time (sec)	RAM (GB)	Disk (GB)	%CPU Utilization (Comment)	Time (sec)	RAM (GB)	Disk (GB)	%CPU Utilization (Comment)
1	Jellyfish 2.2.6	>15 Hours (system hang)				>15 Hours (system hang)			
2	DSK 2.2.0	***7722***	***12***	***133***	***210.2*** **(**Inconsistent)	***9389***	***14***	***48***	***255.862*** **(**Inconsistent)
3	DSK 2.2.0 (gzip)	9240	11	134	218.77 (Initial 55% consistent to 300, last 45% sudden increase to 650)	8480	12	104	284.68 (Initial 60% up to 400 then sudden increased to 800 for the rest)
4	KAnalyze 2.0.0	Failed, Error: IO error writing segment file: No space left on device				Failed, Error: IO error writing segment file: No space left on device			
5	KAnalyze 2.0.0 (gzip)	Failed, Error: IO error writing segment file: No space left on device				Failed, Error: IO error writing segment file: No space left on device			
6	KMC3	**3725***	10	78	276.64 (Gradually declined)	**3466***	**11***	28	270.55 (Inconsistent)
7	KMC3 (gzip)	1964	11	79	620.84 (Inconsistent)	1626	11	29	663.31 (Inconsistent)
8	Gerbil 1.0	4078	**6***	**66***	**370.77*** (Initially within 200, increased towards end to 1200)	3818	**11***	**21***	**320.21*** (Inconsistent)
9	Gerbil 1.0 (gzip)	2849	6	66	569.83 (Initial 70% up to 620 then sudden increased to 1300 for the rest)	2614	11	22	541.63 (Initial 70% up to 600 then sudden increased to 1300 for the rest)
10	KCMBT 1.0	>23 Hour				Not Supported			
11	MSPKmerCounter 0.1	>15 Hours (Phase 2 failed , OutOfMemoryError)				>15 Hours (Phase 2 failed , OutOfMemoryError)			
12	aTurtle 0.3	Aborted (core dumped)				Aborted (core dumped)			
13	GenomeTester4	>15 Hours				Not Supported			
14	BFCounter 1.0	>15 Hours				>15 Hours			
15	BFCounter 1.0 (gzip)	>15 Hours				>15 Hours			

Few programs failed to process the dataset within 15 hours, or due to insufficient RAM/Disk space, corresponding entries are denoted by respective mentioned failure message. Bold and * marked entries indicates best results and bold italic entries (including second lowest for %CPU utilization) show average results excluding gzip results. After validation of results, MSPKmerCounter results are found to have high variance compared to other tools. Hence its results are not considered here. For column 'Disk' the best (bold *) and average (bold italic) are highlighted considering disked based tools only. Abbreviations: sec = Seconds, GB = Gigabytes, MB = Megabytes.

Table 8 Experimental results for HS 2 dataset

SN	Tools (Version)	*k* = 28				*k* = 55			
		**Time (sec)**	**RAM (GB)**	**Disk (GB)**	**%CPU Utilization (Comment)**	**Time (sec)**	**RAM (GB)**	**Disk (GB)**	**%CPU Utilization (Comment)**
1	Jellyfish 2.2.6	**3310***	***58***	0	**1000.29*** **(**Consistent)	***11126***	***48***	0	**376.578*** (Declined from 1000 to 100)
2	DSK 2.2.0	***8879***	13	***145***	***186.66*** (Consistent)	7982	13	***109***	***211.54*** **(**Consistent)
3	DSK 2.2.0 (gzip)	10360	10	146	242.01 (Inconsistent)	10199	12 GB	109	240.21 (Initial 60% consistent to 300 , last 40% sudden increase to 650)
4	KAnalyze 2.0.0	Failed, Error: IO error writing segment file: No space left on device				Failed, Error: IO error writing segment file: No space left on device			
5	KAnalyze 2.0.0 (gzip)	Failed, Error: IO error writing segment file: No space left on device				Failed, Error: IO error writing segment file: No space left on device			
6	KMC3	4252	10	85	218.02 (Increased toward end to 600, otherwise it is up to 12)	**3846***	11	29	214.99 (Increased toward end to 600, otherwise it is up to 12)
7	KMC3 (gzip)	2362	10	86	580.72 (Inconsistent)	1995	11	29	556.31 (Inconsistent)
8	Gerbil 1.0	4553	**5***	**74***	371.26 (Increased toward end to 1000, otherwise it is up to 250)	4260	**9***	**23***	317.65 (Initially 250, increased towards end to 1000)
9	Gerbil 1.0 (gzip)	3358	5	74	553.59 (Initial 70% up to 600 then sudden increased to 1300 for the rest)	3121	9	23	507.19 (Initial 70% up to 550 then suddenly increased to 1300 for the rest)
10	KCMBT 1.0	>15 Hours				Not Supported			
11	MSPKmerCounter0.1	3128	6	22.2	120.17 (Consistent)	3124	9	5.7	340.49 (Consistent)
12	aTurtle 0.3	Aborted (core dumped)				Aborted (core dumped)			
13	GenomeTester4	>15 Hours				Not Supported			
14	BFCounter 1.0	>15 Hours				>15 Hours			
15	BFCounter 1.0 (gzip)	>15 Hours				>15 Hours			

Few programs failed to process the dataset within 15 hours, or due to insufficient RAM/Disk space, corresponding entries are denoted by respective mentioned failure message. Bold and * marked entries indicates best results and bold italic entries (including second lowest for %CPU utilization) show average results excluding gzip results. After validation of results, MSPKmerCounter results are found to have high variance compared to other tools. Hence its results are not considered here. For column 'Disk' the best (bold *) and average (bold italic) are highlighted considering disked based tools only. Abbreviations: sec = Seconds, GB = Gigabytes, MB = Megabytes.

KMC3 often comes on the top for its running time (Table 9, Figure 1), but it is not that memory frugal as compared to its top competitor Gerbil in this regard, but often not far from the best in case of disk utilization. DSK consistently uses a moderate amount of memory with reasonable speed for the whole range of datasets. It is also robust as it pass all the tests.

Table 9 Summary Table

Data-set ID	*k*-length	Time		RAM		Disk		%CPU	
		**Highest**	**Lowest**	**Highest**	**Lowest**	**Highest**	**Lowest**	**Highest**	**Lowest**
FV	28	kAanalyze	Gerbil	KCMBT	Gerbil	kAanalyze	Gerbil , KMC3	Gerbil	GenomeTester4, aTurtle
	55	kAanalyze	KMC3	Jellyfish	Gerbil	kAanalyze	Gerbil	Jellyfish	BFCounter, aTurtle
DM	28	BFCounter	KMC3	GenomeTester4	Gerbil	kAanalyze	Gerbil	Gerbil	GenomeTester4, aTurtle
	55	BFCounter	KMC3	aTurtle	Gerbil	kAanalyze	KMC3	KMC3	BFCounter, aTurtle
MB	28	kAanalyze	Jellyfish	aTurtle	Gerbil	kAanalyze	Gerbil	Jellyfish	KCMBT, aTurtle
	55	kAanalyze	Jellyfish	Jellyfish	Gerbil	kAanalyze	Gerbil	Jellyfish	DSK
HS1	28	DSK	KMC3	DSK	Gerbil	DSK	Gerbil	Gerbil	DSK
	55	DSK	KMC3	DSK	Gerbil, KMC3	DSK	Gerbil	Gerbil	DSK
HS2	28	DSK	Jellyfish	Jellyfish	Gerbil	DSK	Gerbil	Jellyfish	DSK
	55	Jellyfish	KMC3	Jellyfish	Gerbil	DSK	Gerbil	Jellyfish	DSK

In case of lowest %CPU utilization, we have also mentioned the second lowest entries, as aTurtle is single threaded hence its %CPU utilization is by default lowest

Table 9 reflects the summary results of the tools, excluding the compressed input statistics, as only five tools support compressed input format. Interestingly, Gerbil is consistently the most memory and disk frugal superior to KMC3 (Table 9). Even for the most of the datasets, Gerbil's disk utilization is the lowest among all the tools. When it's time is compared to the time of KMC3, it is still remarkable (here we did not have a benchmark for GPU implementation of Gerbil) (Table 9). Gerbil tries to reduce the utilization of disk and memory, revealing an astonishing result - memory and disk economical which can process massive size of input data like human irrespective of underlying hardware configuration. Their results are obtained due to the approach of efficient data partitioning (siganture) similar to KMC3 approach. Moreover, it uses a hashing approach to count *k*-mer with an additional step to handle weak *k*-mers and its dynamic method to predict hash table size that adds vital costs to the memory and disk usages. Low memory usage by Gerbil leaves the buffer space in memory for disk operation which effectively reduces the overall cost of expensive I/O operation. Due to lower I/O activity it has the high % CPU utilization.

Hash table based counting is more hardware frugal as compared to sorting approach. But KMC3 is faster compared to Gerbil, because it uses an efficient approach of sorting, along with other effective schemes like parallelization and input file reading.

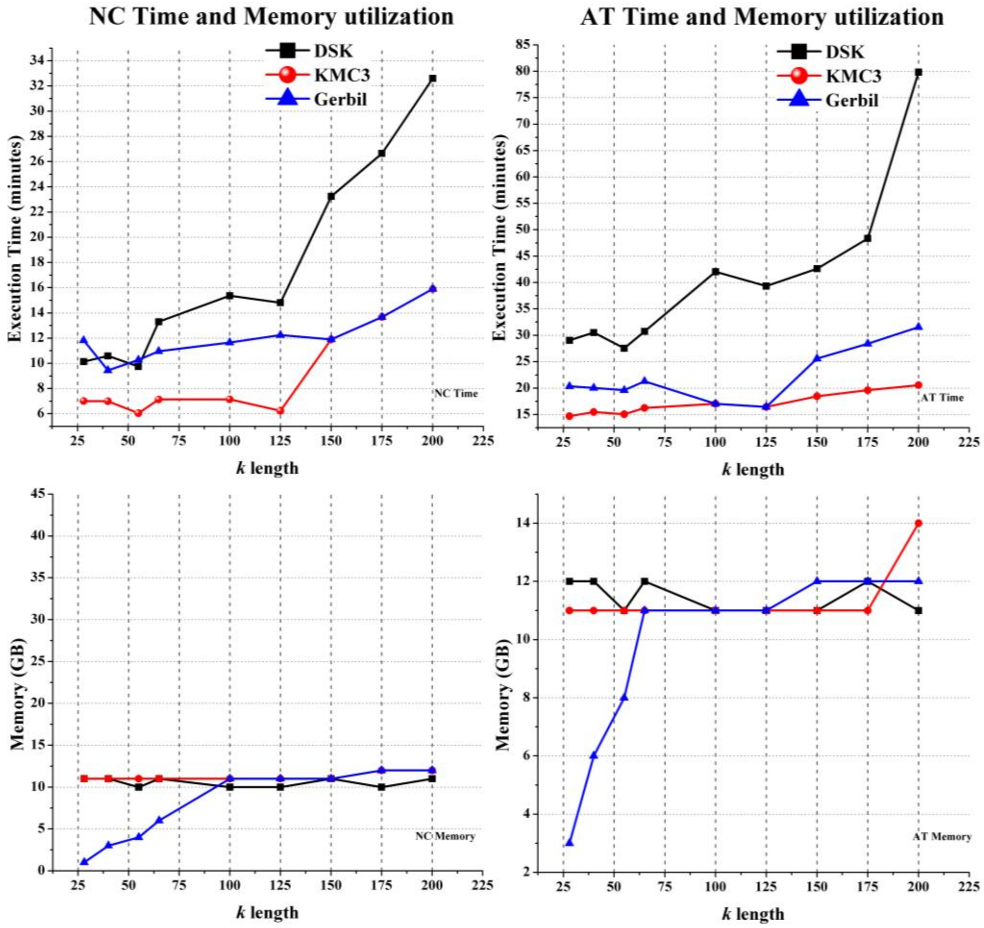

Figure 1. Analysis of time (minutes) and memory (GB) utilization of counting algorithms on AT and GT datasets for longer *k* length with, *k* = 28, 40, 55, 65, 100, 125, 150, 175 and 200

MSP gives astonishing compression ratio which effectively reduces I/O cost and ultimately reduces overall time and space requirement as I/O operation is much more expensive compared to the counting operation. The recent tools which implement this strategy, with balance in the size of bins are KMC3 and Gerbil, and these therefore outperform the rest of the tools. KAnalyze which is a disk-based *k*-mer counter has much higher run-time and disk usage for the most of the datasets for both values of *k* compared to the other disk based approaches (Table 9). Its

performance is significantly worse compared to GenomeTester4 which is an in-memory approach. It is observed that KAanalyze 2.0.0 needs more time for its merging step, as its partitioning step is relatively straightforward.

For in-memory approaches like Jellyfish, aTurtle, KCMBT, GenomeTester4 and BFCounter, only KCMBT and GenomeTester4 do not support higher values of *k*. In-memory approaches need no extra disk space as these are completely memory-based wherein, the entire dataset needs to be in the memory. Amongst all the in-memory approach based tools, BFCounter utilizes the lowest RAM. This is because of the Bloom filter data structure which is highly memory efficient. For data set MB, Jellyfish is the fastest, with highest CPU utilization, but for HS1 it did not finish its execution within 15 hours. Jellyfish executed with comparable time and memory requirements and finished its execution within 15 hours of stipulated time where it was possible.

For small read length dataset, i.e. DM (Table 9), the execution is faster for higher *k* in case of most of the tools. For low machine configuration and bigger size data sets like human dataset, Gerbil, KMC3 and DSK generate outputs within a reasonable time. For both the human datasets, i.e. HS1 and HS2, only Gerbil, KMC3 and DSK could finish within a reasonable time and without any system hang. Gerbil and Jellyfish often have the highest % CPU utilization. For bigger datasets with size > 200 GB, only the disk-based approaches performed well on our machine configuration. The tools using disk-based approach are more efficient in terms of memory utilization and scalability for bigger size datasets and larger value of *k* as compared to tools based on in-memory approach. Perez et al. [25] have reported similar behavior of various *k*-mer counting tools for the parameters time, memory utilization and parallelization.

Currently, DSK, Gerbil and KMC3 are the only programs that handle large *k* values. KMC3 and Gerbil provide better scalability (minimum time required) to larger *k* values for the dataset

with long reads (Figure 1).

We have also tested the impact of input format gzip and bzip2 on the performance of various tools. As the sequencing (genomic) data is very large in size, it is generally stored in a compressed format mostly in gzip. The compressed FASTQ/FASTA files can be directly processed by some tools. This has several advantages like the I/O throughput is improved if hard-drives are used and in this way the data is consumed faster by the algorithms. The data throughput is increased due to the compressed data being directly read from the disk thereby overcoming the cost of decompression of the file in the memory. Only five tools currently support compress file processing namely, KMC3, Gerbil, DSK, KAnalyze and BFCounter. Processing gzip format reduces the time (more visible results in case of large datasets like HS1 and HS2, except in case of dataset DM) which can be seen from Tables 4 - 8. Among the tools supporting compressed input, KMC3 outperforms in terms of time. The gzip time for KMC3 and Gerbil is much less than their normalized input time for datasets HS1 and HS2, whereas the performance of DSK is degraded. KMC3 is the fastest of all the tools running on normalized and compressed input whereas Gerbil is consistent in using the lowest memory and disk irrespective of input types (compressed/normalized). Bz2 has a high compression ratio but decompression is very slow thus, processing a bz2 input is more costly than gzip input. The only tools that can process bz2 are KMC3 and Gerbil.

To assess the CPU-related performance, we tested the performance of all tools (except for aTurtle as it is single threaded) for varying number of threads = 1, 2, 4, 6, 8 and 12 on our machine (Figure 2). Figure 2 demonstrates the information related to the scaling properties of various tools corresponding to the number of threads used. Two datasets, FV and MB are considered for this study for k = 28, such that all tools can complete their execution in specified

time. It is not possible to achieve linear speedup for any tool. Jellyfish (in-memory approach), implemented using a multithreaded lock-free hash table, outperforms (highest speedup, i.e. 8.3 and 6.7 for dataset FV and MB respectively for threads = 12) amongst all the tools for multithreaded execution. However, disk-based tools like DSK and KMC3 also achieved better speedup in case of dataset FV (7.2 and 7.1 for DSK and KMC3 respectively for threads = 12).

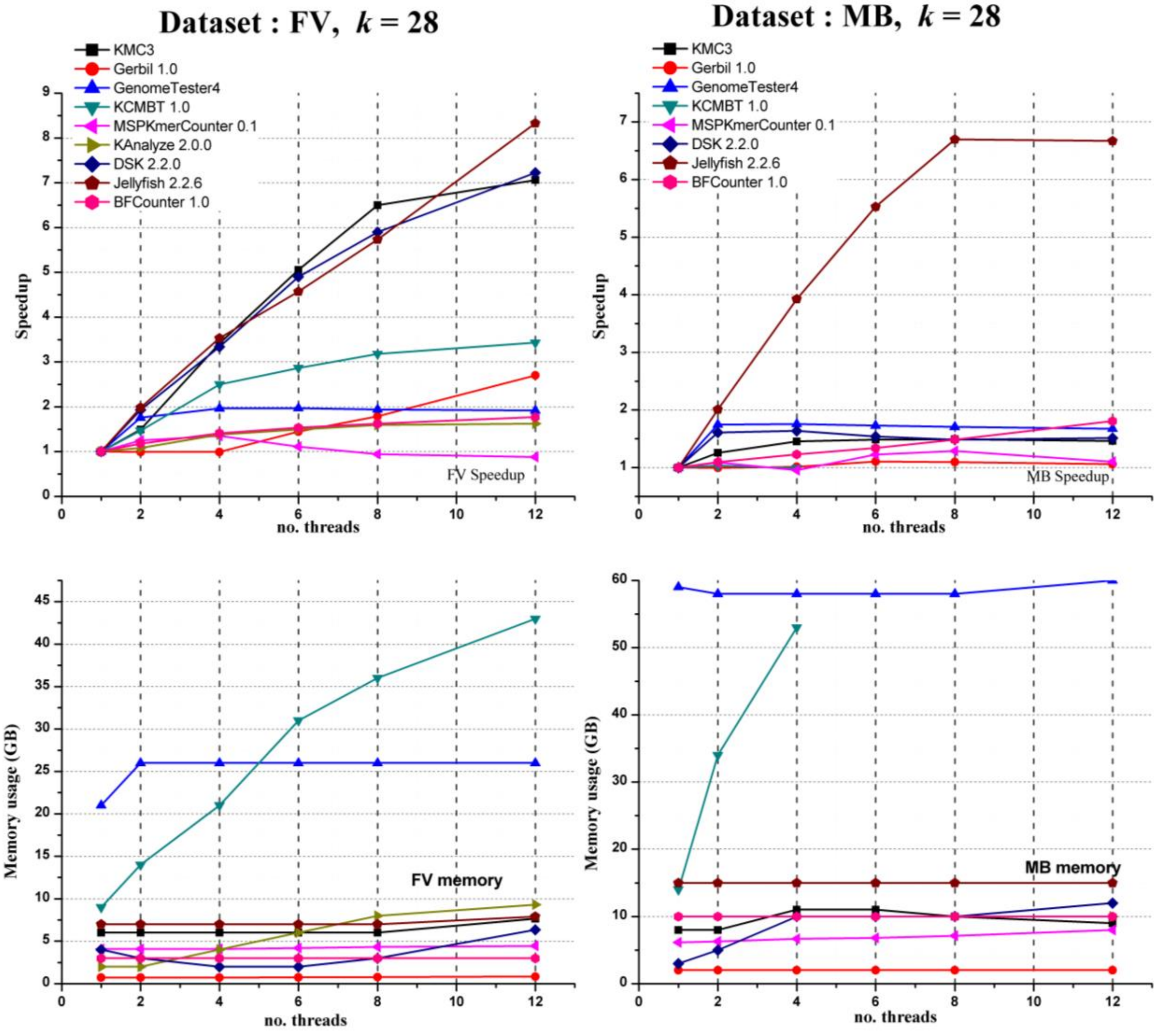

Figure 2. Comparison of scalability of different *k*-mer counting tools on the number of threads

But for larger dataset MB, the speedup achieved by all tools is low and is in the range 1 - 2 (except for Jellyfish). This is due to the increased threading overhead as resource demand is increased by each thread owing to the large input size but having only limited underlying

resources. For MB dataset, KCMBT is fastest for a single thread among all the tools (Table S8 supplementary document). But for thread = 4 it did not finish its execution even in 15 hours because of its huge memory usages which keep on increasing with the increase in the number of threads. However, in case of the other tools, the memory requirement is almost constant for an increasing number of threads.

Conclusions and future directions

There is a wide range of applications for *k*-mer counting used for solving numerous problems in bioinformatics. With the advancement in the high throughput sequencing technologies, several gigabytes of genomic data is generated. There is a continued need to develop a system that offers a time and memory efficient solution for the task of *k*-mer counting to process such gigabytes of data. Here we intend to provide the reader with a reasonable outline of all the latest tools for *k*-mer counting. This survey can be concluded with the following remarks.

Many disk-based and in-memory approaches are available for *k*-mer counting that aimed to generate results in minimum time on large genomic data on a personal computer having limited resources.

Of all the tools considered herein; KMC3, DSK and Gerbil are the most flexible and efficient programs as they have higher speed, ability to scale with larger datasets and robust with respect to the automatic parameter selection. They support wide range of *k* (large *k* is an important use case for longer reads) and can read gzipped FASTQ which tends to improve overall processing time. These *k*-mer counting tools optimized further to gain a significant speedup by implementing parallelization using the available cores in the machine. As *k*-mer counting is fundamentally an I/O intensive process the overall speedup obtained by the tools is not very high.

As sequencing technologies keep on evolving, *k*-mer counting algorithms should improve performance. Research endeavours must keep improving to establish a better system that influences the *k*-mer counting process with respect to change in size of the data.

References

1. Reuter JA, Spacek DV, Snyder MP. High-Throughput Sequencing Technologies. Mol. Cell. 2015;58(4):586-97. doi: 10.1016/j.molcel.2015.05.004
2. Molnar M, Ilie L. Correcting Illumina data. 2014;16:588–99. https://doi.org/10.1093/bib/bbu029
3. Miller JR, Delcher AL, Koren S, Venter E, Walenz BP, Brownley A, et al. Aggressive assembly of pyrosequencing reads with mates. Bioinformatics. 2008;24:2818–24. doi: 10.1093/bioinformatics/btn548
4. Jaffe DB, Butler J, Gnerre S, Mauceli E, Lindblad-toh K, Mesirov JP, et al. Whole-Genome Sequence Assembly for Mammalian Genomes : Arachne 2. 2003;13(1):91–6. doi: 10.1101/gr.828403
5. Miller JR, Koren S, Sutton G. Assembly algorithm for next-generation sequencing data. Genomics. 2010;95(6):315–27. doi: 10.1016/j.ygeno.2010.03.001
6. Pevzner PA, Tang H, Waterman MS. An Eulerian path approach to DNA fragment assembly. Proc. Natl. Acad. Sci. USA. 2001;98(17):9748–53. doi: 10.1073/pnas.171285098
7. Zerbino DR, Birney E. Velvet: Algorithms for de novo short read assembly using de Bruijn graphs. Genome Res. 2008;18(5):821–9. doi: 10.1101/gr.074492.107
8. Simpson JT, Wong K, Jackman SD, et al. ABySS : A parallel assembler for short read sequence data. 2009;19(6):1117–23. doi: 10.1101/gr.089532.108.
9. Newburger DE, Bulyk ML. UniPROBE: An online database of protein binding microarray data on protein-DNA interactions. Nucleic Acids Res. 2009;37:D77–82. doi: 10.1093/nar/gkn660
10. Kelley DR, Schatz MC, Salzberg SL. Quake: quality-aware detection and correction of sequencing errors. Genome Biol. 2010;11:R116. https://doi.org/10.1186/gb-2010-11-11-r116
11. Shi H, Schmidt B, Liu W, Muller-Witting W. A parallel algorithm for error correction in high-throughput short-read data on CUDA-enabled graphics hardware. J Comput Biol. 2010;17(4):603–15. doi: 10.1089/cmb.2009.0062
12. Liu Y, Schröder J, Schmidt B. Musket: A multistage k-mer spectrum-based error corrector for Illumina sequence data. Bioinformatics. 2013;29(3):308–15. doi: 10.1093/bioinformatics/bts690
13. Medvedev P, Scott E, Kakaradov B, Pevzner P. Error correction of high-throughput sequencing datasets with non-uniform coverage. 2011;27(13):i137–41. doi: 10.1093/bioinformatics/btr208
14. Salmela L, Schröder J. Correcting errors in short reads by multiple alignments. 2011;27(11):1455–61. https://doi.org/10.1093/bioinformatics/btr170
15. Edgar RC. MUSCLE: Multiple sequence alignment with high accuracy and high throughput. Nucleic Acids Res. 2004;32(5):1792–7. DOI: 10.1093/nar/gkh340
16. Liu B, Shi Y, Yuan J, Hu X, Zhang H, Li N, et al. Estimation of genomic characteristics by analyzing k-mer frequency in de novo genome projects. 2013; http://arxiv.org/abs/1308.2012
17. Li R, Ye J, Li S, Wang J, Han Y, Ye C, et al. ReAS: Recovery of ancestral sequences for transposable elements from the unassembled reads of a whole genome shotgun. PLoS Comput. Biol. 2005;1:0313–21. https://doi.org/10.1371/journal.pcbi.0010043
18. Price AL, Jones NC, Pevzner PA. De novo identification of repeat families in large genomes. Bioinformatics. 2005;21:i351–8. DOI: 10.1093/bioinformatics/bti1018
19. Campagna D, Romualdi C, Vitulo N, Del Favero M, Lexa M, Cannata N, et al. RAP: A new computer program for de novo identification of repeated sequences in whole genomes. Bioinformatics. 2005;21:582–8. DOI: 10.1093/bioinformatics/bti039
20. Lefebvre A, Lecroq T, Dauchel H, Alexandre J. FORRepeats: Detects repeats on entire chromosomes and between genomes. Bioinformatics. 2003;19:319–26.
21. Healy J, Thomas EE, Schwartz JT, Wigler M. Annotating large genomes with exact word matches. Genome Res. 2003;13:2306–15. doi: 10.1101/gr.1350803

22. Kurtz S, Narechania A, Stein JC, Ware D. A new method to compute K-mer frequencies and its application to annotate large repetitive plant genomes. BMC Genomics. 2008;9:517. https://doi.org/10.1186/1471-2164-9-517
23. Sindi SS, Hunt BR, Yorke JA. Duplication count distributions in DNA sequences. Phys. Rev. E - Stat. Nonlinear, Soft Matter Phys. 2008;78. DOI: 10.1103/PhysRevE.78.061912
24. Pajuste F-D, Kaplinski L, Möls M, Puurand T, Lepamets M, Remm M. FastGT: from raw sequence reads to 30 million genotypes in less than an hour. doi: https://doi.org/10.1101/060822
25. Pérez N, Gutierrez M, Vera N. Computational performance assessment of k-mer counting algorithms. J Comput Biol. 2016;23(4):248–55. doi: 10.1089/cmb.2015.0199
26. Roberts RJ, Carneiro MO, Schatz MC. The advantages of SMRT sequencing. Genome Biol. 2013; 14(6):6–9. https://doi.org/10.1186/gb-2013-14-7-405
27. Laehnemann D, Borkhardt A, McHardy AC. Denoising DNA deep sequencing data-high-throughput sequencing errors and their correction. Brief. Bioinform. 2016;17:154–79. doi: 10.1093/bib/bbv029
28. Sameith K, Roscito JG, Hiller M. Iterative error correction of long sequencing reads maximizes accuracy and improves contig assembly. Brief. Bioinform. 2017;18:1–8. doi: 10.1093/bib/bbw003
29. Xavier BB, Sabirova J, Pieter M, Hernalsteens JP, De Greve H, Goossens H, et al. Employing whole genome mapping for optimal de novo assembly of bacterial genomes. BMC Res. Notes. 2014;7:484. https://doi.org/10.1186/1756-0500-7-484
30. Chikhi R, Medvedev P. Informed and automated k-mer size selection for genome assembly. Bioinformatics. 2014;30:31–7. https://doi.org/10.1093/bioinformatics/btt310
31. Erbert M, Rechner S, Muller-Hannemann M. Gerbil: A fast and memory-efficient k-mer counter with GPU-support. Algorithms Mol. Biol. 2017;**12**:9 https://doi.org/10.1186/s13015-017-0097-9
32. Li Y, XifengYan. MSPKmerCounter: A Fast and Memory Efficient Approach for K-mer Counting. ArXiv e-prints. 2015;1–7. http://arxiv.org/abs/1505.06550
33. Rizk G, Lavenier D, Chikhi R. DSK: K-mer counting with very low memory usage. Bioinformatics. 2013;29:652–3. doi: 10.1093/bioinformatics/btt020
34. Pandey P, Bender MA, Johnson R, Patro R. Squeakr: An Exact and Approximate k-mer Counting System. Bioinformatics. 2017;1–7. doi: https://doi.org/10.1101/122077
35. Marçais G, Kingsford C. A fast, lock-free approach for efficient parallel counting of occurrences of k -mers. Bioinformatics 2011;27(6):764–70. https://doi.org/10.1093/bioinformatics/btr011
36. Melsted P, Pritchard JK. Efficient counting of k-mers in DNA sequences using a bloom filter. BMC Bioinformatics. 2011;12:333. https://doi.org/10.1186/1471-2105-12-333
37. Kokot M, Długosz M, Deorowicz S. KMC 3: counting and manipulating k-mer statistics. Bioinformatics. 2017;2:1–3. http://arxiv.org/abs/1701.08022
38. Kaplinski L, Lepamets M, Remm M. GenomeTester4: a toolkit for performing basic set operations - union, intersection and complement on k-mer lists. GigaScience. 2015;4:58. https://doi.org/10.1186/s13742-015-0097-y
39. Deorowicz S, Kokot M, Grabowski S, Debudaj-grabysz A. KMC 2: fast and resource-frugal k-mer counting. Bioinformatics. 2015; 31(10):1569–76. doi: 10.1093/bioinformatics/btv022
40. Audano P, Vannberg F. KAnalyze: A fast versatile pipelined K-mer toolkit. Bioinformatics. 2014;30:2070–2. doi: 10.1093/bioinformatics/btu152
41. Deorowicz S, Debudaj-grabysz A, Grabowski S. Disk-based k-mer counting on a PC. BMC Bioinformatics. 2013;14:160 https://doi.org/10.1186/1471-2105-14-160
42. Roy RS, Bhattacharya D, Schliep A. Turtle : Identifying frequent k -mers with cache-efficient algorithms. Bioinformatics. 2014;**14**(30):1950–7 doi:10.1093/bioinformatics/btu132
43. Mamun A, Pal S, Rajasekaran S. Sequence analysis KCMBT : a k -mer Counter based on Multiple Burst Trees. 2016;32:2783–90. https://doi.org/10.1093/bioinformatics/btw345
44. Cormen T H, Leiserson C E RRL and SC. Chapter 11: Hash Tables. Introd. to Algorithms. 2nd ed. MIT Press and McGraw-Hill; 2001. p. 221–45. Available from: http://is.ptithcm.edu.vn/~tdhuy/Programming/Introduction.to.Algorithms.pdf
45. Purcell C, Harris T. Non-blocking hashtables with open addressing. Technical Report 639, University of Cambridge, Cambridge, UK. 2005;3724 LNCS:108–21.
46. Gao H, Groote J, Hesselink W. Almost wait-free resizable hashtables. Parallel and Distributed Processing Symposium. 2004;0:1–37. DOI: 10.1109/IPDPS.2004.1302969

47. Shalev O, Shavit N. Split-ordered lists: Lock-free extensible hash tables. J. ACM (JACM). 2006;53:379–405. doi: 10.1145/1147954.1147958

48. Mapleson D, Accinelli GG, Kettleborough G, Wright J, Clavijo BJ, Marc J. KAT : A K-mer Analysis Toolkit to quality control NGS datasets and genome assemblies. Bioinformatics. 2017;33(4):574–6. https://doi.org/10.1093/bioinformatics/btw663

49. Bloom BH. Space/Time Trade-offs in Hash Coding with Allowable Errors. 1970;13(7):422–6 . doi: 10.1145/362686.362692

50. Randall D, Guerrieri A, Jin W. Bloom Filters and Hashing. CS 6550 Design and Analysis of Algorithms. 2006;1–7.

51. Putze F, Sanders P, Singler J. Cache-, hash-, and space-efficient bloom filters. J. Exp. Algorithmics. 2009;14:4.4. doi: 10.1145/1498698.1594230

52. Salomon D. Data compression: the complete reference. Springer Science & Business Media; 2004.https://books.google.co.in/books?hl=en&lr=&id=PT1fcX321I4C&oi=fnd&pg=PR7&dq=%5B55%5D+Salomon+D+2004+Data+compression:+the+complete+reference.+Springer+Science+%26+Business+Media&ots=5iX8lXF0qJ&sig=_GM_InxCPFWXnbN3BZI_mACj8_k#v=onepage&q&f=false

53. Pandey P, Bender MA, Johnson R, Patro R. A General-Purpose Counting Filter. Proc. 2017 ACM Int. Conf. Manag. Data - SIGMOD '17. 2017;775–87. doi: 10.1145/3035918.3035963

54. Abouelhoda MI, Kurtz S, Ohlebusch E. Replacing suffix trees with enhanced suffix arrays. J. Discret. Algorithms. 2004;2:53–86. https://doi.org/10.1016/S1570-8667(03)00065-0

55. Heinz S, Zobel J, Williams HE. Burst tries: a fast, efficient data structure for string keys. ACM Trans. Inf. Syst. 2002;20:192–223. doi: 10.1145/506309.506312

56. Li Y, Kamousi P, Han F, Yang S, Yan X, Suri S. Memory Efficient Minimum Substring Partitioning. 39th Int. Conf. Very Large Data Bases. 2013;6:169–80. doi: 10.14778/2535569.2448951

57. Kokot M, Deorowicz S, Debudaj-Grabysz A. Sorting Data on Ultra-Large Scale with RADULS. New Incarnation of Radix Sort. 2016; http://arxiv.org/abs/1612.02557

58. Melsted P, Halldórsson B V. KmerStream: Streaming algorithms for k-mer abundance estimation. Bioinformatics. 2014;30:3541–7. DOI: 10.1093/bioinformatics/btu713

59. Mohamadi H, Khan H, Birol I. ntCard: A streaming algorithm for cardinality estimation in genomics data. Bioinformatics. 2017;33:1324–30. doi: 10.1093/bioinformatics/btw832

60. Jr LCI, Brown CT. Efficient cardinality estimation for k-mers in large DNA sequencing data sets. 2016;1–5. doi: https://doi.org/10.1101/056846

61. Crusoe MR, Alameldin HF, Awad S, Boucher E, Caldwell A, Cartwright R, et al. The khmer software package: enabling efficient nucleotide sequence analysis. F1000Research. 2015;4:900. doi: 10.12688/f1000research.6924.1

Click here to download Table Appendix.docx

Table A1 Frequency count statistics for FV dataset

***k*-mer freq.**	**28-mers frequency abundance**			**55-mers frequency abundance**			
	**Jellyfish 2.2.6, DSK 2.2.0, kAanalyze 2.0.0, KMC3, Gerbil 1.0, KCMBT 1.0, GenomeTester4, BFCounter 1.0**	**MSPKmerCounter 0.1**	**aTurtle 0.3**	**Jellyfish 2.2.6, DSK 2.2.0, kAanalyze 2.0.0, KMC3, BFCounter 1.0**	**Gerbil 1.0**	**MSPKmer Counter 0.1**	**aTurtle 0.3**
1	363420998	**69066437 ***	**363421125***	652176330	**79102 ***	**39121998 ***	**652176422***
2	49080695	**18115152 ***	**49080694***	76579833	**35492642 ***	**7250617 ***	**76579834***
3	18049815	**5639542 ***	**18049820***	28281157	**13566991 ***	**2084150 ***	**28281161***
4	11150813	**2208574 ***	**11150811***	18211721	**12986541 ***	**832867 ***	**18211720***
5	9843691	**1091696 ***	**9843693***	16401875	**8473838 ***	**414097 ***	**16401874***
6	10378494	**629357 ***	**10378494***	16723214	**8615812 ***	**227106 ***	**16723216***
7	11430920	**390902 ***	**11430919***	17266403	**8908818 ***	**131829 ***	**17266403***
8	12495242	**259176 ***	**12495241***	17420694	**12170688 ***	**82141 ***	**17420693***
9	13289486	**185636 ***	**13289487***	16983119	**8661491 ***	**52736 ***	**16983119***
10	13661314	**144101 ***	**13661314***	16005955	**8144646 ***	**37158 ***	**16005956***

Bold and * marked entries indicate varying results. Abbreviation: freq. = frequency. KCMBT 1.0 and GenomeTester 4 do not support higher *k* value. BFCounter 1.0 does not provide the count for one frequency *k*-mers.

Table A2 Frequency count statistics for DM dataset

***k*-mer freq.**	**28-mers frequency abundance**			**55-mers frequency abundance**		
	**Jellyfish 2.2.6, DSK 2.2.0, kAanalyze 2.0.0, KMC3 Gerbil 1.0, KCMBT 1.0, GenomeTester4, BFCounter 1.0**	**MSPKmerCounter 0.1**	**aTurtle 0.3**	**Jellyfish 2.2.6, DSK 2.2.0, kAanalyze 2.0.0, KMC3, BFCounter 1.0 aTurtle0.3**	**Gerbil 1.0**	**MSPKmerCounter 0.1**
1	155281400	**146698167 ***	**155281818***	132512502	**45255 ***	**127152484 ***
2	8864191	**8707861 ***	**8864201***	15209916	**2025688 ***	**15386744 ***
3	4905173	**4980239 ***	**4905177***	14350714	**1926881 ***	**14551570 ***
4	4626001	**4790519 ***	**4625998***	14070907	**4197271***	**14200127 ***
5	5117297	**5329251 ***	**5117299***	12981796	**1679797 ***	**13035273 ***
6	5763542	**5981513 ***	**5763541***	11361229	**1458782 ***	**11342261 ***
7	6302198	**6510090 ***	**6302198***	9542344	**1232507 ***	**9474395 ***
8	6658595	**6838360 ***	**6658591***	7807048	**2659122 ***	**7709343 ***
9	6817573	**6963587 ***	**6817577***	6272227	**808026 ***	**6169544 ***
10	6799383	**6918078 ***	6799383	4979037	**634728 ***	**4880327 ***

Bold and * marked entries indicate varying results. Abbreviation: freq. = frequency. KCMBT 1.0 and GenomeTester 4 do not support higher *k* value. BFCounter 1.0 does not provide the count for one frequency *k*-mers.

Information regarding the datasets:

For each dataset as listed in Table 2 of manuscript, genome size information is collected from the ncbi website, i.e. https://www.ncbi.nlm.nih.gov/genome/browse/ and the total number of bases information is collected from the website i.e. https://www.ncbi.nlm.nih.gov/sra/?term=SRX040485. The total number of reads in the given FASTA/FASTQ file is calculated by using the commands ‘grep -c "^>" data_set.fasta’ and ‘ awk '{s++}END{print s/4}' data_set.fastq.’ respectively.

All the dataset downloaded in .sar file format are first converted to fastq/fasta files format using the command ‘./fastq-dump SRR_no.sra --fasta’ and ‘./fastq-dump SRR_no.sra’ and then concatenated into a single file. The SRA toolkit is downloaded from https://trace.ncbi.nlm.nih.gov/Traces/sra/sra.cgi?view=software.

The specific details regarding how each tool was run:

For each dataset, genome size information is collected from the ncbi website, i.e. https://www.ncbi.nlm.nih.gov/genome/browse/ and the total number of bases information is collected from the website i.e. https://www.ncbi.nlm.nih.gov/sra/?term=SRX040485. The total number of reads in the given fasta or fastq file is calculated by using the commands ‘grep -c "^>" data_set.fasta’ and ‘ awk '{s++}END{print s/4}' data_set.fastq.’ respectively.

All tools except BFCounter, Jellyfish, aTurtle and MSPKmerCounter have automated parameter selection for most of the program parameters. An improvement could be achieved by including automated parameter selection in these tools.

Jellyfish require pre-specifying the size of the hash table and their documentation recommends to set its size 10% more than the total estimated unique number of *k*-mers. Hence the sizes were set 10 % more than the estimated values taken from KMC3 for all the datasets. For instance, the number of unique counted *k*-mers by KMC3 for DM is 132108719 hence the size of the hash table was set to 250 M (250000000). In case of BFCounter, the estimated number of distinct *k*-mer (an upper-bound) needs to be specified, and therefore we specified the same. As aTurtle also requires a setting for the expected number of unique *k*-mers (including count of single *k*-mers) to select the size of an array, the same values were set as estimated by KMC3.

For KAnalyze, the parameter ‘threads’, amongst *k*-mer generation step (- *l*) and spilt step (-*d*) are set according to the document specification.

For Gerbil, KMC3 and DSK the max amount of RAM in GB, was set to 12 for all the tests. But even the entire maximum memory allocated to them was not used. KCMBT requires the number of threads to be of the order of power of 2. For our machine configuration, threads = 8 gave the best performance with respect to the memory and time requirement. The average length of reads must be set accurately for MSPKmerCounter as improper lengths might not generate the output. The number of blocks and the minimum substring length are set according to the documentation while executing the MSPKmerCounter. The rest of the parameters for all the tools are set by default as per guidelines provided by the tool.

The counting time has been considered for every tool, excluding the dumping time. Each tool has been executed three times for all the input datasets. The average of all three runs is reported as the actual time taken by each tool. All the tools were executed making sure it considered only the canonical forms of *k*-mers. The *k*-mers occurring less than two times were excluded, because singleton *k*-mers were considered to appear in a read library due to sequencing errors. Thus, the uniformity can be maintained, and the statistics for all the tools can be collected. For all tools by default canonical forms of *k*-mers are considered, except for Jellyfish (*-C*) and KAnalyze 2(*-rcanonical*), the option to use only canonical form is explicitly specified by passing a parameter.

For KAnalyze and aTurtle, the time recorded includes the time required for counting the *k*-mers including one frequency *k*-mers, as the program does not provide an option to exclude the singleton *k*-mers. KAnalyze and aTurtle generate the output in a human-readable text format instead of the encoded format, which is not the case with other tools. And hence, other tools require one more additional step to dump the *k*-mers along with their count into a readable format. In case of MSPKmerCounter, the first phase generates nodes files containg super *k*-mers that are written to the disk in text format rather than the encoded form whereas the output file containing *k*-mers with their counts is generated in an encoded format. The

current version of all the listed tools skips counting of *k*-mers containing ‘*N*’ character, which frequently occurs in NGS data.

For the MSPKmerCounter time is calculated by adding the time required for the first phase and second phase and memory utilization by considering the maximum out of two phases. %CPU utilization is calculated as the average of two phases in case of MSPKmerCounter.

Supplementary Material

Click here to access/download
Supplementary Material
supplimentary_revised.doc
