# Supplementary material for: A benchmark study of k-mer counting methods for high-throughput sequencing
Source: Gigascience. 2018 Oct 22;7(12):giy125. doi: 10.1093/gigascience/giy125 (PMC6280066; doi:10.1093/gigascience/giy125)
Supplement: GIGA-D-17-00245_Revision_3.pdf [file giy125_giga-d-17-00245_revision_3.pdf]

# GigaScience

## A benchmark study of k-mer counting methods for high-throughput sequencing

--Manuscript Draft--

| | |
|---|---|
| **Manuscript Number:** | GIGA-D-17-00245R3 |
| **Full Title:** | A benchmark study of k-mer counting methods for high-throughput sequencing |
| **Article Type:** | Review |
| **Funding Information:** | |
| **Abstract:** | With the fast development of high-through sequencing technologies, hundreds of gigabytes of sequence data are produced. Many applications of bioinformatics require counting substrings of length k in such data (DNA/RNA sequencing reads), such as genome and transcriptome assembly, error correction, multiple sequence alignment, repeat detection and many other applications. In recent years, several techniques have been developed to count k-mers in such large sequencing data in a way that realizes time and memory trade-off. This paper presents an assessment strategy for several k-mer counting programs and evaluates their relative performances. Counting performance is evaluated primarily on the basis of runtime and memory usage. The evaluation presented in this paper also considers additional parameters, such as disk usage, accuracy, and parallelism as well as the impact of compressed input, the performance for large values of k and the scalability to larger datasets, such as human genome datasets. This work provides a specific recommendation for the current state-of-the-art program for a particular setup as well as suggestions for further development. All k-mer counting programs evaluated in this article are freely available and can be downloaded from hosting websites. |
| **Corresponding Author:** | Swati Chandrakant Manekar, M.Tech.<br>Visvesvaraya National Institute of Technology<br>Nagpur, Maharashtra INDIA |
| **Corresponding Author Secondary Information:** | |
| **Corresponding Author's Institution:** | Visvesvaraya National Institute of Technology |
| **Corresponding Author's Secondary Institution:** | |
| **First Author:** | Swati Chandrakant Manekar, M.Tech. |
| **First Author Secondary Information:** | |
| **Order of Authors:** | Swati Chandrakant Manekar, M.Tech. |
| | Shailesh Sathe, Ph.D. |
| **Order of Authors Secondary Information:** | |
| **Response to Reviewers:** | We are very thankful for giving acceptance to our manuscript with minor revision. We appreciate the time and effort spent by the reviewer to provide us his valuable insight and helpful feedback. The comments received from the reviewer helped to significantly improve our manuscript. We believe we have incorporated all the comments and suggestions made by the reviewer and enhanced our manuscript where necessary. We address the comments individually with suitable responses as follows,<br><br>Reviewer #2:<br><br>Comment 2.1<br>page 3: "For instance, k-mer frequencies are used to assess a probable misalignment among reads, which is either a sequencing error or a genuine nucleotide variation [10]."<br>-> I am still still unsure about that sentence, as I couldn't find where in the Quake paper is this matter discussed. Could you please point it to me, or remove this sentence? It is not clear to me whether any alignment "among reads" are being made, but instead, alignment between reads and a reference genome. |

>>
We thank the reviewer for a detailed review. We agree to the point raised herein and hence we have removed this sentence from the manuscript as suggested by the reviewer.
>>

Comment 2.2
page 4: "Statistics on the number of frequencies of all the k-mers"
->The meaning of "number of frequencies" is unclear.
>>
In this sentence, "number of frequencies" is replaced with "number of occurrences" in the manuscript.
>>

Comment 2.3
page 4: "use arrays with substring {k-mer} indexing."
->How is a flat array indexed by substrings? isn't it rather a dictionary?
>>
We agree to the point raised herein and hence we have modified the text in this regard.
Modified Text:
A naive approach for k-mer counting is to use a dictionary, with k-mers as keys and their counts as values.
>>

Comment 2.4
page 4: "the approach will soon overwhelm"
->More definitive wording can be used, I suggest "the approach overwhelms".
>>
"the approach will soon overwhelm" is modified to "the approach overwhelms".
>>

Comment 2.5
page 4: "a magnitude"
->"an order of magnitude"
>>
We have replaced "a magnitude" with "an order of magnitude" in the sentence on line number 18 and page number 4.
>>

Comment 2.6
page 5: "The disk-based approaches achieve very high efficiency at a marginal increase in the cost."
->"Cost" in what resource?
>>
Cost is in regard of I/O. As opposed to disk-based approach, in-memory approach puts everything in memory and therefore involves almost no I/O costs [this sentence is referred from Jellyfish {Memory} article]. We have modified the text in this regard in the manuscript.
Modified Text:
The disk-based approaches achieve very high efficiency at a marginal increase in the I/O costs.
>>

Comment 2.7
page 5: Just a remark; on top of what is written there, long reads are also notoriously used at resolving repetition in genome assemblies, not just structural variants.
>>
We agree to the point raised herein and hence we have modified the text in this regard.
Modified Text:
The long reads are used at resolving repetition in genome assemblies; they also facilitate better resolution of structural variants present in DNA samples and genomic repeat content [27], along with many other advantages [26].
>>

Powered by Editorial Manager® and ProduXion Manager® from Aries Systems Corporation

Comment 2.8
page 5: "Large values of k {k values up to 200} facilitate improvement of the accuracy of long sequencing reads {particularly of repeat-overlapping reads} and contig assembly [28]."
->Please note that this article isn't about "long reads" in the PacBio/Nanopore sense, but rather slightly longer Illumina reads.
>>
We agree to the point raised herein and hence we have modified the text in this regard.
Modified Text:
Large values of k {k values up to 200} facilitate improvement of the accuracy of longer Illumina reads {particularly of repeat-overlapping reads} and contig assembly [28].
>>

Comment 2.9
page 6: "highest N50 are obtained at an optimal choice of k, which seems to be larger values of k"
->No, not necessarily for larger values of k. {Only when there is sufficiently high coverage.}
>>
We agree to the point raised herein and hence we have modified the text in this regard.
Modified Text:
Empirically, the best assemblies {without miss-assembly} and the highest N50 {only when there is sufficiently high coverage} are obtained at an optimal choice of k, which seems to be larger values of k [29, 30].
>>

Comment 2.10
page 7 typo: "reprove"
>>
We have incorporated the change in the corresponding sentence as suggested above and hence reprove is replaced with reprobe.
>>

Comment 2.11
page 7: "Jellyfish follows a 'quotienting' technique"
->This seems to me that this quotienting technique is just a regular insertion procedure in a hash table.
>>
We agree to the point raised herein and hence we have removed the text in this regard as it has already been covered in the subsequent sentences.
>>

Comment 2.12
page 8: I suggest that the original Turtle algorithms may be presented in "k-mer counting using the sorting approach" for greater clarity that it is indeed a sorting-based algorithm.
>>
We agree to the point that Turtle follows a sorting-based approach and hence Turtle algorithms are now presented in "k-mer counting using the sorting approach" for greater clarity along with the proper rearrangement of references.
>>

Comment 2.13
page 9: "The k-mers are hashed using a one-way hash function,"
->I understand where this sentence is coming from, but the general concept of Squeakr could in principle also use an invertible hash function {which is in fact the main ingredient in Squeakr-exact}.
>>
We agree to the point raised herein and hence we have modified the text in this regard.
Modified Text:
Squeakr {exact}, which counts the frequency of each k-mer exactly using an invertible hash function, is not considered in this benchmark study, as its code is not available yet {currently not suitable for this benchmark study}.
>>

Powered by Editorial Manager® and ProduXion Manager® from Aries Systems Corporation

| | |
|---|---|
| | Comment 2.14<br>page 10: "Burst trie manages large sets [..]"; grammar, perhaps revise to "Burst tries manage large sets [..]"<br>>><br>We have modified the corresponding sentence as suggested herein. ‘Burst trie manages’ is replaced by ‘Burst tries manage’.<br>>><br><br>Comment 2.15<br>page 10: "extended k-mers, similar to KMC2", for historical accuracy, please mention that {k+x}-mers were introduced by KMC2.<br>>><br>We have added the sentence as suggested by the reviewer in the manuscript as follows.<br>Modified Text:<br>Extended k-mers {{k + x}-mers for x > 0} are substrings of length more than k and were introduced by KMC2.<br>>><br><br>Comment 2.16<br>page 11: "uses hashing approach for k-mer counting such as DSK" grammar, please revise to "uses a hashing approach for k-mer counting similar to DSK".<br>>><br>We agree to the point raised herein and hence we have modified the text in this regard.<br>Modified Text:<br>Gerbil [31] uses a hashing approach for k-mer counting similar to DSK.<br>>><br><br>Comment 2.17<br>page 11: what is a "part hash function" ?<br>>><br>Actually it is ‘partHash’, not ‘part hash’. Hence, we have replaced ‘part hash’ with ‘partHash’ along with appropriate citation. The partHash function is explained in the ‘Gerbil’ article {Page No. 4}.<br>>><br><br>Comment 2.18<br>page 11: "The algorithm avails high parallelization." I am unsure if this is correct in English.<br>>><br>We agree to the point raised herein and hence we have modified the text in this regard.<br>Modified Text:<br>The algorithm is designed to make optimal use of the hardware with multiple threads running concurrently.<br>>><br><br>Comment 2.19<br>page 17: "adopted" typo -> "adapted" and the beginning of the sentence "The commands used to run all the programs" is repeated twice<br>>><br>Corrections suggested herein are reflected in the manuscript and repetition is now removed.<br>Modified Text:<br>The commands used to run all the programs were adapted from their documentation and the publications of KMC3 and KMC2. These commands are given in the Supplementary Material.<br>>><br><br>Comment 2.20<br>page 17: "are available with the histogram subroutine." is awkward wording {- "can create histograms directly"}<br>>><br>Corrections suggested herein are reflected in the manuscript and hence ‘are available |

| | |
|---|---|
| | with the histogram subroutine' is replaced with 'can create histograms directly'.<br>Modified Text:<br>Some tools, such as Jellyfish, DSK, Gerbil and MSPKmerCounter can create histograms directly.<br>>><br><br>Comment 2.21<br>page 18: "The results are not entirely wrong for [..]," is vague<br>>><br>We agree to the point raised herein and hence we have modified the text in this regard to make it precise.<br>Modified Text:<br>Not all the k-mers with their counts in the outputs of Turtle, MSPKmerCounter and Gerbil {only for k = 55} are matching with the outputs of the other tools, for respective datasets.<br>>><br><br>Comment 2.22<br>note: in my version of the manuscript, Figures 1 & 2 are blurry because in JPG format, they should be uploaded in EPS or PDF format<br>>><br>Figures 1 & 2 are now uploaded in PDF format with better clarity.<br>>><br><br>Comment 2.23<br>page 29: "in such large set of reads {massive datasets}" {redundant formulation}<br>>><br>Redundant formulation has been removed from the sentence as suggested herein.<br>Modified Text:<br>There is a need to continue the development of a system that realizes memory and time trade-off for k-mer counting in such large set of reads.<br>>><br><br>Note: Since we are not able to submit 'Respond to Reviewers' having "opening parenthesis" <Error message : Unauthorized Content>, hence we have used "curly braces" in above responses. |
| **Additional Information:** | |
| **Question** | **Response** |
| Are you submitting this manuscript to a special series or article collection? | No |
| **Experimental design and statistics**<br><br>Full details of the experimental design and statistical methods used should be given in the Methods section, as detailed in our Minimum Standards Reporting Checklist. Information essential to interpreting the data presented should be made available in the figure legends.<br><br>Have you included all the information requested in your manuscript? | Yes |
| **Resources** | Yes |

| | |
|---|---|
| A description of all resources used, including antibodies, cell lines, animals and software tools, with enough information to allow them to be uniquely identified, should be included in the Methods section. Authors are strongly encouraged to cite Research Resource Identifiers (RRIDs) for antibodies, model organisms and tools, where possible.<br><br>Have you included the information requested as detailed in our Minimum Standards Reporting Checklist? | |
| **Availability of data and materials**<br><br>All datasets and code on which the conclusions of the paper rely must be either included in your submission or deposited in publicly available repositories (where available and ethically appropriate), referencing such data using a unique identifier in the references and in the "Availability of Data and Materials" section of your manuscript.<br><br>Have you have met the above requirement as detailed in our Minimum Standards Reporting Checklist? | Yes |

Manuscript

Click here to access/download;Manuscript;Manuscript_revision3.doc

Click here to view linked References

# A benchmark study of *k*-mer counting methods for high-throughput sequencing

Swati C. Manekar[1*] and Shailesh R. Sathe[1]

[1]Department of Computer Science and Engineering, Visvesvaraya National Institute of Technology, Nagpur 440 010, India

*corresponding author, Email: swati.manekar@gmail.com

**Abstract**

With the fast development of high-through sequencing technologies, hundreds of gigabytes of sequence data are produced. Many applications of bioinformatics require counting substrings of length *k* in such data (DNA/RNA sequencing reads), such as genome and transcriptome assembly, error correction, multiple sequence alignment, repeat detection and many other applications. In recent years, several techniques have been developed to count *k*-mers in such large sequencing data in a way that realizes time and memory trade-off. This paper presents an assessment strategy for several *k*-mer counting programs and evaluates their relative performances. Counting performance is evaluated primarily on the basis of runtime and memory usage. The evaluation presented in this paper also considers additional parameters, such as disk usage, accuracy, and parallelism as well as the impact of compressed input, the performance for large values of *k* and the scalability to larger datasets, such as human genome datasets. This work provides a specific recommendation for the current state-of-the-art program for a particular setup as well as suggestions for further development. All *k*-mer counting programs evaluated in this article are freely available and can be downloaded from hosting websites.

***Keywords:*** *k*-mer counting; high-throughput sequencing; disk-based counting; in-memory counting; hash table; sorting

## Introduction

*k*-mer counting is an important step in many bioinformatics applications used in the analysis of sequence data. Several tools and techniques have been developed in the last few years to count the frequency of *k*-length substrings (*k*-mer) in sequencing reads generated from high-throughput sequencing [1]. *k*-mer counting is a process of counting the number of occurrences of every substring having length *k* in a string *S* or a set of strings, where *k* is a positive integer. Let ∑= {*A*, *C*, *G*, *T*, *N*} denote the alphabet of nucleotides of DNA sequences, where *N* denotes an undetermined character by the sequencer. A read *r* is a sequence of nucleotides over the alphabet ∑. In a sequence dataset, different reads can contain the same sequence of nucleotides. Let *R* denote a dataset having *n* reads, such that $R = \{r_i;\ 1 \leq i \leq n\}$. Consider an example dataset *R* containing 3 reads each of length 6, *R* = {*ACGTTA*, *ACGTTA*, *ACGTTT*}, having 2 sequences {*ACGTTA*, *ACGTTT*}. For *k* = 4, there are nine 4-mers (3 in each read) {*ACGT*, *CGTT*, *GTTA*, *ACGT*, *CGTT*, *GTTA*, *ACGT*, *CGTT*, *GTTT*}. On counting, four unique 4-mers are obtained, which can be represented along with their counts in a tab-delimited format such as {*ACGT* 3, *CGTT* 3, *GTTA* 2, *GTTT* 1} [2].

*k*-mer counting has applications in genome assembly, e.g., using the overlap layout consensus approach [3–5] or the de Bruijn graph approach [6–9]. Errors in sequencing reads are corrected to improve the quality of the genome assembly. Error correction based on the *k*-mer spectrum approach [10–13] or multiple sequence alignment approach [14] relies on counting and keeping track of *k*-mers. *k*-mer counting is also used (for fast distance estimation) in creating multiple alignments of protein sequences [15]. In *de novo* genome projects, genomic characteristics such as genome size, repeat structure and heterozygous rate have been estimated by analysing the *k*-mer frequency distribution in the sequencing data [16]. Statistics on the number of occurrences

of all the *k*-mers are used to find the high-frequency *k*-mers, which are used as seeds to build a set of repeat families [17]. ReAS [18] also uses such high-frequency *k*-mers (obtained from *k*-mer repeat libraries) to seed the process of transposable element (TE) reconstruction. Identification of repeated sequences is the main step in genome analysis and annotation. *De novo* repeat identification techniques such as RAP [19], FORRepeats [20] and by Healy et al. [21] use *k*-mer occurrences to find candidate regions (repeated regions identification). Tallymer [22] also uses *k*-mer frequencies to annotate repetitive plant genomes. Quantitative features of complex repetitive DNA in several genomes have been studied by studying the distribution of frequencies of long *k*-mers ($20 \leq k \leq 100$) [23]. The counts of *k*-mers are also used to infer the genotypes of known variants [24].

Although *k*-mer counting is a simple and straightforward task, it becomes challenging when billions of next-generation sequencing (NGS) reads need to be processed using reasonable amounts of memory and in minimal time. A naive approach for *k*-mer counting is to use a dictionary, with *k*-mers as keys and their counts as values. When there are billions of such input reads, the approach overwhelms the memory capacity of the commodity computer. Approaches proposed so far have mainly targeted memory- and time-efficient solutions for *k*-mer counting. One of the ways to achieve memory efficiency is to represent the string data using unsigned integers. Disks are always an order of magnitude cheaper than memory. Therefore, many researchers have focused on using the disk to reduce memory usage. The approach is termed the disk based/external memory/out-of-core approach, as opposed to the in-memory/internal memory approach.

In this article, a review of *k*-mer counting approaches for high-throughput sequencing data is presented along with their comparative evaluation. The main purpose of this article is to provide

a general set of benchmarks and assessment metrics. This article also covers the experimental analysis of *k*-mer counting tools, providing a thorough insight for both beginners and consultants. Perez et al. [25] studied various *k*-mer counting tools for two values of *k*, i.e., 31 and 55, on a single dataset. We present a detailed performance evaluation of several of the latest and competitive tools on different datasets of varying sizes. We aim to extensively benchmark different popular *k*-mer counters. The primary focus of our study is to evaluate the *k*-mer counting tools for runtime and memory usage. However, we consider several other parameters in addition to the existing state-of-the-art literature. These parameters are (i) the scalability to larger values of *k*, (ii) the scalability to larger datasets, (iii) the impact (on runtime, memory, disk and central processing unit (CPU) utilization) of compressed inputs, i.e., gzip and bzip2 (multiple compressed FASTA/FASTQ input files), (iv) the scaling properties (speedup) with respect to the number of threads, (v) the accuracy and (vi) the maximum temporary disk usage. The scalability is measured in terms of runtime, memory, disk usage and CPU utilization.

Time, CPU, and memory are the bounded (limited) resources, whereas the disk can be considered a plentiful resource. The disk-based approaches may additionally use hundreds of gigabytes of disk space for large datasets such as the human genome dataset. Hence, we record the maximum disk utilization of all disk-based approaches. The disk-based approaches achieve very high efficiency at a marginal increase in the I/O costs.

In bioinformatics, with the advancements in next-generation sequencing technologies, long reads are generated. Such long reads are excellent at resolving complex RNA-splicing patterns from cDNA libraries [26]. The long reads are used at resolving repetition in genome assemblies; they also facilitate better resolution of structural variants present in DNA samples and genomic repeat content [27], along with many other advantages [26]. However, the longer reads of

Illumina technology suffer from lower accuracy [27]. Large values of *k* (*k* values up to 200) facilitate improvement of the accuracy of longer Illumina reads (particularly of repeat-overlapping reads) and contig assembly [28]. Empirically, the best assemblies (without miss-assembly) and the highest N50 (only when there is sufficiently high coverage) are obtained at an optimal choice of *k*, which seems to be larger values of *k* [29, 30]. Hence, we evaluate the performance of different *k*-mer counting tools for such large values of *k*.

The article is organized as follows. The algorithmic study of each approach is presented in the first section, followed by the benchmark datasets, along with the tools considered for this study. The results of the comparisons are discussed separately for each parameter in the subsequent section. The article concludes with guidelines and future research directions.

## Overview of *k*-mer counting approaches

The different *k*-mer counting tools can be categorized based on the approach and data structures they use, as shown in Table 1. Comprehensive information about each *k*-mer counting tool, i.e., its approach, the data structures used, *k*-size it can handle, etc., are given in Supplementary Table S1.

**Table 1** Ontology of *k*-mer counting approaches

| Approach for *k*-mer counting | Disk-based | In-memory |
|---|---|---|
| Hash table | Gerbil [31], MSPKmerCounter [32], DSK [33] | Squeakr [34], Jellyfish [35], BFCounter [36] |
| Sorting | KMC3 [37], GenomeTester4 [38], KMC2 [39], KAnalyze [40], KMC1 [41] | Turtle [42] |
| Burst tries | - | KCMBT [43] |
| Enhanced suffix array | - | Tallymer [22] |

*k-mer counting using the sorting approach*

This approach works by sorting all *k*-mers extracted from each read. Thus, *k*-mer frequencies can be easily counted because after sorting, repeating *k*-mers lay at adjacent positions in the sorted list.

GenomeTester4 (GListMaker) [38] uses the sorting approach for *k*-mer counting and works as follows: (i) In the reading phase, temporary arrays are used to gather all *k*-mers from the input file; and (ii) the *k*-mers stored in these arrays are first sorted and then counted during the collation phase. The counting results in temporary arrays (tables) are then merged to produce the final *k*-mer count list. GenomeTester4 uses multiple threads to speed up the counting of *k*-mers.

Turtle [42] uses a novel sorting and compaction (SAC)–based algorithm. This algorithm is a memory-efficient solution for *k*-mer counting. Turtle works as follows: *k*-mers are added to a big array up to a certain point, each with a count of one. This array is then sorted. The identical *k*-mers are then compacted, and their counts are added up in the compaction step. This is called the SAC approach. The compaction process frees up space in the array, which is used for a new set of *k*-mers. The SAC approach is then applied on the existing and newly added *k*-mers. This process is performed iteratively until all *k*-mers are counted. The compaction process is similar to run-length encoding [44]. Turtle has three implementations, scTurtle, cTurtle and aTurtle, varying in their outputs. scTurtle has false positive and outputs only *k*-mers with frequency > 1. cTurtle accepts small rates of false positives and false negatives and gives only *k*-mers with frequency > 1 without any counts. cTurtle gives an approximate set of frequent *k*-mers by using a counting Bloom filter. aTurtle provides *k*-mers of all frequencies with their counts. Although multithreaded, cTurtle and scTurtle do not give perfect counting, whereas single-threaded aTurtle provides perfect *k*-mer counting. Hence, in this study, aTurtle is considered.

scTurtle [42] uses a pattern block Bloom filter to remove all single-occurrence (spurious) *k*-mers. All such non-spurious *k*-mers are then counted using the SAC approach. Pattern block Bloom filter [45] is a cache-friendly variant of Bloom filter in which the cache miss ratio is very small.

*k-mer counting using a hash table*

For *k*-mer counting, a hash table [46] can be used, where *k*-mers are stored as keys, and their counts are stored as values. Jellyfish [35] uses hash tables for *k*-mer counting. This approach introduces a lock-free hash table to allow parallel insertion of *k*-mers and frequency updates by multiple threads using a CAS (compare-and-swap) assembly instruction [47]. The CAS operation detects simultaneous access to a shared memory location in the multithreaded environment. For storing the hash table, the entire memory is used. Once the hash table is full, it will get written to disk instead of doubling its size in the memory, and intermediate *k*-mer counts are then merged [48, 49]. Jellyfish works as follows: whenever a new *k*-mer appears, its key is obtained, and it is searched in the hash table; if it exists, its frequency count is incremented by one, else this *k*-mer is inserted (with its frequency set to one) into the hash table using the reprobe strategy. If a collision occurs, it is resolved using a quadratic probing (open addressing) technique [46].

A more efficient version of Jellyfish is available, Jellyfish 2, with an additional Bloom filter-based mode. This mode implements a Bloom filter to remove all singleton *k*-mers (i.e., *k*-mers that occur only once in the set of reads). A modified version of the Jellyfish 2 library is used by KAT [50] for *k*-mer counting.

*k-mer counting with the application of Bloom filter, its variants and counting quotient filter*

A major part of the entire genomic dataset is consumed by single frequency *k*-mers, which are mainly due to sequencing errors. A Bloom filter [51] is a probabilistic data structure used for

dynamic membership query lookup, which can implicitly store all *k*-mers. It is used to filter out singleton *k*-mers. The frequency of every non-singleton *k*-mer can then be counted by using any one of the approaches in Table 1. The Bloom filter has some number of false-positive membership query results, which may lead to miscounting of *k*-mers. However, with a reasonable choice of the number of hash functions, the false-positive rate can be minimized to an acceptable degree [52]. Though Bloom filter has some false-positive membership query results, it requires very low memory (i.e., only to store a bit-vector [52]), which highly reduces the overall memory requirement.

BFCounter [36] uses the same concept of a Bloom filter to filter out singleton *k*-mers and uses a hash table to store and count non-singleton *k*-mers. Some percentage of singleton *k*-mers may also get added in the hash table because of false positives, which produce erroneous counting results. However, BFCounter generates correct results by re-iterating over the sequence reads.

Squeakr [34] is an in-memory approach for both the approximate and exact *k*-mer counting. This approach uses a counting filter data structure (counting quotient filter (CQF) [53]) to store the *k*-mer counts. The *k*-mers are hashed using a one-way hash function, and these hashes are stored in a counting filter. The algorithm consists of a single phase and is based on the following ideas: multiple threads read input data from the disk in chunks and insert *k*-mers simultaneously into a global shared thread safe CQF for *k*-mer counting. Each thread maintains a local CQF to hold the *k*-mer counts temporarily to reduce waiting time while acquiring a lock in the global CQF (it is hardest to acquire the lock when repetitive *k*-mers are present in the dataset). Once the local CQF is full, it dumps the counted *k*-mers into the global CQF before processing a new set of *k*-mers. Squeakr (exact), which counts the frequency of each *k*-mer exactly using an invertible

hash function, is not considered in this benchmark study, as its code is not available yet (currently not suitable for this benchmark study).

*Enhanced suffix array–based counting*

Tallymer [22] is an in-memory approach, which uses a longest common prefix (lcp)-interval tree constructed from an enhanced suffix array [54] for *k*-mer counting. The lcp-interval tree implicitly stores the number of occurrences of all substrings of *s*' (reads are concatenated into a string *s*' with a unique termination symbol ($\$_i$) appended to each read). The algorithm is implemented in two steps: (i) the divide step splits sequence *s*' into smaller distinct partitions. The *k*-mers in each such partition are then counted using the lcp-interval tree; and (ii) in the merging step, a final count is generated by merging the counts generated from all distinct partitions using the sequence *s*'. Suffix array construction of a string is expensive in terms of computation and memory requirement. Suffix array size increases linearly with the length of string *s*'.

*Trie data structure–based k-mer counting*

KCMBT [43] is an in-memory approach, which uses burst trie, a variant form of a trie [55]. Burst tries manage large sets of strings efficiently in memory and maintains strings in sorted or nearly sorted order. KCMBT implements the concept of extended *k*-mers, similar to KMC2. Extended *k*-mers ($(k + x)$-mers for $x > 0$) are substrings of length more than *k* and were introduced by KMC2. The KCMBT algorithm consists of three phases, as explained below.

In the first phase, $(k + x)$-mers are generated from the input reads and inserted into the corresponding trees. For inserting $(k + x)$-mers into trees, a fixed length container is maintained initially for each tree. When a container is full, it bursts and is replaced by a new trie node and a set of child containers. These child containers partition $(k + x)$-mers of the original container

among themselves by taking a one-symbol prefix (matching A/C/G/T) of the ($k + x$)-mers ($x$ is chosen empirically to be $0 \le x \le 3$ for better performance). In the second phase, every ($k + x$)-mer tree (($k + 1$)-mer, ($k + 2$)-mer and ($k + 3$)-mer trees) is traversed to count all unique ($k + x$)-mers [42]. Then, ($k + x$)-mers are broken into *k*-mers. From the counts of the ($k + x$)-mers, the counts of its constituent *k*-mers are obtained. This result is because the ($k + x$)-mer covers ($x + 1$) *k*-mers. These *k*-mers are then inserted into the *k*-mer trees. Finally, the *k*-mer trees are traversed to produce counts of all unique *k*-mers. The *k*-mers, along with their counts, are then written to the disk. As the inserted number of *k*-mers is much reduced due to ($k + x$)-mers, time for traversal in the last phase is reduced, which leads to faster computation. Thousands of trees with smaller heights are generated to reduce the overall insertion and traversal time for counting a huge number of *k*-mers. Burst trie is the data structure where the search is very fast, but the size of trie becomes large for large volumes of sequencing data.

**Disk-based *k*-mer counting**

Furthermore, the large memory requirement of the in-memory approach is reduced by the disk-based approach. The disk-based approach is a memory-frugal approach especially designed to make the *k*-mer counting possible for large genomic datasets such as a human genome dataset on commodity hardware. The memory usage can be greatly reduced using a disk, as *k*-mers are processed in chunks and are stored on disk. In the following section, the disk-based approaches such as DSK, KAnalyze, MSPKmerCounter, KMC2, KMC3 and Gerbil are presented, along with their comparative analysis.

DSK [33] is a disk-based approach that counts the *k*-mers using very low memory and disk space. To achieve this outcome, DSK calculates the number of partitions needed to bring data in parts from disk to memory, depending on (i) the total bits required to store the *k*-mers and (ii) the

disk size available. DSK calculates the number of iterations needed to read the entire set of input in parts, depending on (i) the total number of bits required to represent the entire set of *k*-mers, (ii) the memory size required to hold the hash table, (iii) the number of partitions and (iv) the load factor for which hash table gives the best performance. Each *k*-mer is distributed to one of multiple partitions, depending on its hash value and an iteration number. Partitions are stored on the disk. *k*-mers are counted by loading a partition into the memory one at a time using hash tables in multiple iterations. DSK implements an efficient partitioning strategy to address memory constraints, though it may result in high I/O cost.

KAnalyze [40] is a *k*-mer toolkit that uses a sorting-based approach for *k*-mer counting. The algorithm consists of two phases. In the initial phase, *k*-mers are filled into a temporary array of predefined size. Once the array is full, *k*-mers are sorted and counted. The counted *k*-mers are then written to disk so that space becomes available to count the next incoming chunk of *k*-mers. The process is repeated until all the *k*-mers are processed. In the second phase, the count files are loaded from disk to memory and are merged in multiple steps to generate final *k*-mer counts.

*Approaches using the concept of super k-mer: Minimizers and Signatures*

The disk-based compression technique, i.e., MSP (Minimum Substring Partitioning) [56] is used to reduce the memory requirements and I/O operations further, wherein input reads are broken into multiple disjoint partitions.

*k*-mers of reads carry highly redundant data, as there exists an adjacency relationship between every pair of *k*-mers. In the MSP approach, if consecutive *k*-mers of a read share the same lexicographical minimum substring *s*, then such *k*-mers are stored as one substring of length greater than *k*. This substring is called a super *k*-mer and is stored in a disk partition corresponding to the lexicographical minimum substring *s*, where *s* is termed a minimizer.

Larger numbers of consecutive *k*-mers sharing the same minimum substring *s*, give better compression ratio, which ultimately reduces both the I/O overhead and storage space.

MSPKmerCounter [32] is the first tool to implement MSP for *k*-mer counting and works as follows: (i) Reads are first decomposed into super *k*-mers and distributed to disk partitions (bins) identified by canonical minimizers. The storage of super *k*-mers with same canonical minimizer in the same partition assures that all the occurrences of the same *k*-mer belong to the same partition. This approach eliminates the need to merge the counts of each partition. These smaller partitions are easily accommodated into the memory and are processed independently. (ii) Once the partitions are ready, all super *k*-mers are broken into *k*-mers using simple bit shift operations. (iii) Finally, *k*-mers are counted using hash tables and counts are stored on disk.

KMC2 [39] is another disk-based approach that uses an approach similar to the MSP employed in MSPKmerCounter. Here, the minimizer approach is refined to signatures, which significantly reduce the overall memory requirements and temporary disk space. Canonical minimizers are used as signatures with the following three pre-requisites: canonical minimizers (1) do not begin with prefix *AAA*, (2) do not begin with prefix *ACA* and (3) do not either contain *AA* anywhere apart from the beginning. The KMC2 algorithm consists of two major phases: the distribution phase and sorting phase. The distribution phase is similar to that of MSPKmerCounter. The only difference is that super *k*-mers are distributed to different temporary files (bins) based on signatures instead of minimizers. In the sorting phase, bins are processed by fetching them into the memory. For every such bin, extended *k*-mers, i.e., $(k + x)$-mers are extracted from super *k*-mers, and radix sort is applied to them. *k*-mer statistics are then collected from these sorted $(k + x)$-mers. Finally, the results are stored on the disk.

KMC3 is an extension of KMC2 approach with few improvements such as (i) efficient input file reading to achieve better I/O subsystem, (ii) memory efficient approach of assigning signatures to bins and (iii) efficient sorting approach [57] rather than radix sort to make it work efficiently for larger values of *k*.

Gerbil [31] uses a hashing approach for *k*-mer counting similar to DSK. The algorithm consists of two major phases. The first phase is similar to the distribution phase of KMC2, with a little advancement. The hash values (obtained using a partHash [31] function) of *k*-mers (extracted from super *k*-mers) are used to ensure that multiple occurrences of the same *k*-mer are assigned to the same thread. In the second phase, super *k*-mers stored in the temporary files are sequentially re-read from the working disk. All *k*-mers are extracted from the super *k*-mers and then counted using hash table. The collisions are resolved using quadratic hashing. Every thread counts the assigned *k*-mers using its hash table. Finally, hash tables containing the counts of *k*-mers are written into an output file. The algorithm is designed to make optimal use of the hardware with multiple threads running concurrently. To achieve memory efficiency, hash table size is estimated using a simple linear model. In its GPU implementation, the second phase is performed on the GPU side with a proper load balancing between GPU and CPU.

## Benchmark datasets and evaluation methodology

We use seven datasets of varying sizes. To make a reasonable assessment, most of these datasets are the same as those used by Kokot et al. [37]. The details of the datasets used are summarized in Table 2. FV and DM are two small-sized datasets, whereas HS2 is the largest one. The NC and AT datasets (the same datasets that Gerbil [31] used) are chosen due to the longer length of reads, which facilitates testing of the performance for larger values of *k*. The information for downloading all seven datasets is given in the Supplementary Material (Table S2).

**Table 2** Datasets used in our study

| Sr. No. | Dataset ID | Organism | Genome size (Mbases) | Input FASTQ/FASTA file size (Gbytes) (1 Gbyte = $10^9$ bytes) | Average read length (bases) | Total no. of bases (Gbases) | Total no. of reads |
|---|---|---|---|---|---|---|---|
| 1 | FV | F. vesca | 214 | 10.9 | 353 | 4.5 | 12803137 |
| 2 | DM | D. melanogaster | 122 | 10.5 | 76 | 3.7 | 48432878 |
| 3 | MB | M. balbisiana | 472 | 197.1 | 100 | 56.3 | 562968372 |
| 4 | HS1 | H. sapiens 1 | 2991 | 292.1 | 151 | 123.7 | 819148264 |
| 5 | HS2 | H. sapiens 2 | 2991 | 339.5 | 100 | 135.3 | 1339740542 |
| 6 | NC | N. crassa | 41 | 23.3 | 7778.3 | 22.9 | 2942564 |
| 7 | AT | A. thaliana | 120 | 72.7 | 4804.6 | 36.1 | 7515360 |

The sequencing reads in these files (for each such dataset) are first decompressed and then concatenated into a single FASTA/FASTQ file to facilitate the easy running of each tool. Not all of the considered tools support direct decompression. All of the datasets used in this study have multiple compressed files. Some *k*-mer counting tools support the compressed input (raw input) directly. Such tools can perform parallelization effectively in their first phase by reading from individual input files using separate threads, which means that restricting the input to a single file effectively limits them to 1 or 2 threads (e.g., one to parse and one to bin/partition). It is likely that most of the tools would be able to perform even better on multi-file inputs without being concatenated into a single file (normalized input). Here, we test the impact of compressed input (gzip and bzip2) on the performance of various programs by running them directly on compressed input files.

We evaluate KMC3, Gerbil (version 1.0), KCMBT (version 1.0), MSPKmerCounter (version 0.1), GenomeTester4 (version 4.0), aTurtle (version 0.3), KAnalyze (version 2.0.0), DSK (version 2.2.0), Jellyfish (version 2.2.6) and BFCounter (version 1.0) (in chronological order of publication, oldest tools last.). Tools from 2010 or earlier are excluded, and a recent version of each tool is considered for this study. All mentioned tools are freely available to download (refer to Table S2 of the Supplementary Material).

Tables 4–8 present the comparison, which includes execution time (in seconds), maximum memory (RAM) (in gigabytes (GB)), disk consumption (in gigabytes (GB)) and average CPU utilization (in %). Each program has been tested on the FV, DM, MB, HS1 and HS2 datasets for two values of *k* (28 and 55). A test lasting longer than 15 hours was interrupted.

The tools that give an approximation of the frequency histogram of *k*-mer occurrences (and/or estimation of the number of unique *k*-mers and singleton *k*-mers) by streaming analysis of data are not considered in this paper. These tools include KmerStreame [58], ntCard [59], KmerGenie [30] and Khmer [60]. Khmer [60] is the latest implementation built on the library in [61] that uses hyperloglog, which is the probabilistic approach for approximate cardinality estimation. All these tools use a significantly lower amount of memory and are reasonably fast. To make a fair comparison, we have tested only those tools that generate exact *k*-mer counts.

The time considered is the wall clock time measured using the C++ function, 'gettimeofday()' averaged over three runs. To measure the maximum memory usage, a shell script by Jaeho Shin is used and is available at https://github.com/jhclark/memusg. The script uses the Linux *ps* utility and finds the peak memory uses of a process and its threads. The script monitors the resident set size (*RSS*) values, where *RSS* reports the amount of memory actually allocated to a process and is in memory (RAM). We have written a shell script to report the maximum disk usage by the program, which logs the disk usage at regular time intervals between consecutive checks using the Linux command *du*. The same shell script also captures average CPU utilization in percentage with the help of the Linux command *top*. These scripts are executed with a sampling rate of three for the HS1 and HS2 datasets, and a sampling rate of one for the FV, DM, MB, NC and AT datasets. The invocations of all executables are monitored by the above-mentioned shell

scripts. Running time, memory usage, disk usage and CPU utilization are measured simultaneously in the same run.

All experiments were performed on the test machine with the configuration shown in Table 3. The commands used to run all the programs were adapted from their documentation and the publications of KMC3 and KMC2. These commands are given in the Supplementary Material. The commands to see the results as a list of *k*-mers along with their counts in human-readable format and *k*-mer coverage distribution (histogram for *k*-mer abundance) are given in the Supplementary Material.

**Table 3** Test Machine configuration

| | |
|---|---|
| **Processor** | Intel(R) Xeon(R) CPU E5-2698 v3 @ 2.30GHz |
| **Main memory** | 64GB |
| **Hard Disk Drive** | 1 TB |
| **CPU(s)** | 16 |
| **On-line CPU(s) list** | 0-15 |
| **Thread(s) per core** | 2 |
| **Core(s) per socket** | 16 |
| **No of socket** | 1 |

We evaluate the accuracy of the counting programs by comparing their *k*-mer frequency histograms (including the number of singleton *k*-mers $f_1$) on two small-sized datasets with two values of *k*. This histogram is a table of $f_i$ values, where $f_i$ denotes the number of distinct *k*-mers that appear *i* times in the set of reads [58]. Some tools, such as Jellyfish, DSK, Gerbil and MSPKmerCounter can create histograms directly. For the remaining tools, the *k*-mer frequency histograms were obtained in two steps. First, the dump subroutine of the tool was run, which wrote *k*-mer occurrences into a tab-separated text file. Second, we used our program written in C++ using OpenMP for multithreaded computing and Linux commands (*grep* and *wc*) to generate the *k*-mer frequency histogram from this text file.

The exact numbers of $f_1$ $f_2$ $f_3$.. $f_{10}$ of all ten tools are reported in the appendix for the FV and DM datasets for two values of *k* (28 and 55). The results are reported up to $f_{10}$, owing to the space limitations, even though all the frequency counts are considered and compared. The results of Jellyfish 2.2.6, DSK 2.2.0, KAnalyze 2.0.0, KMC3, Gerbil 1.0, KCMBT 1.0, GenomeTester4 and BFCounter 1.0 are the same for both datasets and values of *k*. In contrast, for MSPKmerCounter 0.1, aTurtle 0.3 and Gerbil 1.0 (only for $k$ = 55), the results are different. The error rates of these three tools are provided in the Supplementary Material (Table S3–S6). MSPKmerCounter has the highest error rates, as depicted in Table S3–S6.

Not all the *k*-mers with their counts in the outputs of Turtle, MSPKmerCounter and Gerbil (only for $k$ = 55) are matching with the outputs of the other tools, for respective datasets. For more rigorous analysis, our shell script written using a set of Linux utilities, i.e., *sort* (to sort in lexicographical order) and *diff* (which analyses two files and prints the lines that are different), is used to validate all the lexicographically sorted *k*-mers along with their counts.

DSK output is used as a reference to validate the output of aTurtle because DSK and aTurtle use the same alphabetical order ($A < C < T < G$) while obtaining canonical *k*-mers. While comparing the lexicographically sorted *k*-mers in the outputs of aTurtle and DSK, variations are found (though the frequency counts of aTurtle match DSK for the DM dataset for $k$ = 55). The unmatched frequency *k*-mers and some additional *k*-mers are present in the output of aTurtle along with some missing *k*-mers compared with the output of DSK. Similar types of variation are observed on comparing the outputs of MSPKmerCounter and Gerbil (only for $k$ = 55) with the output of KMC3. These tools consider the same lexicographic ordering of canonical *k*-mers based on $A < C < G < T$, hence their outputs were compared.

We thus infer that the recent versions of MSPKmerCounter, aTurtle and Gerbil considered in

this study have bugs in their implementations.

## Result and discussion

**Table 4** Experimental results for the FV dataset

| SN | Tools (Version) | ***k* = 28** | | | | ***k* = 55** | | | |
|---|---|---|---|---|---|---|---|---|---|
| | | **Time (sec)** | **RAM (GB)** | **Disk (GB)** | **CPU Utilization (%) (Comment)** | **Time (sec)** | **RAM (GB)** | **Disk (GB)** | **CPU Utilization (%) (Comment)** |
| 1 | Jellyfish 2.2.6 | 138.33 | 7.9 | 0 | 1093.55 (Consistent) | 226 | ***36.19*** | 0 | **1050.93*** (Consistent) |
| 2 | DSK 2.2.0 | 56.33 | 6.35 | 6 | 866.50 (Consistent) | 78.33 | 7.04 | 5 | 633.49 (Declined from ~1174 to ~129.7) |
| 3 | DSK 2.2.0 (gzip) | 197 | 4 | 6 | 402.71 (Starting 80% of time consistent with ~300, for last 20% inconsistent to ~1200 with sudden increase) | 222 | 6 | 5 | 441.21 (Starting 75% of time consistent with ~390, last 25% inconsistent to ~1200 with sudden increase) |
| 4 | KAnalyze 2.0.0 | ***2042*** | 10 | ***22.2*** | 509.20 (Initially, in the range of 1000 – 2000 then declined to ~200) | ***4095*** | 11 | ***42*** | 337.46 (Initially, in the range of 1000 – 2000 then declined to ~150) |
| 5 | KAnalyze 2.0.0 (gzip) | 1999 | 9 | 22.9 | 507.84 (Starting 30% of time inconsistent in the range of 2250 – 750, last 70% consistent with sudden drop to ~200) | 3395 | 11 | 12.8 | 360.456 (Starting 25% of time inconsistent to ~900, last 75% consistent to ~200 with a sudden drop) |
| 6 | KMC3 | 38.66 | 7.66 | **4*** | 998.10 (Consistent) | **35*** | 11.2 | 4 | 987.891 (Consistent) |
| 7 | KMC3 (gzip) | 35 | 7 | 2.2 | 1004.61 (Consistent) | 37 | 11 | 0 | 1056.25 (Consistent) |
| 8 | Gerbil 1.0 | **33.66 *** | **848 MB*** | **4*** | **1110.38*** (Consistent) | 60.33 | **1.29*** | **3*** | 1030.50 (Consistent) |
| 9 | Gerbil 1.0 (gzip) | 49 | 841 MB | 1.5 | 858.46 (Starting 50% of time consistent with ~600, for last 50% of time suddenly increased to ~1200) | 55 | 1 | 1 | 880.77 (Starting 50% of time consistent with ~600, for last 50% of time suddenly increased to ~1300) |
| 10 | KCMBT 1.0 | 137.5 | ***30.98*** | 0 | 628.87 (Inconsistent) | Not supported | | | |
| 11 | MSPKmerCounter 0.1 | 59.33 | 4.45 | 1 | 811.70 (Phase 1: consistent (~200) Phase 2: consistent (~1500)) | 67.33 | 4.61 | 1 | 770.87 (Phase 1: consistent (~200) Phase 2: consistent (~1500)) |
| 12 | aTurtle 0.3 | 671 | 14 | 0 | ***99.14*** (Consistent) | 1185 | 26 | 0 | ***94.12*** (Consistent) |
| 13 | GenomeTester4 | 214 | 26 | 0 | ***202.33*** (Consistent) | Not supported | | | |
| 14 | BFCounter 1.0 | 1731 | 3 | 0 | 274.10 (Starting 80% of time almost 100 then gradually increased to ~1000) | 1790 | 9 | 0 | ***271.80*** (Consistent to ~100, but in the middle and end there are bars of hike to ~1000) |
| 15 | BFCounter 1.0 (gzip) | 1847 | 3 | 0 | 259.70 (Inconsistent) | 1889 | 9 | 0 | 251.49 (Inconsistent) |

Entries in bold with * indicate best results, whereas entries in bold with italic type (including second lowest for CPU utilization) show average results. MSPKmerCounter has the highest error rates. MSPKmerCounter results and the compressed input results are not considered while highlighting the best and average results. For column ‘Disk’, the best and average results are highlighted by considering disk-based tools only. Abbreviations: sec = seconds, GB = gigabytes, MB = megabytes.

**Table 5** Experimental results for the DM dataset

| SN | Tools (Version) | ***k* = 28** | | | | ***k* = 55** | | | |
|---|---|---|---|---|---|---|---|---|---|
| | | **Time (sec)** | **RAM (GB)** | **Disk (GB)** | **CPU Utilization (%) (Comment)** | **Time (sec)** | **RAM (GB)** | **Disk (GB)** | **CPU Utilization (%) (Comment)** |
| 1 | Jellyfish 2.2.6 | 77 | 4 | 0 | 1055.25 (Consistent) | 71 | 9 | 0 | 917.79 (Consistent) |
| 2 | DSK 2.2.0 | 52 | 2 | 4.2 | 736.09 (Initially, ~600 then increased to ~1173) | 49 | 2 | 2.7 | 622.36 (Initially, ~500 then increased to ~1150) |
| 3 | DSK 2.2.0 (gzip) | 183 | 4.68 | 3.6 | 331.88 (Starting 90% of time consistent with ~270, last 10% of time suddenly increased to ~1200) | 173 | 4.45 | 2.4 | 300.70 (Starting 90% of time consistent with ~250 then suddenly increased to ~1200) |
| 4 | KAnalyze 2.0.0 | 794 | 10 | ***14.3*** | 695.64 (Gradually declined) | 393 | 11 | ***12.8*** | 829.45 (Gradually declined from ~2000 to ~100) |
| 5 | KAnalyze 2.0.0 (gzip) | 822 | 9 | 14.4 | 691.15 (Starting 40% of time consistent with ~1250, next 60% of time inconsistent with sudden drop to ~200) | 411 | 11 | 12.9 | 843.79 (Starting 60% of time inconsistent in the range of range 2250 – 900, for the rest of time inconsistent with sudden drop to ~200) |
| 6 | KMC3 | **18*** | 5 | 2.23 | 942.26 (Consistent) | **13*** | 8 | **614.4 MB*** | **1023.54*** (Consistent) |
| 7 | KMC3 (gzip) | 35 | 5 | 1.64 | 739.01 (Last 20% of time consistent with ~1250 remaining of time consistent with ~700) | 31 | 8 | 0 | 637.29 (Last 20% of time consistent with ~1250, rest of time consistent with ~600) |
| 8 | Gerbil 1.0 | 20 | **827 MB*** | **2.11*** | **1184.23*** (Consistent) | 16.5 | **826 MB*** | 4 | 1010.89 (Consistent) |
| 9 | Gerbil 1.0 (gzip) | 33 | 837 MB | 1.31 | 821.69 (Starting 55% of time consistent with ~700, last 45% suddenly increased to ~1200) | 29 | 834 MB | 0 | 685.74 (Starting 55% of time consistent with ~600, last 45% inconsistent with sudden increase to ~1200) |
| 10 | KCMBT 1.0 | 61 | 2 | 0 | 595.37 (Initially, ~300 then increased towards end to ~900) | Not supported | | | |
| 11 | MSPKmer Counter 0.1 | 234 | 5 | 14.2 | 912.92 (For both phases: Consistent) | 219 | 5 | 11.2 | 914.62 (Phase1: Initially, ~1000 then declined to ~300 Phase2: Consistent) |
| 12 | aTurtle 0.3 | 423 | 7 | 0 | ***97.20*** (Consistent) | 330 | 12 | 0 | ***95.39*** (Consistent) |
| 13 | GenomeTester4 | 144 | ***23*** | 0 | ***183.92*** (Consistent) | Not supported | | | |
| 14 | BFCounter 1.0 | ***914*** | 1 | 0 | 307.53 (Consistent) | ***477*** | 2 | 0 | ***331.48*** (Starting 95% of time in the range of 250 – 400, then increased to ~800) |
| 15 | BFCounter 1.0 (gzip) | 1002 | 2 | 0 | 321.78 (Ending 20% of time ~500, rest is ~300) | 559 | 2 | 0 | 306.30 (Ending 20% of time ~500, rest is ~300) |

Entries in bold with * indicate best results, whereas entries in bold with italic type (including second lowest for CPU utilization) show average results. MSPKmerCounter has the highest error rates. MSPKmerCounter results and the compressed input results are not considered while highlighting the best and average results. For column 'Disk', the best and average results are highlighted by considering disk-based tools only. Abbreviations: sec = seconds, GB = gigabytes, MB = megabytes.

**Table 6** Experimental results for the MB dataset

| SN | Tools (Version) | ***k* = 28** | | | | ***k* = 55** | | | |
|---|---|---|---|---|---|---|---|---|---|
| | | **Time (sec)** | **RAM (GB)** | **Disk (GB)** | **CPU Utilization (%) (Comment)** | **Time (sec)** | **RAM (GB)** | **Disk (GB)** | **CPU Utilization (%) (Comment)** |
| 1 | Jellyfish 2.2.6 | **1467*** | 15 | 0 | **800.13*** (Consistent) | **1440*** | ***24*** | 0 | **691.65*** (Consistent) |
| 3 | DSK 2.2.0 | 3358 | 12 | 59 | 185.09 (Consistent) | 3039 | 11 | 45 | ***208.54*** (Consistent) |
| 4 | KAnalyze 2.0.0 | ***51422*** | 10 | ***189*** | 279.40 (Initially, ~2000 then declined to ~150) | ***45367*** | 11 | ***245*** | 248.04 (Declined from ~2000 to ~100) |
| 5 | KMC3 | 2019 | 9 | 36 | 216.93 (Initially, in the range of 12 – 400, increased towards end to ~600) | 1804 | 10 | 14 | 211.12 (Initially, in the range of 12 – 400, increased towards end to ~600) |
| 6 | KMC3 (bz2) | 3341 | 11 | 36.3 | 289.46 ( Starting 90% of time consistent in the range of 200 – 400, last 10% up to ~1300) | 3250 | 11 | 13 | 282.77 (Starting 90% consistent in range of 200 – 400, last 10% up to ~1300) |
| 7 | Gerbil 1.0 | 2238 | **2*** | **32*** | 269.52 (Initially, within 150, increased towards end to ~800) | 1941 | **3*** | **11*** | 250.32 (Initially within 150, increased towards end to ~800) |
| 8 | Gerbil 1.0 (bz2) | 3487 | 2 | 30.7 | 306.37 (Starting 90% of time consistent with ~270, last 10% suddenly increased to ~1300) | 3137 | 3 | 11 | 304.02 (Starting 90% of time consistent with ~270, last 10% suddenly increased to ~1300) |
| 9 | KCMBT 1.0 | 1644 | 34 | 0 | ***135.87*** (Consistent) | Not supported | | | |
| 10 | MSPKmerCounter 0.1 | 11094 | 8 | 173 | 316.90 (Consistent) | 8759 | 9 | 118 | 1284.05 (Consistent) |
| 11 | aTurtle 0.3 | 8764 | ***61*** | 0 | ***75.07*** (Consistent) | > 15 Hours | | | |
| 12 | GenomeTester 4 | 3520 | 60 | 0 | 153.67 (Consistent) | Not supported | | | |
| 13 | BFCounter 1.0 | 18950 | 10 | 0 | 300.37 (Consistent) | 15264 | 19 | 0 | 295.40 (Starting 50% up to ~254 then increased to ~434) |

If a program failed to complete the computation due to insufficient memory/disk space or within a stipulated time (15 hours), the respective entries in the above table are denoted by their failure messages. Entries in bold with * indicate best results, whereas entries in bold with italic type (including second lowest for CPU utilization) show average results. MSPKmerCounter has the highest error rates. MSPKmerCounter results and the compressed input results are not considered while highlighting the best and average results. For column 'Disk', the best and average results are highlighted by considering disk-based tools only. Abbreviations: sec = seconds, GB = gigabytes, MB = megabytes.

For the FV, DM, MB, HS1 and HS2 datasets, the outperforming results of different programs are shown in bold and with an asterisk (*), whereas the average results (including second lowest for CPU utilization) are indicated in bold and with italic type in Tables 4–8. The gzip/bzip2 results are not considered while highlighting the best and average results to facilitate reasonable comparisons.

For the FV and DM datasets, all programs completed the counting of *k*-mers within 15 hours. However, KCMBT and GenomeTester4 for the HS1 and HS2 datasets as well as Jellyfish for the HS1 dataset were unable to finish their work within 15 hours. In addition, their jobs had to be killed due to the large memory usage, which froze the system. We tested Jellyfish in its Bloom filter–based mode to allow it to complete *k*-mer counting for the HS1 dataset, but it could not finish its phase 1 within 15 hours and also due to freezing of system; we stopped its execution. aTurtle failed on the HS1 and HS2 datasets with the ‘std::bad_alloc Aborted (core dumped)’ error message due to its large memory usage. KAnalyze also failed on HS1 and HS2 with the ‘java.io.IOException: No space left on device’ error.

**Table 7** Experimental results for the HS1 dataset

| **SN** | **Tools (Version)** | ***k* = 28** | | | | ***k* = 55** | | | |
|---|---|---|---|---|---|---|---|---|---|
| | | **Time (sec)** | **RAM (GB)** | **Disk (GB)** | **CPU Utilization (%) (Comment)** | **Time (sec)** | **RAM (GB)** | **Disk (GB)** | **CPU Utilization (%) (Comment)** |
| 1 | Jellyfish 2.2.6 | > 15 Hours (system hang) | | | | > 15 Hours (system hang) | | | |
| 2 | DSK 2.2.0 | ***7722*** | ***12*** | ***133*** | ***210.2*** (Inconsistent) | ***9389*** | ***14*** | ***48*** | ***255.862*** (Inconsistent) |
| 3 | DSK 2.2.0 (gzip) | 9240 | 11 | 134 | 218.77 (Inconsistent) | 8480 | 12 | 104 | 284.68 (Inconsistent) |
| 4 | KAnalyze 2.0.0 | Failed, Error: IO error writing segment file: No space left on device | | | | Failed, Error: IO error writing segment file: No space left on device | | | |
| 5 | KAnalyze 2.0.0 (gzip) | Failed, Error: IO error writing segment file: No space left on device | | | | Failed, Error: IO error writing segment file: No space left on device | | | |
| 6 | KMC3 | **3725*** | 10 | 78 | 276.64 (Gradually declined) | **3466*** | **11*** | 28 | 270.55 (Inconsistent) |
| 7 | KMC3 (gzip) | 1964 | 11 | 79 | 620.84 (Inconsistent) | 1626 | 11 | 29 | 663.31 (Inconsistent) |
| 8 | Gerbil 1.0 | 4078 | **6*** | **66*** | **370.77*** (Initially, within ~200, increased towards end to ~1200) | 3818 | **11*** | **21*** | **320.21*** (Inconsistent) |
| 9 | Gerbil 1.0 (gzip) | 2849 | 6 | 66 | 569.83 (Starting 70% of time consistent to ~420 then increased to ~1000 for rest 30% of time) | 2614 | 11 | 22 | 541.63 (Starting 70% of time consistent to ~400 then increased to ~1000 for rest 30% of time) |
| 10 | KCMBT 1.0 | > 23 Hour | | | | Not supported | | | |
| 11 | MSPKmerCounter 0.1 | > 15 Hours (Phase 2 failed , OutOfMemoryError) | | | | > 15 Hours (Phase 2 failed , OutOfMemoryError) | | | |
| 12 | aTurtle 0.3 | Aborted (core dumped) | | | | Aborted (core dumped) | | | |
| 13 | GenomeTester4 | > 15 Hours | | | | Not Supported | | | |
| 14 | BFCounter 1.0 | > 15 Hours | | | | > 15 Hours | | | |
| 15 | BFCounter 1.0 (gzip) | > 15 Hours | | | | > 15 Hours | | | |

If a program failed to complete the computation due to insufficient memory/disk space or within a stipulated time (15 hours), the respective entries in the above table are denoted by their failure messages. Entries in bold with * indicate best results, whereas entries in bold with italic type (including second lowest for CPU utilization) show average results. MSPKmerCounter has the highest error rates. MSPKmerCounter results and the compressed input results are not considered while highlighting the best and average results. For column ‘Disk’, the best and average results are highlighted by considering disk-based tools only. Abbreviations: sec = seconds, GB = gigabytes, MB = megabytes.

**Table 8** Experimental results for the HS2 dataset

| SN | Tools (Version) | *k* = 28 | | | | *k* = 55 | | | |
|---|---|---|---|---|---|---|---|---|---|
| | | **Time (sec)** | **RAM (GB)** | **Disk (GB)** | **CPU Utilization (%) (Comment)** | **Time (sec)** | **RAM (GB)** | **Disk (GB)** | **CPU Utilization (%) (Comment)** |
| 1 | Jellyfish 2.2.6 | **3310*** | ***58*** | 0 | **1000.29*** **(**Consistent) | ***11126*** | ***48*** | 0 | **376.578*** (Declined from ~1000 to ~100) |
| 2 | DSK 2.2.0 | ***8879*** | 13 | ***145*** | ***186.66*** (Consistent) | 7982 | 13 | ***109*** | ***211.54*** (Consistent) |
| 3 | DSK 2.2.0 (gzip) | 10360 | 10 | 146 | 242.01 (Inconsistent ) | 10199 | 12 GB | 109 | 240.21 (Starting 60% of time consistent with ~300 , last 40% suddenly increased to ~650) |
| 4 | KAnalyze 2.0.0 | Failed, Error: IO error writing segment file: No space left on device | | | | Failed, Error: IO error writing segment file: No space left on device | | | |
| 5 | KAnalyze 2.0.0 (gzip) | Failed, Error: IO error writing segment file: No space left on device | | | | Failed, Error: IO error writing segment file: No space left on device | | | |
| 6 | KMC3 | 4252 | 10 | 85 | 218.02 (Increased towards end to ~600, otherwise it is up to ~12) | **3846*** | 11 | 29 | 214.99 (Increased towards end to ~600, otherwise it is up to ~12) |
| 7 | KMC3 (gzip) | 2362 | 10 | 86 | 580.72 (Inconsistent) | 1995 | 11 | 29 | 556.31 (Inconsistent) |
| 8 | Gerbil 1.0 | 4553 | **5*** | **74*** | 371.26 (Increased towards end to ~1000, otherwise it is up to ~250) | 4260 | **9*** | **23*** | 317.65 (Initially, ~250, increased towards end to ~1000) |
| 9 | Gerbil 1.0 (gzip) | 3358 | 5 | 74 | 553.59 (Starting 70% of time consistent ~400 then increased to ~1000 for rest 30% of time) | 3121 | 9 | 23 | 507.19 (Starting 70% of time consistent to ~450 then increased to ~1000 for rest 30% of time) |
| 10 | KCMBT 1.0 | > 15 Hours | | | | Not supported | | | |
| 11 | MSPKmerCounter0.1 | 3128 | 6 | 22.2 | 120.17 (Consistent) | 3124 | 9 | 5.7 | 340.49 (Consistent) |
| 12 | aTurtle 0.3 | Aborted (core dumped) | | | | Aborted (core dumped) | | | |
| 13 | GenomeTester4 | > 15 Hours | | | | Not supported | | | |
| 14 | BFCounter 1.0 | > 15 Hours | | | | > 15 Hours | | | |
| 15 | BFCounter 1.0 (gzip) | > 15 Hours | | | | > 15 Hours | | | |

If a program failed to complete the computation due to insufficient memory/disk space or within a stipulated time (15 hours), the respective entries in the above table are denoted by their failure messages. Entries in bold with * indicate best results, whereas entries in bold with italic type (including second lowest for CPU utilization) show average results. MSPKmerCounter has the highest error rates. MSPKmerCounter results and the compressed input results are not considered while highlighting the best and average results. For column ‘Disk’, the best and average results are highlighted by considering disk-based tools only. Abbreviations: sec = seconds, GB = gigabytes, MB = megabytes.

BFCounter was also unable to finish its work on the HS1 and HS2 datasets within 15 hours, and we killed the job due to system freezing. MSPKmerCounter failed on HS1 in phase 2 with the ‘OutOfMemoryError’ error.

In the following sections, we discuss the performance of different *k*-mer counting programs for each parameter separately.

*Runtime, memory and disk usage*

To present easily readable comparisons of all ten tools, we summarize Tables 4–8 (excluding the compressed input results) in Table 9. Table 9 illustrates the best and average programs for four measures: time, memory, disk and CPU utilization. The following conclusions can be drawn about each program from Tables 4–9.

Among all the programs under comparison, only DSK and KMC3 have generated accurate results (Appendix - Table A1 and Table A2, accuracy checked for the FV and DM datasets only) for both values of *k*, also within the stipulated time and without any system freeze issues for all seven datasets.

**Table 9** Summary of Tables 4–8

| Data-set ID | *k*-length | Time | | RAM | | Disk | | CPU Utilization (%) | |
|---|---|---|---|---|---|---|---|---|---|
| | | Highest | Lowest | Highest | Lowest | Highest | Lowest | Highest | Lowest |
| FV | 28 | KAnalyze | Gerbil | KCMBT | Gerbil | KAnalyze | Gerbil , KMC3 | Gerbil | GenomeTester4, aTurtle |
| | 55 | KAnalyze | KMC3 | Jellyfish | Gerbil | KAnalyze | Gerbil | Jellyfish | BFCounter, aTurtle |
| DM | 28 | BFCounter | KMC3 | GenomeTester4 | Gerbil | KAnalyze | Gerbil | Gerbil | GenomeTester4, aTurtle |
| | 55 | BFCounter | KMC3 | aTurtle | Gerbil | KAnalyze | KMC3 | KMC3 | BFCounter, aTurtle |
| MB | 28 | KAnalyze | Jellyfish | aTurtle | Gerbil | KAnalyze | Gerbil | Jellyfish | KCMBT, aTurtle |
| | 55 | KAnalyze | Jellyfish | Jellyfish | Gerbil | KAnalyze | Gerbil | Jellyfish | DSK |
| HS1 | 28 | DSK | KMC3 | DSK | Gerbil | DSK | Gerbil | Gerbil | DSK |
| | 55 | DSK | KMC3 | DSK | Gerbil, KMC3 | DSK | Gerbil | Gerbil | DSK |
| HS2 | 28 | DSK | Jellyfish | Jellyfish | Gerbil | DSK | Gerbil | Jellyfish | DSK |
| | 55 | Jellyfish | KMC3 | Jellyfish | Gerbil | DSK | Gerbil | Jellyfish | DSK |

CPU utilization will always remain lowest for single-threaded aTurtle. Hence, the second lowest entries are also mentioned in the ‘CPU Utilization (%)’ column.

DSK consistently uses a moderate amount of memory with reasonable speed. DSK is also robust, as it passes all the tests. KMC3 is often superior in terms of running time, but it is not memory frugal compared to its top competitor, Gerbil, though it is often not far from the best in terms of disk utilization.

Interestingly, Gerbil is consistently the most memory and disk frugal approach. For most of the datasets, the disk utilization of Gerbil is the lowest, but it is slower than KMC3 (The GPU implementation of Gerbil was not considered). Gerbil tries to reduce the utilization of disk and memory, revealing a result that is both memory and disk economical. It can be observed from Table 9 that the hash table–based counting is more hardware frugal than sorting-based counting. The more recent tools, specifically KMC3 and Gerbil, which implement the MSP approach along with a balance in the sizes of bins (signatures), show the best performance in memory requirements.

KAnalyze has much higher runtime and disk usage for most of the datasets and both values of *k* compared to the other disk-based approaches. KAnalyze needs more time for the merging step because its partitioning step is relatively straightforward.

In-memory approaches need no extra disk space, as these are completely memory-based. Among all in-memory algorithms, BFCounter utilizes the lowest memory because of the underlying memory-efficient Bloom filter. For the MB dataset, Jellyfish is the fastest in-memory algorithm and has the highest CPU utilization. But for the HS1 dataset, it could not complete its work within 15 hours. Among those datasets for which Jellyfish could generate complete results within the stipulated time, it has executed within a comparable time and with comparable memory requirements. Gerbil and Jellyfish often have the highest CPU utilization. Perez et al. [25] have reported similar behaviour of various *k*-mer counting tools for runtime and memory

usage.

*Performance for larger values of k*

GenomeTester4, KCMBT and aTurtle do not support large values of *k* (Table S1 of the Supplementary Material). For the NC and AT datasets, BFCounter failed with 'segmentation fault (core dumped)' error, whereas Jellyfish and KAnalyze could not complete counting of *k*-mers within 15 hours. MSPKmerCounter was unable to generate output for the NC dataset but succeeded in generating output for the AT dataset for all values of *k* (28, 40, 55, 65, 100, 125, 150, 175 and 200). Here, MSPKmerCounter is not included in the comparisons (Figure 1), owing to its high error rates. Only KMC3, DSK and Gerbil succeeded in generating results for different values of *k* (28, 40, 55, 65, 100, 125, 150, 175 and 200) for the NC and AT datasets within the stipulated time. Figure 1 shows the runtime and memory usage of these three tools for different values of *k*. These tools are highly optimized to support large values of *k*. The following conclusions can be drawn from Figure 1.

Gerbil is consistently the most memory frugal approach, but when *k* grows, Gerbil, DSK and KMC3 utilize almost the same amount of memory. KMC3 is faster than Gerbil on the NC dataset, but when *k* reaches higher values (150, 175 and 200), the runtime for both of them is similar. In the case of the AT dataset, KMC3 is faster than DSK and Gerbil. DSK uses almost the same memory as KMC3, but it is slower than KMC3 and Gerbil. KMC3 and Gerbil efficiently (minimum time and using moderate amounts of memory) support both small and large values of *k*.

**Figure 1.** Analysis of time (minutes) and memory (GB) utilization of *k*-mer counting algorithms for the NC and AT datasets for different values of *k* = 28, 40, 55, 65, 100, 125, 150, 175 and 200

*Scalability to varying sizes of datasets*

For datasets with shorter reads, such as the DM dataset, the runtime of each tool decreases with growing $k$ (Table 5). The human genome is a vast and important dataset. Only three programs, Gerbil, KMC3 and DSK, succeeded in generating results for large datasets (HS1 and HS2) within a reasonable time and without freezing the system (Table 7–8). These tools use disk-based approach, are more efficient in terms of time and memory utilization, and are scalable for large datasets (size > 200 GB) compared to tools based on in-memory approach. Jellyfish is memory efficient and the only in-memory algorithm that finished the counting of $k$-mers within a reasonable time for the HS2 dataset, consuming 58 GB and 48 GB memory for $k$ = 28 and 55, respectively (Table 8).

*The impact of compressed input*

The sequencing data is generally stored in a compressed format, mostly gzip, due to its large size. Programs that support compressed input have advantages, e.g., the I/O throughput is improved when hard drives are used, as the data are consumed faster by the algorithm. The data throughput is increased because the compressed data is directly read from the disk, thereby overcoming the cost of decompression of the file in memory.

Currently, only five tools support compressed input, namely, KMC3, Gerbil, DSK, KAnalyze and BFCounter. Reading the input from compressed form reduces the running time. A noticeable positive impact can be observed in the case of large datasets, i.e., HS1 and HS2, as shown in Tables 4–8. KMC3 is the fastest among the programs that support compressed input. For the HS1 and HS2 datasets, the gzipped input time of KMC3 and Gerbil is much less than the normalized input time, whereas the opposite trend can be seen in case of DSK. KMC3 on gzipped input is approximately 47% and 53% faster ($k$ = 28 and $k$ = 55 for the HS1 dataset, respectively) and 44% and 48% faster ($k$ = 28 and $k$ = 55, for the HS2 dataset) than on

normalized input. Gerbil on gzipped input is approximately 30% and 32% faster ($k$ = 28 and $k$ = 55 for the HS1 dataset, respectively) and approximately 26% and 27% faster ($k$ = 28 and $k$ = 55, for the HS2 dataset) than on normalized input. DSK on gzipped input is approximately 19% slower ($k$ = 28, for HS1) and 17% and 28% slower ($k$ = 28 and $k$ = 55, for HS2, respectively) than on normalized input (Table 7 and 8).

Regardless of input format (compressed or normalized), KMC3 is the fastest, whereas Gerbil is consistent in using the lowest memory and disk space. bzip2 has a high compression ratio, but its decompression is very slow. Thus, processing a bzip2 file is costlier than gzipped input (Tables 4–8). Only KMC3 and Gerbil support input data compressed with the bzip2 data compressor. They took a longer time for bzip2 than normalized input for the MB dataset (Table 6).

*Scalability on the number of threads*

All programs (except for aTurtle, as it is single threaded) are run with a different number of threads (1, 2, 4, 6, 8 and 12) to assess the CPU-related performance. The FV and MB datasets with $k$ = 28 are chosen so that all tools can complete their execution within the stipulated time. Figure 2 shows the scalability of these tools on the number of threads. It is not possible for any tool to achieve linear speedup. The following conclusions can be drawn from Figure 2.

**Figure 2.** Comparisons of scalability of different $k$-mer counting tools on the number of threads

Jellyfish (in-memory approach), implemented using a multithreaded lock-free hash table, has the highest speedup, i.e., 8.3 and 6.7, for the FV and MB datasets, respectively, for twelve threads. DSK and KMC3 have good speedup for the FV dataset, i.e., 7.2 and 7.1, respectively, for twelve threads. In the case of the MB dataset, the speedup achieved by each program is low and is in the range of 1-2 (except for Jellyfish). This result is due to increased threading overhead, as resource demand was increased by each thread owing to the large input size but having limited underlying resources. KCMBT is the fastest for a single thread on the MB dataset

(Table S8 Supplementary Material). However, for the four threads, KCMBT could not even complete the counting of *k*-mers in 15 hours for the same dataset owing to threading overheads. However, with the other tools, memory requirements are almost constant for an increasing number of threads. The overall speedup achieved by each program is not very high because *k*-mer counting is fundamentally an I/O-intensive task.

**Conclusions and future directions**

*k*-mer counting is used for solving many problems in bioinformatics. The main progress offered by high-throughput sequencing technologies is the ability to generate some billions of reads per instrument run. There is a need to continue the development of a system that realizes memory and time trade-off for *k*-mer counting in such large set of reads.

Many disk-based and in-memory approaches are available for *k*-mer counting that aim to generate results in minimum amount of time on large genomic data on a personal computer that has limited resources (memory, disk, etc.).

Of all the tools considered herein, KMC3, DSK and Gerbil are the most flexible and efficient programs, as they have higher speed with minimum memory requirements and better scalability to larger datasets. These three programs have automatic parameter selection for all the program parameters and are more robust. These programs support larger values of *k* (large *k* is an important use case for longer reads) and compressed input. Reading the input in compressed form improves the overall processing time. These tools are optimized to gain significant speedup by implementing parallelization using the available cores in the machine.

As sequencing technologies evolve, research endeavours must keep improving to develop a better system that influences the *k*-mer counting process with respect to the increase in the size of sequencing data.

## References


1. Reuter JA, Spacek DV, Snyder MP. High-Throughput Sequencing Technologies. Mol. Cell. 2015;58(4):586–97. doi:10.1016/j.molcel.2015.05.004
2. Molnar M, Ilie L. Correcting Illumina data. 2014;16:588–99. https://doi.org/10.1093/bib/bbu029.
3. Miller JR, Delcher AL, Koren S, Venter E, Walenz BP, Brownley A, et al. Aggressive assembly of pyrosequencing reads with mates. Bioinformatics. 2008;24:2818–24. doi:10.1093/bioinformatics/btn548
4. Myers EW, Sutton GG, Delcher AL, Dew IM, Fasulo DP, Flanigan MJ, Kravitz SA, Mobarry CM, Reinert KH, Remington KA, Anson EL. A whole-genome assembly of Drosophila. Science. 2000;287(5461):2196–204. doi:10.1126/science.287.5461.2196
5. Jaffe DB, Butler J, Gnerre S, Mauceli E, Lindblad-toh K, Mesirov JP, et al. Whole-Genome Sequence Assembly for Mammalian Genomes : Arachne 2. Genome Res. 2003;13(1):91–6. doi:10.1101/gr.828403
6. Miller JR, Koren S, Sutton G. Assembly algorithm for next-generation sequencing data. Genomics. 2010;95(6):315–27. doi:10.1016/j.ygeno.2010.03.001
7. Pevzner PA, Tang H, Waterman MS. An Eulerian path approach to DNA fragment assembly. Proc Natl Acad Sci U S A. 2001;98(17):9748–53. doi:10.1073/pnas.171285098
8. Zerbino DR, Birney E. Velvet: Algorithms for de novo short read assembly using de Bruijn graphs. Genome Res. 2008;18(5):821–9. doi:10.1101/gr.074492.107
9. Simpson JT, Wong K, Jackman SD, et al. ABySS : A parallel assembler for short read sequence data. Genome Res. 2009;19(6):1117–23. doi:10.1101/gr.089532.108.
10. Kelley DR, Schatz MC, Salzberg SL. Quake: quality-aware detection and correction of sequencing errors. Genome Biol. 2010;11:R116. https://doi.org/10.1186/gb-2010-11-11-r116
11. Shi H, Schmidt B, Liu W, Muller-Witting W. A parallel algorithm for error correction in high-throughput short-read data on CUDA-enabled graphics hardware. J Comput Biol. 2010;17(4):603–15. doi:10.1089/cmb.2009.0062
12. Liu Y, Schröder J, Schmidt B. Musket: A multistage k-mer spectrum-based error corrector for Illumina sequence data. Bioinformatics. 2013;29(3):308–15. doi:10.1093/bioinformatics/bts690
13. Medvedev P, Scott E, Kakaradov B, Pevzner P. Error correction of high-throughput sequencing datasets with non-uniform coverage. Bioinformatics. 2011;27(13):i137–41. doi:10.1093/bioinformatics/btr208
14. Salmela L, Schröder J. Correcting errors in short reads by multiple alignments. Bioinformatics. 2011;27(11):1455–61. https://doi.org/10.1093/bioinformatics/btr170
15. Edgar RC. MUSCLE: Multiple sequence alignment with high accuracy and high throughput. Nucleic Acids Res. 2004;32(5):1792–7. doi:10.1093/nar/gkh340
16. Liu B, Shi Y, Yuan J, Hu X, Zhang H, Li N, et al. Estimation of genomic characteristics by analyzing k-mer frequency in de novo genome projects. arXiv preprint arXiv:1308.2012. 2013; http://arxiv.org/abs/1308.2012
17. Price AL, Jones NC, Pevzner PA. De novo identification of repeat families in large genomes. Bioinformatics. 2005;21:i351–8. doi:10.1093/bioinformatics/bti1018
18. Li R, Ye J, Li S, Wang J, Han Y, Ye C, et al. ReAS: Recovery of ancestral sequences for transposable elements from the unassembled reads of a whole genome shotgun. PLoS Comput Biol. 2005;1:0313–21. https://doi.org/10.1371/journal.pcbi.0010043
19. Campagna D, Romualdi C, Vitulo N, Del Favero M, Lexa M, Cannata N, et al. RAP: A new computer program for de novo identification of repeated sequences in whole genomes. Bioinformatics. 2005;21:582–8. doi:10.1093/bioinformatics/bti039
20. Lefebvre A, Lecroq T, Dauchel H, Alexandre J. FORRepeats: Detects repeats on entire chromosomes and between genomes. Bioinformatics. 2003;19:319–26.
21. Healy J, Thomas EE, Schwartz JT, Wigler M. Annotating large genomes with exact word matches. Genome Res. 2003;13:2306–15. doi:10.1101/gr.1350803
22. Kurtz S, Narechania A, Stein JC, Ware D. A new method to compute K-mer frequencies and its application to annotate large repetitive plant genomes. BMC Genomics. 2008;9:517. https://doi.org/10.1186/1471-2164-9-517
23. Sindi SS, Hunt BR, Yorke JA. Duplication count distributions in DNA sequences. Phys Rev E. 2008;78. doi:10.1103/PhysRevE.78.061912
24. Pajuste F-D, Kaplinski L, Möls M, Puurand T, Lepamets M, Remm M. FastGT: from raw sequence reads to 30 million genotypes in less than an hour. Sci Rep. doi: https://doi.org/10.1101/060822
25. Pérez N, Gutierrez M, Vera N. Computational performance assessment of k-mer counting algorithms. J COMPUT BIOL. 2016;23(4):248–55. doi:10.1089/cmb.2015.0199

26. Roberts RJ, Carneiro MO, Schatz MC. The advantages of SMRT sequencing. Genome Biol. 2013; 14(6):6–9. https://doi.org/10.1186/gb-2013-14-7-405
27. Laehnemann D, Borkhardt A, McHardy AC. Denoising DNA deep sequencing data-high-throughput sequencing errors and their correction. BRIEF BIOINFORM. 2016;17:154–79. doi:10.1093/bib/bbv029
28. Sameith K, Roscito JG, Hiller M. Iterative error correction of long sequencing reads maximizes accuracy and improves contig assembly. BRIEF BIOINFORM. 2017;18:1–8. doi:10.1093/bib/bbw003
29. Xavier BB, Sabirova J, Pieter M, Hernalsteens JP, De Greve H, Goossens H, et al. Employing whole genome mapping for optimal de novo assembly of bacterial genomes. BMC Res Notes. 2014;7:484. https://doi.org/10.1186/1756-0500-7-484
30. Chikhi R, Medvedev P. Informed and automated k-mer size selection for genome assembly. Bioinformatics. 2014;30:31–7. https://doi.org/10.1093/bioinformatics/btt310
31. Erbert M, Rechner S, Muller-Hannemann M. Gerbil: A fast and memory-efficient k-mer counter with GPU-support. Algorithms Mol Biol. 2017;12:9 https://doi.org/10.1186/s13015-017-0097-9
32. Li Y, XifengYan. MSPKmerCounter: A Fast and Memory Efficient Approach for K-mer Counting. ArXiv e-prints. 2015;1–7. http://arxiv.org/abs/1505.06550
33. Rizk G, Lavenier D, Chikhi R. DSK: K-mer counting with very low memory usage. Bioinformatics. 2013;29:652–3. doi:10.1093/bioinformatics/btt020
34. Pandey P, Bender MA, Johnson R, Patro R. Squeakr: An Exact and Approximate k-mer Counting System. Bioinformatics. 2017;1–7. https://doi.org/10.1101/122077
35. Marçais G, Kingsford C. A fast, lock-free approach for efficient parallel counting of occurrences of k -mers. Bioinformatics. 2011;27(6):764–70. https://doi.org/10.1093/bioinformatics/btr011
36. Melsted P, Pritchard JK. Efficient counting of k-mers in DNA sequences using a bloom filter. BMC Bioinformatics. 2011;12:333. https://doi.org/10.1186/1471-2105-12-333
37. Kokot M, Długosz M, Deorowicz S. KMC 3: counting and manipulating k-mer statistics. Bioinformatics. 2017;2:1–3. http://arxiv.org/abs/1701.08022
38. Kaplinski L, Lepamets M, Remm M. GenomeTester4: a toolkit for performing basic set operations - union, intersection and complement on k-mer lists. Gigascience**.** 2015;4:58. https://doi.org/10.1186/s13742-015-0097-y
39. Deorowicz S, Kokot M, Grabowski S, Debudaj-grabysz A. KMC 2: fast and resource-frugal k-mer counting. Bioinformatics. 2015; 31(10):1569–76. doi:10.1093/bioinformatics/btv022
40. Audano P, Vannberg F. KAnalyze: A fast versatile pipelined K-mer toolkit. Bioinformatics. 2014;30:2070–2. doi:10.1093/bioinformatics/btu152
41. Deorowicz S, Debudaj-grabysz A, Grabowski S. Disk-based k-mer counting on a PC. BMC Bioinformatics. 2013;14:160. https://doi.org/10.1186/1471-2105-14-160
42. Roy RS, Bhattacharya D, Schliep A. Turtle : Identifying frequent k -mers with cache-efficient algorithms. Bioinformatics. 2014;14(30):1950–7. doi:10.1093/bioinformatics/btu132
43. Mamun A, Pal S, Rajasekaran S. KCMBT : a k -mer Counter based on Multiple Burst Trees. Bioinformatics. 2016;32:2783–90. https://doi.org/10.1093/bioinformatics/btw345
44. Salomon D. Data compression: the complete reference. Springer Science & Business Media; 2004. https://books.google.co.in/books?hl=en&lr=&id=PT1fcX321I4C&oi=fnd&pg=PR7&dq=%5B55%5D+Salomon+D+2004+Data+compression:+the+complete+reference.+Springer+Science+%26+Business+Media&ots=5iX8lXF0qJ&sig=_GM_InxCPFWXnbN3BZI_mACj8_k#v=onepage&q&f=false
45. Putze F, Sanders P, Singler J. Cache-, hash-, and space-efficient bloom filters. Journal of Experimental Algorithmics. 2009;14:4.4. doi:10.1145/1498698.1594230
46. Leiserson CE, Rivest RL, Stein C. Chapter 11: Hash Tables. Introd. to Algorithms. 2nd ed. Cambridge, Massachusetts London, England, McGraw-Hill; 2001. p. 221–45. Available from: http://web.ist.utl.pt/fabio.ferreira/material/asa/clrs.pdf
47. Purcell C, Harris T. Non-blocking hashtables with open addressing. Technical Report 639, University of Cambridge, Cambridge, UK. 2005;3724 LNCS:108–21.
48. Gao H, Groote JF, Hesselink WH. Almost wait-free resizable hashtables. IPDPS'04. 2004;0:1–37. doi:10.1109/IPDPS.2004.1302969
49. Shalev O, Shavit N. Split-ordered lists: Lock-free extensible hash tables. J. ACM (JACM). 2006;53:379–405. doi:10.1145/1147954.1147958
50. Mapleson D, Accinelli GG, Kettleborough G, Wright J, Clavijo BJ, Marc J. KAT : A K-mer Analysis Toolkit to quality control NGS datasets and genome assemblies. Bioinformatics. 2017;33(4):574–6. https://doi.org/10.1093/bioinformatics/btw663

51. Bloom BH. Space/Time Trade-offs in Hash Coding with Allowable Errors. COMMUN ACM. 1970;13(7):422–6. doi:10.1145/362686.362692
52. Randall D, Guerrieri A, Jin W. Bloom Filters and Hashing. CS 6550 Design and Analysis of Algorithms. 2006;1–7.
53. Pandey P, Bender MA, Johnson R, Patro R. A General-Purpose Counting Filter. Proc. 2017 ACM Int. Conf. Manag. Data - SIGMOD ’17. 2017;775–87. doi:10.1145/3035918.3035963
54. Abouelhoda MI, Kurtz S, Ohlebusch E. Replacing suffix trees with enhanced suffix arrays. J. Discret. Algorithms. 2004;2:53–86. https://doi.org/10.1016/S1570-8667(03)00065-0
55. Heinz S, Zobel J, Williams HE. Burst tries: a fast, efficient data structure for string keys. ACM T INFORM SYST. 2002;20:192–223. doi:10.1145/506309.506312
56. Li Y, Kamousi P, Han F, Yang S, Yan X, Suri S. Memory Efficient Minimum Substring Partitioning. 39th Int. Conf. Very Large Data Bases. 2013;6:169–80. doi:10.14778/2535569.2448951
57. Kokot M, Deorowicz S, Debudaj-Grabysz A. Sorting Data on Ultra-Large Scale with RADULS. New Incarnation of Radix Sort. arXiv:1612.02557. 2016; http://arxiv.org/abs/1612.02557
58. Melsted P, Halldórsson B V. KmerStream: Streaming algorithms for k-mer abundance estimation. Bioinformatics. 2014;30:3541–7. doi:10.1093/bioinformatics/btu713
59. Mohamadi H, Khan H, Birol I. ntCard: A streaming algorithm for cardinality estimation in genomics data. Bioinformatics. 2017;33:1324–30. doi:10.1093/bioinformatics/btw832
60. Junior LC, Brown CT. Efficient cardinality estimation for k-mers in large DNA sequencing data sets. bioRxiv. 2016;1–5. https://doi.org/10.1101/056846
61. Crusoe MR, Alameldin HF, Awad S, Boucher E, Caldwell A, Cartwright R, et al. The khmer software package: enabling efficient nucleotide sequence analysis. F1000Research. 2015;4:900. doi: 10.12688/f1000research.6924.1

Figure 1

Click here to access/download;Figure;Figure 1.pdf

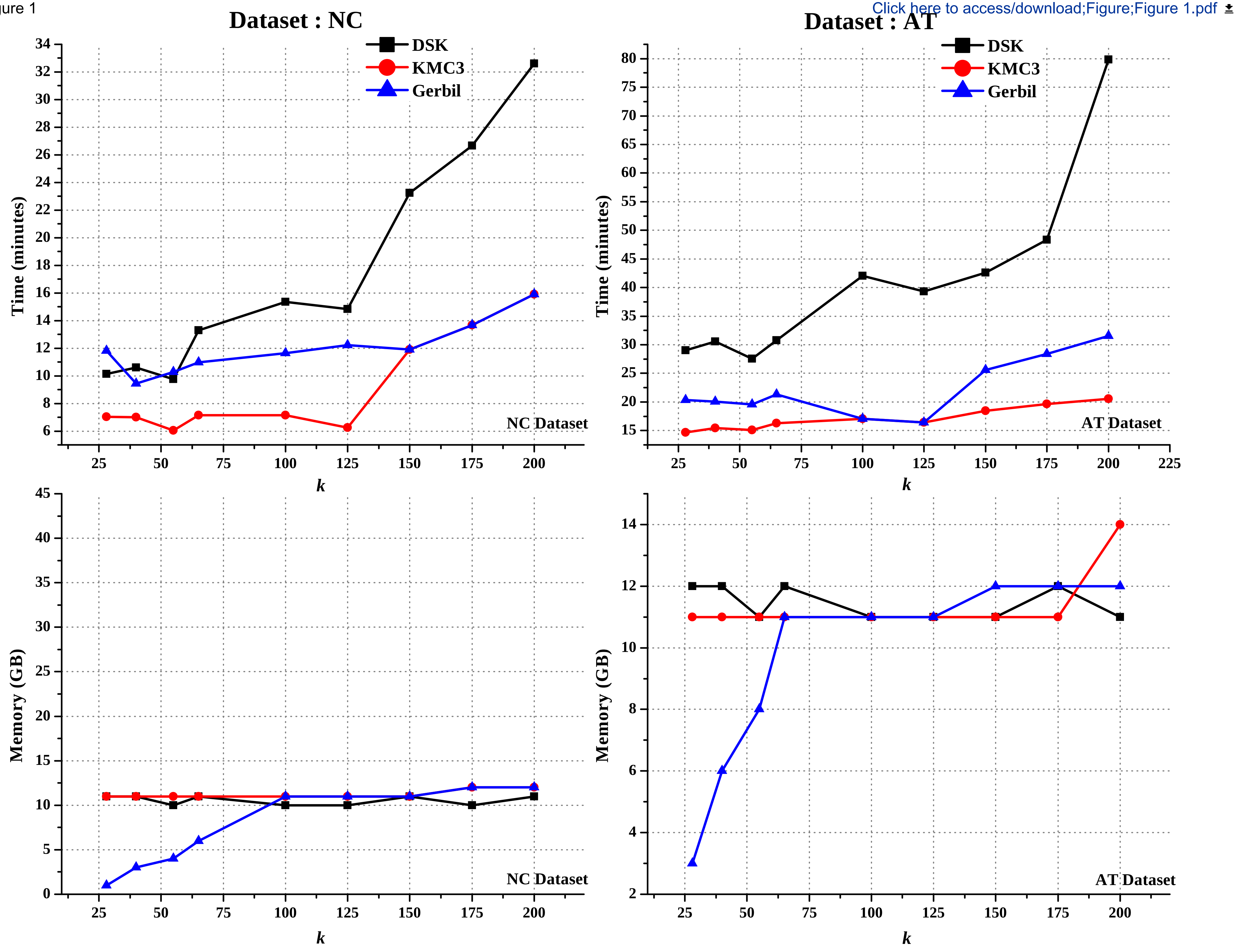

Figure 2

Click here to access/download;Figure;Figure 2.pdf

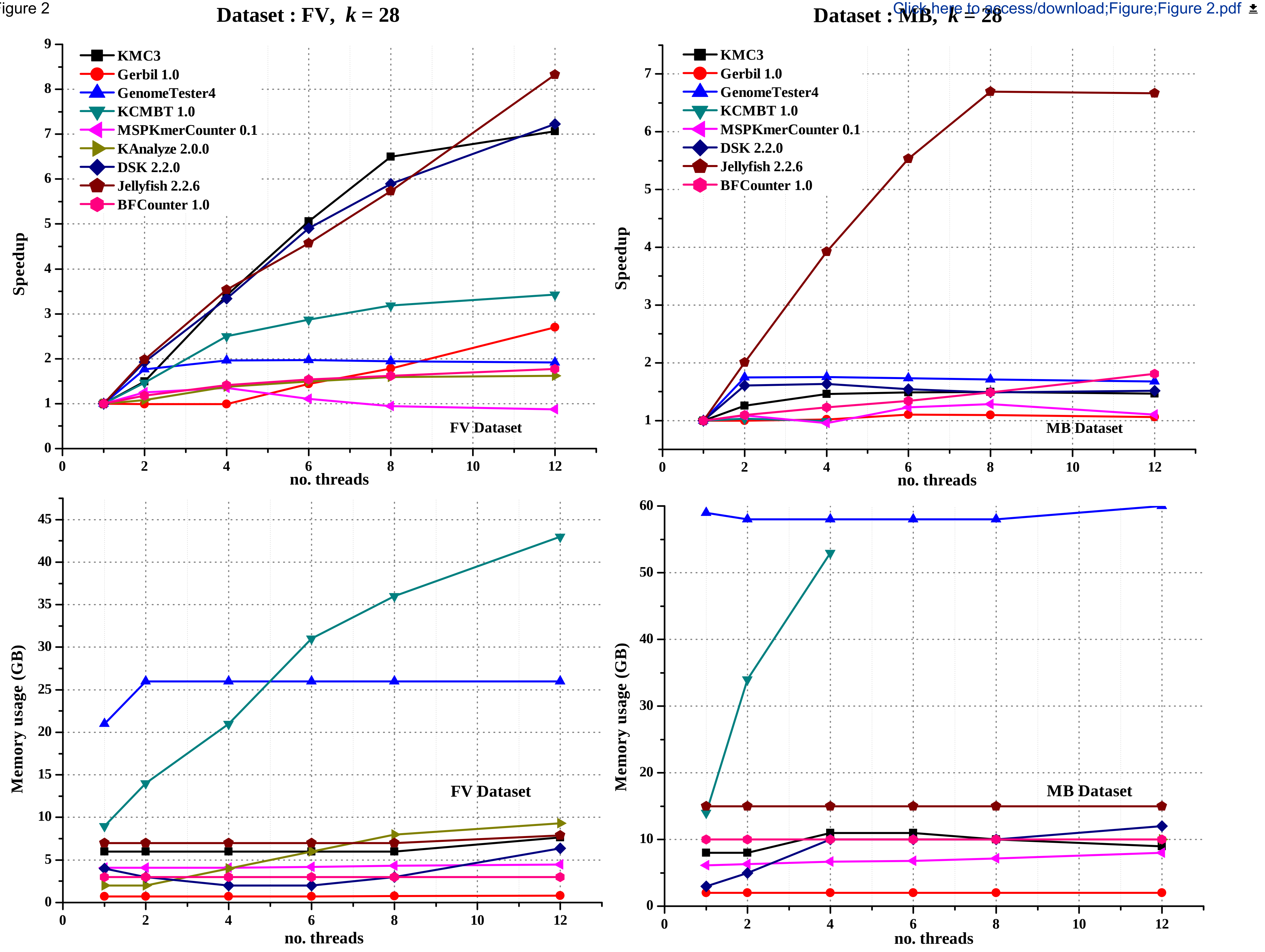

Click here to access/download
**Supplementary Material**
Appendix_revised_2.doc

Click here to access/download
**Supplementary Material**
Supplimentary_revised_2.doc
