# Supplementary material for: A benchmark study of k-mer counting methods for high-throughput sequencing
Source: Gigascience. 2018 Oct 22;7(12):giy125. doi: 10.1093/gigascience/giy125 (PMC6280066; doi:10.1093/gigascience/giy125)
Supplement: GIGA-D-17-00245_Revision_4.pdf [file giy125_giga-d-17-00245_revision_4.pdf]

GigaScience

A benchmark study of k-mer counting methods for high-throughput sequencing

--Manuscript Draft--

**Manuscript Number:**	GIGA-D-17-00245R4
**Full Title:**	A benchmark study of k-mer counting methods for high-throughput sequencing
**Article Type:**	Review
**Funding Information:**	
**Abstract:**	The rapid development of high-through sequencing technologies means that hundreds of gigabytes of sequencing data can be produced in a single study. Many bioinformatics tools require counts of substrings of length k in DNA/RNA sequencing reads obtained for applications such as genome and transcriptome assembly, error correction, multiple sequence alignment, and repeat detection. Recently, several techniques have been developed to count k-mers in large sequencing datasets, with a trade-off between the time and memory required to perform this function. We assessed several k-mer counting programs and evaluated their relative performance, primarily on the basis of runtime and memory usage. We also considered additional parameters, such as disk usage, accuracy, and parallelism, and the impact of compressed input, performance in terms of counting large k values, and the scalability of the application to larger datasets. We make specific recommendations for the set-up of a current state-of-the-art program, and suggestions for further development.
**Corresponding Author:**	Swati Chandrakant Manekar, M.Tech.<br>Visvesvaraya National Institute of Technology<br>Nagpur, Maharashtra INDIA
**Corresponding Author Secondary Information:**	
**Corresponding Author's Institution:**	Visvesvaraya National Institute of Technology
**Corresponding Author's Secondary Institution:**	
**First Author:**	Swati Chandrakant Manekar, M.Tech.
**First Author Secondary Information:**	
**Order of Authors:**	Swati Chandrakant Manekar, M.Tech.
	Shailesh Sathe, Ph.D.
**Order of Authors Secondary Information:**	
**Response to Reviewers:**	Dear editor,<br><br>We appreciate the time and effort spent by the copy editor for careful copy-editing and proofreading the manuscript. Many thanks for care and attention to detail by the most impressive copy editor and the amount of work you put into this manuscript which made it a much better review article. We have incorporated all the comments and suggestions made by the copy editor and accepted all the track changes. We address the comments individually with suitable responses as follows,<br><br>Comment [SE1]: Do you have an ORCID too? Is it 0000-0002-5297-2018<br>>><br>Yes and it is 0000-0002-5297-2018<br>>><br><br>Comment [LM2]: Please insert the ORCID of the second author.<br>>><br>Inserted the ORCID of the second author in the manuscript as follows,<br>ORCID IDs: Shailesh R. Sathe: 0000-0002-5297-2018<br>>>

Powered by Editorial Manager® and ProduXion Manager® from Aries Systems Corporation

Comment [LM3]: Possibly not needed in the Abstract – delete?
>>
The line is deleted from the Abstract.
All k-mer counting programs evaluated in this article are freely available and can be downloaded from their respective websites.
>>

Comment [LM4]: Disjointed?
>>
Disjoint is correct as we have checked from the source document for 'Minimum Substring Partitioning' (MSPKmerCounter). Hence we have kept 'Disjoint' as it is (Line No. 15, Page No.11).The referred line from manuscript of MSPKmerCounter is as follows,

'MSP breaks short reads into multiple disjoint partitions such that each partition can be loaded into memory and processed individually.'
>>

Comment [LM5]: To me, it makes more sense to describe the tools you are going to test first, and then describe the datasets you are going to run on them, so I have moved some paragraphs around here and amended the subheading slightly.
>>
We agree to the point raised herein and hence accepted the format changes.
>>

Comment [LM6]: I have deleted some of the detail in this paragraph as I felt it was too much detail about something that is not discussed in the paper!
>>
We agree and accepted the format changes. The references are changed accordingly. [61(khmer library)] is now removed from the manuscript.
>>

Comment [LM7]: The URL should be listed in the references list. Please update the reference numbering accordingly.
>>
The URL is now listed in the references list and the reference numbering is updated accordingly.
[61]
https://gist.github.com/netj/526585/36515c55a3b25232bddfdd51e43cadc1a5f296de.
>>

Comments [SE8] [SE9]: Can we get a copy of this?
>>
The scripts are uploaded (as supplementary files) with the revised documents. The script 'cpu_disk_util.sh' which captures the disk usage and % CPU utilization has been written by referring the script by Jaeho Shin, licensed under the Apache License, Version 2.0 (the "License"), http://www.apache.org/licenses/LICENSE-2.0. The script 'dsk_max_cpu_avg.sh' gives the disk usage values (peak and minimum) and average over the %CPU utilizations captured by 'cpu_disk_util.sh'. To calculate the maximum disk used by the program, subtract the three values given by the script 'dsk_max_cpu_avg.sh', as shown below,
The maximum disk used by the program = Peak disk used - Disk used before the start of program execution - The file size of output file generated by the tool.
>>

Comments [LM10] [LM11] [LM12] [LM13] [LM14]: This should be expressed as GB, in line with the other figures in this column.
>>
We agree & now expressed figures which were in MBs in GBs as follows,
Table 4: Gerbil, RAM , k=25 : 848 MB* => 0.83*
Table 4: Gerbil(gzip), RAM , k=25 : 841 MB => 0.82
Table 5: KMC3, Disk , k=50 : 614.4 MB* => 0.6*
Table 5: Gerbil, RAM , k=25 : 827 MB* => 0.81*, k=50 : 826 MB* => 0.81*
Table 5: Gerbil(gzip), RAM , k=25 : 837 MB => 0.82, k=50 : 834 MB => 0.81

Powered by Editorial Manager® and ProduXion Manager® from Aries Systems Corporation

	Hence removed MB = megabytes from Table footnotes.<br><br>Comment [SE15]: Please include abbreviations here<br>>><br>RAM: Random Access Memory; NGS: Next-generation Sequencing; SAC: Sort-and-compact; CQF: Counting Quotient Filter; MSP: Minimum Substring Partitioning; FV: F. Vesca; DM: D. Melanogaster; MB: M. Balbisiana; HS1: H. sapiens 1; HS2: H. sapiens 2; NC: N. Crassa; AT: A. thaliana<br>>><br><br>Comment [LM16]: Please ensure that the references are formatted according to GigaScience's preferred style.<br>>><br>We have ensured that the references are formatted according to GigaScience's preferred style.<br>>>
Additional Information:	
Question	Response
Are you submitting this manuscript to a special series or article collection?	No
**Experimental design and statistics**<br><br>Full details of the experimental design and statistical methods used should be given in the Methods section, as detailed in our Minimum Standards Reporting Checklist. Information essential to interpreting the data presented should be made available in the figure legends.<br><br>Have you included all the information requested in your manuscript?	Yes
**Resources**<br><br>A description of all resources used, including antibodies, cell lines, animals and software tools, with enough information to allow them to be uniquely identified, should be included in the Methods section. Authors are strongly encouraged to cite Research Resource Identifiers (RRIDs) for antibodies, model organisms and tools, where possible.<br><br>Have you included the information requested as detailed in our Minimum Standards Reporting Checklist?	Yes

**Availability of data and materials**<br><br>All datasets and code on which the conclusions of the paper rely must be either included in your submission or deposited in publicly available repositories (where available and ethically appropriate), referencing such data using a unique identifier in the references and in the "Availability of Data and Materials" section of your manuscript.<br><br>Have you have met the above requirement as detailed in our Minimum Standards Reporting Checklist?	Yes

Manuscript
Click here to access/download;Manuscript;Manuscript_revisied_4.doc
Click here to view linked References

A benchmark study of *k*-mer counting methods for high-throughput sequencing

Swati C. Manekar[1*] and Shailesh R. Sathe[1]

[1]Department of Computer Science and Engineering, Visvesvaraya National Institute of Technology,

Nagpur 440 010, India

*corresponding author, Email: swati.manekar@gmail.com.

ORCID IDs: Swati C. Manekar: 0000-0003-4682-7065;

ORCID IDs: Shailesh R. Sathe: 0000-0002-5297-2018;

Abstract

The rapid development of high-through sequencing technologies means that hundreds of gigabytes of sequencing data can be produced in a single study. Many bioinformatics tools require counts of substrings of length *k* in DNA/RNA sequencing reads obtained for applications such as genome and transcriptome assembly, error correction, multiple sequence alignment, and repeat detection. Recently, several techniques have been developed to count *k*-mers in large sequencing datasets, with a trade-off between the time and memory required to perform this function. We assessed several *k*-mer counting programs and evaluated their relative performance, primarily on the basis of runtime and memory usage. We also considered additional parameters, such as disk usage, accuracy, and parallelism, and the impact of compressed input, performance in terms of counting large *k* values, and the scalability of the application to larger datasets. We

make specific recommendations for the set-up of a current state-of-the-art program, and suggestions for further development.

Keywords: *k*-mer counting; high-throughput sequencing; disk-based counting; in-memory counting; hash table; sorting

Introduction

k-mer counting is an important step in many bioinformatics applications that are used to analyze sequencing data. Recently, several tools and techniques have been developed to count the frequency of *k*-length substrings (*k*-mers) in reads generated from high-throughput sequencing [1]. *k*-mer counting involves counting the number of substrings that have length *k* in a string *S*, or a set of strings, where *k* is a positive integer.

Let ∑= {*A, C, G, T, N*} denote the alphabet of DNA nucleotide sequences, where *N* denotes an undetermined character. A read *r* is a sequence of nucleotides over the alphabet ∑. In a sequence dataset, different reads can contain the same sequence of nucleotides. Let *R* denote a dataset having *n* reads, such that $R = \{r_i;\ 1 \leq i \leq n\}$. Consider an example dataset *R* containing three reads each of length 6, *R* = {*ACGTTA*, *ACGTTA, ACGTTT*}, having two sequences {*ACGTTA*, *ACGTTT*}. For *k* = 4, there are nine 4-mers (three in each read): {*ACGT*, *CGTT*, *GTTA*, *ACGT*, *CGTT*, *GTTA*, *ACGT*, *CGTT*, *GTTT*}. On counting, four unique 4-mers are obtained, which can be represented along with their counts in a tab-delimited format, e.g., {*ACGT* 3, *CGTT* 3, *GTTA* 2, *GTTT* 1} [2].

k-mer counting has applications in genome assembly, e.g., using the overlap layout consensus approach [3–5] or the de Bruijn graph approach [6–9]. Errors in sequencing reads are corrected

to improve the quality of genome assemblies. Error correction based on the *k*-mer spectrum approach [10–13], or multiple sequence alignment approach [14], relies on counting and keeping track of *k*-mers. *k*-mer counting is also used (for fast distance estimation) to create multiple protein sequence alignments [15]. In *de novo* genome projects, genomic characteristics such as genome size, repeat structure, and rate of heterozygosity are estimated by analyzing the *k*-mer frequency distribution in a sequencing dataset [16]. Statistical analysis can reveal the high-frequency *k*-mers in a given dataset, which are used as 'seeds' to build a set of repeat families [17]. ReAS [18] also uses these high-frequency *k*-mers (obtained from *k*-mer repeat libraries) in the reconstruction of transposable elements (TEs). Identifying repeated sequences is a main step in genome analysis and annotation. *De novo* repeat identification techniques such as RAP [19], FORRepeats [20], and that described by Healy et al. [21], use *k*-mer occurrences to find candidate regions (i.e. to identify repeated regions). Tallymer [22] also uses *k*-mer frequencies to annotate repetitive plant genomes. Quantitative features of complex repetitive DNA have been studied in several genomes by determining the distribution of frequencies of long *k*-mers ($20 \leq k \leq 100$) [23]. *k*-mer counts are also used to infer the genotypes of known variants [24].

Although *k*-mer counting is simple and straightforward, it becomes challenging when billions of reads generated by next-generation sequencing (NGS) techniques must be processed using reasonable amounts of memory and in minimal time. A naive approach for *k*-mer counting is to use a dictionary, with *k*-mers as keys and their counts as values. However, when there are billions of such input reads, computer memory is often overwhelmed. Approaches to *k*-mer counting proposed so far have mainly targeted memory-efficient and time-efficient solutions. One way to achieve memory efficiency is to represent the string data using unsigned integers. Disks are always cheaper than memory. Therefore, many researchers have focused on using the

disk-based/external memory/out-of-core approach, as opposed to the in-memory/internal memory approach, to reduce memory usage.

Here, we review and comparatively evaluate *k*-mer counting approaches for high-throughput sequencing data. The main aim is to provide a general set of benchmarks and assessment metrics of some popular *k*-mer counters, but we also cover experimental analysis of *k*-mer counting tools to provide thorough insight for beginners and consultants alike. Perez et al. [25] studied various *k*-mer counting tools for two values of *k*, i.e., 31 and 55, on a single dataset. Building on this, we evaluate the performance of several of the latest and competitive tools on different datasets of varying sizes, primarily focusing on runtime and memory usage. However, we also consider several other parameters: (i) scalability to larger values of *k*; (ii) scalability to larger datasets; (iii) the impact (on runtime, memory, disk and central processing unit (CPU) utilization) of compressed inputs, i.e., gzip and bzip2 (multiple compressed FASTA/FASTQ input files); (iv) scaling properties (speedup) with respect to thread number; and (v) accuracy and (vi) maximum temporary disk usage. Scalability is measured in terms of runtime, memory, disk usage, and CPU utilization.

Time, CPU, and memory, are bounded (limited) resources, whereas the disk can be considered a plentiful resource. Disk-based approaches may additionally use hundreds of gigabytes of disk space for large datasets, such as the human genome. Hence, we record the maximum disk utilization of all disk-based approaches. Disk-based approaches achieve very high efficiency with marginal increase in I/O costs.

Advancements in NGS technologies mean that long reads are generated in bioinformatics. Among other advantages, such long reads are excellent for resolving complex RNA-splicing patterns from cDNA libraries [26], resolving repetition in genome assemblies, and to facilitate

better resolution of structural variants present in DNA samples and genomic repeat content [26, 27]. However, longer Illumina reads suffer from lower accuracy [27]. Large *k* values (up to 200) help improve the accuracy of longer Illumina reads (particularly of repeat-overlapping reads) and contig assemblies [28]. Empirically, the best assemblies (i.e., those without misassembly errors) and the highest N50 (only when there is sufficiently high coverage) are obtained at an optimal choice of *k*, which seems to be larger values of *k* [29, 30]. Hence, we evaluate the performance of different *k*-mer counting tools at large *k* values.

Overview of *k*-mer counting approaches

k-mer counting tools can be categorized based on the approach and data structures they use, as shown in Table 1. Comprehensive information about each *k*-mer counting tool is given in Supplementary Table S1.

Table 1 Ontology of *k*-mer counting approaches

Approach for *k*-mer counting	Disk-based	In-memory
Hash table	Gerbil [31], MSPKmerCounter [32], DSK [33]	Squeakr [34], Jellyfish [35], BFCounter [36]
Sorting	KMC3 [37], GenomeTester4 [38], KMC2 [39], KAnalyze [40], KMC1 [41]	Turtle [42]
Burst tries	-	KCMBT [43]

Enhanced suffix array	-	Tallymer [22]

k-mer counting using the sorting approach

This approach works by sorting all *k*-mers extracted from each read. Thus, *k*-mer frequencies can be easily counted because, after sorting, repeating *k*-mers lay at adjacent positions in the sorted list.

GenomeTester4 (GListMaker) [38] uses the sorting approach and works as follows: (i) in the reading phase, temporary arrays are used to gather all *k*-mers from the input file; and (ii) *k*-mers stored in these arrays are first sorted and then counted during the collation phase. Counting results in temporary arrays (tables), which are then merged to produce the final *k*-mer count list. GenomeTester4 uses multiple threads to speed up *k*-mer counting.

Turtle [42] uses a novel sorting and compaction (SAC)-based algorithm, which is memory-efficient. Turtle works as follows: *k*-mers are added to a large array up to a certain point, each with a count of one. This array is then sorted, identical *k*-mers are compacted, and their counts are added up in the compaction step. The compaction process frees up space in the array, which is used for a new set of *k*-mers. The SAC approach is then applied to existing and newly added *k*-mers and is performed iteratively until all *k*-mers are counted. The compaction process is similar to run-length encoding [44]. Turtle has three implementations, scTurtle, cTurtle, and aTurtle, which vary in their outputs. scTurtle has false positive and outputs only *k*-mers with frequency > 1. cTurtle accepts small rates of false positives and false negatives and gives only *k*-mers with frequency > 1 without any counts. cTurtle gives an approximate set of frequent *k*-mers by using a counting Bloom filter. aTurtle provides *k*-mers of all frequencies with their counts. Although

multithreaded, cTurtle and scTurtle do not count perfectly, whereas single-threaded aTurtle does. Hence, in this study, aTurtle is considered.

scTurtle [42] uses a pattern block Bloom filter to remove all single occurrence (spurious) *k*-mers. The remaining *k*-mers are then counted using the SAC approach. Pattern block Bloom filter [45] is a cache-friendly variant of the Bloom filter, which has a very small cache miss ratio.

k-mer counting using a hash table

A hash table [46] can be used to count *k*-mers, in which *k*-mers are stored as keys, and their counts are stored as values. Jellyfish [35] uses a lock-free hash table to allow parallel insertion of *k*-mers and frequency updates by multiple threads using a CAS (compare-and-swap) assembly instruction [47]. The CAS operation detects simultaneous access to a shared memory location in the multithreaded environment. The entire memory capacity is used to store the hash table. Once the hash table is full, it is written to disk instead of doubling its size in the memory, and intermediate *k*-mer counts are then merged [48, 49]. Jellyfish works as follows: whenever a new *k*-mer appears, the program obtains its key and searches for it in the hash table. If it exists, the frequency count increases by one. If not, this *k*-mer is inserted (with frequency set to 1) into the hash table using the reprobe strategy. If a collision occurs, it is resolved using a quadratic probing (open addressing) technique [46].

Jellyfish 2 is a more efficient version of Jellyfish, which has an additional Bloom filter-based mode to remove all singleton *k*-mers (i.e., *k*-mers occurring only once in the dataset). KAT [50] counts *k*-mers using a modified version of the Jellyfish 2 library.

k-mer counting using a Bloom filter and its variants, and counting quotient filter

A majority of any genomic dataset is made up of single frequency *k*-mers, which are mainly attributed to sequencing errors. A Bloom filter [51] is a probabilistic data structure used for dynamic membership query lookup, which can implicitly store all *k*-mers. It is used to filter out singleton *k*-mers. The frequency of every non-singleton *k*-mer can then be counted using any of the approaches in Table 1. The Bloom filter returns some false-positive membership query results, which may lead to *k*-mer miscounts. However, with a reasonable choice of hash functions, the false-positive rate can be minimized to an acceptable degree [52]. It also requires very little memory (i.e., enough to store a bit-vector [52]), reducing the overall memory requirement.

Using a similar concept, BFCounter [36] filters out singleton *k*-mers and uses a hash table to store and count non-singleton *k*-mers. Some false-positive singleton *k*-mers are erroneously included in the hash table, leading to miscounts, but BFCounter generates correct results by re-iterating over the sequence reads.

Squeakr [34] is an in-memory approach for counting *k*-mers both approximately and exactly. It uses a counting filter data structure (counting quotient filter (CQF) [53]) to store *k*-mer counts. *k*-mers are hashed using a one-way hash function, and these hashes are stored in a counting filter. The single-phase algorithm is based on the following ideas: multiple threads read input data from the disk in chunks and simultaneously insert *k*-mers into a global shared thread safe CQF for *k*-mer counting. Each thread maintains a local CQF to temporarily hold the *k*-mer counts to reduce waiting time, while acquiring a lock in the global CQF (it is hardest to acquire the lock when repetitive *k*-mers are present in the dataset). Once the local CQF is full, it dumps counted *k*-mers into the global CQF before processing a new set of *k*-mers. For this benchmark study, we have

not considered Squeakr (exact), which counts the frequency of each *k*-mer exactly using an invertible hash function, because its code is not yet available.

Enhanced suffix array-based counting

Tallymer [22] is an in-memory approach, which uses a longest common prefix (lcp)-interval tree constructed from an enhanced suffix array [54] to count *k*-mers. The lcp-interval tree implicitly stores the number of occurrences of all substrings of *s*' (reads are concatenated into a string *s*' with a unique termination symbol ($\$_i$) appended to each read). The algorithm has two steps: (i) the divide step splits sequence *s*' into smaller distinct partitions, and the *k*-mers in each partition are then counted using the lcp-interval tree; and (ii) the merging step, in which a final count is generated by merging the counts generated from all distinct partitions using the sequence *s*'. Suffix array construction of a string is expensive in terms of computation and memory requirement. Suffix array size increases linearly with the length of string *s*'.

Trie data structure-based k-mer counting

KCMBT [43] is an in-memory approach using burst trie, a variant form of a trie [55]. Burst tries efficiently manage large string sets in the memory, and maintain strings in a sorted or nearly sorted order. KCMBT uses extended *k*-mers, similar to KMC2. Extended *k*-mers (($k + x$)-mers for $x > 0$) are substrings of length greater than *k*, and were introduced by KMC2.

The KCMBT algorithm has three phases. Firstly, ($k + x$)-mers are generated from the input reads and inserted into the corresponding trees. To do this, a fixed-length container is initially maintained for each tree. When a container is full, it bursts, and is replaced by a new trie node and a set of child containers. These child containers partition ($k + x$)-mers of the original

container among themselves by taking a one-symbol prefix (matching A/C/G/T) of the ($k + x$)-mers (x is chosen empirically to be $0 \leq x \leq 3$ for better performance). Secondly, each ($k + x$)-mer tree ((k + 1)-mer, (k + 2)-mer and (k + 3)-mer trees) is traversed to count all unique ($k + x$)-mers [42], which are then broken into *k*-mers to obtain a count of constituent *k*-mers. *k*-mers are then inserted into *k*-mer trees. Finally, *k*-mer trees are traversed to produce counts of all unique *k*-mers, and these, along with their counts, are written to disk. Because of ($k + x$)-mers, the inserted number of *k*-mers, and the time required for traversal in the last phase are much reduced, leading to faster computation. Thousands of trees with smaller heights are generated to reduce the overall insertion and traversal time required to count huge numbers of *k*-mers. Burst trie has a very rapid search time, but its size becomes large for large volumes of sequencing data.

Disk-based *k*-mer counting

The disk-based approach to *k*-mer counting has a much lower memory requirement than in-memory approaches, and was designed to make it possible to count *k*-mers in large genomic datasets, such as a human genome dataset, on commodity hardware. Memory usage can be greatly reduced using a disk, because *k*-mers are processed in chunks and stored on disk.

DSK [33] is a disk-based approach to counting *k*-mers using very little memory and disk space. To achieve this, DSK calculates the number of partitions needed to bring data in parts from disk to memory, depending on (i) the total bits required to store the *k*-mers, and (ii) the disk size available. DSK calculates the number of iterations needed to read the entire set of input in parts, depending on (i) the total number of bits required to represent the entire set of *k*-mers, (ii) the memory size required to hold the hash table, (iii) the number of partitions, and (iv) the load factor for which hash table gives the best performance. Each *k*-mer is distributed to one of

multiple disk-stored partitions, depending on its hash value, and an iteration number. *k*-mers are counted by loading a partition into the memory one at a time, using hash tables in multiple iterations. The partition strategy means that DSK efficiently addresses memory constraints, but it may result in a high I/O cost.

KAnalyze [40] uses a sorting-based approach to count *k*-mers. The algorithm has two phases. Firstly, *k*-mers are filled into a temporary array of predefined size. Once the array is full, *k*-mers are sorted, counted, and written to disk so that space becomes available to count the next incoming chunk of *k*-mers. The process is repeated until all the *k*-mers are processed. In the second phase, count files are loaded from disk to memory, and are merged in multiple steps to generate final *k*-mer counts.

Approaches using the concept of super k-mer: minimizers and signatures

The disk-based compression technique MSP (Minimum Substring Partitioning) [56] is used to further reduce memory requirements and I/O operations. In this technique, input reads are broken into multiple disjoint partitions.

The adjacency relationship between each pair of *k*-mers means that *k*-mers carry highly redundant data. With MSP, if consecutive *k*-mers share the same lexicographical minimum substring *s*, then they are stored as one substring of length greater than *k*. This substring is called a ‘super *k*-mer’ and is stored in a disk partition corresponding to the lexicographical minimum substring *s*, where *s* is termed a ‘minimizer’. Larger numbers of consecutive *k*-mers sharing the same minimum substring *s* give a better compression ratio, which ultimately reduces I/O overhead and storage space.

MSPKmerCounter [32] is the first tool to implement MSP for *k*-mer counting. It works as follows: (i) reads are decomposed into super *k*-mers and distributed to disk partitions (bins) identified by canonical minimizers. The storage of super *k*-mers with same canonical minimizer in the same partition assures that all the occurrences of the same *k*-mer belong to the same partition, thus eliminating the need to merge the counts of each partition. These smaller partitions are easily accommodated into the memory and are processed independently. (ii) Once the partitions are ready, all super *k*-mers are broken into *k*-mers using simple bit shift operations. (iii) Finally, *k*-mers are counted using hash tables, and counts are stored on disk.

KMC2 [39] is another disk-based approach that is similar to the MSP employed in MSPKmerCounter. Here, the minimizer is refined to signatures, which significantly reduce the overall memory requirements and temporary disk space. Canonical minimizers are used as signatures with the following three prerequisites: canonical minimizers (1) do not begin with prefix *AAA*, (2) do not begin with prefix *ACA*, and (3) do not contain *AA* anywhere apart from at the beginning. The KMC2 algorithm has two major phases: distribution and sorting. The distribution phase is similar to that of MSPKmerCounter, the only difference being that super *k*-mers are distributed to different temporary files (bins) based on signatures instead of minimizers. In the sorting phase, bins are processed by fetching them into the memory. For every such bin, extended *k*-mers, i.e., $(k + x)$-mers, are extracted from super *k*-mers, and a radix sort is applied. *k*-mer statistics are then collected from these sorted $(k + x)$-mers, and stored on disk.

KMC3 is an extension of the KMC2 approach, with few improvements: (i) efficient input file reading to achieve a better I/O subsystem, (ii) a memory-efficient way of assigning signatures to bins, and (iii) an efficient sorting approach [57], rather than using a radix sort, for larger values of *k*.

Gerbil [31] uses a hashing approach to *k*-mer counting that is similar to DSK. The algorithm has two major phases. The first is slightly advanced but similar to the KMC2 distribution phase in which the hash values (obtained using a partHash [31] function) of *k*-mers (extracted from super *k*-mers) are used to ensure that multiple occurrences of the same *k*-mer are assigned to the same thread. In the second phase, super *k*-mers stored in the temporary files are sequentially re-read from the working disk. All *k*-mers are extracted from the super *k*-mers, and then counted using a hash table. Collisions are resolved using quadratic hashing. Each thread counts the assigned *k*-mers using its hash table, and these are then written into an output file. The algorithm makes optimal use of the hardware by concurrently running multiple threads. To achieve memory efficiency, hash table size is estimated using a simple linear model. In its GPU implementation, the second phase is performed on the GPU side with proper load balancing between GPU and CPU.

Tools assessed, benchmark datasets used, and evaluation methodology

Our study evaluated the most recently available versions of: KMC3, Gerbil (version 1.0), KCMBT (version 1.0), MSPKmerCounter (version 0.1), GenomeTester4 (version 4.0), aTurtle (version 0.3), KAnalyze (version 2.0.0), DSK (version 2.2.0), Jellyfish (version 2.2.6), and BFCounter (version 1.0). Tools from 2010 or earlier were excluded. All tools used are freely available to download (refer to Table S2 of the Supplementary Material).

To make a reasonable assessment of these tools, we applied them to seven datasets of varying sizes, mostly those used by Kokot et al. [37]. Table 2 summarizes details of the datasets used. FV and DM are small datasets; HS2 is the largest. NC and AT (the same used by Gerbil [31]) were

chosen because their longer read lengths would allow performance with larger values of *k* to be tested. All seven datasets are available to freely download (see Supplementary Table S2).

Table 2 Datasets used in our study

Sr. no.	Dataset ID	Organism	Genome size (Mbases)	Input FASTQ/FASTA file size (Gbytes) (1 Gbyte = 10^9 bytes)	Average read length (bases)	Total no. of bases (Gbases)	Total no. of reads
1	FV	*F. vesca*	214	10.9	353	4.5	12803137
2	DM	*D. melanogaster*	122	10.5	76	3.7	48432878
3	MB	*M. balbisiana*	472	197.1	100	56.3	562968372
4	HS1	*H. sapiens 1*	2991	292.1	151	123.7	819148264
5	HS2	*H. sapiens 2*	2991	339.5	100	135.3	1339740542
6	NC	*N. crassa*	41	23.3	7778.3	22.9	2942564
7	AT	*A. thaliana*	120	72.7	4804.6	36.1	7515360

Sequencing reads in each file (for each dataset) were first decompressed, and then concatenated into a single FASTA/FASTQ file to facilitate the smooth running of each tool. However, not all tools support direct decompression. All datasets used in this study had multiple compressed files. Some *k*-mer counting tools directly support compressed (raw) input, and can thus effectively perform parallelization in their first phase by reading from individual input files using separate threads. This means that restricting the input to a single file effectively limits them

to one or two threads (e.g., one to parse, and one to bin/partition). Most tools would likely perform even better on multi-file inputs without being concatenated (normalized) into a single file. We tested the effect of compressed input (gzip and bzip2) on the performance of various programs by running them directly on compressed input files.

Tables 4–8 present comparisons of the different tools, which were tested on the FV, DM, MB, HS1 and HS2 datasets for two values of *k* (28 and 55). Tests lasting >15 hours were interrupted.

Tools that approximate the frequency histogram of *k*-mer occurrences (and/or estimate the number of unique *k*-mers and singleton *k*-mers) by streaming data analysis are not considered in this paper. These include KmerStreame [58], ntCard [59], KmerGenie [30] and Khmer [60]. To make a fair comparison, we have only tested tools that generate exact *k*-mer counts.

The wall clock time was measured using the C++ function, 'gettimeofday()' averaged over three runs. A shell script by Shin [61] was used to measure the maximum memory usage. This script uses the Linux *ps* utility to determine the peak memory use of a process and its threads by monitoring resident set size (*RSS*) values, where *RSS* reports the amount of memory actually allocated to a process and is in memory (RAM). We reported the maximum disk usage by the program using our own shell script, which logs the disk usage at regular time intervals between consecutive checks using the Linux command *du*. This script also captures average CPU utilization as a percentage, with the help of the Linux command *top*. These scripts were executed with a sampling rate of 3 for HS1 and HS2 datasets, and a sampling rate of 1 for FV, DM, MB, NC, and AT datasets. Invocations of all executables were monitored by these two scripts. Run time, memory usage, disk usage, and CPU utilization were measured simultaneously.

All experiments were performed on a test machine configured as shown in Table 3. Commands used to run all programs were adapted from their documentation and/or publications (see

Supplementary Material). Commands used to list *k*-mers and their counts in human-readable format and *k*-mer coverage distribution (histogram for *k*-mer abundance) are also given in the Supplementary Material.

Table 3 Test machine configuration

**Processor**	Intel(R) Xeon(R) CPU E5-2698 v3 @ 2.30GHz
**Main memory**	64 GB
**Hard disk drive**	1 TB
**CPU(s)**	16
**Online CPU(s) list**	0–15
**Thread(s) per core**	2
**Core(s) per socket**	16
**No. of sockets**	1

We evaluated the accuracy of each counting program by comparing their *k*-mer frequency histograms on two small datasets with two values of *k*. This histogram is a table of f_i values, where f_i denotes the number of distinct singleton *k*-mers that appear *i* times in the set of reads [58]. Some tools, such as Jellyfish, DSK, Gerbil and MSPKmerCounter can directly create histograms. For the other tools, *k*-mer frequency histograms were obtained as follows. First, the dump subroutine of the tool was run, to write *k*-mer occurrences into a tab-separated text file. Second, we used our program, written in C++ using OpenMP for multithreaded computing and Linux commands (*grep* and *wc*), to generate the *k*-mer frequency histogram from this text file.

For each tool, exact numbers of f_1–f_{10} are given in the appendix. Space limitation means that results are only reported up to f_{10}, but all frequency counts were considered and compared. Results for Jellyfish 2.2.6, DSK 2.2.0, KAnalyze 2.0.0, KMC3, Gerbil 1.0, KCMBT 1.0, GenomeTester4, and BFCounter 1.0 were the same for both datasets and values of k. In contrast, the results for MSPKmerCounter 0.1, aTurtle 0.3, and Gerbil 1.0 (only for k = 55) were different. Error rates of these three tools are provided in the Supplementary Material (Tables S3–S6). MSPKmerCounter had the highest error rates, as depicted in Table S3–S6.

Lists of k-mers and their counts generated by Turtle, MSPKmerCounter, and Gerbil (only for k = 55) did not always match with outputs from other tools, for the same datasets. For more rigorous analysis, we used our shell script, written using a set of Linux utilities, i.e., *sort* (to sort in lexicographical order) and *diff* (which analyses two files and prints the lines that are different) to validate all lexicographically sorted k-mers and their counts.

DSK output is used as a reference to validate the output of aTurtle because these two tools use the same alphabetical order ($A < C < T < G$), while obtaining canonical k-mers. Variations were found in the lexicographically sorted k-mers in the outputs of aTurtle and DSK (although the frequency counts of aTurtle matched with those of DSK for the DM dataset for k = 55). The aTurtle output included unmatched frequency k-mers, and some additional k-mers that were not present in the DSK output, and some k-mers present in the DSK output were missing from aTurtle. Similar variations were observed between MSPKmerCounter and KMC3, and between Gerbil and KMC3 (but only for k = 55).

We thus infer that the recent versions of MSPKmerCounter, aTurtle and Gerbil may contain bugs in their implementations.

Result and discussion

Table 4 Experimental results for the FV dataset

SN	Tools (version; compression type)	*k* = 28				*k* = 55			
		Time (s)	RAM (GB)	Disk (GB)	CPU utilization (%) (comment)	Time (s)	RAM (GB)	Disk (GB)	CPU Utilization (%) (comment)
1	Jellyfish (2.2.6)	138.33	7.9	0	1093.55 (consistent)	226	***36.19***	0	**1050.93*** (consistent)
2	DSK (2.2.0)	56.33	6.35	6	866.50 (consistent)	78.33	7.04	5	633.49 (declined from ~1174 to ~129.7)
3	DSK (2.2.0; gzip)	197	4	6	402.71 (first 80% of time consistent with ~300; last 20% inconsistent to ~1200 with sudden increase)	222	6	5	441.21 (first 75% of time consistent with ~390; last 25% inconsistent to ~1200 with sudden increase)
4	KAnalyze (2.0.0)	***2042***	10	***22.2***	509.20	***4095***	11	***42***	337.46

					(initially in the range 1000–2000 then declined to ~200)				(initially in the range 1000–2000 then declined to ~150)
5	KAnalyze (2.0.0; gzip)	1999	9	22.9	507.84 (first 30% of time inconsistent in the range 2250–750; last 70% consistent with sudden drop to ~200)	3395	11	12.8	360.456 (first 25% of time inconsistent to ~900; last 75% consistent to ~200 with a sudden drop)
6	KMC3	38.66	7.66	**4***	998.10 (consistent)	**35***	11.2	4	987.891 (consistent)
7	KMC3 (gzip)	35	7	2.2	1004.61 (consistent)	37	11	0	1056.25 (consistent)
8	Gerbil (1.0)	**33.66 ***	**0.83***	**4***	**1110.38*** (consistent)	60.33	**1.29***	**3***	1030.50 (consistent)
9	Gerbil (1.0; gzip)	49	0.82	1.5	858.46 (first 50% of time consistent	55	1	1	880.77 (first 50% of time consistent

					with ~600; last 50% suddenly increased to ~1200)				with ~600; last 50% suddenly increased to ~1300)
10	KCMBT (1.0)	137.5	***30.98***	0	628.87 (inconsistent)	Not supported			
11	MSPKmerCounter (0.1)	59.33	4.45	1	811.70 (phase 1: consistent (~200); phase 2: consistent (~1500))	67.33	4.61	1	770.87 (Phase 1: consistent (~200); phase 2: consistent (~1500))
12	aTurtle (0.3)	671	14	0	***99.14*** (consistent)	1185	26	0	***94.12*** (consistent)
13	GenomeTester4	214	26	0	***202.33*** (consistent)	Not supported			
14	BFCounter (1.0)	1731	3	0	274.10 (first 80% of time almost 100, then gradual increase to ~1000)	1790	9	0	***271.80*** (consistent to ~100, but bars hiking to ~1000 in the middle and end)
15	BFCounter (1.0;	1847	3	0	259.70	1889	9	0	251.49

	gzip)				(inconsistent)				(inconsistent)

Entries in bold with * indicate best results; entries in bold italic (including second lowest for CPU utilization) show average results. Since MSPKmerCounter has the highest error rates, these results, and those for compressed input, were not considered in the best and average results. In the 'Disk' column, only disk-based tools were considered in the best and average results. Abbreviations: s = seconds, GB = gigabytes.

Table 5 Experimental results for the DM dataset

**SN**	**Tools (version; compression type)**	***k* = 28**				***k* = 55**			
		**Time (s)**	**RAM (GB)**	**Disk (GB)**	**CPU utilization (%) (comment)**	**Time (s)**	**RAM (GB)**	**Disk (GB)**	**CPU utilization (%) (comment)**
1	Jellyfish (2.2.6)	77	4	0	1055.25 (consistent)	71	9	0	917.79 (consistent)
2	DSK (2.2.0)	52	2	4.2	736.09 (initially ~600, increasing to ~1173)	49	2	2.7	622.36 (initially ~500, increasing to ~1150)
3	DSK (2.2.0; gzip)	183	4.68	3.6	331.88 (first 90% of time	173	4.45	2.4	300.70 (first 90% of time

					consistent with ~270, last 10% suddenly increasing to ~1200)				consistent with ~250 then suddenly increasing to ~1200)
4	KAnalyze (2.0.0)	794	10	***14.3***	695.64 (gradually declined)	393	11	***12.8***	829.45 (gradually declined from ~2000 to ~100)
5	KAnalyze (2.0.0; gzip)	822	9	14.4	691.15 (first 40% of time consistent with ~1250; last 60% inconsistent with sudden drop to ~200)	411	11	12.9	843.79 (first 60% of time inconsistent in the range 2250–900, rest of time inconsistent with sudden drop to ~200)
6	KMC3	**18***	5	2.23	942.26 (consistent)	**13***	8	**0.6***	**1023.54*** (consistent)

7	KMC3 (gzip)	35	5	1.64	739.01 (last 20% of time consistent with ~1250, rest consistent with ~700)	31	8	0	637.29 (last 20% of time consistent with ~1250, rest consistent with ~600)
8	Gerbil (1.0)	20	**0.81***	**2.11***	**1184.23** (consistent)	16.5	**0.81***	4	1010.89 (consistent)
9	Gerbil (1.0; gzip)	33	0.82	1.31	821.69 (first 55% of time consistent with ~700, last 45% suddenly increased to ~1200)	29	0.81	0	685.74 (first 55% of time consistent with ~600, last 45% inconsistent with sudden increase to ~1200)
10	KCMBT (1.0)	61	2	0	595.37 (initially ~300 then increased towards end	Not supported			

					to ~900)				
11	MSPKmerCounter (0.1)	234	5	14.2	912.92 (both phases: consistent)	219	5	11.2	914.62 (phase 1: initially ~1000 then declined to ~300; phase 2: consistent)
12	aTurtle (0.3)	423	7	0	***97.20*** (consistent)	330	12	0	***95.39*** (consistent)
13	GenomeTester4	144	***23***	0	***183.92*** (consistent)	Not supported			
14	BFCounter (1.0)	***914***	1	0	307.53 (consistent)	***477***	2	0	***331.48*** (first 95% of time in the range 250–400, then increasing to ~800)
15	BFCounter (1.0; gzip)	1002	2	0	321.78 (last 20% of time ~500, rest is	559	2	0	306.30 (last 20% of time ~500, rest

					~300)				~300)

Entries in bold with * indicate best results; entries in bold italic (including second lowest for CPU utilization) show average results. Since MSPKmerCounter had the highest error rates, these results, and those for compressed input, were not considered in the best and average results. In the ‘Disk’ column, only disk-based tools were considered for the best and average results. Abbreviations: s = seconds, GB = gigabytes.

Table 6 Experimental results for the MB dataset

**SN**	**Tools (version; compression type)**	***k* = 28**				***k* = 55**			
		**Time (s)**	**RAM (GB)**	**Disk (GB)**	**CPU utilization (%) (comment)**	**Time (s)**	**RAM (GB)**	**Disk (GB)**	**CPU utilization (%) (comment)**
1	Jellyfish (2.2.6)	**1467***	15	0	**800.13*** (consistent)	**1440***	***24***	0	**691.65*** (consistent)
3	DSK (2.2.0)	3358	12	59	185.09 (consistent)	3039	11	45	***208.54*** (consistent)
4	KAnalyze (2.0.0)	***51422***	10	***189***	279.40 (initially ~2000, then declining to ~150)	***45367***	11	***245***	248.04 (declined from ~2000 to ~100)

5	KMC3	2019	9	36	216.93 (initially in the range 12–400; increasing towards end to ~600)	1804	10	14	211.12 (initially in the range 12–400, increasing towards end to ~600)
6	KMC3 (bz2)	3341	11	36.3	289.46 (first 90% of time consistent in the range 200–400; last 10% up to ~1300)	3250	11	13	282.77 (first 90% consistent in range 200–400; last 10% up to ~1300)
7	Gerbil (1.0)	2238	**2***	**32***	269.52 (initially within 150, increasing towards end to ~800)	1941	**3***	**11***	250.32 (initially within 150, increasing towards end to ~800)
8	Gerbil (1.0; bz2)	3487	2	30.7	306.37 (first 90% of time consistent	3137	3	11	304.02 (first 90% of time consistent

					with ~270; last 10% suddenly increasing to ~1300)				with ~270; last 10% suddenly increasing to ~1300)
9	KCMBT (1.0)	1644	34	0	***135.87*** (consistent)	Not supported			
10	MSPKmerCounter (0.1)	11094	8	173	316.90 (consistent)	8759	9	118	1284.05 (consistent)
11	aTurtle 0.3	8764	***61***	0	***75.07*** (consistent)	> 15 Hours			
12	GenomeTester4	3520	60	0	153.67 (consistent)	Not supported			
13	BFCounter (1.0)	18950	10	0	300.37 (consistent)	15264	19	0	295.40 (first 50% up to ~254 then increasing to ~434)

Failure messages are indicated above if a program failed to complete the computation because of insufficient memory/disk space, or within a stipulated time (15 hours). Entries in bold with * indicate best results; entries in bold italic (including second lowest for CPU utilization) show average results. Since MSPKmerCounter had the highest error rates, these results, and those for

compressed input, were not considered in the best and average results. In the ‘Disk’ column, only disk-based tools were considered for the best and average results. Abbreviations: s = seconds, GB = gigabytes.

For the FV and DM datasets, all programs completed the *k*-mer count within 15 hours. However, for the HS1 and HS2 datasets, KCMBT and GenomeTester4 did not complete within 15 hours, and neither did Jellyfish for the HS1 dataset, even in Bloom-filter-based mode. These jobs also had to be killed because high memory usage froze the system. For the HS1 and HS2 datasets, aTurtle failed, returning the ‘std::bad_alloc Aborted (core dumped)’ error message because of high memory usage, and KAnalyze with the ‘java.io.IOException: No space left on device’ error.

Table 7 Experimental results for the HS1 dataset

SN	Tools (version; compression type)	*k* = 28				*k* = 55			
		Time (s)	RAM (GB)	Disk (GB)	CPU utilization (%) (comment)	Time (s)	RAM (GB)	Disk (GB)	CPU utilization (%) (comment)
1	Jellyfish (2.2.6)	> 15 hours (system hang)				> 15 hours (system hang)			
2	DSK (2.2.0)	***7722***	***12***	***133***	***210.2*** (inconsistent)	***9389***	***14***	***48***	***255.862*** (inconsistent)
3	DSK (2.2.0; gzip)	9240	11	134	218.77 (inconsistent)	8480	12	104	284.68 (inconsistent)

4	KAnalyze (2.0.0)	Failed: ‘IO error writing segment file: no space left on device’				Failed: ‘IO error writing segment file: No space left on device’			
5	KAnalyze (2.0.0; gzip)	Failed: ‘IO error writing segment file: no space left on device’				Failed: ‘IO error writing segment file: No space left on device’			
6	KMC3	**3725***	10	78	276.64 (gradually declined)	**3466***	**11***	28	270.55 (inconsistent)
7	KMC3 (gzip)	1964	11	79	620.84 (inconsistent)	1626	11	29	663.31 (inconsistent)
8	Gerbil (1.0)	4078	**6***	**66***	**370.77*** (initially within ~200, increasing towards end to ~1200)	3818	**11***	**21***	**320.21*** (inconsistent)
9	Gerbil (1.0; gzip)	2849	6	66	569.83 (first 70% of time consistent to ~420, then increasing to ~1000 for last 30%)	2614	11	22	541.63 (first 70% of time consistent to ~400, then increasing to ~1000 for last 30%)
10	KCMBT (1.0)	> 23 hours				Not supported			

11	MSPKmerCounter (0.1)	> 15 hours (Phase 2 failed: 'OutOfMemoryError')	> 15 hours (Phase 2 failed: 'OutOfMemoryError')
12	aTurtle (0.3)	Aborted (core dumped)	Aborted (core dumped)
13	GenomeTester4	> 15 hours	Not supported
14	BFCounter 1.0	> 15 hours	> 15 hours
15	BFCounter (1.0; gzip)	> 15 hours	> 15 hours

Failure messages are indicated above if a program failed to complete the computation because of insufficient memory/disk space, or within a stipulated time (15 hours). Entries in bold with * indicate best results; entries in bold italic (including second lowest for CPU utilization) show average results. Since MSPKmerCounter had the highest error rates, these results, and those for compressed input, were not considered in the best and average results. In the 'Disk' column, only disk-based tools were considered for the best and average results. Abbreviations: s = seconds, GB = gigabytes.

Table 8 Experimental results for the HS2 dataset

**SN**	**Tools (version; compression type)**	***k* = 28**				***k* = 55**			
		**Time (s)**	**RAM (GB)**	**Disk (GB)**	**CPU utilization (%) (comment)**	**Time (s)**	**RAM (GB)**	**Disk (GB)**	**CPU utilization (%) (comment)**
1	Jellyfish (2.2.6)	**3310***	***58***	0	**1000.29***	***11126***	***48***	0	**376.578***

					(consistent)				(declined from ~1000 to ~100)
2	DSK (2.2.0)	***8879***	13	***145***	***186.66*** (consistent)	7982	13	***109***	***211.54*** (consistent)
3	DSK (2.2.0; gzip)	10360	10	146	242.01 (inconsistent)	10199	12	109	240.21 (first 60% of time consistent with ~300, last 40% suddenly increasing to ~650)
4	KAnalyze (2.0.0)	Failed: ‘IO error writing segment file: no space left on device’				Failed: ‘IO error writing segment file: no space left on device’			
5	KAnalyze (2.0.0; gzip)	Failed: ‘IO error writing segment file: no space left on device’				Failed: ‘IO error writing segment file: no space left on device’			
6	KMC3	4252	10	85	218.02 (increased towards end to ~600, otherwise up to ~12)	**3846***	11	29	214.99 (increased towards end to ~600, otherwise up to ~12)

7	KMC3 (gzip)	2362	10	86	580.72 (inconsistent)	1995	11	29	556.31 (inconsistent)
8	Gerbil (1.0)	4553	**5***	**74***	371.26 (increased towards end to ~1000, otherwise up to ~250)	4260	**9***	**23***	317.65 (initially ~250, increasing towards end to ~1000)
9	Gerbil (1.0; gzip)	3358	5	74	553.59 (first 70% of time consistent to ~400, then increasing to ~1000 for last 30%)	3121	9	23	507.19 (first 70% of time consistent to ~450, then increasing to ~1000 for last 30%)
10	KCMBT (1.0)	> 15 hours				Not supported			
11	MSPKmerCounter (0.1)	3128	6	22.2	120.17 (consistent)	3124	9	5.7	340.49 (consistent)
12	aTurtle (0.3)	Aborted (core dumped)				Aborted (core dumped)			
13	GenomeTester4	> 15 hours				Not supported			
14	BFCounter (1.0)	> 15 hours				> 15 hours			
15	BFCounter (1.0; gzip)	> 15 hours				> 15 hours			

Failure messages are indicated above if a program failed to complete the computation because of insufficient memory/disk space, or within a stipulated time (15 hours). Entries in bold with * indicate best results; entries in bold italic (including second lowest for CPU utilization) show average results. Since MSPKmerCounter had the highest error rates, these results, and those for compressed input, were not considered in the best and average results. In the 'Disk' column, only disk-based tools were considered for the best and average results. Abbreviations: s = seconds, GB = gigabytes.

For the HS1 and HS2 datasets, BFCounter was unable to complete within 15 hours and the system froze, therefore the job was killed. For the HS1 dataset, MSPKmerCounter failed during phase 2, returning the 'OutOfMemoryError' error.

Runtime, memory and disk usage

Table 9 provides an easily readable comparison of all ten tools (excluding compressed input results), including the best and average programs in terms of time, memory, disk, and CPU utilization.

Of all the tested programs, only DSK and KMC3 generated accurate results for both *k* values, within the stipulated time, and without system freeze issues for all seven datasets (see Tables A1 and A2 in the Appendix; accuracy checked against FV and DM datasets only),

Table 9 Summary of Tables 4–8

**Dataset**	***k*-length**	**Time**	**RAM**	**Disk**	**CPU Utilization (%)**

ID		Highest	Lowest	Highest	Lowest	Highest	Lowest	Highest	Lowest
FV	28	KAnalyze	Gerbil	KCMBT	Gerbil	KAnalyze	Gerbil, KMC3	Gerbil	GenomeTester4, aTurtle
	55	KAnalyze	KMC3	Jellyfish	Gerbil	KAnalyze	Gerbil	Jellyfish	BFCounter, aTurtle
DM	28	BFCounter	KMC3	GenomeTester4	Gerbil	KAnalyze	Gerbil	Gerbil	GenomeTester4, aTurtle
	55	BFCounter	KMC3	aTurtle	Gerbil	KAnalyze	KMC3	KMC3	BFCounter, aTurtle
MB	28	KAnalyze	Jellyfish	aTurtle	Gerbil	KAnalyze	Gerbil	Jellyfish	KCMBT, aTurtle
	55	KAnalyze	Jellyfish	Jellyfish	Gerbil	KAnalyze	Gerbil	Jellyfish	DSK
HS1	28	DSK	KMC3	DSK	Gerbil	DSK	Gerbil	Gerbil	DSK
	55	DSK	KMC3	DSK	Gerbil, KMC3	DSK	Gerbil	Gerbil	DSK
HS2	28	DSK	Jellyfish	Jellyfish	Gerbil	DSK	Gerbil	Jellyfish	DSK
	55	Jellyfish	KMC3	Jellyfish	Gerbil	DSK	Gerbil	Jellyfish	DSK

Because it is single-threaded, aTurtle will always be lowest in terms of CPU utilization. Therefore, the second lowest entries are also mentioned in the ‘CPU Utilization (%)’ column.

DSK consistently used a moderate amount of memory, had reasonable speeds, and in passing all the tests, demonstrated robustness. KMC3 was often superior in terms of running time, but

used more memory than its top competitor, Gerbil. However, it was often close to being the best in terms of disk utilization.

Interestingly, Gerbil was consistently the most memory and disk-efficient approach. For most of the datasets, Gerbil had the lowest disk utilization, but was slower than KMC3 (the GPU implementation of Gerbil was not considered). Gerbil attempts to reduce disk and memory utilization, making it economical in terms of both of these parameters. As seen in Table 9, hash table-based counting approaches seem to be more efficient than sorting-based approaches in terms of hardware use. Newer tools, specifically KMC3 and Gerbil, which use MSP and bin size (signature) balancing, performed best in terms of memory requirements.

For most of the datasets, and for both *k* values, KAnalyze had much higher runtime and disk usage compared to the other disk-based approaches. KAnalyze needs more time for the merging step because its partitioning step is relatively straightforward.

In-memory approaches need no extra disk space because these are completely memory-based. Among the in-memory algorithms, BFCounter utilized the lowest amount of memory because of the underlying memory-efficient Bloom filter. For the MB dataset, Jellyfish was the fastest in-memory algorithm and had the highest CPU utilization, but for the HS1 dataset, it was not able to complete within 15 hours. For the datasets that Jellyfish was able to complete within the stipulated time, both time and memory requirements were comparable. Gerbil and Jellyfish often had the highest CPU utilization. Perez et al. [25] reported similar behaviors of various *k*-mer counting tools in terms of runtime and memory usage.

Performance for larger values of k

GenomeTester4, KCMBT and aTurtle do not support large values of *k* (see Table S1 of the Supplementary Material). For the NC and AT datasets, BFCounter failed, returning a 'segmentation fault (core dumped)' error, whereas Jellyfish and KAnalyze did not complete within 15 hours. Only KMC3, DSK, and Gerbil succeeded in generating results for the different *k* values (28, 40, 55, 65, 100, 125, 150, 175 and 200) for these datasets within the stipulated time (see Figure 1). MSPKmerCounter was unable to generate output for the NC dataset, but for the AT dataset, succeeded for all values of *k* (28, 40, 55, 65, 100, 125, 150, 175 and 200). However, because of its high error rate, MSPKmerCounter is not included in our comparisons.

Gerbil was consistently the most memory-efficient approach, but when the value of *k* increased, it utilized almost the same amount of memory as DSK and KMC3. For the NC dataset, KMC3 was faster than Gerbil, but at higher *k* values (150, 175 and 200), they had similar runtime. For the AT dataset, KMC3 was faster than DSK and Gerbil. DSK used almost the same amount of memory as KMC3, but was slower than KMC3 and Gerbil. In taking minimal time and using moderate amounts of memory, KMC3 and Gerbil efficiently support both small and large values of *k*.

Figure 1. Analysis of time (minutes) and memory (GB) utilization of *k*-mer counting algorithms for the NC and AT datasets for different values of *k* (28, 40, 55, 65, 100, 125, 150, 175 and 200)

Scalability to varying sizes of datasets

For datasets with shorter reads, such as DM, the runtime of each tool decreases with increasing values of *k* (Table 5). Only three programs, Gerbil, KMC3 and DSK, succeeded in generating results for large datasets (HS1 and HS2) within a reasonable timeframe and without freezing

(Tables 7 and 8). These tools use a disk-based approach, are more efficient in terms of time and memory utilization, and are scalable for large datasets (size > 200 GB) compared to tools based on an in-memory approach. Jellyfish is memory-efficient, and for the HS2 dataset, was the only in-memory algorithm that completed a *k*-mer count within a reasonable time, consuming 58 GB and 48 GB memory for $k = 28$ and 55, respectively (Table 8).

The impact of compressed input

Because of their large size, sequencing data are generally stored in a compressed format, usually gzip. One advantage of programs that support compressed input is that the I/O throughput improves with the use hard drives because the algorithm consumes the data faster. Data throughput is increased because compressed data are read directly from the disk, thereby overcoming the cost of file decompression in memory.

Currently, only five tools support compressed input: KMC3, Gerbil, DSK, KAnalyze, and BFCounter. As shown in Tables 4–8, reading the input in a compressed form reduces the running time, having a noticeable, positive impact on counting *k*-mers in large datasets, i.e., HS1 and HS2. KMC3 was the fastest of the tested programs that support compressed input. For the HS1 and HS2 datasets, KMC3 and Gerbil's gzipped input time was much less than the normalized input time, whereas the opposite trend was seen with DSK. As shown in Tables 7 and 8, using gzipped input compared to normalized input, KMC3 was approximately 47% and 53% faster ($k = 28$ and $k = 55$, respectively) for the HS1 dataset, and 44% and 48% faster ($k = 28$ and $k = 55$, respectively) for the HS2 dataset; Gerbil was approximately 30% and 32% faster ($k = 28$ and $k = 55$, respectively) for the HS1 dataset, and approximately 26% and 27% faster ($k = 28$ and $k = 55$,

respectively) for the HS2 dataset; and DSK was approximately 19% slower (k = 28(for HS1, and 17% and 28% slower (k = 28 and k = 55, respectively) for HS2.

Regardless of input format (compressed or normalized), KMC3 was the fastest, whereas Gerbil was the most consistent in terms of using the lowest memory and disk space. bzip2 has a high compression ratio, but very slow decompression. Thus, processing a bzip2 file is costlier than gzipped input (Tables 4–8). Only KMC3 and Gerbil support input data that are compressed with the bzip2 data compressor; these took long to process bzip2 files than normalized input for the MB dataset (Table 6).

Scalability by the number of threads

All programs (except for aTurtle, as it is single-threaded) run with different numbers of threads (1, 2, 4, 6, 8 and 12) to assess CPU-related performance. FV and MB datasets were chosen with a k value of 28 so that all tools could complete their execution within the stipulated time. Figure 2 shows the scalability of these tools according to the number of threads. No tool was able to achieve linear speedup.

Figure 2. Scalability comparison of different k-mer counting tools based on the number of threads

Jellyfish, an in-memory approach using a multithreaded lock-free hash table, has the highest speedup, i.e., 8.3 and 6.7, for FV and MB datasets, respectively, for 12 threads. DSK and KMC3 have good speedup for the FV dataset, i.e., 7.2 and 7.1, respectively, for 12 threads. For the MB dataset, each program achieves only low speedup in the range of 1–2 (except for Jellyfish). This

is caused by increased threading overhead; resource demand is increased by each thread because of large input size but limited underlying resources. KCMBT is the fastest for a single thread on the MB dataset (Table S8 Supplementary Material). However, for the four threads, KCMBT was not able to complete a *k*-mer count within 15 hours for the same dataset owing to threading overheads, while memory requirements are almost constant for increasing numbers of threads with the other tools. The overall speedup achieved by each program is not very high because *k*-mer counting is fundamentally an I/O-intensive task.

Conclusions and future directions

k-mer counting is used to solve many problems in bioinformatics. While high-throughput sequencing technologies can generate billions of reads per instrument run, there is a need to continue developing a memory and time-efficient *k*-mer counting system for large reads.

Many disk-based and in-memory *k*-mer counting approaches are available, which aim to generate results from large genomic datasets in a minimum amount of time, using personal computers with limited resources (memory, disk, etc.).

Of all the tools considered herein, KMC3, DSK and Gerbil are the most flexible and efficient, as they have higher speeds, minimum memory requirements and better scalability to larger datasets. They also have automatic parameter selection, are more robust, support larger values of *k* (large *k* being an important use case for longer reads), and compressed input. Reading the input in a compressed form improves the overall processing time. These tools are optimized to gain significant speedup by parallelizing the available cores in the machine.

As sequencing technologies evolve, research endeavors must continue to improve to develop better *k*-mer counting systems for increasingly large sequencing datasets.

Abbreviations

CPU: Central Processing Unit; GB: Gigabytes; GPU: Graphics Processing Unit; I/O: input/output; RAM: Random Access Memory; NGS: Next-generation Sequencing; SAC: Sort-and-compact; CQF: Counting Quotient Filter; MSP: Minimum Substring Partitioning; SRA: Sequence Read Archive; FV: F.vesca; DM: D. melanogaster; MB: M. balbisiana; HS1: H. sapiens 1; HS2: H. sapiens 2; NC: N. crassa; AT: A. thalia

References

1. Reuter JA, Spacek DV, Snyder MP. High-Throughput Sequencing Technologies. Mol Cell 2015;**58**(4):586–97.
2. Molnar M, Ilie L. Correcting Illumina data. Brief Bioinform 2015;**16**(4):588–99.
3. Miller JR, Delcher AL, Koren S, et al. Aggressive assembly of pyrosequencing reads with mates. Bioinformatics 2008;**24**(24):2818–24.
4. Myers EW, Sutton GG, Delcher AL et al. A whole-genome assembly of Drosophila. Science 2000;**287**(5461):2196–204.
5. Jaffe DB, Butler J, Gnerre S, et al. Whole-Genome Sequence Assembly for Mammalian Genomes : Arachne 2. Genome Res 2003;**13**(1):91–6.
6. Miller JR, Koren S, Sutton G. Assembly algorithm for next-generation sequencing data. Genomics 2010;**95**(6):315–27.
7. Pevzner PA, Tang H, Waterman MS. An Eulerian path approach to DNA fragment assembly. Proc Natl Acad Sci U S A 2001;**98**(17):9748–53.
8. Zerbino DR, Birney E. Velvet: Algorithms for de novo short read assembly using de Bruijn graphs. Genome Res 2008;**18**(5):821–9.

9. Simpson JT, Wong K, Jackman SD, et al. ABySS: A parallel assembler for short read sequence data. Genome Res 2009;**19**(6):1117–23.

10. Kelley DR, Schatz MC, Salzberg SL. Quake: quality-aware detection and correction of sequencing errors. Genome Biol 2010;**11**(11):R116.

11. Shi H, Schmidt B, Liu W, et al. A parallel algorithm for error correction in high-throughput short-read data on CUDA-enabled graphics hardware. J Comput Biol 2010;**17**(4):603–15.

12. Liu Y, Schroder J, Schmidt B. Musket: A multistage k-mer spectrum-based error corrector for Illumina sequence data. Bioinformatics 2013;**29**(3):308–15.

13. Medvedev P, Scott E, Kakaradov B, et al. Error correction of high-throughput sequencing datasets with non-uniform coverage. Bioinformatics 2011;**27**(13):i137–41.

14. Salmela L, Schröder J. Correcting errors in short reads by multiple alignments. Bioinformatics 2011;**27**(11):1455–61.

15. Edgar RC. MUSCLE: Multiple sequence alignment with high accuracy and high throughput. Nucleic Acids Res 2004;**32**(5):1792–7.

16. Liu B, Shi Y, Yuan J, et al. Estimation of genomic characteristics by analyzing k-mer frequency in de novo genome projects. arXiv preprint arXiv:1308.2012 2013. http://arxiv.org/abs/1308.2012.

17. Price AL, Jones NC, Pevzner PA. De novo identification of repeat families in large genomes. Bioinformatics 2005;**21**(suppl_1):i351–8.

18. Li R, Ye J, Li S, et al. ReAS: Recovery of ancestral sequences for transposable elements from the unassembled reads of a whole genome shotgun. PLoS Comput Biol 2005;**1**(4):e43.

19. Campagna D, Romualdi C, Vitulo N, et al. RAP: A new computer program for de novo identification of repeated sequences in whole genomes. Bioinformatics 2005;**21**(5):582–8.

20. Lefebvre A, Lecroq T, Dauchel H, et al. FORRepeats: Detects repeats on entire chromosomes and between genomes. Bioinformatics 2003;**19**(3):319–26.

21. Healy J, Thomas EE, Schwartz JT, et al. Annotating large genomes with exact word matches. Genome Res 2003;**13**(10):2306–15.

22. Kurtz S, Narechania A, Stein JC, et al. A new method to compute K-mer frequencies and its application to annotate large repetitive plant genomes. BMC Genomics 2008;**9**(1):517.

23. Sindi SS, Hunt BR, Yorke JA. Duplication count distributions in DNA sequences. Phys Rev E 2008;**78**(6):061912.

24. Pajuste FD, Kaplinski L, Mols M, Puurand T, Lepamets M, Remm M. FastGT: an alignment-free method for calling common SNVs directly from raw sequencing reads. Sci Rep 2017;**7**(1):2537.

25. Pérez N, Gutierrez M, Vera N. Computational performance assessment of k-mer counting algorithms. J Comput Biol 2016;**23**(4):248–55.

26. Roberts RJ, Carneiro MO, Schatz MC. The advantages of SMRT sequencing. Genome Biol 2013; **14**(6):6–9.

27. Laehnemann D, Borkhardt A, McHardy AC. Denoising DNA deep sequencing data-high-throughput sequencing errors and their correction. Brief Bioinform 2016;**17**(1):154–79.

28. Sameith K, Roscito JG, Hiller M. Iterative error correction of long sequencing reads maximizes accuracy and improves contig assembly. Brief Bioinform 2017;**18**(1):1–8.

29. Xavier BB, Sabirova J, Pieter M, et al. Employing whole genome mapping for optimal de novo assembly of bacterial genomes. BMC Res Notes 2014;**7**(1):484.

30. Chikhi R, Medvedev P. Informed and automated k-mer size selection for genome assembly. Bioinformatics 2014;**30**(1):31–7.

31. Erbert M, Rechner S, Muller-Hannemann M. Gerbil: A fast and memory-efficient k-mer counter with GPU-support. Algorithms Mol Biol 2017;**12**(1):9.

32. Li Y, XifengYan. MSPKmerCounter: A Fast and Memory Efficient Approach for K-mer Counting. ArXiv e-prints 2015. http://arxiv.org/abs/1505.06550.

33. Rizk G, Lavenier D, Chikhi R. DSK: K-mer counting with very low memory usage. Bioinformatics 2013;**29**(5):652–3.

34. Pandey P, Bender MA, Johnson R, et al. Squeakr: An Exact and Approximate k-mer Counting System. Bioinformatics 2017;**34**(4):568–575.

35. Marçais G, Kingsford C. A fast, lock-free approach for efficient parallel counting of occurrences of k -mers. Bioinformatics 2011;**27**(6):764–70.

36. Melsted P, Pritchard JK. Efficient counting of k-mers in DNA sequences using a bloom filter. BMC Bioinformatics 2011;**12**(1):333.

37. Kokot M, Długosz M, Deorowicz S. KMC 3: counting and manipulating k-mer statistics. Bioinformatics 2017;**33**(17):2759–2761.

38. Kaplinski L, Lepamets M, Remm M. GenomeTester4: a toolkit for performing basic set operations - union, intersection and complement on k-mer lists. GigaScience 2015;**4**(1):58.

39. Deorowicz S, Kokot M, Grabowski S, et al. KMC 2: fast and resource-frugal k-mer counting. Bioinformatics 2015;**31**(10):1569–76.

40. Audano P, Vannberg F. KAnalyze: A fast versatile pipelined K-mer toolkit. Bioinformatics 2014;**30**(14):2070–2.

41. Deorowicz S, Debudaj-grabysz A, Grabowski S. Disk-based k-mer counting on a PC. BMC Bioinformatics 2013;**14**(1):160.

42. Roy RS, Bhattacharya D, Schliep A. Turtle : Identifying frequent k -mers with cache-efficient algorithms. Bioinformatics 2014;**14**(30):1950–7.

43. Mamun A, Pal S, Rajasekaran S. KCMBT : a k -mer Counter based on Multiple Burst Trees. Bioinformatics 2016;**32**(18):2783–90.

44. Salomon D. Data compression: the complete reference. Springer Science & Business Media; 2004.

45. Putze F, Sanders P, Singler J. Cache-, hash-, and space-efficient bloom filters. J Exp Algorithmics 2010;**14**:4:4.4–4:4.18.

46. Leiserson CE, Rivest RL, Stein C. Chapter 11: Hash Tables. Introd. to Algorithms. 2nd ed. Cambridge, Massachusetts London, England, McGraw-Hill; 2001. pp. 221–45. http://web.ist.utl.pt/fabio.ferreira/material/asa/clrs.pdf.

47. Purcell C, Harris T. Non-blocking hashtables with open addressing. Technical Report 639, University of Cambridge, Cambridge, UK; 2005.

48. Gao H, Groote JF, Hesselink WH. Almost wait-free resizable hashtables. IPDPS’04 IEEE Computer Society, Los Alamitos, CA, USA 2004;50a.

49. Shalev O, Shavit N. Split-ordered lists: Lock-free extensible hash tables. J ACM 2006;**53**(3):379–405.

50. Mapleson D, Accinelli GG, Kettleborough G, et al. KAT : A K-mer Analysis Toolkit to quality control NGS datasets and genome assemblies. Bioinformatics 2017;**33**(4):574–6.

51. Bloom BH. Space/Time Trade-offs in Hash Coding with Allowable Errors. COMMUN ACM 1970;**13**(7):422–6.

52. Randall D, Guerrieri A, Jin W. Bloom Filters and Hashing. CS 6550 Design and Analysis of Algorithms 2006.

53. Pandey P, Bender MA, Johnson R, et al. A General-Purpose Counting Filter. Proc 2017 ACM Int Conf Manag Data - SIGMOD'17 2017;775–87.

54. Abouelhoda MI, Kurtz S, Ohlebusch E. Replacing suffix trees with enhanced suffix arrays. J Discret Algorithms 2004;**2**(1):53–86.

55. Heinz S, Zobel J, Williams HE. Burst tries: a fast, efficient data structure for string keys. ACM T INFORM SYST 2002;**20**(2):192–223.

56. Li Y, Kamousi P, Han F, et al. Memory Efficient Minimum Substring Partitioning. In Proceedings of the VLDB Endowment 2013;**6**(3):169–80.

57. Kokot M, Deorowicz S, Debudaj-Grabysz A. Sorting Data on Ultra-Large Scale with RADULS New Incarnation of Radix Sort. arxiv.org/ 2016;1612.02557. http://arxiv.org/abs/1612.02557.

58. Melsted P, Halldórsson B V. KmerStream: Streaming algorithms for k-mer abundance estimation. Bioinformatics 2014;**30**(24):3541–7.

59. Mohamadi H, Khan H, Birol I. ntCard: A streaming algorithm for cardinality estimation in genomics data. Bioinformatics 2017;**33**(9):1324–30.

60. Junior LC, Brown CT. Efficient cardinality estimation for k-mers in large DNA sequencing data sets. bioRxiv 2016;1–5. https://doi.org/10.1101/056846.

61. https://gist.github.com/netj/526585/36515c55a3b25232bddfdd51e43cadc1a5f296de.

Figure 1

Click here to access/download;Figure;Figure 1.pdf

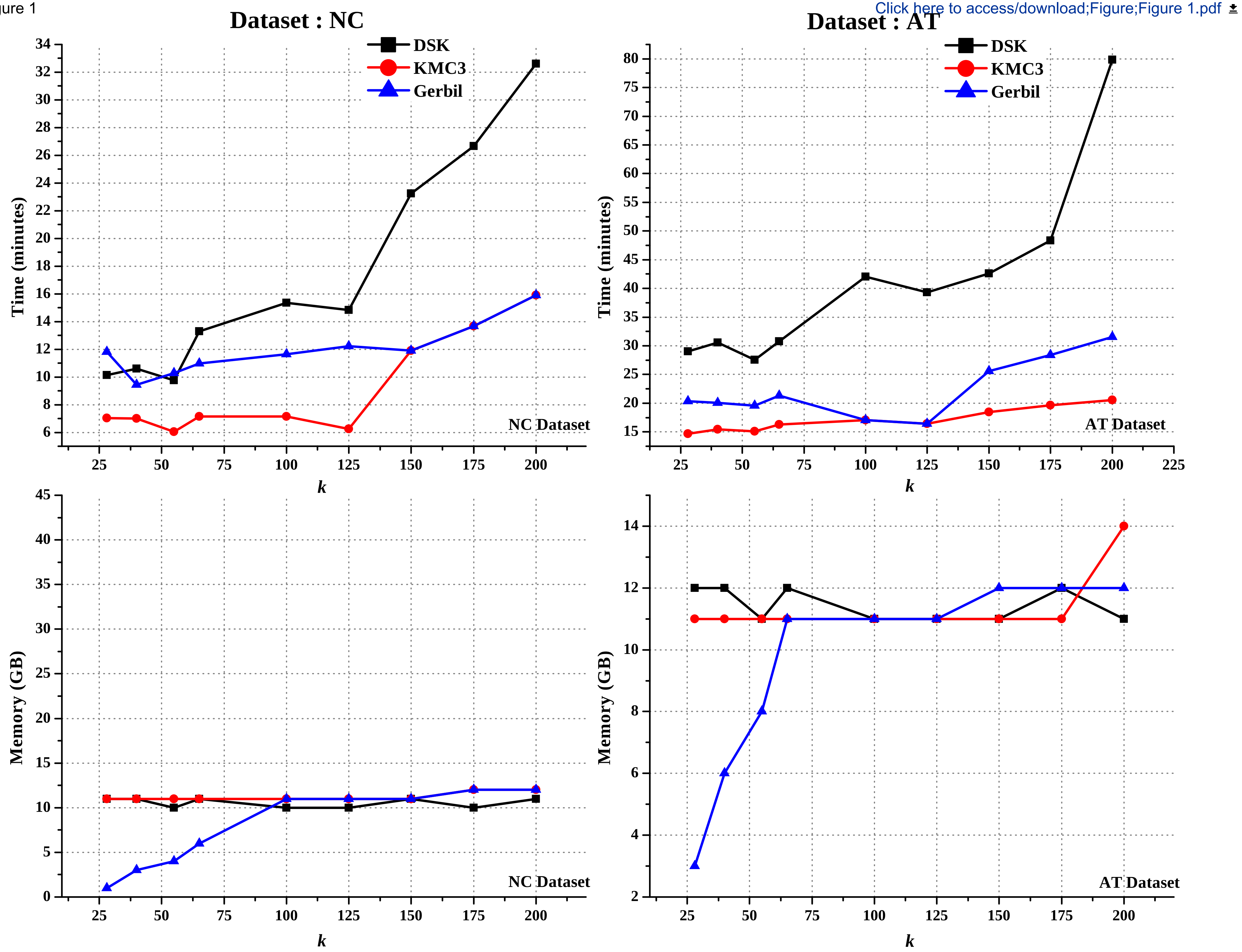

Figure 2

Click here to access/download;Figure;Figure 2.pdf

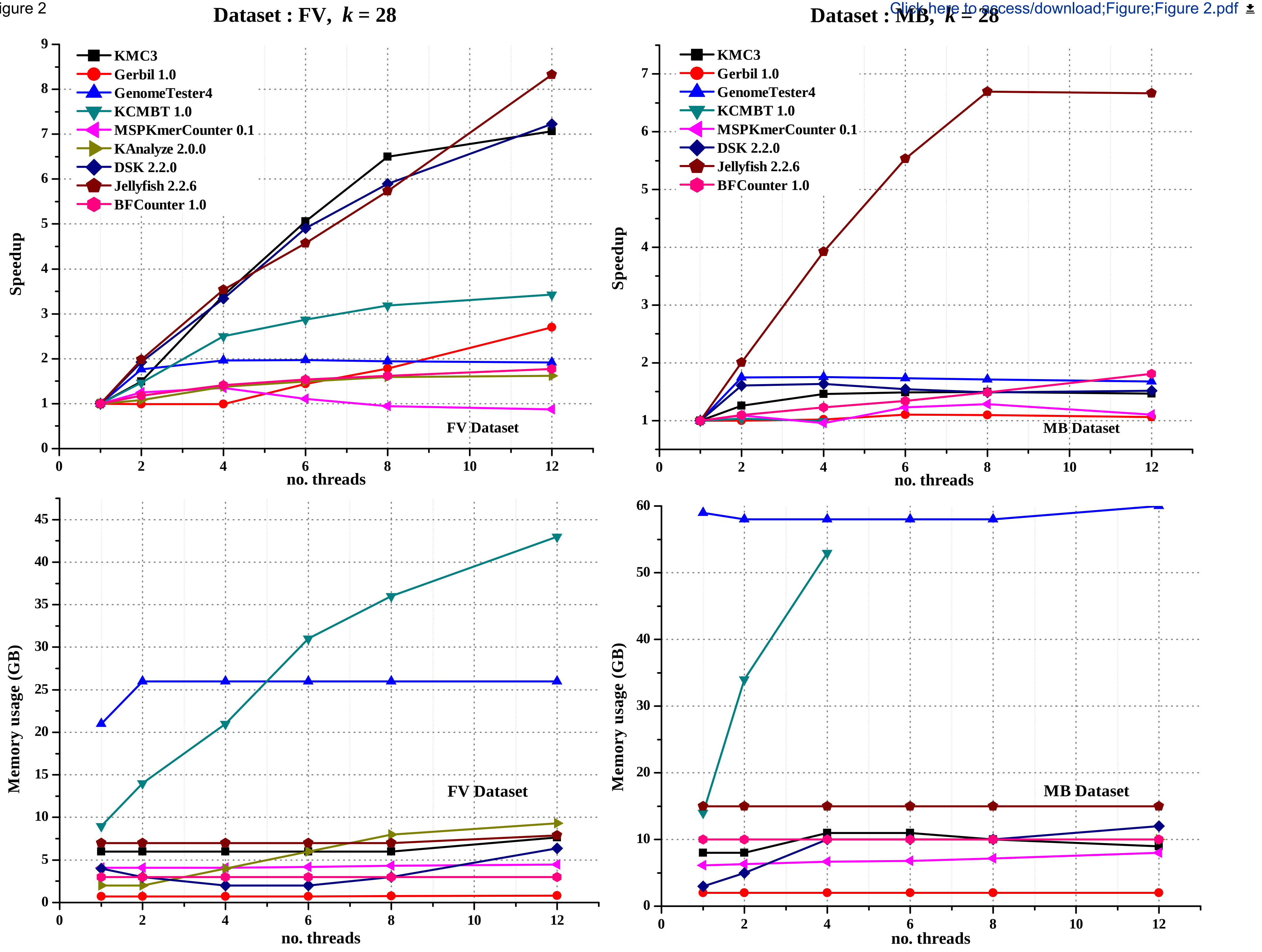

Appendix

Click here to access/download
Supplementary Material
Appendix_revised_4.doc

Click here to access/download
Supplementary Material
Supplimentary_revised_4.doc

script_disk_cpu

Click here to access/download
Supplementary Material
cpu_disk_util.sh

Click here to access/download
Supplementary Material
disk_max_cpu_avg.sh
