# Supplementary material for: A benchmark study of k-mer counting methods for high-throughput sequencing
Source: Gigascience. 2018 Oct 22;7(12):giy125. doi: 10.1093/gigascience/giy125 (PMC6280066; doi:10.1093/gigascience/giy125)
Supplement: Response_to_Reviewer_Comments_Original_Submission.pdf [file giy125_response_to_reviewer_comments_original_submission.pdf]

Author's Response To Reviewer Comments

Close

Reply Letter (GIGA-D-17-00245)
Title : A benchmark study of k-mer counting methods for high-throughput sequencing

Authors : Swati Chandrakant Manekar, M.Tech.; Shailesh Sathe, Ph.D.
Date: 20th February 2018

Dear Editors,
We gratefully acknowledge the status of our manuscript and your review result. We are thankful to you for the time and effort spent by the reviewers to provide us their valuable insights and helpful feedback. The comments received from the reviewers helped to significantly improve our manuscript. We have worked hard to suffice each of the review comments with our responses. We believe we have incorporated all the comments and suggestions made by the reviewers and enhanced our manuscript wherever necessary. In this letter we address the comments individually with suitable responses. The comments from the reviewers are indicated in italic bold, followed by our response in normal text for each of them.

Reviewer #1:

Comment 1.1
In the testing of the k-mer counting tools, k = 22 and 55 were used. But later k size with range from 28 to 200 was used to do the testing. Firstly, it will be nice if the authors can explain more about the applications of the counting of long k-mers like 150, 175, 200. In most situation in bioinformatics, we do not count the k-mer with such long length.
>>
We thank the reviewer for a detailed review. The applications for counting of long k-mers (for k = 150 to 200) are appended in the manuscript with related references.

Text Added in manuscript is on the page no.5 line no.16-23, page no.6 line no 1-3.
References are added to support the text added is on page no.33 line no.10-20.
>>

Comment 1.1.2
Secondly, if some of the k-mer counting tools make it clear that they do not support long k-mers counting, the authors may not need to actually test them and make them fail.
>>
We agree to the point raised herein, and hence, we have modified the text in this regards as KCMBT which does not support the longer k length has been removed from Figure 1 (old) titled “Analysis of time (second), memory (GB) and disk (GB) utilization of counting algorithms on AT and GT datasets for longer k length with, k = 28, 40, 55, 65, 100, 125, 150, 175 and 200” along with its description. Though the tools like BFCounter, KAnalyze 2.0.0 etc support long k-mer counting, they were unable to execute completely on AT and NC datasets. The reason possibly could be that they cannot handle longer length reads.
>>

Comment 1.2
One important application of k-mer counting is to get the frequency of a specific k-mer. It will be nice to test the performance of these tools to retrieve the frequencies of a group of k-mers. For example, this may be tested by retrieving the frequency of the first 100000 k-mers (or randomly selected k-mers) in a reads data set and measure the time, memory and disk usage of this process.
>>
Out of all the tools considered in this study, only five tools support the online retrieval of k-mer frequencies namely: 1) KCMBT 2) Jellyfish 3) MSPKmerCounter, 4) Tallymer and 5) Squeakr. These tools do not support the same forms of retrieval of the k-mers frequencies. For example:
1) KCMBT supports retrieval of a range of frequency of k-mers but not a k-mer or a group of k-mers.
2) MSPKmerCounter supports retrieval of a k-mer, one at a time but not a set of k-mers,
3) Jellyfish, Tallymer and Squeakr retrieve a set of k-mers.
Tallymer is not considered in our analysis, as we have only studied tools that were released after 2010. Squeakr is also not considered in this study as its exact k-mer counter code has not been released yet. Hence, we could not test the performance of these tools, to retrieve the frequencies of a group of k-mers. As these tools support retrieval of varying types of input, a fair comparison amongst them cannot be achieved. Secondly, there is only one tool, Jellyfish, out of the other tools in this study that support the retrieval of a frequency of a set of k-mers.
>>

Comment 1.3
Related to the previous points, it will be nice to have a more comprehensive table listing more details of each k-mer counting tools, like the algorithm/data structure used, if the tool support long k-mers, or the limit of k-size, if the tool support online k-mer frequency retrieval, etc.
>>
In the original manuscript the section titled "Overview of k-mer counting approaches" cover the information regarding data structure and the algorithm used by each tool. Hence the table covering comprehensive information of all the mentioned points as above is added in the supplementary (please see Table S1 along with its description).
We believe that the original table titled "Approaches for k-mer counting" is the best way to show the ontology of k-mer counting approaches, which categorizes different tools according to the underlying principles they use as well as the relationship between these approaches (especially for those tools spanning multiple approaches or borrowing / using many different ideas). Hence, we kept the original Table 1 as it is in the manuscript.
>>

Comment 1.4
In Tables such as Table 4, Table 5, Table 6, some tools have varying results compared to other tools. Are the results purely wrong? If it is, what is the reason? Is it because the tools do not support the experiment by design? Here more discussions may be needed since the accuracy of the counting of these tools is important information to the readers.
>>
As suggested by Reviewer #2, Table 4-7 are merged and now reflected in two Tables numbered as A1 and A2 in the appendix with the removal of redundant data. The varying results may be due to the bugs in the considered recent versions of the tools. Actually we cannot state the exact reason for the non-matching results as there can be n number of reasons like synchronization problem while multithreading, estimation of the size of an array, etc. at the implementation level of the various

approaches. We have taken special care while executing the tools by carefully setting all the parameters as per the guidelines given in the documentation of the tools. The answers to the above queries are reflected in the manuscript.

Text Added in manuscript is on the page no.18 line no.14-23, page no.19 line no 1-7.
>>

Comment 1.5
On Page 17, the authors made it clear that "The tools which give an approximation of k-mer counts histogram by streaming analysis of the data are not considered in this paper." However on Page 20 Line 14, the authors mentioned that "scTurtle is itself having some false positive results". What causes such false positive results? Is it some kind of approximation of k-mer counts? Does this contradict the previous statement?
>>
We agree to your point that scTurtle is not an exact k-mer counter. The scTurtle reports only an approximate count of the genuine k-mers because of its probabilistic nature (false positive results) owing to the underlying bloom filter it has used. Whereas BFCounter also uses a bloom filter, it makes one more pass to remove all the possibilities of false positive results (additional filtering to get exact results). To remove the contradiction as pointed out in the above comment, the scTurtle results are replaced by single threaded aTurtle (one of the variants of Turtle implementation) results as the aTurtle provides perfect counting. This benchmark study now considers only the exact k-mer counters. The paragraph related to aTurtle has been added to the manuscript.

Text Added in manuscript is on the page no.9 line no. 5-14.
>>

Comment 1.6
In Table2, "Genome size (M Base)", may be "M Bases".
>>
We have modified the above term in Table 2 titled as “Dataset specifications”, on page number 16.
>>

Comment 1.7
On Page3, the description of the k-mer counting problem is a little bit confusing, which may need be rewritten, like the sentence on Line 10, "Every read of set S indicated by r with r[i] accounts for every ith character of r with index starting at 0 to l-1, where l is the length of r. "
>>
To respond to the above comment, the paragraph explaining the k-mer counting process in the introduction section has been carefully revised, and the changes made in the manuscript are as follows,

Text Added in manuscript is on the page no.3 line no. 6-16.
>>

Reviewer #2:
Major remarks:

Comment 2.1

Another k-mer counting benchmark is cited: "Computational Performance Assessment of k-mer Counting Algorithms" from Perez et al, 2016. I will agree that the current manuscript is much more up to date with respect to the software and versions. Please nevertheless discuss whether your conclusions are in line with what Perez et al report. Please also recall what the conclusions of Perez et al are, in your introduction.

>>

Our conclusions are in line with Perez et al. who have reported on parameters like time, memory utilization and parallelization. However, we have also tested the performance of the tools; with respect to additional parameters e.g. impact of compressed input (as suggested by reviewer #3) and scalability of different tools for long k-mers and accuracy. We have also tested the performance of tools on a range of datasets from small (D. Melanogaster) to large (human). We have considered only the exact k-mer counters as opposed to stochastic k-mer counters to provide a fair comparison. These parameters not only provide a good assessment of the actual performance of the k-mer counting programs but also provide a clear picture of the current state of the art programs used for counting of k-mers. Our study also considers the latest tools to date. We have added the supporting text for each such additional parameter in the manuscript.

Text Added in manuscript in Introduction Section, page no.5 line no.1-16
Text Added in manuscript in Result and discussion Section, page no.28 line no.20-21
>>

Comment 2.2
The authors put much similar emphasis on the memory usage and disk usage as on running time. However one should note that, from a user perspective, when it comes to counting large datasets such as human, it does not matter so much if a tool uses 100 GB of disk or 150 GB of disk, as this resource is generally plentiful. Thus, I would have appreciated a more pragmatic viewpoint when it comes to comparing tools with respect to the various metrics.

>>

We have agreed to your point, and hence we have mainly focused on the prime parameters of time and memory usage instead of focusing on disk usages of any tools. To support this, we have removed the old figure numbered as 2 titled "Analysis of disk (GB) utilization of the disk based algorithms for k-mer counting for increasing value of k, k = 8 and k = 55" from the manuscript along with its description. For newly added parameters like the impact of the compressed input file and multithreading analysis, the disk utilization is not discussed.
We responded to this comment by adding the paragraphs in the manuscript which gives a more pragmatic viewpoint, to the comparison of tools concerning the various metrics.

Text is added in Abstract and Result and discussion section
Text Added in manuscript page no.5, line no3-16
>>

Comment 2.3
The middle of the results section, for instance the specific details regarding how each tool was run, should be moved to the appendix (a section or table of subsidiary matter at the end of a book or document.).

>>

Thank you very much for your valuable comment. The specific details regarding how each tool was run

have been moved to the appendix (page no.2, 3 and 4).
>>

Comment 2.4
Tables 4,5,6,7 too. The authors nicely spotted that MSPKmerCounter and Gerbil have bugs, apparently yet all these tables in the main text contain a lot of redundant information.
>>
We are grateful for your comment regarding Tables 4,5,6,7 and redundant data. We have merged Table 4, Table 5, Table 6 and Table 7(old) now and have prepared two tables (Table A1 and A2) in the appendix. We have also done our best to remove the redundant data.
As suggested by Reviewer #1 the scTurtle results have been replaced by single threaded aTurtle (one of the variants of Turtle implementation) results. We have used aTurtle to perform the benchmark study having only exact k-mer counters. The paragraph related to aTurtle is also added to the manuscript.
>>

Minor remarks:

Comment 2.1*
grammar/typo: "till" in Introduction, "approache" page 5.
>>
Typo errors have been corrected carefully.
>>

Comment 2.2*
Formatting, lines 17 and 19 page 3 e.g. "S = {{ACGTTA}, {ACGTTT}}.A": lack of space after comma. Also why is the set "s" a set of sets? Counting k-mer implies returning the set s = {ACGT, CGTT, GTTT}, as it does not matter which read the k-mer comes from.
>>
The paragraph in the introduction section that explains the k-mer counting process has been rewritten carefully as the same was also suggested by Reviewer #1 and is now reflected in the manuscript.

Text Added in manuscript is on the page no.3, line no. 6-16.
>>

Comment 2.3*
Page 4 line 10, reference [15] is Jellyfish, and it does not perform k-mer frequency statistics.
>>
We agree to your point on Jellyfish, and a proper sentence with proper citation is added to the manuscript as follows,

Text added in manuscript on page no.4, line no.2-3 and reference is added on page no.32, line no.35
>>

Comment 2.4*
Page 5, regarding the sentence "In the light of this, many heuristic techniques and 2 approaches have been implemented in the various research works". Not all k-mer counters are heuristics, in fact the majority of them return exact results.

>>
We agree with your point and hence removed the sentences creating confusion from the manuscript.
>>
Comment 2.5*
Page 5, the sentence lacks citations: "Many memory efficient data structures used by various researchers can be listed as, enhanced suffix array, burst trie, lock free hash table, membership query data structure like bloom filter, pattern block bloom filter, counting quotient filter (CQF) and so on". Although I realize each of these objects will be described later, it is confusing to list them all here.
>>
Sir, we agree with your comment. We have removed the sentence as mentioned above from the manuscript. Citations for each of the data structures referred have been added in manuscript at respective places.
Also to be in line with Reviewer #1 (Comment: to have a more comprehensive table listing more details of each k-mer counting tools, like the algorithm/data structure used, whether the tool supports long k-mers, or the limit of k-size, whether the tool support online k-mer frequency retrieval, etc.) we have added Table S1 titled 'Overview of various k-mer counting tools' in the supplementary.

Comment 2.6*
Page 5, please define "primary/secondary" memory.
>>
To maintain uniformity throughout, we have used the terms memory and disk instead of primary and secondary memory respectively. The line including the above terms is added in the manuscript.

Text added in manuscript on page no.4, line no.17-18.
>>

Comment 2.7*
Reference 24 is badly formatted.
>>
The said reference is now correctly formatted and added to the manuscript.

Reference added in manuscript on page no.33, line no.8-9
>>

Comment 2.8*
Page 5, the sentence "The presented benchmark study covers all the areas of evaluation where as the existing literature review is incomplete or inadequate" is quite vague.
>>
We removed the inconsistency by modifying and adding supporting sentences in the manuscript.

Text added in manuscript on page no.5, line no.1-15.
>>

Comment 2.9*
Page 5, the terms "capability to deal with enormous real world data" and "various categories" are vague.
>>

When we use the term “capability to deal with enormous real-world data” we want to suggest that “the scalability of tools for processing large datasets like human datasets has also been considered in this benchmark study”. However, we modified the sentence containing the above terms with an additional sentence to improve the manuscript.

Text added in manuscript on page no.5, line no.1-15.
>>

Comment 2.10*
GTester4 is actually named GenomeTester4. I would argue that the term "efficiently" in "efficiently uses the sort and count approach" page 6 is disputable, given that other tools also do that and yet are more efficient.
>>
We have modified the sentence containing the above terms to improve the manuscript.

Text added in manuscript on page no. 7, line no.1.
>>

Comment 2.11*
Page 7, the sentence "For k-mer counting, a typical data structure which can hold k-mer against its count is needed such as hash table [37] and is found suitable." reads awkwardly.
>>
We have modified the sentence containing the above terms to improve the manuscript.

Text added in manuscript on page no. 7, line no.8.
>>

Comment 2.12*
Page 7, the terms "proper hash function" and "best possible location" are vague.
>>
The sentence containing the above terms has been removed from the manuscript. To above the repetition, the paragraph is removed. Because the use of hash table for k-mer counting has already been coved in the subsequent paragraph.
>>

Comment 2.13*
Page 8 and onwards, there are several more minor remarks regarding the use of the English language as well as the precision of technical terms, but I would rather read a revised manuscript after it has been proof-read by a native speaker.
>>
We have rigorously worked on the suggestions recommended by you and have worked hard to improve the quality of written English and precision of technical terms throughout the manuscript.
>>

Technical remarks:
Comment 2.1**
Bloom should be capitalized.

>>
Bloom is capitalized in the manuscript at the respective places.
>>

Comment 2.2**
scTurtle is not an exact k-mer counter, please mention it.
>>
We have mentioned that scTurtle is not an exact k-mer counter. As suggested by Reviewer #1 (Comment: On Page 17, the authors made it clear that "The tools which give an approximation of k-mer counts histogram by streaming analysis of the data are not considered in this paper." However, on Page 20 Line 14, the authors mentioned that "scTurtle is itself having some false positive results". What causes such false positive results? Is it some kind of approximation of k-mer counts? Does this contradict the previous statement?) we have replaced results of scTurtle with single threaded aTurtle (one of the variants of Turtle implementation) as the aTurtle provides perfect counting.

Text Added in manuscript is on the page no.9 line no. 5-14.
>>

Comment 2.3** and 2.4**

2.3** : Page 9, "Ability to modify [..] k-mers" is inaccurate, k-mers are not "modified".

2.4** : The description of CQF is unclear. For instance: What are the values of the array? What does it mean for an array to have a "key"? (dictionaries have keys, not arrays). How is CQF "better" in terms of loss-less compression of k-mers than count-min sketches? (Count-min sketches do not compress k-mers at all and are not even exact data structures.) Why are CQFs sensitive to high counts? ("highly distorted" is not the right term)
>>
Necessary changes have been made in the sentence containing the above terms, and we have removed the vague terms from the paragraph, explaining the CQF.

Text added in manuscript on page no. 9, line no.15-20
>>

Comment 2.5**
Page 10: no, a burst trie is not a modified suffix trie.
>>
We agree with your point Sir, and the necessary change has been made in the sentence containing the above terms.

Text added in manuscript on page no.10, line no.18-19
>>

Comment 2.6**
The KCMBT section overall reads quite awkwardly, it needs to be revised carefully.
>>
We have carefully revised the KCMBT section, to improve the manuscript.

Text added in manuscript on page no.10, line no.18-22 and page no.11, line no.1-17
>>

Comment 2.7**
Page 11: no, DSK does not use a single large hash table. (it uses multiple ones, as k-mers are partitioned).
>>
The sentence corresponding to the above point is rewritten.

Text added in manuscript on page no.12, line no.11
>>

Comment 2.8**
MSPKC is actually MSPKmerCounter.
>>
MSPKC is replaced with MSPKmerCounter at respective places in the manuscript, appendix and supplementary.
>>

Comment 2.9**
Page 7: the same tool (khmer) is described 4 times.
>>
We accept your point, Sir. We have described the latest implementation/approach of khmer, published under the title “Efficient cardinality estimation for k-mers in large DNA sequencing datasets.” only in the manuscript. The old khmer implementations/approaches have been removed from the manuscript.

Text added in manuscript on page no. 16, line no.14-16
>>

Comment 2.10**
Note: DSK handles k > 127 when it is recompiled (as per the github readme).
>>
Sir, we recompiled the code and took the reading for k = 28,40, 55, 60,100,125,150,175 and 200 once again for datasets NC and AT, and the changes are reflected as shown in Figure No.1 on page no.28 along with the modified description in the manuscript.
>>

Reviewer #3
Comment 3.1
As a general, minor comment; though the manuscript is nicely laid out, the quality of the written English should be improved. There are a number of places with awkward phrasing, typos, etc.
>>
We have rigorously worked on the suggestions recommended by you and have worked hard to improve the quality of written English throughout the manuscript. We have also removed the awkward phrasing, typos, etc. as suggested by you.
>>

Major technical comments

Comment 3.1*
For example, the authors state that input datasets are decompressed and "concatenated" into a single input file before running each of the tools. While this may seem a reasonable approach to normalize for potential differences (e.g., if not all of the tools support decompression directly), it may have a non-trivial (significant.)effect on the results as they could deviate from how the tools might be used following best practices. Specifically, it is common to run most k-mer counting tools directly on the compressed files without first decompressing the reads. This has the benefit, especially on "traditional" hard drives, of improving the I/O throughput and allowing the actual counting algorithms to consume data faster.
This isbecause certain compression schemes like gzip have quite limited computational overhead, and so the cost of having to decompress the data in memory is overcome by the corresponding increase in data throughput that results from reading compressed data from disk. Since this is likely a common strategy for running k-mer counters, it should be included in the existing benchmarks (at least in addition to the current benchmarks).
Another potential issue stems from normalizing the data by concatenating the reads into a single file. Some k-mer counting tools (e.g. KMC2, though I am not entirely certain of the changes made in KMC3) effectively perform parallelization in their first phase by reading from individual input files using separate threads. This means that restricting the input to a single file effectively limits them to 1 or 2 threads (e.g., one to parse and one to bin / partition). Given KMC3 %CPU utilization numbers this may, indeed, be occurring. It looks as though parallelism effectively increases when KMC3 enters the second phase (that doesn't depend on input), but it would likely be able to do even better on multi-file inputs if those inputs were not first concatenated into a single file. This may also be true of other tools.
>>
Thank you, sir, for the above comment. The benchmarking of all the k-mer counting tools supporting compressed input is performed by running them on compressed input files (gzip/bz2), and the results are uploaded in table numbered as 4, 5, 6, 7 and 8. The related description has also been added in manuscript.

Text added in manuscript on page no.29 and line no. 2-18
>>

Comment 3.2*
My final concern is not, unfortunately, one where I am able to offer a great solution. That is, given the wide variation in adopted settings, it's not completely clear to me that the CPU resource comparisons are fair. For example, when different programs are executed with different numbers of threads, their running times aren't directly comparable. Moreover, given the difficulty in achieving "perfect scaling", it's not necessarily much better to examine non-wall-clock-time metrics. I realize that it is not always possible to compare all programs with exactly equal parameters --- as some programs, for example, restrict the specific number of threads that can / must be used. Perhaps, in this case, the best approach to analyzing CPU-related performance comprehensively is to run each program using a few configurations in terms of the number of threads. Though things may still not be directly comparable, it will at least give some insight into the scaling properties of the different tools with respect to the number of threads they are allowed to use.

>>
We agree to the suggestion made herein, and we have performed the benchmark study of all the tools using the following number of threads (thread = 1, 2, 4, 6, 8 and 12) on two different datasets, FV and MB. The results of which (speedup and memory plot) are shown in the figure no. 2 and the corresponding readings are added to the supplementary document (Table S8 – S9). The related description is added in the manuscript.

Text added in manuscript on page no. 29 and 30
>>

Comment 3.3*
As an aside, when certain in-memory tools were not able to complete a task in the specified time or resources (e.g., running out of memory), I wonder if the authors considered the strategies such programs had to minimize RAM usage. For example, Jellyfish 2 supports filtering "erroneous" k-mers using a Bloom filter. This capability is mentioned in the introduction. However, I wonder if the authors attempted to use this feature when Jellyfish 2 exceeded the allocated resources when processing certain datasets. It is possible that other programs also expose such options. Perhaps it is reasonable to avoid benchmarking all tools in such "non-standard" configurations, nonetheless, it would be useful to know if it is at least possible to allow such tools to complete processing these datasets on the benchmarking machine.
>>
Thank you, sir, for your valuable comment. As on our benchmarking machine Jellyfish in its default mode could not execute on HS1. Hence, we tried to run Jellyfish in BF-based mode on HS1 for both values of k (28 and 55). But as Jellyfish's - BF-based mode for phase 1 could not finish execution within 15 hours for both k values and as the system froze, we had to terminate its execution forcefully.
Jellyfish was also tested in the BF-based mode for dataset MB (M. balbisiana). Where its performance degraded regarding time (by 21% for k = 28 and 45% for k = 55) and memory (by 25% for k = 28 and 45% for k = 55) as compared to the Jellyfish's default mode. The results of execution for Jellyfish in BF-based mode on dataset MB for k = 28 and k = 55 are added in supplementary documents.

Text added in manuscript on page no. 20 , line no.1-3
>>

Close
