# Supplementary material for: A benchmark study of k-mer counting methods for high-throughput sequencing
Source: Gigascience. 2018 Oct 22;7(12):giy125. doi: 10.1093/gigascience/giy125 (PMC6280066; doi:10.1093/gigascience/giy125)
Supplement: Response_to_Reviewer_Comments_Revision_1.pdf [file giy125_response_to_reviewer_comments_revision_1.pdf]

Author's Response To Reviewer Comments

Close

We are grateful to all reviewers for encouraging and guiding us for improving the technical content as well as the English writing style of manuscript. The suggestions offered by the reviewers have been immensely helpful. We have worked hard to suffice each of the review comments with our responses. Most of the revisions prompted by the reviewers' comments are related to English grammar. We have incorporated all the comments and suggestions made by the reviewers and enhanced our manuscript wherever necessary. Regarding more minor matters, we have now changed our phrasing patterns to be more precise. We have taken help from the highly qualified native English speaking editors at American Journal Experts also. They further edited our manuscript for proper English language, grammar, punctuation, spelling, and overall style. We address the comments individually with suitable responses as follows,

Reviewer #1:

Comment 1.1
The authors made great efforts to address the issues brought up by the reviewers. The authors rewrote many parts in the manuscript to make it more clear and precise. The new table S1 is very helpful. Now I think that all of my previous concerns with the manuscript
have been addressed.
>>
We thank the reviewer for a detailed review and encouragement.
>>
Reviewer #2:

The authors have done extensive corrections and responded to all my comments in a satisfactory fashion, except one: I had recommended that the paper gets an extensive proof-reading by a competent English speaker, yet there are still numerous mistakes.

Major remarks:

Comment 1.1
Reading the first two pages, part of the results and the conclusion, it is apparent that the usage of English is still largely flawed throughout the paper.
>>
We thank the reviewer for a detailed review. We believe that we have incorporated all the suggestions to correct English usage in our manuscript and thus enhanced our manuscript wherever necessary.
>>

Comment 1.2
The "Result and discussion" has become very long (12 pages with figures), please use subsections.
>>
We agree to your point that "Result and discussion" has become very long; hence we have divided it into 5 subsections as listed below,
1. Runtime, memory and disk usage

2. Performance for larger values of k
3. Scalability to varying sizes of datasets
4. The impact of compressed input
5. Scalability on the number of threads
>>

English mistakes found on page 1:
Comment 2.1
"A read say r"
-> "a read r"
Comment 2.2
“Let R denote the dataset having n number of reads such that R = {ri; 1 ≤ i ≤ n}."
-> "having n reads"
>>
We have modified the sentences related to the above mistakes. Similarly we have removed the unnecessary/extra words from the entire manuscript to improve English usage.
>>

Comment 2.3
"a sequence of nucleotide"
-> "a sequence of nucleotides"
>>
‘Plural’ and ‘singular’ terms are taken care of throughout the manuscript to improve English usage.
>>

Comment 2.4
"denotes alphabet "
-> "denotes the alphabet" or "denotes an alphabet"
>>
Articles in English, i.e. ‘a/an’ and ‘the’ are used properly at proper places in the entire manuscript to improve English grammar usage.
>>

Comment_2.5
"applied in the de novo genome assembly viz., the overlap layout consensus approach [3, 4] and the de Bruijn graph [5-8] based assembly"
-> "applied in de novo genome assembly, e.g. using the overlap layout consensus approach [3,4] or the de Bruijn graph approach [5-8]".
>>
Appropriate terms with proper punctuations are used at the appropriate places throughout the manuscript to improve the manuscript. The sentences are rephrased to grammatically correct usage while keeping the meanings intact in the manuscript.
>>

Comment_2.6
"The probable misalignment in the reads is either due to errors or genuine nucleotide variations can be estimated using k-mer frequencies"

-> This sentence does not make sense to me. What is "the misalignment" in this context, is it related to multiple sequence alignment or error-correction or both? Could you give a citation that shows an approach that perform such "misalignment" estimation task? (but I did not understand what this task is)
>>
We agree to the point raised herein and hence we have modified the text in this regard and the proper citation is thus given. In this context “the misalignment" is related to error-correction.
Modified Text:
For instance, k-mer frequencies are used to assess a probable misalignment among reads, which is either a sequencing error or a genuine nucleotide variation [10].
Citation:
10. Kelley DR, Schatz MC, Salzberg SL. Quake: quality-aware detection and correction of sequencing errors. Genome Biol. 2010;11:R116. https://doi.org/10.1186/gb-2010-11-11-r116
>>

on page 2:
Comment 3.1
"de novo repeat annotation techniques like ReAS makes"
-> "make"
Comment 3.2
"billions of next-generation sequencing (NGS) data needs to be processed"
-> "billions [..] need"
Comment 3.3
page 25: "it pass" -> it passes
Comment 3.4
page 31: "several gigabytes of genomic data is generated." -> are generated
>>
Singular verbs and plural verbs are carefully and correctly used in the entire manuscript.
>>

Comment 3.4
"high-frequency k-mer as a seed"
-> "high-frequency k-mers as seeds"
Comment 3.5
"to annotate repetitive plant genome"
-> "to annotate repetitive plant genomes"
>>
We have incorporated the above changes in the corresponding sentences. The sentences carrying similar types of mistakes are modified to make them technically correct in the manuscript.
>>

Comment 3.6
"disked based"
-> "disk based" throughout the paper
>>
“disked based” is replaced by “disk-based” throughout the paper.
>>

Comment 3.7
pages 9 and 12: "till" should be replaced by "until"
>>
Till is replaced by until at the places wherever is needed (correct prepositions and conjunctions). Similar types of appropriate words are used to make the sentence more appropriate in the paper.
>>

Comment 3.8
page 19: "within15 hours" space missing
>>
All the formatting mistakes are rectified throughout the paper including the suggested one.
>>

Comment 3.9
page 26: "When it's time " -> When its time
>>
Possessive pronoun is properly used in the paper to make the sentences meaningful.
>>

Comment 3.10
page 26: "[..], it is still remarkable" -> this is a non-quantitative personal judgement. Same for "astonishing" in the next sentence.
>>
We have modified the sentences in a way that these will not be reflecting a non-quantitative personal judgement and the modified sentences are as follows:
Interestingly, Gerbil is consistently the most memory and disk frugal approach. For most of the datasets, the disk utilization of Gerbil is the lowest, but it is slower than KMC3 (The GPU implementation of Gerbil was not considered). Gerbil tries to reduce the utilization of disk and memory, revealing a result that is both memory and disk economical.
>>

Note: this is not an exhaustive list of style corrections for the paper, just a few that I spotted.
>>
We made all the English usage corrections throughout the manuscripts as pointed out and likewise in the comments. Our manuscript has been edited for proper English language, grammar, punctuation, spelling, and overall style by the highly qualified native English speaking editors at American Journal Experts.
>>

Close
