# Supplementary material for: A benchmark study of k-mer counting methods for high-throughput sequencing
Source: Gigascience. 2018 Oct 22;7(12):giy125. doi: 10.1093/gigascience/giy125 (PMC6280066; doi:10.1093/gigascience/giy125)
Supplement: Response_to_Reviewer_Comments_Revision_2.pdf [file giy125_response_to_reviewer_comments_revision_2.pdf]

Author's Response To Reviewer Comments

Close

We are very thankful for giving acceptance to our manuscript with minor revision. We appreciate the time and effort spent by the reviewer to provide us his valuable insight and helpful feedback. The comments received from the reviewer helped to significantly improve our manuscript. We believe we have incorporated all the comments and suggestions made by the reviewer and enhanced our manuscript where necessary. We address the comments individually with suitable responses as follows,

Reviewer #2:

Comment 2.1
page 3: "For instance, k-mer frequencies are used to assess a probable misalignment among reads, which is either a sequencing error or a genuine nucleotide variation [10]."
-> I am still still unsure about that sentence, as I couldn't find where in the Quake paper is this matter discussed. Could you please point it to me, or remove this sentence? It is not clear to me whether any alignment "among reads" are being made, but instead, alignment between reads and a reference genome.
>>
We thank the reviewer for a detailed review. We agree to the point raised herein and hence we have removed this sentence from the manuscript as suggested by the reviewer.
>>

Comment 2.2
page 4: "Statistics on the number of frequencies of all the k-mers"
->The meaning of "number of frequencies" is unclear.
>>
In this sentence, "number of frequencies" is replaced with "number of occurrences" in the manuscript.
>>

Comment 2.3
page 4: "use arrays with substring {k-mer} indexing."
->How is a flat array indexed by substrings? isn't it rather a dictionary?
>>
We agree to the point raised herein and hence we have modified the text in this regard.
Modified Text:
A naive approach for k-mer counting is to use a dictionary, with k-mers as keys and their counts as values.
>>

Comment 2.4
page 4: "the approach will soon overwhelm"
->More definitive wording can be used, I suggest "the approach overwhelms".
>>
"the approach will soon overwhelm" is modified to "the approach overwhelms".
>>

Comment 2.5
page 4: "a magnitude"
->"an order of magnitude"
>>
We have replaced "a magnitude" with "an order of magnitude" in the sentence on line number 18 and page number 4.
>>

Comment 2.6
page 5: "The disk-based approaches achieve very high efficiency at a marginal increase in the cost."
->"Cost" in what resource?
>>
Cost is in regard of I/O. As opposed to disk-based approach, in-memory approach puts everything in memory and therefore involves almost no I/O costs [this sentence is referred from Jellyfish {Memory} article]. We have modified the text in this regard in the manuscript.
Modified Text:
The disk-based approaches achieve very high efficiency at a marginal increase in the I/O costs.
>>

Comment 2.7
page 5: Just a remark; on top of what is written there, long reads are also notoriously used at resolving repetition in genome assemblies, not just structural variants.
>>
We agree to the point raised herein and hence we have modified the text in this regard.
Modified Text:
The long reads are used at resolving repetition in genome assemblies; they also facilitate better resolution of structural variants present in DNA samples and genomic repeat content [27], along with many other advantages [26].
>>

Comment 2.8
page 5: "Large values of k {k values up to 200} facilitate improvement of the accuracy of long sequencing reads {particularly of repeat-overlapping reads} and contig assembly [28]."
->Please note that this article isn't about "long reads" in the PacBio/Nanopore sense, but rather slightly longer Illumina reads.
>>
We agree to the point raised herein and hence we have modified the text in this regard.
Modified Text:
Large values of k {k values up to 200} facilitate improvement of the accuracy of longer Illumina reads {particularly of repeat-overlapping reads} and contig assembly [28].
>>

Comment 2.9
page 6: "highest N50 are obtained at an optimal choice of k, which seems to be larger values of k"
->No, not necessarily for larger values of k. {Only when there is sufficiently high coverage.}
>>

We agree to the point raised herein and hence we have modified the text in this regard.
Modified Text:
Empirically, the best assemblies {without miss-assembly} and the highest N50 {only when there is sufficiently high coverage} are obtained at an optimal choice of k, which seems to be larger values of k [29, 30].
>>

Comment 2.10
page 7 typo: "reprove"
>>
We have incorporated the change in the corresponding sentence as suggested above and hence reprove is replaced with reprobe.
>>

Comment 2.11
page 7: "Jellyfish follows a 'quotienting' technique"
->This seems to me that this quotienting technique is just a regular insertion procedure in a hash table.
>>
We agree to the point raised herein and hence we have removed the text in this regard as it has already been covered in the subsequent sentences.
>>

Comment 2.12
page 8: I suggest that the original Turtle algorithms may be presented in "k-mer counting using the sorting approach" for greater clarity that it is indeed a sorting-based algorithm.
>>
We agree to the point that Turtle follows a sorting-based approach and hence Turtle algorithms are now presented in "k-mer counting using the sorting approach" for greater clarity along with the proper rearrangement of references.
>>

Comment 2.13
page 9: "The k-mers are hashed using a one-way hash function,"
->I understand where this sentence is coming from, but the general concept of Squeakr could in principle also use an invertible hash function {which is in fact the main ingredient in Squeakr-exact}.
>>
We agree to the point raised herein and hence we have modified the text in this regard.
Modified Text:
Squeakr {exact}, which counts the frequency of each k-mer exactly using an invertible hash function, is not considered in this benchmark study, as its code is not available yet {currently not suitable for this benchmark study}.
>>

Comment 2.14
page 10: "Burst trie manages large sets [..]"; grammar, perhaps revise to "Burst tries manage large sets [..]"
>>

We have modified the corresponding sentence as suggested herein. ‘Burst trie manages’ is replaced by ‘Burst tries manage’.
>>

Comment 2.15
page 10: "extended k-mers, similar to KMC2", for historical accuracy, please mention that {k+x}-mers were introduced by KMC2.
>>
We have added the sentence as suggested by the reviewer in the manuscript as follows.
Modified Text:
Extended k-mers {{k + x}-mers for x > 0} are substrings of length more than k and were introduced by KMC2.
>>

Comment 2.16
page 11: "uses hashing approach for k-mer counting such as DSK" grammar, please revise to "uses a hashing approach for k-mer counting similar to DSK".
>>
We agree to the point raised herein and hence we have modified the text in this regard.
Modified Text:
Gerbil [31] uses a hashing approach for k-mer counting similar to DSK.
>>

Comment 2.17
page 11: what is a "part hash function" ?
>>
Actually it is ‘partHash’, not ‘part hash’. Hence, we have replaced ‘part hash’ with ‘partHash’ along with appropriate citation. The partHash function is explained in the ‘Gerbil’ article {Page No. 4}.
>>

Comment 2.18
page 11: "The algorithm avails high parallelization." I am unsure if this is correct in English.
>>
We agree to the point raised herein and hence we have modified the text in this regard.
Modified Text:
The algorithm is designed to make optimal use of the hardware with multiple threads running concurrently.
>>

Comment 2.19
page 17: "adopted" typo -> "adapted" and the beginning of the sentence "The commands used to run all the programs" is repeated twice
>>
Corrections suggested herein are reflected in the manuscript and repetition is now removed.
Modified Text:
The commands used to run all the programs were adapted from their documentation and the publications of KMC3 and KMC2. These commands are given in the Supplementary Material.

>>

Comment 2.20
page 17: "are available with the histogram subroutine." is awkward wording {- "can create histograms directly"}
>>
Corrections suggested herein are reflected in the manuscript and hence ‘are available with the histogram subroutine’ is replaced with ‘can create histograms directly’.
Modified Text:
Some tools, such as Jellyfish, DSK, Gerbil and MSPKmerCounter can create histograms directly.
>>

Comment 2.21
page 18: "The results are not entirely wrong for [..]," is vague
>>
We agree to the point raised herein and hence we have modified the text in this regard to make it precise.
Modified Text:
Not all the k-mers with their counts in the outputs of Turtle, MSPKmerCounter and Gerbil {only for k = 55} are matching with the outputs of the other tools, for respective datasets.
>>

Comment 2.22
note: in my version of the manuscript, Figures 1 & 2 are blurry because in JPG format, they should be uploaded in EPS or PDF format
>>
Figures 1 & 2 are now uploaded in PDF format with better clarity.
>>

Comment 2.23
page 29: "in such large set of reads {massive datasets}" {redundant formulation}
>>
Redundant formulation has been removed from the sentence as suggested herein.
Modified Text:
There is a need to continue the development of a system that realizes memory and time trade-off for k-mer counting in such large set of reads.
>>

Note: Since we are not able to submit 'Respond to Reviewers' having "opening parenthesis" , hence we have used "curly braces" in above responses.

Close
