# Supplementary material for: A benchmark study of k-mer counting methods for high-throughput sequencing
Source: Gigascience. 2018 Oct 22;7(12):giy125. doi: 10.1093/gigascience/giy125 (PMC6280066; doi:10.1093/gigascience/giy125)
Supplement: Response_to_Reviewer_Comments_Revision_3.pdf [file giy125_response_to_reviewer_comments_revision_3.pdf]

Author's Response To Reviewer Comments

Close

Dear editor,

We appreciate the time and effort spent by the copy editor for careful copy-editing and proofreading the manuscript. Many thanks for care and attention to detail by the most impressive copy editor and the amount of work you put into this manuscript which made it a much better review article. We have incorporated all the comments and suggestions made by the copy editor and accepted all the track changes. We address the comments individually with suitable responses as follows,

Comment [SE1]: Do you have an ORCID too? Is it 0000-0002-5297-2018
>>
Yes and it is 0000-0002-5297-2018
>>

Comment [LM2]: Please insert the ORCID of the second author.
>>
Inserted the ORCID of the second author in the manuscript as follows,
ORCID IDs: Shailesh R. Sathe: 0000-0002-5297-2018
>>

Comment [LM3]: Possibly not needed in the Abstract – delete?
>>
The line is deleted from the Abstract.
All k-mer counting programs evaluated in this article are freely available and can be downloaded from their respective websites.
>>

Comment [LM4]: Disjointed?
>>
Disjoint is correct as we have checked from the source document for 'Minimum Substring Partitioning' (MSPKmerCounter). Hence we have kept 'Disjoint' as it is (Line No. 15, Page No.11).The referred line from manuscript of MSPKmerCounter is as follows,

'MSP breaks short reads into multiple disjoint partitions such that each partition can be loaded into memory and processed individually.'
>>

Comment [LM5]: To me, it makes more sense to describe the tools you are going to test first, and then describe the datasets you are going to run on them, so I have moved some paragraphs around here and amended the subheading slightly.
>>
We agree to the point raised herein and hence accepted the format changes.
>>

Comment [LM6]: I have deleted some of the detail in this paragraph as I felt it was too much detail about something that is not discussed in the paper!
>>
We agree and accepted the format changes. The references are changed accordingly.
[61(khmer library)] is now removed from the manuscript.
>>

Comment [LM7]: The URL should be listed in the references list. Please update the reference numbering accordingly.
>>
The URL is now listed in the references list and the reference numbering is updated accordingly.
[61] https://gist.github.com/netj/526585/36515c55a3b25232bddfdd51e43cadc1a5f296de.
>>

Comments [SE8] [SE9]: Can we get a copy of this?
>>
The scripts are uploaded (as supplementary files) with the revised documents. The script 'cpu_disk_util.sh' which captures the disk usage and % CPU utilization has been written by referring the script by Jaeho Shin, licensed under the Apache License, Version 2.0 (the "License"), http://www.apache.org/licenses/LICENSE-2.0. The script 'dsk_max_cpu_avg.sh' gives the disk usage values (peak and minimum) and average over the %CPU utilizations captured by 'cpu_disk_util.sh'. To calculate the maximum disk used by the program, subtract the three values given by the script 'dsk_max_cpu_avg.sh', as shown below,
The maximum disk used by the program = Peak disk used - Disk used before the start of program execution - The file size of output file generated by the tool.
>>

Comments [LM10] [LM11] [LM12] [LM13] [LM14]: This should be expressed as GB, in line with the other figures in this column.
>>
We agree & now expressed figures which were in MBs in GBs as follows,
Table 4: Gerbil, RAM , k=25 : 848 MB* => 0.83*
Table 4: Gerbil(gzip), RAM , k=25 : 841 MB => 0.82
Table 5: KMC3, Disk , k=50 : 614.4 MB* => 0.6*
Table 5: Gerbil, RAM , k=25 : 827 MB* => 0.81*, k=50 : 826 MB* => 0.81*
Table 5: Gerbil(gzip), RAM , k=25 : 837 MB => 0.82, k=50 : 834 MB => 0.81

Hence removed MB = megabytes from Table footnotes.

Comment [SE15]: Please include abbreviations here
>>
RAM: Random Access Memory; NGS: Next-generation Sequencing; SAC: Sort-and-compact; CQF: Counting Quotient Filter; MSP: Minimum Substring Partitioning; FV: F. Vesca; DM: D. Melanogaster; MB: M. Balbisiana; HS1: H. sapiens 1; HS2: H. sapiens 2; NC: N. Crassa; AT: A. thaliana
>>

Comment [LM16]: Please ensure that the references are formatted according to GigaScience's preferred

style.
>>
We have ensured that the references are formatted according to GigaScience’s preferred style.
>>

Close
