# Supplementary material for: A benchmark study of k-mer counting methods for high-throughput sequencing
Source: Gigascience. 2018 Oct 22;7(12):giy125. doi: 10.1093/gigascience/giy125 (PMC6280066; doi:10.1093/gigascience/giy125)
Supplement: Reviewer_1_Report_(Original_Submission) -- Qingpeng Zhang — 11/4/2017 Reviewed [file giy125_reviewer_1_report_(original_submission).pdf]

Reviewer Report

Title: A benchmark study of k-mer counting methods for high-throughput sequencing

Version: Original Submission **Date:** 11/4/2017

Reviewer name: Qingpeng Zhang

Reviewer Comments to Author:

In this manuscript, the authors tested the performance of the various k-mer counting tools and compared the memory, time, disk usage of each tool. In general, the manuscript was well written, with the results from the benchmarking of the tools comprehensively explained, though there are some points to improve.1. In the testing of the k-mer counting tools, k = 22 and 55 were used. But later k size with range from 28 to 200 was used to do the testing. Firstly, it will be nice if the authors can explain more about the applications of the counting of long k-mers like 150, 175, 200. In most situation in bioinformatics, we do not count the k-mer with such long length. Secondly, if some of the k-mer counting tools make it clear that they do not support long k-mers counting, the authors may not need to actually test them and make them fail.2. One important application of k-mer counting, is to get the frequency of a specific k-mer. It will be nice to test the performance of these tools to retrieve the frequencies of a group of k-mers. For example, this may be tested by retrieving the frequency of the first 100000 k-mers (or randomly selected k-mers) in a reads data set and measure the time, memory and disk usage of this process.3. Related to the previous points, it will be nice to have a more comprehensive table listing more details of each k-mer counting tools, like the algorithm/data structure used, if the tool support long k-mers, or the limit of k-size, if the tool support online k-mer frequency retrieval, etc. 4. In Tables such as Table 4, Table 5, Table 6, some tools have varying results compared to other tools. Are the results purely wrong? If it is, what is the reason? Is it because the tools do not support the experiment by design? Here more discussions may be needed since the accuracy of the counting of these tools is important information to the readers.5. On Page 17, the authors made it clear that "The tools which give an approximation of k-mer counts histogram by streaming analysis of the data are not considered in this paper." However on Page 20 Line 14, the authors mentioned that "scTurtle is itself having some false positive results". What causes such false positive results? Is it some kind of approximation of k-mer counts? Does this contradict the previous statement?6. In Table2, "Genome size (M Base)", may be "M Bases".7. On Page3, the description of the k-mer counting problem is a little bit confusing, which may need be rewritten, like the sentence on Line 10, "Every read of set S indicated by r with r[i] accounts for every ith character of r with index starting at 0 to l-1, where l is the length of r. "

Level of Interest

Please indicate how interesting you found the manuscript: An article of importance in its field

Quality of Written English

Please indicate the quality of language in the manuscript: Acceptable

Declaration of Competing Interests

Please complete a declaration of competing interests, considering the following questions:

- Have you in the past five years received reimbursements, fees, funding, or salary from an organisation that may in any way gain or lose financially from the publication of this manuscript, either now or in the future?
- Do you hold any stocks or shares in an organisation that may in any way gain or lose financially from the publication of this manuscript, either now or in the future?
- Do you hold or are you currently applying for any patents relating to the content of the manuscript?
- Have you received reimbursements, fees, funding, or salary from an organization that holds or has applied for patents relating to the content of the manuscript?
- Do you have any other financial competing interests?
- Do you have any non-financial competing interests in relation to this paper?

If you can answer no to all of the above, write 'I declare that I have no competing interests' below. If your reply is yes to any, please give details below.

I declare that I have no competing interests

I agree to the open peer review policy of the journal. I understand that my name will be included on my report to the authors and, if the manuscript is accepted for publication, my named report including any attachments I upload will be posted on the website along with the authors' responses. I agree for my report to be made available under an Open Access Creative Commons CC-BY license (http://creativecommons.org/licenses/by/4.0/). I understand that any comments which I do not wish to be included in my named report can be included as confidential comments to the editors, which will not be published.

I agree to the open peer review policy of the journal

To further support our reviewers, we have joined with Publons, where you can gain additional credit to further highlight your hard work (see: https://publons.com/journal/530/gigascience). On publication of this paper, your review will be automatically added to Publons, you can then choose whether or not to claim your Publons credit. I understand this statement.

Yes
