# Supplementary material for: A benchmark study of k-mer counting methods for high-throughput sequencing
Source: Gigascience. 2018 Oct 22;7(12):giy125. doi: 10.1093/gigascience/giy125 (PMC6280066; doi:10.1093/gigascience/giy125)
Supplement: Reviewer_2_Report_(Original_Submission) -- Rayan Chikhi — 11/13/2017 Reviewed [file giy125_reviewer_2_report_(original_submission).pdf]

Reviewer Report

Title: **A benchmark study of k-mer counting methods for high-throughput sequencing**

Version: **Original Submission** **Date:** 11/13/2017

Reviewer name: **Rayan Chikhi**

Reviewer Comments to Author:

This manuscript is a review of recent k-mer counters. The description of the tools is passable overall. In some places, could be made less vague and there are some factual inaccuracies (I list some specific ones below). I recognize also that it is difficult to estimate what is the right level of details to give for each k-mer counter. Where the article shines is in its benchmark section. The authors did a painstaking job at reporting relevant results for all the k-mer counters. They even commented on the CPU usage across execution of tools. The benchmark looks good to me, the tools behaved somewhat as I expected. As it is, the manuscript really needs one more good pass of proof-reading by a native speaker, and another pass by a senior computer scientist. I therefore recommend a major revision but would be happy to review a revised version later, as I believe that such a benchmark will be valuable.signed, Rayan ChikhiMajor remarks:* Another k-mer counting benchmark is cited: "Computational Performance Assessment of k-mer Counting Algorithms" from Perez et al, 2016. I will agree that the current manuscript is much more up to date with respect to the software and versions. Please nevertheless discuss whether your conclusions are in line with what Perez et al report. Please also recall what the conclusions of Perez et al are, in your introduction.* The authors put much similar emphasis on the memory usage and disk usage as on running time. However one should note that, from a user perspective, when it comes to counting large datasets such as human, it does not matter so much if a tool uses 100 GB of disk or 150 GB of disk, as this resource is generally plentiful. Thus, I would have appreciated a more pragmatic viewpoint when it comes to comparing tools with respect to the various metrics.* The middle of the results section, for instance the specific details regarding how each tool was run, should be moved to the appendix. * Tables 4,5,6,7 too. The authors nicely spotted that MSPKmerCounter and Gerbil have bugs, apparently.. yet all these tables in the main text contain a lot of redundant information.Minor remarks:grammar/typo: "till" in Introduction, "approache" page 5.* Formatting, lines 17 and 19 page 3 e.g. "S = {{ACGTTA},{ACGTTT}}.A": lack of space after comma. Also why is the set "s" a set of sets? Counting k-mer implies returning the set s = {ACGT, CGTT, GTTT}, as it does not matter which read the k-mer comes from. The following sentence is awkward: "A 4-mer is a 4-character long substring of every such reads in input set S is obtained as set.* Page 4 line 10, reference [15] is Jellyfish, and it does not perform k-mer frequency statistics* Page 5, regarding the sentence "In the light of this, many heuristic techniques and 2 approaches have been implemented in the various research works". Not all k-mer counters are heuristics, in fact the majority of them return exact results.* Page 5, the sentence lacks citations: "Many memory efficient data structures used by various researchers can be listed as, enhanced suffix array, burst trie, lock free hash table, membership query data structure like bloom filter, pattern block bloom filter, counting quotient filter (CQF) and so on". Although I realize each of these objects will be described later, it is confusing to list them all here.* Page 5, please define "primary/secondary" memory.* Reference 24 is badly formatted.* Page 5, the sentence "The presented benchmark study covers all the areas of evaluation where as the existing literature review is incomplete or inadequate" is quite vague.* Page 5, the terms "capability to deal with enormous real world data" and "various categories" are vague.* GTester4 is actually named GenomeTester4. I would argue that the term "efficiently" in "efficiently uses the sort and count approach" page 6 is disputable, given that other tools also do that and yet are more efficient.* Page 7, the sentence "For k-mer counting, a typical data structure which can hold k-mer against its count is needed such as hash table [37] and is found suitable." reads awkwardly.* Page 7, the terms "proper hash function" and "best possible location" are vague.Page 8 and onwards, there are several more minor remarks regarding the use of the English language as well as the precision of technical terms, but I would rather read a revised manuscript after it has been proof-read by a native speaker. I'll now focus on more technical remarks.*

Bloom should be capitalized.* scTurtle is not an exact k-mer counter, please mention it.* Page 9, "Ability to modify [..] k-mers" is inaccurate, k-mers are not "modified".* The description of CQF is unclear. For instance: What are the values of the array? What does it mean for an array to have a "key"? (dictionaries have keys, not arrays). How is CQF "better" in terms of loss-less compression of k-mers than count-min sketches? (Count-min sketches do not compress k-mers at all and are not even exact data structures.) Why are CQFs sensitive to high counts? ("highly distorted" is not the right term)* Page 10: no, a burst trie is not a modified suffix trie.* The KCMBT section overall reads quite awkwardly, it needs to be revised carefully.* Page 11: no, DSK does not use a single large hash table. (it uses multiple ones, as k-mers are partitioned)* MSPKC is actually MSPKmerCounter.* Page 7: the same tool (khmer) is described 4 times.* Note: DSK handles k > 127 when it is recompiled (as per the github readme).

Level of Interest

Please indicate how interesting you found the manuscript: An article whose findings are important to those with closely related research interests

Quality of Written English

Please indicate the quality of language in the manuscript: Not suitable for publication unless extensively edited

Declaration of Competing Interests

Please complete a declaration of competing interests, considering the following questions:

- Have you in the past five years received reimbursements, fees, funding, or salary from an organisation that may in any way gain or lose financially from the publication of this manuscript, either now or in the future?
- Do you hold any stocks or shares in an organisation that may in any way gain or lose financially from the publication of this manuscript, either now or in the future?
- Do you hold or are you currently applying for any patents relating to the content of the manuscript?
- Have you received reimbursements, fees, funding, or salary from an organization that holds or has applied for patents relating to the content of the manuscript?
- Do you have any other financial competing interests?
- Do you have any non-financial competing interests in relation to this paper?

If you can answer no to all of the above, write 'I declare that I have no competing interests' below. If your reply is yes to any, please give details below.

I declare that I have no competing interest

I agree to the open peer review policy of the journal. I understand that my name will be included on my report to the authors and, if the manuscript is accepted for publication, my named report including any attachments I upload will be posted on the website along with the authors' responses. I agree for my report to be made available under an Open Access Creative Commons CC-BY license (http://creativecommons.org/licenses/by/4.0/). I understand that any comments which I do not wish to be included in my named report can be included as confidential comments to the editors, which will not be published.

I agree to the open peer review policy of the journal

To further support our reviewers, we have joined with Publons, where you can gain additional credit to further highlight your hard work (see: https://publons.com/journal/530/gigascience). On publication of this paper, your review will be automatically added to Publons, you can then choose whether or not to claim your Publons credit. I understand this statement.

Yes
