# Supplementary material for: A benchmark study of k-mer counting methods for high-throughput sequencing
Source: Gigascience. 2018 Oct 22;7(12):giy125. doi: 10.1093/gigascience/giy125 (PMC6280066; doi:10.1093/gigascience/giy125)
Supplement: Reviewer_2_Report_(Revision_1) -- Rayan Chikhi — 3/14/2018 Reviewed [file giy125_reviewer_2_report_(revision_1).pdf]

Reviewer Report

Title: A benchmark study of k-mer counting methods for high-throughput sequencing

Version: Revision 1 Date: 3/14/2018

Reviewer name: Rayan Chikhi

Reviewer Comments to Author:

The authors have done extensive corrections and responded to all my comments in a satisfactory fashion, except one: I had recommended that the paper gets an extensive proof-reading by a competent English speaker, yet there are still numerous mistakes.Major remarks:1. Reading the first two pages, part of the results and the conclusion, it is apparent that the usage of English is still largely flawed throughout the paper.2. The "Result and discussion" has become very long (12 pages with figures), please use subsections.English mistakes found on page 1:"A read say r"-> "a read r""a sequence of nucleotide"-> "a sequence of nucleotides""denotes alphabet "-> "denotes the alphabet" or "denotes an alphabet"" Let R denote the dataset having n number of reads such that R = {ri; 1 ≤ i ≤ n}."-> "having n reads""applied in the de novo genome assembly viz., the overlap layout consensus approach [3, 4] and the de Bruijn graph [5-8] based assembly"-> "applied in de novo genome assembly, e.g. using the overlap layout consensus approach [3,4] or the de Bruijn graph approach [5-8]"."The probable misalignment in the reads is either due to errors or genuine nucleotide variations can be estimated using k-mer frequencies"-> This sentence does not make sense to me. What is "the misalignment" in this context, is it related to multiple sequence alignment or error-correction or both? Could you give a citation that shows an approach that perform such "misalignment" estimation task? (but I did not understand what this task is)on page 2:"de novo repeat annotation techniques like ReAS makes"-> "make""high-frequency k-mer as a seed"-> "high-frequency k-mers as seeds""to annotate repetitive plant genome"-> "to annotate repetitive plant genomes""billions of next-generation sequencing (NGS) data needs to be processed"-> "billions [..] need""disked based"-> "disk based" throughout the paperpages 9 and 12: "till" should be replaced by "until"page 19: "within15 hours" space missingpage 25: "it pass" -> it passespage 26: "When it's time " -> When its timepage 26: "[..], it is still remarkable" -> this is a non-quantitative personal judgement. Same for "astonishing" in the next sentence.page 31: "several gigabytes of genomic data is generated." -> are generatedNote: this is not an exhaustive list of style corrections for the paper, just a few that I spotted.

Level of Interest

Please indicate how interesting you found the manuscript: An article whose findings are important to those with closely related research interests

Quality of Written English

Please indicate the quality of language in the manuscript: Not suitable for publication unless extensively edited

Declaration of Competing Interests

Please complete a declaration of competing interests, considering the following questions:

- Have you in the past five years received reimbursements, fees, funding, or salary from an organisation that may in any way gain or lose financially from the publication of this manuscript, either now or in the future?
- Do you hold any stocks or shares in an organisation that may in any way gain or lose financially from the publication of this manuscript, either now or in the future?
- Do you hold or are you currently applying for any patents relating to the content of the manuscript?
- Have you received reimbursements, fees, funding, or salary from an organization that holds or has applied for patents relating to the content of the manuscript?
- Do you have any other financial competing interests?
- Do you have any non-financial competing interests in relation to this paper?

If you can answer no to all of the above, write 'I declare that I have no competing interests' below. If your reply is yes to any, please give details below.

I declare that I have no competing interests

I agree to the open peer review policy of the journal. I understand that my name will be included on my report to the authors and, if the manuscript is accepted for publication, my named report including any attachments I upload will be posted on the website along with the authors' responses. I agree for my report to be made available under an Open Access Creative Commons CC-BY license (http://creativecommons.org/licenses/by/4.0/). I understand that any comments which I do not wish to be included in my named report can be included as confidential comments to the editors, which will not be published.

I agree to the open peer review policy of the journal

To further support our reviewers, we have joined with Publons, where you can gain additional credit to further highlight your hard work (see: https://publons.com/journal/530/gigascience). On publication of this paper, your review will be automatically added to Publons, you can then choose whether or not to claim your Publons credit. I understand this statement.

Yes
