# Supplementary material for: A benchmark study of k-mer counting methods for high-throughput sequencing
Source: Gigascience. 2018 Oct 22;7(12):giy125. doi: 10.1093/gigascience/giy125 (PMC6280066; doi:10.1093/gigascience/giy125)
Supplement: Reviewer_3_Report_(Original_Submission) -- Rob Patro — 11/22/2017 Reviewed [file giy125_reviewer_3_report_(original_submission).pdf]

**Reviewer Report**

**Title: A benchmark study of k-mer counting methods for high-throughput sequencing**

**Version: Original Submission** **Date:** 11/22/2017

**Reviewer name: Rob Patro**

**Reviewer Comments to Author:**

Manekar and Sathe survey the current state of the literature (and available tools) related to the problem of k-mer counting. The manuscript begins with a rather nice background section that defines the problem and then touches upon many of the advancements made in tackling this problem with ever-more-efficient solutions. The current k-mer counting approaches are divided into categories based on the strategies they take for solving the problem --- though the boundaries between different approaches isn't always entirely clear. Actually, I think that an overview figure corresponding to the "ontology of k-mer counting approaches", categorizing the different tools mentioned according to the underlying principles they use as well as show the relationship between these approaches (especially for those tools spanning multiple approaches or borrowing / using many different ideas). The manuscript then describes a fairly wide-ranging benchmark of many different popular k-mer counters, showing how their resource utilization (in terms of CPU, RAM and disk) scale with different data sets, k-mer sizes, etc. This turns out to be a useful benchmark, as general "broad scale" conclusions seem to be reasonably drawn. For example, Gerbil and KMC3 represent good defaults as they have consistently good performance metrics with well-controlled resource usage among a wide range of inputs and parameters. As a general, minor comment; though the manuscript is nicely laid out, the quality of the written English should be improved. There are a number of places with awkward phrasing, typos, etc.My major technical comments regard the methodology used to prepare the input datasets as well as the comparison based on metrics (e.g., wall clock time) that might not always be comparable given the different parameter settings required by different tools. For example, the authors state that input datasets are decompressed and "concatenated" into a single input file before running each of the tools. While this may seem a reasonable approach to normalize for potential differences (e.g., if not all of the tools support decompression directly), it may have a non-trivial effect on the results as they could deviate from how the tools might be used following best practices. Specifically, it is common to run most k-mer counting tools directly on the compressed files without first decompressing the reads. This has the benefit, especially on "traditional" hard drives, of improving the I/O throughput and allowing the actual counting algorithms to consume data faster. This is because certain compression schemes like gzip have quite limited computational overhead, and so the cost of having to decompress the data in memory is overcome by the corresponding increase in data throughput that results from reading compressed data from disk. Since this is likely a common strategy for running k-mer counters, it should be included in the existing benchmarks (at least in addition to the current benchmarks).Another potential issue stems from normalizing the data by concatenating the reads into a single file. Some k-mer counting tools (e.g. KMC2, though I am not entirely certain of the changes made in KMC3) effectively perform parallelization in their first phase by reading from individual input files using separate threads. This means that restricting the input to a single file effectively limits them to 1 or 2 threads (e.g., one to parse and one to bin / partition). Given KMC3 %CPU utilization numbers this may, indeed, be occurring. It looks as though parallelism effectively increases when KMC3 enters the second phase (that doesn't depend on input), but it would likely be able to do even better on multi-file inputs if those inputs were not first concatenated into a single file. This may also be true of other tools.My final concern is not, unfortunately, one where I am able to offer a great solution. That is, given the wide variation in adopted settings, it's not completely clear to me that the CPU resource comparisons are fair. For example, when different programs are executed with different numbers of threads, their running times aren't directly comparable. Moreover, given the difficulty in achieving "perfect scaling", it's not necessarily much better to examine non-wall-clock-time metrics. I realize that it is not always possible to compare all programs with exactly equal parameters --- as some programs, for example, restrict

the specific number of threads that can / must be used. Perhaps, in this case, the best approach to analyzing CPU-related performance comprehensively is to run each program using a few configurations in terms of the number of threads. Though things may still not be directly comparable, it will at least give some insight into the scaling properties of the different tools with respect to the number of threads they are allowed to use.As an aside, when certain in-memory tools were not able to complete a task in the specified time or resources (e.g., running out of memory), I wonder if the authors considered the strategies such programs had to minimize RAM usage. For example, Jellyfish 2 supports filtering "erroneous" k-mers using a Bloom filter. This capability is mentioned in the introduction. However, I wonder if the authors attempted to use this feature when Jellyfish 2 exceeded the allocated resources when processing certain datasets. It is possible that other programs also expose such options. Perhaps it is reasonable to avoid benchmarking all tools in such "non-standard" configurations, nonetheless, it would be useful to know if it is at least possible to allow such tools to complete processing these datasets on the benchmarking machine.Overall, this is a nice review article, and presents a useful summary of the current state of the field. However, some improvements could be made to both the quality of the text as well as the benchmarking methodology itself.

**Level of Interest**

Please indicate how interesting you found the manuscript: An article whose findings are important to those with closely related research interests

**Quality of Written English**

Please indicate the quality of language in the manuscript: Needs some language corrections before being published

**Declaration of Competing Interests**

Please complete a declaration of competing interests, considering the following questions:

- Have you in the past five years received reimbursements, fees, funding, or salary from an organisation that may in any way gain or lose financially from the publication of this manuscript, either now or in the future?
- Do you hold any stocks or shares in an organisation that may in any way gain or lose financially from the publication of this manuscript, either now or in the future?
- Do you hold or are you currently applying for any patents relating to the content of the manuscript?
- Have you received reimbursements, fees, funding, or salary from an organization that holds or has applied for patents relating to the content of the manuscript?
- Do you have any other financial competing interests?
- Do you have any non-financial competing interests in relation to this paper?

If you can answer no to all of the above, write 'I declare that I have no competing interests' below. If your reply is yes to any, please give details below.

I declare that I have no competing interests

I agree to the open peer review policy of the journal. I understand that my name will be included on my report to the authors and, if the manuscript is accepted for publication, my named report including any attachments I upload will be posted on the website along with the authors' responses. I agree for my report to be made available under an Open Access Creative Commons CC-BY license (http://creativecommons.org/licenses/by/4.0/). I understand that any comments which I do not wish to be included in my named report can be included as confidential comments to the editors, which will not be published.

I agree to the open peer review policy of the journal

To further support our reviewers, we have joined with Publons, where you can gain additional credit to further highlight your hard work (see: https://publons.com/journal/530/gigascience). On publication of this paper, your review will be automatically added to Publons, you can then choose whether or not to claim your Publons credit. I understand this statement.

Yes
