# Supplementary material for: A benchmark study of k-mer counting methods for high-throughput sequencing
Source: Gigascience. 2018 Oct 22;7(12):giy125. doi: 10.1093/gigascience/giy125 (PMC6280066; doi:10.1093/gigascience/giy125)
Supplement: Supplemental Files [file giy125_supplemental_files.zip › Appendix_revised_4.doc]

**Dataset information**

For each dataset listed in Table 2, ‘Genome size’ information was collected from the NCBI website (‘https://www.ncbi.nlm.nih.gov/genome/browse/’), and ‘Total number of bases’ was collected from ‘https://www.ncbi.nlm.nih.gov/sra/?’. The total number of reads in the given FASTA/FASTQ file was calculated using the Linux utilities *grep* and *awk* (‘grep -c "^>" dataset.fasta’, for FASTA format and ‘awk '{s++}END{print s/4}' dataset.fastq’, for FASTQ format).

Sequence data (input dataset) available as SRA data were converted to FASTA/FASTQ format using the SRA toolkit utility ‘fastq-dump’ (using the commands ‘./fastq-dump SRR_no.sra’ and SRR_no.sra --fasta’). FASTQ/FASTA files were then concatenated into a single file. The SRA toolkit is available at: ‘https://trace.ncbi.nlm.nih.gov/Traces/sra/sra.cgi?view=software’.

**Specific details about how each tool was run**

All of the tools considered in our study, except for BFCounter, Jellyfish, aTurtle and MSPKmerCounter, have automated parameter selection for most of the program parameters. BFCounter, Jellyfish, aTurtle and MSPKmerCounter might be improved by including automated parameter selection.

Jellyfish requires the size of the hash table to be pre-specified. In the documentation, it is recommended that the size is set to 10% more than the total estimated number of unique *k*-mers. Hence, for each dataset, hash table sizes were set to 10% more than the total number of unique *k*-mers given by KMC3. The same approach was taken for BFCounter. aTurtle also requires a unique number of *k*-mers to be specified (including the singletons) to select the size of the array. Therefore, the sizes were set to 10% more than the number of unique *k*-mers, as given by KMC3 for each dataset. For KAnalyze, the parameter ‘threads’ in the *k*-mer generation step (- *l*) and spilt step (-*d*) was set to 6 for each step. For Gerbil, KMC3 and DSK, the maximum amount of memory (RAM) was set to 12 GB for each dataset. However, they did not use the full amount of memory allocated to them. KCMBT requires the number of threads to be in the order of two. We configured our machine to 8 threads, since this gave the best performance with respect to memory and time requirements. For MSPKmerCounter, the average length of reads must be set accurately otherwise it might not generate output. While running MSPKmerCounter, the number of blocks and minimum substring length were set according to the documentation. The rest of the parameters for each tool were set as per the guidelines provided by the tool.

The runtime reported for each tool does not include the time required for the dump subroutine. It is considered that sequencing errors cause singleton *k*-mers to appear in a read library. Each tool was run ensuring that it would consider only the canonical representation of *k*-mers, and count *k*-mers occurring more than once. The option to use the canonical form is explicitly specified by passing a parameter for Jellyfish (-*C*) and KAnalyze (-*rcanonical*), but other tools consider canonical *k*-mers as default. For KAnalyze and aTurtle, time reported represents the time taken to count both singleton and non-singleton *k*-mers, because there is no option to exclude singleton *k*-mers. For the remaining tools, the time taken to count only non-singleton *k*-mers (*k*-mers occurring more than once) is reported. Of the ten programs considered in this study, only KAnalyze and aTurtle generate output in human-readable format. Therefore, for the remaining programs, we ran an additional step, i.e., the dump subroutine, to be able to generate *k*-mers and their counts in a human-readable format. The current versions of all the tested programs skip *k*-mers that contain the character ‘*N*’, which frequently occurs in NGS data.

MSPKmerCounter generates output in two phases: a partition phase and a count phase. Its actual runtime is reported by summing the time taken for each phases. Memory usage is reported by taking the maximum memory utilized by each phase. Similarly, disk usage is reported by taking the maximum disk utilized by each phase. CPU utilization is reported by calculating the average CPU utilized by each phase.

**Table A1** 28-mer and 55-mer frequencies generated by each program for the FV dataset

| ***k*-mer freq.** | **Frequency abundance for *k* = 28** | | | **Frequency abundance for *k* = 55** | | | |
| --- | --- | --- | --- | --- | --- | --- | --- |
|  | **Jellyfish 2.2.6, DSK 2.2.0, KAnalyze 2.0.0, KMC3,**  **Gerbil 1.0,**  **KCMBT 1.0,**  **GenomeTester4, BFCounter 1.0** | **MSPKmer**  **Counter 0.1** | **aTurtle 0.3** | **Jellyfish 2.2.6, DSK 2.2.0, KAnalyze 2.0.0, KMC3, BFCounter 1.0** | **Gerbil 1.0** | **MSPKmer-Counter 0.1** | **aTurtle**  **0.3** |
| 1 | 363420998 | **69066437 *** | **363421125*** | 652176330 | **79102 *** | **39121998 *** | **652176422*** |
| 2 | 49080695 | **18115152 *** | **49080694*** | 76579833 | **35492642 *** | **7250617 *** | **76579834*** |
| 3 | 18049815 | **5639542 *** | **18049820*** | 28281157 | **13566991 *** | **2084150 *** | **28281161*** |
| 4 | 11150813 | **2208574 *** | **11150811*** | 18211721 | **12986541 *** | **832867 *** | **18211720*** |
| 5 | 9843691 | **1091696 *** | **9843693*** | 16401875 | **8473838 *** | **414097 *** | **16401874*** |
| 6 | 10378494 | **629357 *** | **10378494*** | 16723214 | **8615812 *** | **227106 *** | **16723216*** |
| 7 | 11430920 | **390902 *** | **11430919*** | 17266403 | **8908818 *** | **131829 *** | **17266403*** |
| 8 | 12495242 | **259176 *** | **12495241*** | 17420694 | **12170688 *** | **82141 *** | **17420693*** |
| 9 | 13289486 | **185636 *** | **13289487*** | 16983119 | **8661491 *** | **52736 *** | **16983119*** |
| 10 | 13661314 | **144101 *** | **13661314*** | 16005955 | **8144646 *** | **37158 *** | **16005956*** |

Entries in bold with * indicate varying results. KCMBT 1.0 and GenomeTester4 do not support higher values of *k*. BFCounter 1.0 does not provide the count for singleton *k*-mers. Abbreviation: freq. = frequency.

**Table A2** 28-mer and 55-mer frequencies generated by each program for the DM dataset

| ***k*-mer freq.** | **Frequency abundance for *k* = 28** | | | **Frequency abundance for *k* = 55** | | |
| --- | --- | --- | --- | --- | --- | --- |
|  | **Jellyfish 2.2.6,**  **DSK 2.2.0, KAnalyze 2.0.0, KMC3,**  **Gerbil 1.0,**  **KCMBT 1.0, GenomeTester4, BFCounter 1.0** | **MSPKmerCounter 0.1** | **aTurtle 0.3** | **Jellyfish 2.2.6,**  **DSK 2.2.0, KAnalyze 2.0.0, KMC3,**  **BFCounter 1.0,**  **aTurtle0.3** | **Gerbil 1.0** | **MSPKmerCounter 0.1** |
| 1 | 155281400 | **146698167 *** | **155281818*** | 132512502 | **45255 *** | **127152484 *** |
| 2 | 8864191 | **8707861 *** | **8864201*** | 15209916 | **2025688 *** | **15386744 *** |
| 3 | 4905173 | **4980239 *** | **4905177*** | 14350714 | **1926881 *** | **14551570 *** |
| 4 | 4626001 | **4790519 *** | **4625998*** | 14070907 | **4197271*** | **14200127 *** |
| 5 | 5117297 | **5329251 *** | **5117299*** | 12981796 | **1679797 *** | **13035273 *** |
| 6 | 5763542 | **5981513 *** | **5763541*** | 11361229 | **1458782 *** | **11342261 *** |
| 7 | 6302198 | **6510090 *** | **6302198*** | 9542344 | **1232507 *** | **9474395 *** |
| 8 | 6658595 | **6838360 *** | **6658591*** | 7807048 | **2659122 *** | **7709343 *** |
| 9 | 6817573 | **6963587 *** | **6817577*** | 6272227 | **808026 *** | **6169544 *** |
| 10 | 6799383 | **6918078 *** | 6799383 | 4979037 | **634728 *** | **4880327 *** |

Entries in bold with * indicate varying results. KCMBT 1.0 and GenomeTester4 do not support higher values of *k*. BFCounter 1.0 does not provide the count for singleton *k*-mers. Abbreviation: freq. = frequency.
