# Supplementary material for: A benchmark study of k-mer counting methods for high-throughput sequencing
Source: Gigascience. 2018 Oct 22;7(12):giy125. doi: 10.1093/gigascience/giy125 (PMC6280066; doi:10.1093/gigascience/giy125)
Supplement: Supplemental Files [file giy125_supplemental_files.zip › Supplimentary_revised_4.doc]

**Table S1** Overview of programs implementing the *k*-mer counting approaches discussed in the manuscript

| ***k*-mer counting tool (year of publication) [citation]** | **(i) Data structure**  **(ii) Algorithm (sorting/hashing, disk-based/in-memory)** | **Limit on *k* size** | **Supports online *k*-mer frequency retrieval?** | **Supports compressed file processing?** |
| --- | --- | --- | --- | --- |
| KMC3 (2017) [37] | (i) Array, priority queue  (ii) Sorting, disk-based approach, modified MSP (signature), radix sort, counting sort | Arbitrary large *k*-mer lengths | No | Yes |
| Gerbil 1.0 (2017) [31] | (i) Array  (ii) Hashing, disk-based, modified MSP (signature), GPU implementation | < 521 | No | Yes |
| GenomeTester4 (2015) [38] | (i) Custom hybrid data structure, array  (ii) Sorting, disk-based | < 33 | No | No |
| KCMBT 1.0 (2016) [43] | (i) Burst tries  (ii) Sorting, in-memory, radix sort | < 33 | Yes | No |
| MSPKmerCounter 0.1 (2015) [32] | (i) Hash table  (ii) Hashing, disk-based, MSP (minimizer) | Arbitrary large *k*-mer lengths | Yes | No |
| Turtle 0.3 (2014) [42] | (i) Pattern block Bloom filter, array  (ii) Sorting, in-memory, SAC | < 65 | No | No |
| KAnalyze 2.0.0 (2014) [40] | (i) Array  (ii) sorting, disk-based, dual-pivot quick sort (Java’s Arrays.sort()) | Arbitrary large *k*-mer lengths | No | Yes |
| DSK 2.2.0 (2013) [33] | (i) Hash table  (ii) Sorting, disk-based approach, radix sort | Arbitrary large *k*-mer lengths | No | Yes |
| Jellyfish 2.2.6 (2011) [35] | (i) Hash table  (ii) Hashing, in-memory, Jellyfish-BF(mode) (i) Bloom filter  (ii) Disk-based | Arbitrary large *k*-mer lengths | Yes | No |
| BFCounter 1.0 (2011) [36] | (i) Bloom filter, hash table  (ii) Hashing, in-memory | Arbitrary large *k*-mer lengths | No | Yes |
| Squeakr (2017) [34] | (i) CQF  (ii) Hashing, in-memory | Arbitrary large *k*-mer lengths | Yes | Yes |
| Tallymer (2008) [22] | (i) Enhanced suffix arrays  (ii) Counting using the lcp-interval tree, in-memory | Arbitrary large *k*-mer lengths | Yes | No |

Abbreviations: MSP = minimum substring partitioning, SAC = sort and compaction, CAS = compare and swap, CQF = counting quotient filter, lcp = longest common prefix

One important application of *k*-mer counting is to obtain the frequency of a specific *k*-mer. In general, most *k*-mer counters do support frequency retrieval of a *k*-mer or a set of *k*-mers. Fast frequency retrieval is suitable for many bioinformatics applications, e.g., in de Bruijn graph traversal [1] or computing inner products between datasets [2]. Of all the tools mentioned in this study, only five support the online retrieval of *k*-mer frequencies: KCMBT, Jellyfish, MSPKmerCounter, Tallymer and Squeakr. However, they do not do so in the same ways, e.g.:

1) KCMBT supports retrieval of a range of frequencies of *k*-mers but not for a *k*-mer or a group of *k*-mers.

2) MSPKmerCounter supports retrieval of a *k*-mer one at a time but not for a set of *k*-mers.

3) Jellyfish, Tallymer and Squeakr support retrieval for a *k*-mer and a set of *k*-mers.

**Table** **S2 *k*-mer counting tools and their software URLs**

| **Tool** | **Software URL** |
| --- | --- |
| KMC3 | http://sun.aei.polsl.pl/REFRESH/kmc |
| Gerbil | https://github.com/uni-halle/gerbil |
| Squeakr | https://github.com/splatlab/squeakr |
| KCMBT | https://github.com/abdullah009/kcmbt_mt |
| MSPKmerCounter | http://www.cs.ucsb.edu/~yangli/MSPKmerCounter |
| GenomeTester4 | https://github.com/bioinfo-ut/GenomeTester4 |
| Turtle | http://bioinformatics.rutgers.edu/Software/Turtle and  http://figshare.com/articles/Turtle/791582 |
| KAnalyze | https://sourceforge.net/projects/kanalyze/ |
| DSK | http://minia.genouest.org/dsk, https://github.com/GATB/dsk |
| Jellyfish | http://www.cbcb.umd.edu/software/jellyfish  http://www.genome.umd.edu/jellyfish.html  https://github.com/gmarcais/Jellyfish |
| BFCounter | http://web.stanford.edu/group/pritchardlab/bfcounter.html  https://github.com/pmelsted/BFCounter |
| Squeakr | https://github.com/splatlab/squeakr/tree/exact) |
| Tallymer | https://github.com/dbolser/PGSC/tree/master/kmer-filter  https://www.zbh.unihamburg.de/forschung/arbeitsgruppegenominformatik/software/tallymer.html |

**Table S3** Error rates (percent errors) of MSPKmerCounter 0.1 and aTurtle 0.3 reported in the ‘Error%’ column for the FV dataset at *k* = 28. The ‘DSK 2.2.0’ column reports exact *k*-mer counts.

| ***k*-mer frequency** | **DSK 2.2.0** | **MSPKmer**  **Counter 0.1** | **Error%** | **aTurtle 0.3** | **Error%** |
| --- | --- | --- | --- | --- | --- |
| 1 | 363420998 | 69066437 | 426.19 | 363421125 | 0.00003 |
| 2 | 49080695 | 18115152 | 170.94 | 49080694 | 0.00000 |
| 3 | 18049815 | 5639542 | 220.06 | 18049820 | 0.00003 |
| 4 | 11150813 | 2208574 | 404.89 | 11150811 | 0.00002 |
| 5 | 9843691 | 1091696 | 801.69 | 9843693 | 0.00002 |
| 6 | 10378494 | 629357 | 1549.06 | 10378494 | 0.00000 |
| 7 | 11430920 | 390902 | 2824.24 | 11430919 | 0.00001 |
| 8 | 12495242 | 259176 | 4721.14 | 12495241 | 0.00001 |
| 9 | 13289486 | 185636 | 7058.89 | 13289487 | 0.00001 |
| 10 | 13661314 | 144101 | 9380.37 | 13661314 | 0.00000 |
| Average | | | 2755.75 | Average | 0.00001 |
| Maximum | | | 9380.37 | Maximum | 0.00003 |

**Table S4** Error rates (percent errors) of Gerbil 1.0, MSPKmerCounter 0.1 and aTurtle 0.3 reported in the ‘Error%’ column for the FV dataset at *k* = 55. The ‘DSK 2.2.0’ column reports exact *k*-mer counts.

| ***k*-mer frequency** | **DSK 2.2.0** | **Gerbil 1.0** | **Error%** | **MSPKmerCounter 0.1** | **Error%** | **aTurtle0.3** | **Error%** |
| --- | --- | --- | --- | --- | --- | --- | --- |
| 1 | 652176330 | 79102 | 459333.39 | 39121998 | 828.94 | 652176422 | 0.00001 |
| 2 | 76579833 | 35492642 | 38.28 | 7250617 | 576.92 | 76579834 | 0.00000 |
| 3 | 28281157 | 13566991 | 33.04 | 2084150 | 766.05 | 28281161 | 0.00001 |
| 4 | 18211721 | 12986541 | 14.14 | 832867 | 1238.85 | 18211720 | 0.00001 |
| 5 | 16401875 | 8473838 | 16.17 | 414097 | 2277.15 | 16401874 | 0.00001 |
| 6 | 16723214 | 8615812 | 20.46 | 227106 | 4469.89 | 16723216 | 0.00001 |
| 7 | 17266403 | 8908818 | 28.31 | 131829 | 8571.02 | 17266403 | 0.00000 |
| 8 | 17420694 | 12170688 | 2.67 | 82141 | 15111.94 | 17420693 | 0.00001 |
| 9 | 16983119 | 8661491 | 53.43 | 52736 | 25100.03 | 16983119 | 0.00000 |
| 10 | 16005955 | 8144646 | 67.73 | 37158 | 36665.47 | 16005956 | 0.00001 |
| Average | | | 45960.76 | Average | 9560.63 | Average | 0.00001 |
| Maximum | | | 459333.4 | Maximum | 36665.47 | Maximum | 0.00001 |

**Table S5** Error rates (percent errors) of MSPKmerCounter 0.1 and aTurtle 0.3 reported in the ‘Error%’ column for the DM dataset at *k* = 28. The ‘DSK 2.2.0’ column reports exact *k*-mer counts.

| ***k*-mer frequency** | **DSK 2.2.0** | **MSPKmer**  **Counter 0.1** | **Error%** | **aTurtle 0.3** | **Error%** |
| --- | --- | --- | --- | --- | --- |
| 1 | 155281400 | 146698167 | 5.85 | 155281818 | 0.0003 |
| 2 | 8864191 | 8707861 | 1.80 | 8864201 | 0.0001 |
| 3 | 4905173 | 4980239 | 1.51 | 4905177 | 0.0001 |
| 4 | 4626001 | 4790519 | 3.43 | 4625998 | 0.0001 |
| 5 | 5117297 | 5329251 | 3.98 | 5117299 | 0.0000 |
| 6 | 5763542 | 5981513 | 3.64 | 5763541 | 0.0000 |
| 7 | 6302198 | 6510090 | 3.19 | 6302198 | 0.0000 |
| 8 | 6658595 | 6838360 | 2.63 | 6658591 | 0.0001 |
| 9 | 6817573 | 6963587 | 2.10 | 6817577 | 0.0001 |
| 10 | 6799383 | 6918078 | 1.72 | 6799383 | 0.0000 |
| Average | | | 2.98 | Average | 0.0001 |
| Maximum | | | 5.85 | Maximum | 0.0003 |

**Table S6** Error rates (percent errors) of Gerbil 1.0, MSPKmerCounter 0.1 reported in the ‘Error%’ column for the DM dataset at *k* = 55. The ‘DSK 2.2.0’ column reports exact *k*-mer counts.

| ***k*-mer frequency** | **DSK 2.2.0** | **Gerbil 1.0** | **Error%** | **MSPKmerCounter 0.1** | **Error%** |
| --- | --- | --- | --- | --- | --- |
| 1 | 132512502 | 45255 | 292712.95 | 127152484 | 4.22 |
| 2 | 15209916 | 2025688 | 650.85 | 15386744 | 1.15 |
| 3 | 14350714 | 1926881 | 644.76 | 14551570 | 1.38 |
| 4 | 14070907 | 4197271 | 235.24 | 14200127 | 0.91 |
| 5 | 12981796 | 1679797 | 672.82 | 13035273 | 0.41 |
| 6 | 11361229 | 1458782 | 678.82 | 11342261 | 0.17 |
| 7 | 9542344 | 1232507 | 674.22 | 9474395 | 0.72 |
| 8 | 7807048 | 2659122 | 193.59 | 7709343 | 1.27 |
| 9 | 6272227 | 808026 | 676.24 | 6169544 | 1.66 |
| 10 | 4979037 | 634728 | 684.44 | 4880327 | 2.02 |
| Average | | | 29782.39 | Average | 1.39 |
| Maximum | | | 292712.95 | Maximum | 4.22 |

**Information regarding download of datasets**

1. We downloaded 11 ***F. vesca*** files from EBI:

http://www.ebi.ac.uk/ena/data/view/SRX030576 (SRR072006, SRR072007)

http://www.ebi.ac.uk/ena/data/view/SRX030575 (SRR072005, SRR072010, SRR072011, SRR072012)

http://www.ebi.ac.uk/ena/data/view/SRX030577 (SRR072008, SRR072009)

http://www.ebi.ac.uk/ena/data/view/SRX030578 (SRR072013, SRR072014, SRR072029)

2. We downloaded the Illumina Genome Analyzer II paired-end sequence of ***D. melanogaster*** from EBI with read library SRX040485 (2 files):

https://www.ebi.ac.uk/ena/data/view/SRX040485

3. Two ***M. balbisiana*** files were downloaded from DDBJ with Read library SRX339427:

ftp://ftp.ddbj.nig.ac.jp/ddbj_database/dra/fastq/SRA098/SRA098922/SRX339427/SRR956987.fastq.bz2

ftp://ftp.ddbj.nig.ac.jp/ddbj_database/dra/fastq/SRA098/SRA098922/SRX339427/SRR957627.fastq.bz

4. We downloaded a ***H. sapiens* 1** dataset from the dnanexus project, for the individual NA12878 (2 files):

https://dnanexus-rnd.s3.amazonaws.com/NA12878-xten/reads/NA12878D_HiSeqX_R1.fastq.gz

https://dnanexus-rnd.s3.amazonaws.com/NA12878-xten/reads/NA12878D_HiSeqX_R2.fastq.gz

5. We downloaded a ***H. sapiens* 2** dataset from EBI (48 files):

ftp.sra.ebi.ac.uk/vol1/fastq/ERR024/ERR024163/ERR024163_1.fastq.gz

ftp.sra.ebi.ac.uk/vol1/fastq/ERR024/ERR024163/ERR024163_2.fastq.gz

ftp.sra.ebi.ac.uk/vol1/fastq/ERR024/ERR024164/ERR024164_1.fastq.gz

ftp.sra.ebi.ac.uk/vol1/fastq/ERR024/ERR024164/ERR024164_2.fastq.gz

ftp.sra.ebi.ac.uk/vol1/fastq/ERR024/ERR024165/ERR024165_1.fastq.gz

ftp.sra.ebi.ac.uk/vol1/fastq/ERR024/ERR024165/ERR024165_2.fastq.gz

ftp.sra.ebi.ac.uk/vol1/fastq/ERR024/ERR024166/ERR024166_1.fastq.gz

ftp.sra.ebi.ac.uk/vol1/fastq/ERR024/ERR024166/ERR024166_2.fastq.gz

ftp.sra.ebi.ac.uk/vol1/fastq/ERR024/ERR024167/ERR024167_1.fastq.gz

ftp.sra.ebi.ac.uk/vol1/fastq/ERR024/ERR024167/ERR024167_2.fastq.gz

ftp.sra.ebi.ac.uk/vol1/fastq/ERR024/ERR024168/ERR024168_1.fastq.gz

ftp.sra.ebi.ac.uk/vol1/fastq/ERR024/ERR024168/ERR024168_2.fastq.gz

ftp.sra.ebi.ac.uk/vol1/fastq/ERR024/ERR024169/ERR024169_1.fastq.gz

ftp.sra.ebi.ac.uk/vol1/fastq/ERR024/ERR024169/ERR024169_2.fastq.gz

ftp.sra.ebi.ac.uk/vol1/fastq/ERR024/ERR024170/ERR024170_1.fastq.gz

ftp.sra.ebi.ac.uk/vol1/fastq/ERR024/ERR024170/ERR024170_2.fastq.gz

ftp.sra.ebi.ac.uk/vol1/fastq/ERR024/ERR024171/ERR024171_1.fastq.gz

ftp.sra.ebi.ac.uk/vol1/fastq/ERR024/ERR024171/ERR024171_2.fastq.gz

ftp.sra.ebi.ac.uk/vol1/fastq/ERR024/ERR024172/ERR024172_1.fastq.gz

ftp.sra.ebi.ac.uk/vol1/fastq/ERR024/ERR024172/ERR024172_2.fastq.gz

ftp.sra.ebi.ac.uk/vol1/fastq/ERR024/ERR024173/ERR024173_1.fastq.gz

ftp.sra.ebi.ac.uk/vol1/fastq/ERR024/ERR024173/ERR024173_2.fastq.gz

ftp.sra.ebi.ac.uk/vol1/fastq/ERR024/ERR024174/ERR024174_1.fastq.gz

ftp.sra.ebi.ac.uk/vol1/fastq/ERR024/ERR024174/ERR024174_2.fastq.gz

ftp.sra.ebi.ac.uk/vol1/fastq/ERR024/ERR024175/ERR024175_1.fastq.gz

ftp.sra.ebi.ac.uk/vol1/fastq/ERR024/ERR024175/ERR024175_2.fastq.gz

ftp.sra.ebi.ac.uk/vol1/fastq/ERR024/ERR024176/ERR024176_1.fastq.gz

ftp.sra.ebi.ac.uk/vol1/fastq/ERR024/ERR024176/ERR024176_2.fastq.gz

ftp.sra.ebi.ac.uk/vol1/fastq/ERR024/ERR024177/ERR024177_1.fastq.gz

ftp.sra.ebi.ac.uk/vol1/fastq/ERR024/ERR024177/ERR024177_2.fastq.gz

ftp.sra.ebi.ac.uk/vol1/fastq/ERR024/ERR024178/ERR024178_1.fastq.gz

ftp.sra.ebi.ac.uk/vol1/fastq/ERR024/ERR024178/ERR024178_2.fastq.gz

ftp.sra.ebi.ac.uk/vol1/fastq/ERR024/ERR024179/ERR024179_1.fastq.gz

ftp.sra.ebi.ac.uk/vol1/fastq/ERR024/ERR024179/ERR024179_2.fastq.gz

ftp.sra.ebi.ac.uk/vol1/fastq/ERR024/ERR024180/ERR024180_1.fastq.gz

ftp.sra.ebi.ac.uk/vol1/fastq/ERR024/ERR024180/ERR024180_2.fastq.gz

ftp.sra.ebi.ac.uk/vol1/fastq/ERR024/ERR024181/ERR024181_1.fastq.gz

ftp.sra.ebi.ac.uk/vol1/fastq/ERR024/ERR024181/ERR024181_2.fastq.gz

ftp.sra.ebi.ac.uk/vol1/fastq/ERR024/ERR024182/ERR024182_1.fastq.gz

ftp.sra.ebi.ac.uk/vol1/fastq/ERR024/ERR024182/ERR024182_2.fastq.gz

ftp.sra.ebi.ac.uk/vol1/fastq/ERR024/ERR024183/ERR024183_1.fastq.gz

ftp.sra.ebi.ac.uk/vol1/fastq/ERR024/ERR024183/ERR024183_2.fastq.gz

ftp.sra.ebi.ac.uk/vol1/fastq/ERR024/ERR024184/ERR024184_1.fastq.gz

ftp.sra.ebi.ac.uk/vol1/fastq/ERR024/ERR024184/ERR024184_2.fastq.gz

ftp.sra.ebi.ac.uk/vol1/fastq/ERR024/ERR024185/ERR024185_1.fastq.gz

ftp.sra.ebi.ac.uk/vol1/fastq/ERR024/ERR024185/ERR024185_2.fastq.gz

ftp.sra.ebi.ac.uk/vol1/fastq/ERR024/ERR024186/ERR024186_1.fastq.gz

ftp.sra.ebi.ac.uk/vol1/fastq/ERR024/ERR024186/ERR024186_2.fastq.gz

6. Eighteen SRA files were downloaded from the links below for the ***N. crassa*** dataset:

ftp://ftp.ncbi.nlm.nih.gov/sra/sra-instant/reads/ByExp/sra/SRX/SRX533/SRX533606/SRR1284075/

<ftp://ftp.ncbi.nlm.nih.gov/sra/sra-instant/reads/ByExp/sra/SRX/SRX533/SRX533606/SRR1284076/>

<ftp://ftp.ncbi.nlm.nih.gov/sra/sra-instant/reads/ByExp/sra/SRX/SRX533/SRX533606/SRR1284078/>

<ftp://ftp.ncbi.nlm.nih.gov/sra/sra-instant/reads/ByExp/sra/SRX/SRX533/SRX533606/SRR1284079/>

<ftp://ftp.ncbi.nlm.nih.gov/sra/sra-instant/reads/ByExp/sra/SRX/SRX533/SRX533606/SRR1284081/>

<ftp://ftp.ncbi.nlm.nih.gov/sra/sra-instant/reads/ByExp/sra/SRX/SRX533/SRX533606/SRR1284681/>

<ftp://ftp.ncbi.nlm.nih.gov/sra/sra-instant/reads/ByExp/sra/SRX/SRX533/SRX533606/SRR1284682/>

<ftp://ftp.ncbi.nlm.nih.gov/sra/sra-instant/reads/ByExp/sra/SRX/SRX533/SRX533606/SRR1284683/>

<ftp://ftp.ncbi.nlm.nih.gov/sra/sra-instant/reads/ByExp/sra/SRX/SRX533/SRX533606/SRR1284684/>

<ftp://ftp.ncbi.nlm.nih.gov/sra/sra-instant/reads/ByExp/sra/SRX/SRX533/SRX533606/SRR1284685/>

<ftp://ftp.ncbi.nlm.nih.gov/sra/sra-instant/reads/ByExp/sra/SRX/SRX533/SRX533606/SRR1284686/>

<ftp://ftp.ncbi.nlm.nih.gov/sra/sra-instant/reads/ByExp/sra/SRX/SRX533/SRX533606/SRR1284690/>

<ftp://ftp.ncbi.nlm.nih.gov/sra/sra-instant/reads/ByExp/sra/SRX/SRX533/SRX533606/SRR1284691/>

<ftp://ftp.ncbi.nlm.nih.gov/sra/sra-instant/reads/ByExp/sra/SRX/SRX533/SRX533606/SRR1284692/>

<ftp://ftp.ncbi.nlm.nih.gov/sra/sra-instant/reads/ByExp/sra/SRX/SRX533/SRX533606/SRR1284694/>

<ftp://ftp.ncbi.nlm.nih.gov/sra/sra-instant/reads/ByExp/sra/SRX/SRX533/SRX533606/SRR1284943/>

ftp://ftp.ncbi.nlm.nih.gov/sra/sra-instant/reads/ByExp/sra/SRX/SRX533/SRX533606/SRR1284944/

<ftp://ftp.ncbi.nlm.nih.gov/sra/sra-instant/reads/ByExp/sra/SRX/SRX533/SRX533606/SRR1284945/>

7. Forty-six SRA files were downloaded from the links below for the ***A. thaliana*** dataset:

<ftp://ftp.ncbi.nlm.nih.gov/sra/sra-instant/reads/ByExp/sra/SRX/SRX533/SRX533607/SRR1284093/>

<ftp://ftp.ncbi.nlm.nih.gov/sra/sra-instant/reads/ByExp/sra/SRX/SRX533/SRX533607/SRR1284094/>

<ftp://ftp.ncbi.nlm.nih.gov/sra/sra-instant/reads/ByExp/sra/SRX/SRX533/SRX533607/SRR1284095/>

<ftp://ftp.ncbi.nlm.nih.gov/sra/sra-instant/reads/ByExp/sra/SRX/SRX533/SRX533607/SRR1284096/>

<ftp://ftp.ncbi.nlm.nih.gov/sra/sra-instant/reads/ByExp/sra/SRX/SRX533/SRX533607/SRR1284703/>

<ftp://ftp.ncbi.nlm.nih.gov/sra/sra-instant/reads/ByExp/sra/SRX/SRX533/SRX533607/SRR1284704/>

[ftp://ftp.ncbi.nlm.nih.gov/sra/sra-instant/reads/ByExp/sra/SRX/SRX533/SRX533607/SRR1284705/](ftp://ftp.ncbi.nlm.nih.gov/sra/sra-instant/reads/ByExp/sra/SRX/SRX533/SRX533607/SRR1284704/)

[ftp://ftp.ncbi.nlm.nih.gov/sra/sra-instant/reads/ByExp/sra/SRX/SRX533/SRX533607/SRR1284706/](ftp://ftp.ncbi.nlm.nih.gov/sra/sra-instant/reads/ByExp/sra/SRX/SRX533/SRX533607/SRR1284704/)

[ftp://ftp.ncbi.nlm.nih.gov/sra/sra-instant/reads/ByExp/sra/SRX/SRX533/SRX533607/SRR1284707/](ftp://ftp.ncbi.nlm.nih.gov/sra/sra-instant/reads/ByExp/sra/SRX/SRX533/SRX533607/SRR1284704/)

[ftp://ftp.ncbi.nlm.nih.gov/sra/sra-instant/reads/ByExp/sra/SRX/SRX533/SRX533607/SRR1284708/](ftp://ftp.ncbi.nlm.nih.gov/sra/sra-instant/reads/ByExp/sra/SRX/SRX533/SRX533607/SRR1284704/)

[ftp://ftp.ncbi.nlm.nih.gov/sra/sra-instant/reads/ByExp/sra/SRX/SRX533/SRX533607/SRR1284709/](ftp://ftp.ncbi.nlm.nih.gov/sra/sra-instant/reads/ByExp/sra/SRX/SRX533/SRX533607/SRR1284704/)

<ftp://ftp.ncbi.nlm.nih.gov/sra/sra-instant/reads/ByExp/sra/SRX/SRX533/SRX533607/SRR1284710/>

<ftp://ftp.ncbi.nlm.nih.gov/sra/sra-instant/reads/ByExp/sra/SRX/SRX533/SRX533607/SRR1284902/>

<ftp://ftp.ncbi.nlm.nih.gov/sra/sra-instant/reads/ByExp/sra/SRX/SRX533/SRX533607/SRR1284903/>

[ftp://ftp.ncbi.nlm.nih.gov/sra/sra-instant/reads/ByExp/sra/SRX/SRX533/SRX533607/SRR1284904/](ftp://ftp.ncbi.nlm.nih.gov/sra/sra-instant/reads/ByExp/sra/SRX/SRX533/SRX533607/SRR1284902/)

[ftp://ftp.ncbi.nlm.nih.gov/sra/sra-instant/reads/ByExp/sra/SRX/SRX533/SRX533607/SRR1284905/](ftp://ftp.ncbi.nlm.nih.gov/sra/sra-instant/reads/ByExp/sra/SRX/SRX533/SRX533607/SRR1284902/)

[ftp://ftp.ncbi.nlm.nih.gov/sra/sra-instant/reads/ByExp/sra/SRX/SRX533/SRX533607/SRR1284906/](ftp://ftp.ncbi.nlm.nih.gov/sra/sra-instant/reads/ByExp/sra/SRX/SRX533/SRX533607/SRR1284902/)

[ftp://ftp.ncbi.nlm.nih.gov/sra/sra-instant/reads/ByExp/sra/SRX/SRX533/SRX533607/SRR1284907/](ftp://ftp.ncbi.nlm.nih.gov/sra/sra-instant/reads/ByExp/sra/SRX/SRX533/SRX533607/SRR1284902/)

[ftp://ftp.ncbi.nlm.nih.gov/sra/sra-instant/reads/ByExp/sra/SRX/SRX533/SRX533607/SRR1284908/](ftp://ftp.ncbi.nlm.nih.gov/sra/sra-instant/reads/ByExp/sra/SRX/SRX533/SRX533607/SRR1284902/)

[ftp://ftp.ncbi.nlm.nih.gov/sra/sra-instant/reads/ByExp/sra/SRX/SRX533/SRX533607/SRR1284909/](ftp://ftp.ncbi.nlm.nih.gov/sra/sra-instant/reads/ByExp/sra/SRX/SRX533/SRX533607/SRR1284902/)

[ftp://ftp.ncbi.nlm.nih.gov/sra/sra-instant/reads/ByExp/sra/SRX/SRX533/SRX533607/SRR1284910/](ftp://ftp.ncbi.nlm.nih.gov/sra/sra-instant/reads/ByExp/sra/SRX/SRX533/SRX533607/SRR1284902/)

[ftp://ftp.ncbi.nlm.nih.gov/sra/sra-instant/reads/ByExp/sra/SRX/SRX533/SRX533607/SRR1284911/](ftp://ftp.ncbi.nlm.nih.gov/sra/sra-instant/reads/ByExp/sra/SRX/SRX533/SRX533607/SRR1284902/)

[ftp://ftp.ncbi.nlm.nih.gov/sra/sra-instant/reads/ByExp/sra/SRX/SRX533/SRX533607/SRR1284912/](ftp://ftp.ncbi.nlm.nih.gov/sra/sra-instant/reads/ByExp/sra/SRX/SRX533/SRX533607/SRR1284902/)

[ftp://ftp.ncbi.nlm.nih.gov/sra/sra-instant/reads/ByExp/sra/SRX/SRX533/SRX533607/SRR1284913/](ftp://ftp.ncbi.nlm.nih.gov/sra/sra-instant/reads/ByExp/sra/SRX/SRX533/SRX533607/SRR1284902/)

<ftp://ftp.ncbi.nlm.nih.gov/sra/sra-instant/reads/ByExp/sra/SRX/SRX533/SRX533607/SRR1284914/>

<ftp://ftp.ncbi.nlm.nih.gov/sra/sra-instant/reads/ByExp/sra/SRX/SRX533/SRX533607/SRR1284915/>

<ftp://ftp.ncbi.nlm.nih.gov/sra/sra-instant/reads/ByExp/sra/SRX/SRX533/SRX533607/SRR1284916/>

<ftp://ftp.ncbi.nlm.nih.gov/sra/sra-instant/reads/ByExp/sra/SRX/SRX533/SRX533607/SRR1284917/>

<ftp://ftp.ncbi.nlm.nih.gov/sra/sra-instant/reads/ByExp/sra/SRX/SRX533/SRX533607/SRR1284918/>

<ftp://ftp.ncbi.nlm.nih.gov/sra/sra-instant/reads/ByExp/sra/SRX/SRX533/SRX533607/SRR1284919/>

<ftp://ftp.ncbi.nlm.nih.gov/sra/sra-instant/reads/ByExp/sra/SRX/SRX533/SRX533607/SRR1284920/>

[ftp://ftp.ncbi.nlm.nih.gov/sra/sra-instant/reads/ByExp/sra/SRX/SRX533/SRX533607/SRR128492](ftp://ftp.ncbi.nlm.nih.gov/sra/sra-instant/reads/ByExp/sra/SRX/SRX533/SRX533607/SRR1284921/)1/

[ftp://ftp.ncbi.nlm.nih.gov/sra/sra-instant/reads/ByExp/sra/SRX/SRX533/SRX533607/SRR128492](ftp://ftp.ncbi.nlm.nih.gov/sra/sra-instant/reads/ByExp/sra/SRX/SRX533/SRX533607/SRR1284922/)2/

[ftp://ftp.ncbi.nlm.nih.gov/sra/sra-instant/reads/ByExp/sra/SRX/SRX533/SRX533607/SRR128492](ftp://ftp.ncbi.nlm.nih.gov/sra/sra-instant/reads/ByExp/sra/SRX/SRX533/SRX533607/SRR1284923/)3/

[ftp://ftp.ncbi.nlm.nih.gov/sra/sra-instant/reads/ByExp/sra/SRX/SRX533/SRX533607/SRR128492](ftp://ftp.ncbi.nlm.nih.gov/sra/sra-instant/reads/ByExp/sra/SRX/SRX533/SRX533607/SRR1284924/)4/

[ftp://ftp.ncbi.nlm.nih.gov/sra/sra-instant/reads/ByExp/sra/SRX/SRX533/SRX533607/SRR128492](ftp://ftp.ncbi.nlm.nih.gov/sra/sra-instant/reads/ByExp/sra/SRX/SRX533/SRX533607/SRR1284925/)5/

<ftp://ftp.ncbi.nlm.nih.gov/sra/sra-instant/reads/ByExp/sra/SRX/SRX533/SRX533607/SRR1313323/>

<ftp://ftp.ncbi.nlm.nih.gov/sra/sra-instant/reads/ByExp/sra/SRX/SRX533/SRX533607/SRR1313324/>

[ftp://ftp.ncbi.nlm.nih.gov/sra/sra-instant/reads/ByExp/sra/SRX/SRX533/SRX533607/SRR1313325/](ftp://ftp.ncbi.nlm.nih.gov/sra/sra-instant/reads/ByExp/sra/SRX/SRX533/SRX533607/SRR1313324/)

[ftp://ftp.ncbi.nlm.nih.gov/sra/sra-instant/reads/ByExp/sra/SRX/SRX533/SRX533607/SRR1313326/](ftp://ftp.ncbi.nlm.nih.gov/sra/sra-instant/reads/ByExp/sra/SRX/SRX533/SRX533607/SRR1313324/)

[ftp://ftp.ncbi.nlm.nih.gov/sra/sra-instant/reads/ByExp/sra/SRX/SRX533/SRX533607/SRR1313327/](ftp://ftp.ncbi.nlm.nih.gov/sra/sra-instant/reads/ByExp/sra/SRX/SRX533/SRX533607/SRR1313324/)

[ftp://ftp.ncbi.nlm.nih.gov/sra/sra-instant/reads/ByExp/sra/SRX/SRX533/SRX533607/SRR1313328/](ftp://ftp.ncbi.nlm.nih.gov/sra/sra-instant/reads/ByExp/sra/SRX/SRX533/SRX533607/SRR1313324/)

[ftp://ftp.ncbi.nlm.nih.gov/sra/sra-instant/reads/ByExp/sra/SRX/SRX533/SRX533607/SRR1313329/](ftp://ftp.ncbi.nlm.nih.gov/sra/sra-instant/reads/ByExp/sra/SRX/SRX533/SRX533607/SRR1313324/)

<ftp://ftp.ncbi.nlm.nih.gov/sra/sra-instant/reads/ByExp/sra/SRX/SRX533/SRX533607/SRR1313330/>

[ftp://ftp.ncbi.nlm.nih.gov/sra/sra-instant/reads/ByExp/sra/SRX/SRX533/SRX533607/SRR1313331/](ftp://ftp.ncbi.nlm.nih.gov/sra/sra-instant/reads/ByExp/sra/SRX/SRX533/SRX533607/SRR1313324/)

<ftp://ftp.ncbi.nlm.nih.gov/sra/sra-instant/reads/ByExp/sra/SRX/SRX533/SRX533607/SRR1313332/>

**Command lines for the ten tools for the FV, DM, MB, HS1, HS2, NC and AT datasets**

1. **Gerbil 1.0**

./gerbil -k 28 -t 12 -l 2 -e 12GB fv.fastq temp/ fv_28

./gerbil -k 55 -t 12 -l 2 -e 12GB fv.fastq temp/ fv_55

./gerbil -k 28 -t 12 -l 2 -e 12GB dm.fastq temp/ dm_28

./gerbil -k 55 -t 12 -l 2 -e 12GB dm.fastq temp/ dm_55

./gerbil -k 28 -t 12 -l 2 -e 12GB mb.fastq temp/ mb_28

./gerbil -k 55 -t 12 -l 2 -e 12GB mb.fastq temp/ mb_55

./gerbil -k 28 -t 12 -l 2 -e 12GB hs1.fastq temp/ hs1_28

./gerbil -k 55 -t 12 -l 2 -e 12GB hs1.fastq temp/ hs1_55

./gerbil -k 28 -t 12 -l 2 -e 12GB hs2.fastq temp/ hs2_28

./gerbil -k 55 -t 12 -l 2 -e 12GB hs2.fastq temp/ hs2_55

Dump Subroutine

./toFasta fv_28 28 dump_28

./toFasta fv_55 55 dump_55

./toFasta dm_28 28 dump_28

./toFasta dm_55 55 dump_55

NC dataset

./gerbil -k 28 -t 12 -l 2 -e 12GB nc.fasta temp/ nc_28

./gerbil -k 40 -t 12 -l 2 -e 12GB nc.fasta temp/ nc_40

./gerbil -k 55 -t 12 -l 2 -e 12GB nc.fasta temp/ nc_55

./gerbil -k 65 -t 12 -l 2 -e 12GB nc.fasta temp/ nc_65

./gerbil -k 100 -t 12 -l 2 -e 12GB nc.fasta temp/ nc_100

./gerbil -k 125 -t 12 -l 2 -e 12GB nc.fasta temp/ nc_125

./gerbil -k 150 -t 12 -l 2 -e 12GB nc.fasta temp/ nc_150

./gerbil -k 175 -t 12 -l 2 -e 12GB nc.fasta temp/ nc_175

./gerbil -k 200 -t 12 -l 2 -e 12GB nc.fasta temp/ nc_200

AT dataset

./gerbil -k 28 -t 12 -l 2 -e 12GB at.fastq temp/ at_28

./gerbil -k 40 -t 12 -l 2 -e 12GB at.fastq temp/ at_40

/gerbil -k 55 -t 12 -l 2 -e 12GB at.fastq temp/ at_55

./gerbil -k 65 -t 12 -l 2 -e 12GB at.fastq temp/ at_65

./gerbil -k 100 -t 12 -l 2 -e 12GB at.fastq temp/ at_100

./gerbil -k 125 -t 12 -l 2 -e 12GB at.fastq temp/ at_125

./gerbil -k 150 -t 12 -l 2 -e 12GB at.fastq temp/ at_150

./gerbil -k 175 -t 12 -l 2 -e 12GB at.fastq temp/ at_175

./gerbil -k 200 -t 12 -l 2 -e 12GB at.fastq temp/ at_200

Gzip

./gerbil -k 28 -t 12 -l 2 -e 12GB -i fv.txt temp/ fv_28

./gerbil -k 55 -t 12 -l 2 -e 12GB -i fv.txt temp/ fv_55

./gerbil -k 28 -t 12 -l 2 -e 12GB -i dm.txt temp/ dm_28

./gerbil -k 55 -t 12 -l 2 -e 12GB -i dm.txt temp/ dm_55

./gerbil -k 28 -t 12 -l 2 -e 12GB -i mb.txt temp/ mb_28

./gerbil -k 55 -t 12 -l 2 -e 12GB -i mb.txt temp/ mb_55

./gerbil -k 28 -t 12 -l 2 -e 12GB -i hs1.txt temp/ hs1_28

./gerbil -k 55 -t 12 -l 2 -e 12GB -i hs1.txt temp/ hs1_55

./gerbil -k 28 -t 12 -l 2 -e 12GB -i hs2.txt temp/ hs2_28

./gerbil -k 55 -t 12 -l 2 -e 12GB -i hs2.txt temp/ hs2_55

2. **KAnalyze 2.0.0**

java -jar ./kanalyze.jar count -d 6 -f fastq -k 28 -l 6 -rcanonical -c kmercount:2 -v -o fv_28 fv.fastq

java -jar ./kanalyze.jar count -d 6 -f fastq -k 55 -l 6 -rcanonical -c kmercount:2 -v -o fv_55 fv.fastq

java -jar ./kanalyze.jar count -d 6 -f fastq -k 28 -l 6 -rcanonical -c kmercount:2 -v -o dm_28 dm.fastq

java -jar ./kanalyze.jar count -d 6 -f fastq -k 55 -l 6 -rcanonical -c kmercount:2 -v -o dm_55 dm.fastq

java -jar ./kanalyze.jar count -d 6 -f fastq -k 28 -l 6 -rcanonical -c kmercount:2 -v -o mb_28 mb.fastq

java -jar ./kanalyze.jar count -d 6 -f fastq -k 55 -l 6 -rcanonical -c kmercount:2 -v -o mb_55 mb.fastq

java -jar ./kanalyze.jar count -d 6 -f fastq -k 28 -l 6 -rcanonical -c kmercount:2 -v -o hs1_28 hs1.fastq

java -jar ./kanalyze.jar count -d 6 -f fastq -k 55 -l 6 -rcanonical -c kmercount:2 -v -o hs1_55 hs1.fastq

java -jar ./kanalyze.jar count -d 6 -f fastq -k 28 -l 6 -rcanonical -c kmercount:2 -v -o hs2_28 hs2.fastq

java -jar ./kanalyze.jar count -d 6 -f fastq -k 55 -l 6 -rcanonical -c kmercount:2 -v -o hs2_55 hs2.fastq

Gzip

java -jar ./kanalyze.jar count -d 6 -f fastq -k 28 -l 6 -rcanonical -c kmercount:2 -v -o fv_28 -f fastqgz SRR072005.fastq.gz SRR072006.fastq.gz SRR072007.fastq.gz SRR072008.fastq.gz SRR072009.fastq.gz SRR072010.fastq.gz SRR072011.fastq.gz SRR072012.fastq.gz SRR072013.fastq.gz SRR072014.fastq.gz SRR072029.fastq.gz

java -jar ./kanalyze.jar count -d 6 -f fastq -k 55 -l 6 -rcanonical -c kmercount:2 -v -o fv_55 -f fastqgz SRR072005.fastq.gz SRR072006.fastq.gz SRR072007.fastq.gz SRR072008.fastq.gz SRR072009.fastq.gz SRR072010.fastq.gz SRR072011.fastq.gz SRR072012.fastq.gz SRR072013.fastq.gz SRR072014.fastq.gz SRR072029.fastq.gz

java -jar ./kanalyze.jar count -d 6 -f fastq -k 28 -l 6 -rcanonical -c kmercount:2 -v -o dm_28 -f fastqgz SRR097732_1.fastq.gz SRR097732_2.fastq.gz

java -jar ./kanalyze.jar count -d 6 -f fastq -k 55 -l 6 -rcanonical -c kmercount:2 -v -o dm_55 -f fastqgz SRR097732_1.fastq.gz SRR097732_2.fastq.gz

java -jar ./kanalyze.jar count -d 6 -f fastq -k 28 -l 6 -rcanonical -c kmercount:2 -v -o hs1_28 -f fastqgz NA12878D_HiSeqX_R1.fastq.gz NA12878D_HiSeqX_R2.fastq.gz

java -jar ./kanalyze.jar count -d 6 -f fastq -k 55 -l 6 -rcanonical -c kmercount:2 -v -o hs1_55 -f fastqgz NA12878D_HiSeqX_R1.fastq.gz NA12878D_HiSeqX_R2.fastq.gz

java -jar ./kanalyze.jar count -d 6 -f fastq -k 28 -l 6 -rcanonical -c kmercount:2 -v -o hs2_28 -f fastqgz ERR024163_1.fastq.gz ERR024163_2.fastq.gz ERR024164_1.fastq.gz ERR024164_2.fastq.gz ERR024165_1.fastq.gz ERR024165_2.fastq.gz ERR024166_1.fastq.gz ERR024166_2.fastq.gz ERR024167_1.fastq.gz ERR024167_2.fastq.gz ERR024168_1.fastq.gz ERR024168_2.fastq.gz ERR024169_1.fastq.gz ERR024169_2.fastq.gz ERR024170_1.fastq.gz ERR024170_2.fastq.gz ERR024171_1.fastq.gz ERR024171_2.fastq.gz ERR024172_1.fastq.gz ERR024172_2.fastq.gz ERR024173_1.fastq.gz ERR024173_2.fastq.gz ERR024174_1.fastq.gz ERR024174_2.fastq.gz ERR024175_1.fastq.gz ERR024175_2.fastq.gz ERR024176_1.fastq.gz ERR024176_2.fastq.gz ERR024177_1.fastq.gz ERR024177_2.fastq.gz ERR024178_1.fastq.gz ERR024178_2.fastq.gz ERR024179_1.fastq.gz ERR024179_2.fastq.gz ERR024180_1.fastq.gz ERR024180_2.fastq.gz ERR024181_1.fastq.gz ERR024181_2.fastq.gz ERR024182_1.fastq.gz ERR024182_2.fastq.gz ERR024183_1.fastq.gz ERR024183_2.fastq.gz ERR024184_1.fastq.gz ERR024184_2.fastq.gz ERR024185_1.fastq.gz ERR024185_2.fastq.gz ERR024186_1.fastq.gz ERR024186_2.fastq.gz

java -jar ./kanalyze.jar count -d 6 -f fastq -k 55 -l 6 -rcanonical -c kmercount:2 -v -o hs2_55 -f fastqgz ERR024163_1.fastq.gz ERR024163_2.fastq.gz ERR024164_1.fastq.gz ERR024164_2.fastq.gz ERR024165_1.fastq.gz ERR024165_2.fastq.gz ERR024166_1.fastq.gz ERR024166_2.fastq.gz ERR024167_1.fastq.gz ERR024167_2.fastq.gz ERR024168_1.fastq.gz ERR024168_2.fastq.gz ERR024169_1.fastq.gz ERR024169_2.fastq.gz ERR024170_1.fastq.gz ERR024170_2.fastq.gz ERR024171_1.fastq.gz ERR024171_2.fastq.gz ERR024172_1.fastq.gz ERR024172_2.fastq.gz ERR024173_1.fastq.gz ERR024173_2.fastq.gz ERR024174_1.fastq.gz ERR024174_2.fastq.gz ERR024175_1.fastq.gz ERR024175_2.fastq.gz ERR024176_1.fastq.gz ERR024176_2.fastq.gz ERR024177_1.fastq.gz ERR024177_2.fastq.gz ERR024178_1.fastq.gz ERR024178_2.fastq.gz ERR024179_1.fastq.gz ERR024179_2.fastq.gz ERR024180_1.fastq.gz ERR024180_2.fastq.gz ERR024181_1.fastq.gz ERR024181_2.fastq.gz ERR024182_1.fastq.gz ERR024182_2.fastq.gz ERR024183_1.fastq.gz ERR024183_2.fastq.gz ERR024184_1.fastq.gz ERR024184_2.fastq.gz ERR024185_1.fastq.gz ERR024185_2.fastq.gz ERR024186_1.fastq.gz ERR024186_2.fastq.gz

3. **DSK 2.1.0**

./dsk -file fv.fastq -kmer-size 28 -abundance-min 2 -verbose 2 -nb-cores 12 -max-memory 12000 -out fv_28

./dsk -file fv.fastq -kmer-size 55 -abundance-min 2 -verbose 2 -nb-cores 12 -max-memory 12000 -out fv_55

./dsk -file dm.fastq -kmer-size 28 -abundance-min 2 -verbose 2 -nb-cores 12 -max-memory 12000 -out dm_28

./dsk -file dm.fastq -kmer-size 55 -abundance-min 2 -verbose 2 -nb-cores 12 -max-memory 12000 -out dm_55

./dsk -file mb.fastq -kmer-size 28 -abundance-min 2 -verbose 2 -nb-cores 12 -max-memory 12000 -out mb_28

./dsk -file mb.fastq -kmer-size 55 -abundance-min 2 -verbose 2 -nb-cores 12 -max-memory 12000 -out mb_55

./dsk -file hs1.fastq -kmer-size 28 -abundance-min 2 -verbose 2 -nb-cores 12 -max-memory 12000 -out hs1_28

./dsk -file hs1.fastq -kmer-size 55 -abundance-min 2 -verbose 2 -nb-cores 12 -max-memory 12000 -out hs1_55

./dsk -file hs2.fastq -kmer-size 28 -abundance-min 2 -verbose 2 -nb-cores 12 -max-memory 12000 -out hs2_28

./dsk -file hs2.fastq -kmer-size 55 -abundance-min 2 -verbose 2 -nb-cores 12 -max-memory 12000 -out hs2_55

Histogram Subroutine

h5dump -y -d histogram/histogram fv_28.h5 >> histo_28

h5dump -y -d histogram/histogram fv_55.h5 >> histo_55

h5dump -y -d histogram/histogram dm_28.h5 >> histo_28

h5dump -y -d histogram/histogram dm_55.h5 >> histo_55

NC dataset

./dsk -file nc.fasta -kmer-size 28 -abundance-min 2 -verbose 2 -nb-cores 12 -max-memory 12000 -out nc_28

./dsk -file nc.fasta -kmer-size 40 -abundance-min 2 -verbose 2 -nb-cores 12 -max-memory 12000 -out nc_40

./dsk -file nc.fasta -kmer-size 55 -abundance-min 2 -verbose 2 -nb-cores 12 -max-memory 12000 -out nc_55

./dsk -file nc.fasta -kmer-size 65 -abundance-min 2 -verbose 2 -nb-cores 12 -max-memory 12000 -out nc_65

./dsk -file nc.fasta -kmer-size 100 -abundance-min 2 -verbose 2 -nb-cores 12 -max-memory 12000 -out nc_100

./dsk -file nc.fasta -kmer-size 125 -abundance-min 2 -verbose 2 -nb-cores 12 -max-memory 12000 -out nc_125

./dsk -file nc.fasta -kmer-size 150 -abundance-min 2 -verbose 2 -nb-cores 12 -max-memory 12000 -out nc_150

./dsk -file nc.fasta -kmer-size 175 -abundance-min 2 -verbose 2 -nb-cores 12 -max-memory 12000 -out nc_175

./dsk -file nc.fasta -kmer-size 200 -abundance-min 2 -verbose 2 -nb-cores 12 -max-memory 12000 -out nc_200

AT dataset

./dsk -file at.fastq -kmer-size 28 -abundance-min 2 -verbose 2 -nb-cores 12 -max-memory 12000 -out at_28

./dsk -file at.fastq -kmer-size 40 -abundance-min 2 -verbose 2 -nb-cores 12 -max-memory 12000 -out at_40

./dsk -file at.fastq -kmer-size 55 -abundance-min 2 -verbose 2 -nb-cores 12 -max-memory 12000 -out at_55

./dsk -file at.fastq -kmer-size 65 -abundance-min 2 -verbose 2 -nb-cores 12 -max-memory 12000 -out at_65

./dsk -file at.fastq -kmer-size 100 -abundance-min 2 -verbose 2 -nb-cores 12 -max-memory 12000 -out at_100

./dsk -file at.fastq -kmer-size 125 -abundance-min 2 -verbose 2 -nb-cores 12 -max-memory 12000 -out at_125

./dsk -file at.fastq -kmer-size 150 -abundance-min 2 -verbose 2 -nb-cores 12 -max-memory 12000 -out at_150

./dsk -file at.fastq -kmer-size 175 -abundance-min 2 -verbose 2 -nb-cores 12 -max-memory 12000 -out at_175

./dsk -file at.fastq -kmer-size 200 -abundance-min 2 -verbose 2 -nb-cores 12 -max-memory 12000 -out at_200

Gzip

./dsk -file fv.txt -kmer-size 28 -max-memory 12000 -verbose 2 -nb-cores 12 -out fv_28 -abundance-min 2

./dsk -file fv.txt -kmer-size 55 -max-memory 12000 -verbose 2 -nb-cores 12 -out fv_55 -abundance-min 2

./dsk -file dm.txt -kmer-size 28 -max-memory 12000 -verbose 2 -nb-cores 12 -out dm_28 -abundance-min 2

./dsk -file dm.txt -kmer-size 55 -max-memory 12000 -verbose 2 -nb-cores 12 -out dm_55 -abundance-min 2

./dsk -file hs1.txt -kmer-size 28 -max-memory 12000 -verbose 2 -nb-cores 12 -out hs1_28 -abundance-min 2

./dsk -file hs1.txt -kmer-size 55 -max-memory 12000 -verbose 2 -nb-cores 12 -out hs1_55 -abundance-min 2

./dsk -file hs2.txt -kmer-size 28 -max-memory 12000 -verbose 2 -nb-cores 12 -out hs2_28 -abundance-min 2

./dsk -file hs2.txt -kmer-size 55 -max-memory 12000 -verbose 2 -nb-cores 12 -out hs2_55 -abundance-min 2

4. **Jellyfish 2.2.6**

./jellyfish count -m 28 -C -s 300M -t 12 -L 2 -o fv_28 fv.fastq

./jellyfish count -m 55 -C -s 400M -t 12 -L 2 -o fv_55 fv.fastq

./jellyfish count -m 28 -C -s 250M -t 12 -L 2 -o dm_28 dm.fastq

./jellyfish count -m 55 -C -s 250M -t 12 -L 2 -o dm_55 dm.fastq

./jellyfish count -m 28 -C -s 1G -t 12 -L 2 -o mb_28 mb.fastq

./jellyfish count -m 55 -C -s 1G -t 12 -L 2 -o mb_55 mb.fastq

./jellyfish count -m 28 -C -s 3G -t 12 -L 2 -o hs1_28 hs1.fastq

./jellyfish count -m 55 -C -s 3G -t 12 -L 2 -o hs1_55 hs1.fastq

./jellyfish count -m 28 -C -s 3G -t 12 -L 2 -o hs2_28 hs2.fastq

./jellyfish count -m 55 -C -s 3G -t 12 -L 2 -o hs2_55 hs2.fastq

Histogram Subroutine

./jellyfish histo -o histo_28 fv_28

./jellyfish histo -o histo_55 fv_55

./jellyfish histo -o histo_28 dm_28

./jellyfish histo -o histo_55 dm_55

5. **KMC3**

./kmc -k28 -m12 -t12 -ci2 fv.fastq NA.res \data

./kmc -k55 -m12 -t12 -ci2 fv.fastq NA.res \data

./kmc -k28 -m12 -t12 -ci2 dm.fastq NA.res \data

./kmc -k55 -m12 -t12 -ci2 dm.fastq NA.res \data

./kmc -k28 -m12 -t12 -ci2 mb.fastq NA.res \data

./kmc -k55 -m12 -t12 -ci2 mb.fastq NA.res \data

./kmc -k28 -m12 -t12 -ci2 hs1.fastq NA.res \data

./kmc -k55 -m12 -t12 -ci2 hs1.fastq NA.res \data

./kmc -k28 -m12 -t12 -ci2 hs2.fastq NA.res \data

./kmc -k55 -m12 -t12 -ci2 hs2.fastq NA.res \data

Dump subroutine

./kmc_dump NA.res fv_28

./kmc_dump NA.res fv_55

./kmc_dump NA.res dm_28

./kmc_dump NA.res dm_55

NC dataset

./kmc -k28 -m12 -t12 -ci2 -fm nc.fasta NA.res \data

./kmc -k40 -m12 -t12 -ci2 -fm nc.fasta NA.res \data

./kmc -k55 -m12 -t12 -ci2 -fm nc.fasta NA.res \data

./kmc -k65 -m12 -t12 -ci2 -fm nc.fasta NA.res \data

./kmc -k100 -m12 -t12 -ci2 -fm nc.fasta NA.res \data

./kmc -k125 -m12 -t12 -ci2 -fm nc.fasta NA.res \data

./kmc -k150 -m12 -t12 -ci2 -fm nc.fasta NA.res \data

./kmc -k175 -m12 -t12 -ci2 -fm nc.fasta NA.res \data

./kmc -k200 -m12 -t12 -ci2 -fm nc.fasta NA.res \data

AT dataset

./kmc -k28 -m12 -t12 -ci2 -fm at.fastq NA.res \data

./kmc -k40 -m12 -t12 -ci2 -fm at.fastq NA.res \data

./kmc -k55 -m12 -t12 -ci2 -fm at.fastq NA.res \data

./kmc -k65 -m12 -t12 -ci2 -fm at.fastq NA.res \data

./kmc -k100 -m12 -t12 -ci2 -fm at.fastq NA.res \data

./kmc -k125 -m12 -t12 -ci2 -fm at.fastq NA.res \data

./kmc -k150 -m12 -t12 -ci2 -fm at.fastq NA.res \data

./kmc -k175 -m12 -t12 -ci2 -fm at.fastq NA.res \data

./kmc -k200 -m12 -t12 -ci2 -fm at.fastq NA.res \data

Gzip

./kmc -k28 -m12 -t12 -ci2 @fv.txt NA.res \data

./kmc -k55 -m12 -t12 -ci2 @fv.txt NA.res \data

./kmc -k28 -m12 -t12 -ci2 @dm.txt NA.res \data

./kmc -k55 -m12 -t12 -ci2 @dm.txt NA.res \data

./kmc -k28 -m12 -t12 -ci2 @mb.txt NA.res \data

./kmc -k55 -m12 -t12 -ci2 @mb.txt NA.res \data

./kmc -k28 -m12 -t12 -ci2 @hs1.txt NA.res \data

./kmc -k55 -m12 -t12 -ci2 @hs1.txt NA.res \data

./kmc -k28 -m12 -t12 -ci2 @hs2.txt NA.res \data

./kmc -k55 -m12 -t12 -ci2 @hs2.txt NA.res \data

6. **KCMBT 1.0**

The tool is executed for eight threads for the FV and MB datasets. Its performance degraded with 12 threads because it requires more memory compared to execution on eight threads on our test machine. For the MB dataset, two threads gave the best result, and for the HS1 and HS2 datasets, readings are taken with a single thread owing to its large memory requirements.

./kcmbt -k 28 -t 8 -i fv.fastq

./kcmbt -k 28 -t 8 -i dm.fastq

./kcmbt -k 28 -t 2 -i mb.fastq

./kcmbt -k 28 -t 1 -i hs1.fastq

./kcmbt -k 28 -t 1 -i hs2.fastq

Dump Subroutine

./kcmbt_dump 8

7. **MSPKmerCounter 0.1**

java -jar ./Partition.jar -in fv.fastq -k 28 -L 353 -NB 1000 -p 10 -t 12

java -jar ./Count32.jar -t 12 -k 28 -NB 1000

java -jar ./Partition.jar -in fv.fastq -k 55 -L 353 -NB 1000 -p 10 -t 12

java -jar ./Count64.jar -t 12 -k 55 -NB 1000

java -jar ./Partition.jar -in dm.fastq -k 28 -L 76 -NB 1000 -p 10 -t 12

java -jar ./Count32.jar -t 12 -k 28 -NB 1000

java -jar ./Partition.jar -in dm.fastq -k 55 -L 76 -NB 1000 -p 10 -t 12

java -jar ./Count64.jar -t 12 -k 55 -NB 1000

java -jar ./Partition.jar -in mb.fastq -k 28 -L 100 -NB 1000 -p 10 -t 12

java -jar ./Count32.jar -t 12 -k 28 -NB 1000

java -jar ./Partition.jar -in mb.fastq -k 55 -L 100 -NB 1000 -p 10 -t 12

java -jar ./Count64.jar -t 12 -k 55 -NB 1000

java -jar ./Partition.jar -in hs1.fastq -k 28 -L 151 -NB 1000 -p 10 -t 12

java -jar ./Count32.jar -t 12 -k 28 -NB 1000

java -jar ./Partition.jar -in hs1.fastq -k 55 -L 151 -NB 1000 -p 10 -t 12

java -jar ./Count64.jar -t 12 -k 55 -NB 1000

java -jar ./Partition.jar -in hs2.fastq -k 28 -L 100 -NB 1000 -p 10 -t 12

java -jar ./Count32.jar -t 12 -k 28 -NB 1000

java -jar ./Partition.jar -in hs2.fastq -k 55 -L 100 -NB 1000 -p 10 -t 12

java -jar ./Count64.jar -t 12 -k 55 -NB 1000

Histogram Subroutine

java -jar Stat64.jar -k 28 -NB 353 -m 30000 -b 10000

java -jar Stat64.jar -k 28 -NB 353 -m 30000 -b 10000

java -jar Dump64.jar -k 28 -NB 1000 -t 12

java -jar Dump64.jar -k 55 -NB 1000 -t 12

cat * > dump_28

cat * > dump_55

AT dataset

java -jar ./Partition.jar -in at.fastq -k 28 -L 4805 -NB 1000 -p 10 -t 12

java -jar ./Count64.jar -t 12 -k 28 -NB 1000

java -jar ./Partition.jar -in at.fastq -k 40 -L 4805 -NB 1000 -p 10 -t 12

java -jar ./Count64.jar -t 12 -k 28 -NB 1000

java -jar ./Partition.jar -in at.fastq -k 55 -L 4805 -NB 1000 -p 10 -t 12

java -jar ./Count64.jar -t 12 -k 28 -NB 1000

java -jar ./Partition.jar -in at.fastq -k 65 -L 4805 -NB 1000 -p 10 -t 12

java -jar ./Count64.jar -t 12 -k 28 -NB 1000

java -jar ./Partition.jar -in at.fastq -k 100 -L 4805 -NB 1000 -p 10 -t 12

java -jar ./Count64.jar -t 12 -k 28 -NB 1000

java -jar ./Partition.jar -in at.fastq -k 125 -L 4805 -NB 1000 -p 10 -t 12

java -jar ./Count64.jar -t 12 -k 28 -NB 1000

java -jar ./Partition.jar -in at.fastq -k 150 -L 4805 -NB 1000 -p 10 -t 12

java -jar ./Count64.jar -t 12 -k 28 -NB 1000

java -jar ./Partition.jar -in at.fastq -k 175 -L 4805 -NB 1000 -p 10 -t 12

java -jar ./Count64.jar -t 12 -k 28 -NB 1000

java -jar ./Partition.jar -in at.fastq -k 200 -L 4805 -NB 1000 -p 10 -t 12

java -jar ./Count64.jar -t 12 -k 28 -NB 1000

8. **aTurtle 0.3**

./aTurtle32 -f fv.fastq -o fv_28 -k 28 -q -n 800000000

./aTurtle64 -f fv.fastq -o fv_55 -k 55 -q -n 2100000000

./aTurtle32 -f dm.fastq -o dm_28 -k 28 -q -n 420000000

./aTurtle64 -f dm.fastq -o dm_55 -k 55 -q -n 380000000

./aTurtle32 -f mb.fastq -o mb_28 -k 28 -q -n 2100000000

./aTurtle64 -f mb.fastq -o mb_55 -k 55 -q -n 3000000000

./aTurtle32 -f hs1.fastq -o hs1_28 -k 28 -q -n 24000000000

./aTurtle64 -f hs1.fastq -o hs1_55 -k 55 -q -n 26000000000

./aTurtle32 -f hs2.fastq -o hs2_28 -k 28 -q -n 8300000000

./aTurtle64 -f hs2.fastq -o hs2_55 -k 55 -q -n 9000000000

9. **GenomeTester4**

./glistmaker fv.fastq -w 28 --num_threads 12 -c 2 -o fv_28

./glistmaker dm.fastq -w 28 --num_threads 12 -c 2 -o dm_28

./glistmaker mb.fastq -w 28 --num_threads 12 -c 2 -o mb_28

./glistmaker hs1.fastq -w 28 --num_threads 12 -c 2 -o hs1_28

./glistmaker hs2.fastq -w 28 --num_threads 12 -c 2 -o hs2_28

Dump Subroutine

./glistquery fv_28_28.list >> dump_28

./glistquery dm_28_28.list >> dump_28

10. **BFCounter 1.0**

./BFCounter count -k 28 -n 400000000 -t 12 -o fv_28 fv.fastq

./BFCounter count -k 55 -n 400000000 -t 12 -o fv_55 fv.fastq

./BFCounter count -k 28 -n 250000000 -t 12 -o dm_28 dm.fastq

./BFCounter count -k 55 -n 250000000 -t 12 -o dm_55 dm.fastq

./BFCounter count -k 28 -n 1100000000 -t 12 -o mb_28 mb.fastq

./BFCounter count -k 55 -n 1100000000 -t 12 -o mb_55 mb.fastq

./BFCounter count -k 28 -n 3000000000 -t 12 -o hs1_28 hs1.fastq

./BFCounter count -k 55 -n 3000000000 -t 12 -o hs1_55 hs1.fastq

./BFCounter count -k 28 -n 3000000000 -t 12 -o hs2_28 hs2.fastq

./BFCounter count -k 55 -n 3000000000 -t 12 -o hs2_55 hs2.fastq

Dump Subroutine

./BFCounter dump -k 28 -i fv_28 -o dump_28

./BFCounter dump -k 28 -i fv_55 -o dump_55

Gzip

./BFCounter count -k 28 -n 400000000 -t 12 -o fv_28 SRR072005.fastq.gz SRR072006.fastq.gz SRR072007.fastq.gz SRR072008.fastq.gz SRR072009.fastq.gz SRR072010.fastq.gz SRR072011.fastq.gz SRR072012.fastq.gz SRR072013.fastq.gz SRR072014.fastq.gz SRR072029.fastq.gz

./BFCounter count -k 55 -n 400000000 -t 12 -o fv_55 SRR072005.fastq.gz SRR072006.fastq.gz SRR072007.fastq.gz SRR072008.fastq.gz SRR072009.fastq.gz SRR072010.fastq.gz SRR072011.fastq.gz SRR072012.fastq.gz SRR072013.fastq.gz SRR072014.fastq.gz SRR072029.fastq.gz

./BFCounter count -k 28 -n 250000000 -t 12 -o dm_28 SRR097732_1.fastq.gz SRR097732_2.fastq.gz

./BFCounter count -k 55 -n 250000000-t 12 -o dm_55 SRR097732_1.fastq.gz SRR097732_2.fastq.gz

./BFCounter count -k 28 -n 3000000000 -t 12 -o hs1_28 NA12878D_HiSeqX_R1.fastq.gz

NA12878D_HiSeqX_R2.fastq.gz

./BFCounter count -k 55 -n 3000000000 -t 12 -o hs1_55 NA12878D_HiSeqX_R1.fastq.gz

NA12878D_HiSeqX_R2.fastq.gz

./BFCounter count -k 28 -n 3000000000 -t 12 -o hs2_28 ERR024163_1.fastq.gz ERR024163_2.fastq.gz ERR024164_1.fastq.gz ERR024164_2.fastq.gz ERR024165_1.fastq.gz ERR024165_2.fastq.gz ERR024166_1.fastq.gz ERR024166_2.fastq.gz ERR024167_1.fastq.gz ERR024167_2.fastq.gz ERR024168_1.fastq.gz ERR024168_2.fastq.gz ERR024169_1.fastq.gz ERR024169_2.fastq.gz ERR024170_1.fastq.gz ERR024170_2.fastq.gz ERR024171_1.fastq.gz ERR024171_2.fastq.gz ERR024172_1.fastq.gz ERR024172_2.fastq.gz ERR024173_1.fastq.gz ERR024173_2.fastq.gz ERR024174_1.fastq.gz ERR024174_2.fastq.gz ERR024175_1.fastq.gz ERR024175_2.fastq.gz ERR024176_1.fastq.gz ERR024176_2.fastq.gz ERR024177_1.fastq.gz ERR024177_2.fastq.gz ERR024178_1.fastq.gz ERR024178_2.fastq.gz ERR024179_1.fastq.gz ERR024179_2.fastq.gz ERR024180_1.fastq.gz ERR024180_2.fastq.gz ERR024181_1.fastq.gz ERR024181_2.fastq.gz ERR024182_1.fastq.gz ERR024182_2.fastq.gz ERR024183_1.fastq.gz ERR024183_2.fastq.gz ERR024184_1.fastq.gz ERR024184_2.fastq.gz ERR024185_1.fastq.gz ERR024185_2.fastq.gz ERR024186_1.fastq.gz ERR024186_2.fastq.gz

./BFCounter count -k 55 -n 3000000000 -t 12 -o hs2_55 ERR024163_1.fastq.gz ERR024163_2.fastq.gz ERR024164_1.fastq.gz ERR024164_2.fastq.gz ERR024165_1.fastq.gz ERR024165_2.fastq.gz ERR024166_1.fastq.gz ERR024166_2.fastq.gz ERR024167_1.fastq.gz ERR024167_2.fastq.gz ERR024168_1.fastq.gz ERR024168_2.fastq.gz ERR024169_1.fastq.gz ERR024169_2.fastq.gz ERR024170_1.fastq.gz ERR024170_2.fastq.gz ERR024171_1.fastq.gz ERR024171_2.fastq.gz ERR024172_1.fastq.gz ERR024172_2.fastq.gz ERR024173_1.fastq.gz ERR024173_2.fastq.gz ERR024174_1.fastq.gz ERR024174_2.fastq.gz ERR024175_1.fastq.gz ERR024175_2.fastq.gz ERR024176_1.fastq.gz ERR024176_2.fastq.gz ERR024177_1.fastq.gz ERR024177_2.fastq.gz ERR024178_1.fastq.gz ERR024178_2.fastq.gz ERR024179_1.fastq.gz ERR024179_2.fastq.gz ERR024180_1.fastq.gz ERR024180_2.fastq.gz ERR024181_1.fastq.gz ERR024181_2.fastq.gz ERR024182_1.fastq.gz ERR024182_2.fastq.gz ERR024183_1.fastq.gz ERR024183_2.fastq.gz ERR024184_1.fastq.gz ERR024184_2.fastq.gz ERR024185_1.fastq.gz ERR024185_2.fastq.gz ERR024186_1.fastq.gz ERR024186_2.fastq.gz

**Results**

Additional results that are not included in the main part of the paper are given below.

**Figure 1.** Analysis of disk utilization (GB) of disk-based approaches at *k* = 28 and 55

With disk-based approaches, disk utilization decreases when *k* increases (Figure S1). This is because as *k* increases, the number of *k*-mers/super *k*-mers becomes lower.

**Table S7** Comparison of scalability of *k*-mer counting tools for different numbers of threads for the FV dataset at *k* = 28

| **Time (seconds)** | | | | | | | | | |
| --- | --- | --- | --- | --- | --- | --- | --- | --- | --- |
| **No. of threads** | **KMC3** | **Gerbil 1.0** | **GenomeTester4** | **KCMBT 1.0** | **MSPKmerCounter 0.1** | **KAnalyze 2.0.0** | **DSK 2.2.0** | **Jellyfish 2.2.6** | **BFCounter 1.0** |
| 1 | 273 | 91 | 410 | 175 | 52 | 3307 | 407 | 1152 | 3065 |
| 2 | 183 | 92 | 233 | 119 | 41.5 | 3072 | 211 | 582 | 2595 |
| 4 | 80 | 92 | 209 | 70 | 38.5 | 2394 | 122 | 326 | 2175 |
| 6 | 54 | 63 | 208 | 61 | 47 | 2214 | 83 | 252 | 1995 |
| 8 | 42 | 51 | 211 | 55 | 55 | 2080 | 69 | 201 | 1888 |
| 12 | 38.66 | 33.66 | 214 | 51 | 59.33 | 2042 | 56.33 | 138.33 | 1731 |
| **Memory usage (GB)** | | | | | | | | | |
| **No. of threads** | **KMC3** | **Gerbil 1.0** | **GenomeTester4** | **KCMBT 1.0** | **MSPKmerCounter 0.1** | **KAnalyze 2.0.0** | **DSK 2.2.0** | **Jellyfish 2.2.6** | **BFCounter 1.0** |
| 1 | 6 | 0.71 | 21 | 9 | 4.07 | 2 | 4 | 7 | 3 |
| 2 | 6 | 0.71 | 26 | 14 | 4.07 | 2 | 3 | 7 | 3 |
| 4 | 6 | 0.71 | 26 | 21 | 4.07 | 4 | 2 | 7 | 3 |
| 6 | 6 | 0.73 | 26 | 31 | 4.19 | 6 | 2 | 7 | 3 |
| 8 | 6 | 0.76 | 26 | 36 | 4.34 | 8 | 3 | 7 | 3 |
| 12 | 7.66 | 0.82 | 26 | 43 | 4.45 | 9.3 | 6.35 | 7.9 | 3 |
| **CPU utilization (%)** | | | | | | | | | |
| **No. of threads** | **KMC3** | **Gerbil 1.0** | **GenomeTester4** | **KCMBT 1.0** | **MSPKmerCounter 0.1** | **KAnalyze 2.0.0** | **DSK 2.2.0** | **Jellyfish 2.2.6** | **BFCounter 1.0** |
| 1 | 103.83 | 354.54 | 99.54 | 99.46 | 133.30 | 294.21 | 99.7 | 99 | 99.71 |
| 2 | 159.78 | 353.55 | 179.98 | 191.78 | 233.76 | 298.40 | 192.01 | 198.1 | 138.62 |
| 4 | 362.48 | 352.15 | 204.88 | 359.83 | 337.19 | 377.23 | 353.56 | 383.9 | 186.67 |
| 6 | 533.79 | 573.53 | 202.55 | 485.90 | 433.86 | 441.04 | 515.51 | 551.15 | 220.26 |
| 8 | 719.56 | 746.21 | 203.11 | 542.25 | 549.33 | 492.35 | 688.66 | 723.89 | 243.33 |
| 12 | 998.08 | 1110.38 | 202.33 | 785.62 | 811.69 | 509.20 | 866.5 | 1093.55 | 274.05 |
| **Disk usage (GB)** | | | | | | | | | |
| **No. of threads** | **KMC3** | **Gerbil 1.0** | **GenomeTester4** | **KCMBT 1.0** | **MSPKmerCounter 0.1** | **KAnalyze 2.0.0** | **DSK 2.2.0** | **Jellyfish 2.2.6** | **BFCounter 1.0** |
| 1 | 2 | 2 | - | - | 1 | 22.2 | 6 | - | - |
| 2 | 2 | 2 | - | - | 1 | 22.2 | 6 | - | - |
| 4 | 2 | 2 | - | - | 1 | 22.2 | 6 | - | - |
| 6 | 2 | 2 | - | - | 1 | 22.2 | 6 | - | - |
| 8 | 2 | 2 | - | - | 1 | 22.2 | 6 | - | - |
| 12 | 4 | 4 | - | - | 1 | 22.2 | 6 | - | - |

**Table S8** Comparison of scalability of *k*-mer counting tools for different numbers of threads for the MB dataset at *k* = 28

| **Time (seconds)** | | | | | | | | | | | | | | | | | |
| --- | --- | --- | --- | --- | --- | --- | --- | --- | --- | --- | --- | --- | --- | --- | --- | --- | --- |
| **No. of threads** | | **KMC3** | | | **Gerbil 1.0** | | | **GenomeTester4** | **KCMBT 1.0** | **MSPKmerCounter 0.1** | **KAnalyze 2.0.0** | **DSK 2.2.0** | | **Jellyfish 2.2.6** | | | **BFCounter 1.0** |
| 1 | | 2959 | | | 2372 | | | 6101 | 1699 | 13209 | - | 5080 | | 9782 | | | 34291 |
| 2 | | 2357 | | | 2383 | | | 3495 | 1644 | 12146 | - | 3159 | | 4871 | | | 31278 |
| 4 | | 2032 | | | 2338 | | | 3475 | 1718 | 11709 | - | 3105 | | 2492 | | | 27932 |
| 6 | | 1988 | | | 2147 | | | 3528 | - | 10771 | - | 3295 | | 1592 | | | 25608 |
| 8 | | 1983 | | | 2160 | | | 3573 | - | 10264 | - | 3418 | | 1461 | | | 23091 |
| 12 | | 2019 | | | 2238 | | | 3520 | - | 11094 | 51422 | 3358 | | 1467 | | | 18950 |
| **Memory usage (GB)** | | | | | | | | | | | | | | | | | |
| **No. of threads** | **KMC3** | | **Gerbil 1.0** | | | | **GenomeTester4** | | **KCMBT 1.0** | **MSPKmerCounter 0.1** | **KAnalyze 2.0.0** | **DSK 2.2.0** | | **Jellyfish 2.2.6** | | | **BFCounter 1.0** |
| 1 | 8 | | 2 | | | | 59 | | 14 | 6.11 | - | 3 | | 15 | | | 10 |
| 2 | 8 | | 2 | | | | 58 | | 34 | 6.30 | - | 5 | | 15 | | | 10 |
| 4 | 11 | | 2 | | | | 58 | | 53 | 6.67 | - | 10 | | 15 | | | 10 |
| 6 | 11 | | 2 | | | | 58 | | - | 6.78 | - | 10 | | 15 | | | 10 |
| 8 | 10 | | 2 | | | | 58 | | - | 7.12 | - | 10 | | 15 | | | 10 |
| 12 | 9 | | 2 | | | | 60 | | - | 8 | 10 | 12 | | 15 | | | 10 |
| **CPU utilization (%)** | | | | | | | | | | | | | | | | | |
| **No. of threads** | **KMC3** | | | **Gerbil 1.0** | | | | **GenomeTester4** | **KCMBT 1.0** | **MSPKmerCounter 0.1** | **KAnalyze 2.0.0** | **DSK 2.2.0** | | **Jellyfish 2.2.6** | | | **BFCounter 1.0** |
| 1 | 102.18 | | | 194.76 | | | | 89.9126 | 98.74 | 133.73 | - | 91.08 | | 99.525 | | | 96.73 |
| 2 | 135.45 | | | 192.18 | | | | 163.28 | 135.86 | 164.97 | - | 159.79 | | 198.14 | | | 131.88 |
| 4 | 220.65 | | | 199.18 | | | | 164.74 | 132.50 | 232.06 | - | 193.23 | | 392.16 | | | 178.55 |
| 6 | 243.87 | | | 241.72 | | | | 164.85 | - | 284.45 | - | 197.52 | | 580.22 | | | 217.31 |
| 8 | 249.42 | | | 251.16 | | | | 160.54 | - | 336.60 | - | 182.96 | | 760.98 | | | 237.10 |
| 12 | 216.92 | | | 269.52 | | | | 153.67 | - | 316.89 | 279.40 | 185.08 | | 800.12 | | | 300.36 |
| **Disk usage (GB)** | | | | | | | | | | | | | | | | | |
| **No. of threads** | **KMC3** | | **Gerbil 1.0** | | | **GenomeTester4** | | | **KCMBT 1.0** | **MSPKmerCounter 0.1** | **KAnalyze 2.0.0** | | **DSK 2.2.0** | | **Jellyfish 2.2.6** | **BFCounter 1.0** | |
|  | 36.3 | | 31.7 | | | - | | | - | 173 | - | | 59 | | - | - | |
| 2 | 36.3 | | 31.7 | | | - | | | - | 173 | - | | 59 | | - | - | |
| 4 | 36.3 | | 31.7 | | | - | | | - | 173 | - | | 59 | | - | - | |
| 6 | 36.3 | | 31.7 | | | - | | | - | 173 | - | | 59 | | - | - | |
| 8 | 36.3 | | 31.7 | | | - | | | - | 173 | - | | 59 | | - | - | |
| 12 | 36.3 | | 32 | | | - | | | - | 173 | 189 | | 59 | | - | - | |

**Disk and CPU utilization for multiple threads**

Disk utilization is not affected by the number of threads (Table S7–S8), but for each program, there is a visible impact of the number of threads on CPU utilization (except for KAnalyze and BFCounter for the FV dataset). Jellyfish, on the other hand, has better scalability (in terms of CPU utilization) to the number of threads.

**References:**

1.	Chikhi R, Rizk G. Space-efficient and exact de Bruijn graph representation based on a Bloom filter. Algorithms Mol Biol 2013;**8**(1):1.

2.	Vinga S, Almeida J. Alignment-Free Sequence Comparison–A Review. Bioinformatics 2003;**19**(4):513–23.`
